# Asymmetric Dearomatization of Phthalazines by Anion-Binding Catalysis

**DOI:** 10.1021/acs.orglett.3c03325

**Published:** 2023-12-01

**Authors:** Marta Velázquez, Rosario Fernández, José M. Lassaletta, David Monge

**Affiliations:** † Departamento de Química Orgánica, Facultad de Química, Universidad de Sevilla and Centro de Innovación en Química Avanzada (ORFEO-CINQA), C/Prof. García González, 1, 41012 Sevilla, Spain; ‡ Instituto de Investigaciones Químicas (CSIC-US) and Centro de Innovación en Química Avanzada (ORFEO-CINQA), Avenida Américo Vespucio, 49, 41092 Sevilla, Spain

## Abstract

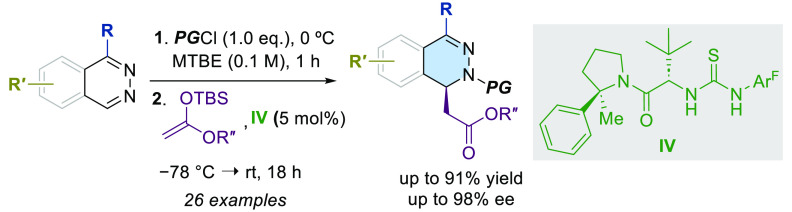

A straightforward methodology for the enantioselective synthesis of 1,2-dihydrophthalazines *via* dearomatization of phthalazines by anion-binding catalysis has been developed. The process involves the Mannich-type addition of silyl ketene acetals to *in situ* generated *N*-acylphthalazinium chlorides using a *tert*-leucine derived thiourea as a H-bond donor catalyst. Ensuing selective and high-yielding transformations provide appealing dihydro- and tetrahydro-phthalazines, phthalazones, and piperazic acid homologues, en route to biologically relevant molecules.

Benzodiazines are important bioactive heterocycles with a wide range of applications in the pharmaceutical and agrochemical fields.^1^ Among them, phthalazine (2,3-diazanaphthalene) and dihydrophthalazine derivatives are probably the most privileged pharmacophores (Figure 1).^2^ For example, azelastine (a phthalazone) is an efficient histamine antagonist approved for the treatment of allergic rhinitis^3^ and dihydrophthalazine SYM2206 is an AMPA receptor modulator with anticonvulsant activity.^4^ Chiral racemic dihydrophthalazines BAL0030543^5^ and (*S*)-RAB1 and their analogues^6^ are potent dihydrofolate reductase inhibitors that have demonstrated activity against antibiotic-resistant strains of *Staphylococcus aureus*, among other Gram-positive bacteria.

**Figure 1:**
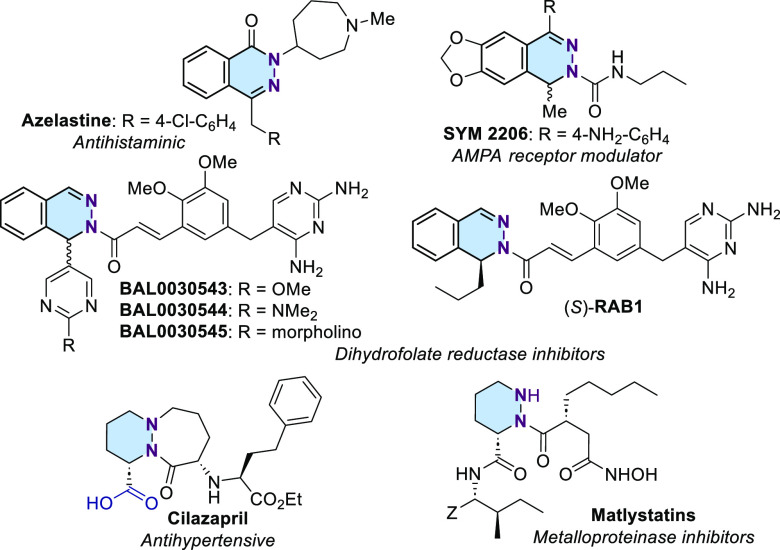
Selective bioactive diazaheterocycles.

On the other hand, monocyclic derivatives have also shown interesting bioactivities. Selected examples are cilazapril, a marketed saturated pyridazine (piperazic acid derivative) drug for the treatment of hypertension,^7^ and matlystatins, a group of potent metalloproteinase inhibitors.^8^ However, their potential as therapeutic weapons contrasts with the scarcity of methodologies for accessing these chiral molecules in an enantioselective fashion. In this regard, a straightforward approach consists of the stereoselective dearomatization of phthalazin-2-ium salts derived from readily available phthalazines.

Several examples demonstrate the use of phthalazinium dicyanomethanides as stable azomethine ylides in [3 + 3] and [3 + 2] cycloaddition reactions. These reactions are enabled by Cu(I) and iminium catalysis, respectively (Scheme 1A).^9^ Furthermore, an *N*-alkyl phthalazinium iodide was subjected to enamine catalysis for a nucleophilic dearomatization reaction in an intramolecular version. However, only a single example was reported (Scheme 1B).^10^ Herein, we report the asymmetric synthesis of 1,2-dihydrophthalazines through dearomatization of phthalazines via anion-binding catalysis using H-bond donor organocatalysts (Scheme 1C). The process involves the nucleophilic addition of silyl ketene acetals to *in situ* generated *N*-acyl-phthalazinium halides.

**Scheme 1:**
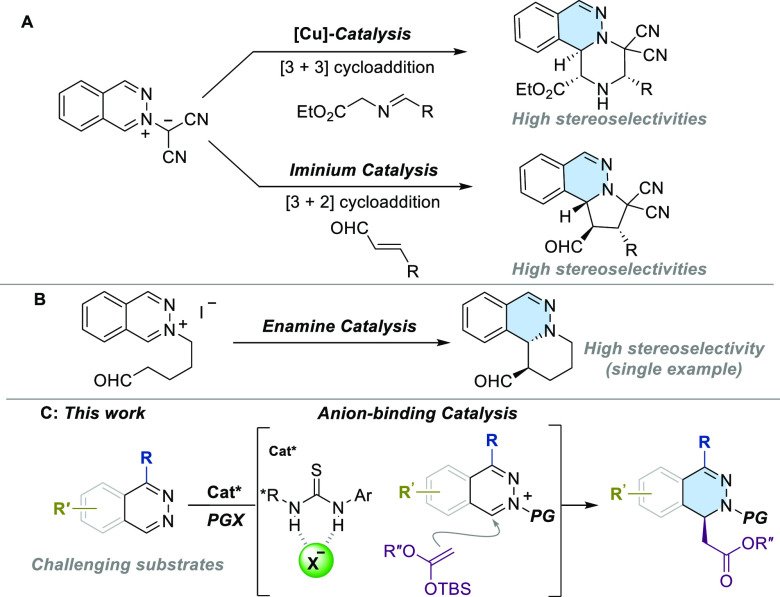
Catalytic Asymmetric Dearomatization of Phthalazines

Pioneered by Jacobsen,^11^ anion-binding catalysis^12^ has enabled a wide range of nucleophilic dearomatizations of mono aza-heterocycles, such as isoquinolines, quinolines, and pyridines.^13^ However, attempts to implement these methodologies in diaza-heterocycles remain challenging due to the increased complexity associated with the presence of two nitrogens prone to be acylated, resulting in multiple reactive positions and thereby lower yields and side reactions. Moreover, enantioselectivities have been often compromised. To the best of our knowledge, only quinazolines (1,3-diazanaphthalenes) have been efficiently dearomatized with high enantioselectivities by asymmetric anion-binding catalysis. Employing chiral triazoles (CH-bond donors), García Mancheño and co-workers successfully developed the dearomatization of quinazoline through a nucleophilic C2-addition of silyl ketene acetals (up to 92% ee), while phthalazine and pyridazine underwent dearomatization reactions with moderate enantioselectivities.^14^ It is worth mentioning that alternative metal-catalyzed dearomatization of pyridazine also remains virtually unknown.

Preliminary experiments were conducted employing phthalazine (**1a**) as a model substrate, 2,2,2-trichloroethyl chloroformate (TrocCl) as acylating reagent, and isopropyl TBS-ketene acetal **2a** as the nucleophile. The model reaction was initially studied in methyl *tert-*butyl ether (MTBE) employing a temperature gradient (from −78 °C to room temperature over 18 h). Although a quite insoluble phthalazinium salt was generated, a significant background reaction took place in the absence of any catalyst (entry 1, Table 1). From the initial screening of hydrogen-bond donor (HBD) organocatalysts (see Supporting Information), *tert*-leucine derived thiourea **I** emerged as the most promising candidate, affording (*S*)-**3aa** in 57% NMR-yield and 81% ee (entry 2). Organocatalyst **II**, featuring a combination of *tert*-leucine and (1*R*,2*R*)-1,2-diaminocyclohexane scaffolds similar to the optimal catalyst originally reported for the dearomatization of isoquinolines,^11^ displayed almost no stereocontrol (entry 3). Next, HBDs **III**-**VII**, bearing the more rigid 2-substituted pyrrolidino amide motifs, were evaluated (entries 4–8). In general, these catalysts exhibited good catalytic activities, achieving a maximum yield of 75% with thiourea **IV**. In accordance with a better conformational control exerted by the 2-methyl-2-phenylpyrrolidine scaffold,^15^ (*S*)-**3aa** was obtained in 89% ee employing either thiourea **IV** or urea **V** (entries 5 and 6). Remarkably, catalyst **VII**, featuring a more extended π moiety, afforded **3aa** as a racemic mixture (entry 8). Other ethereal solvents (Et_2_O or THF) and toluene were tolerated (entries 9–12). However, none of them overcame the results obtained with MTBE. The influence of the acylating reagent on the reactivity and selectivity was further investigated (Scheme 2). In the chlorinated series, 2-chloroethoxycarbonyl-protected adduct **4aa** was obtained in lower enantioselectivity (66% ee), while only traces of product were formed with 1,1,1-trichloromethoxycabonyl chloride **5aa**. Phthalazines **6aa** and **7aa**, bearing Cbz and Ac protecting groups, were isolated in 75 and 98% yields, albeit with lower enantioselectivities (55 and 64%, respectively). To our delight, Bz-protected adduct **8aa** was obtained with essentially the same enantioselectivity than Troc-**3aa** (88% ee) and a better yield (87%). Other substitution patterns in the phenyl ring [**9aa** (*o*-Cl), **10aa** (*p*-Cl), **11aa** (*p*-NO_2_), and **12aa** (*p*-Me)] did not improve the enantioselectivity of the process (up to 86% ee for **10aa**). According to an anion-binding activation mode, the nature of the halide anion modified the reaction outcome. Hence, product **8aa** was isolated in lower enantioselectivity from reactions performed using benzoyl bromide or fluoride as the acylating reagent (73% and 30% ee, respectively). With the optimal conditions in hand, the catalyst loading could be reduced to 5 mol % without compromising yield or enantioselectivity. Next, the reactions of **1a** and **2a**, employing BzCl as the acylating reagent, were investigated in more detail. A comparison of noncatalyzed *vs* catalyzed reaction kinetics revealed maximum differentiation at the beginning of the process (from −78 °C to −30 °C), affording **8aa** in 75% NMR-yield after 6 h (vs <5% in the background) (Figure 2). Moreover, it was observed that the enantioselectivity was slightly lower at the early stages (from −78 to −60 °C; 79% and 82% ee after 2 and 4 h, respectively), reaching a maximum value of 88% ee after 6 h, which remained constant until the end of the reaction. Therefore, a slow temperature gradient (from −78 °C to rt) was employed as optimal methodology for further studies (Scheme 3). Different nucleophiles were initially tested.

**Table 1:**
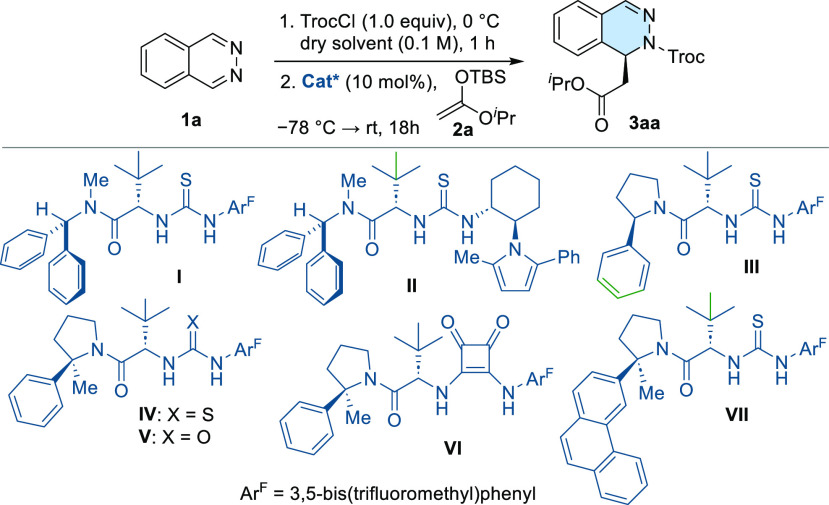

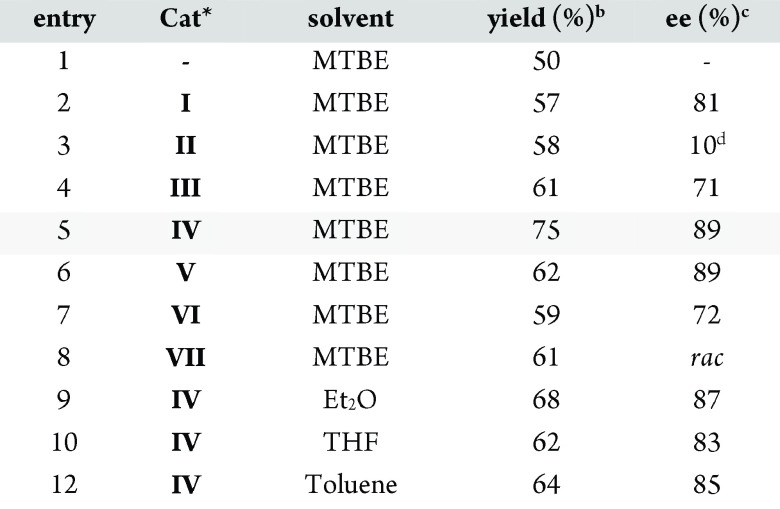
Screening of Organocatalysts and Optimization of the Reaction Parameters^a^

a Reactions performed at 0.1 mmol scale.

b Determined by ^1^H NMR using mesitylene as internal standard.

c Determined by HPLC.

d (*R*)-enantiomer.

**Scheme 2:**
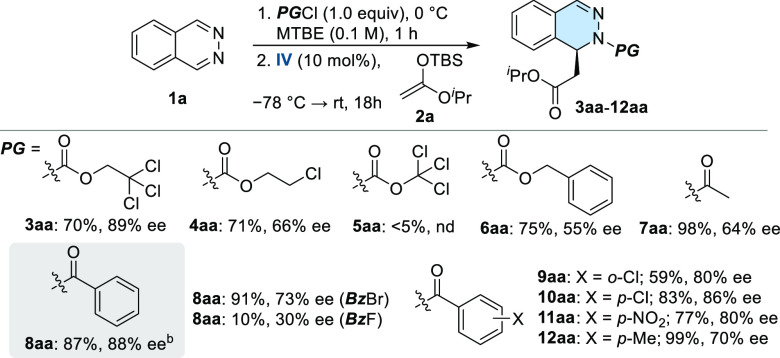
Optimization of Acylating Reagents (a) Reactions performed at 0.2 mmol scale. Yields given for isolated products after chromatography. ee’s were determined by HPLC on chiral stationary phases. (b) Reaction performed with 5 mol % of catalyst loading.

**Figure 2:**
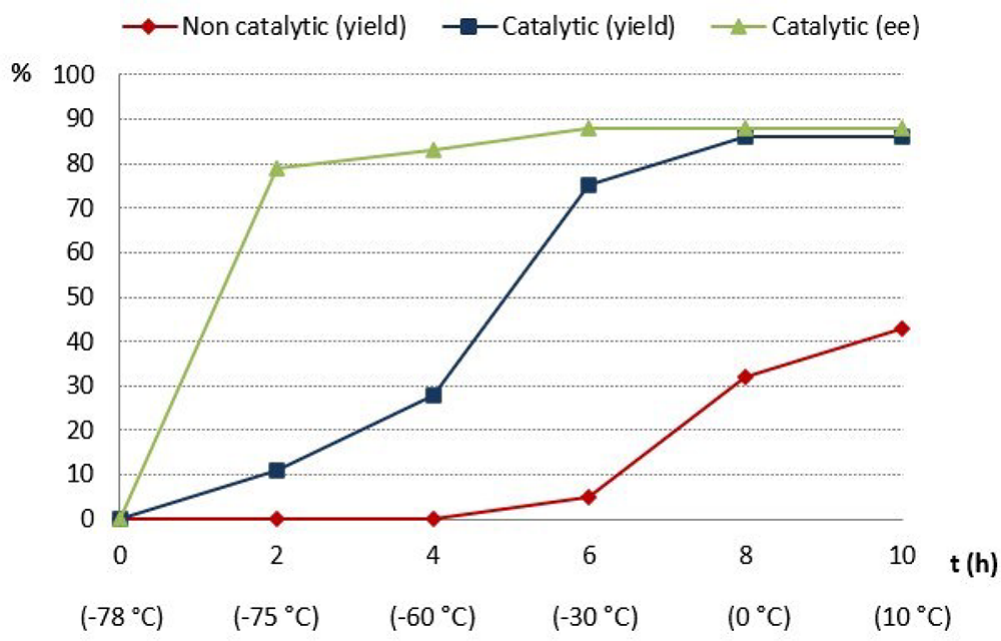
Noncatalyzed vs catalyzed reaction kinetics. Reactions were performed in MTBE with 5 mol % of catalyst from −78 to 10 °C.

**Scheme 3:**
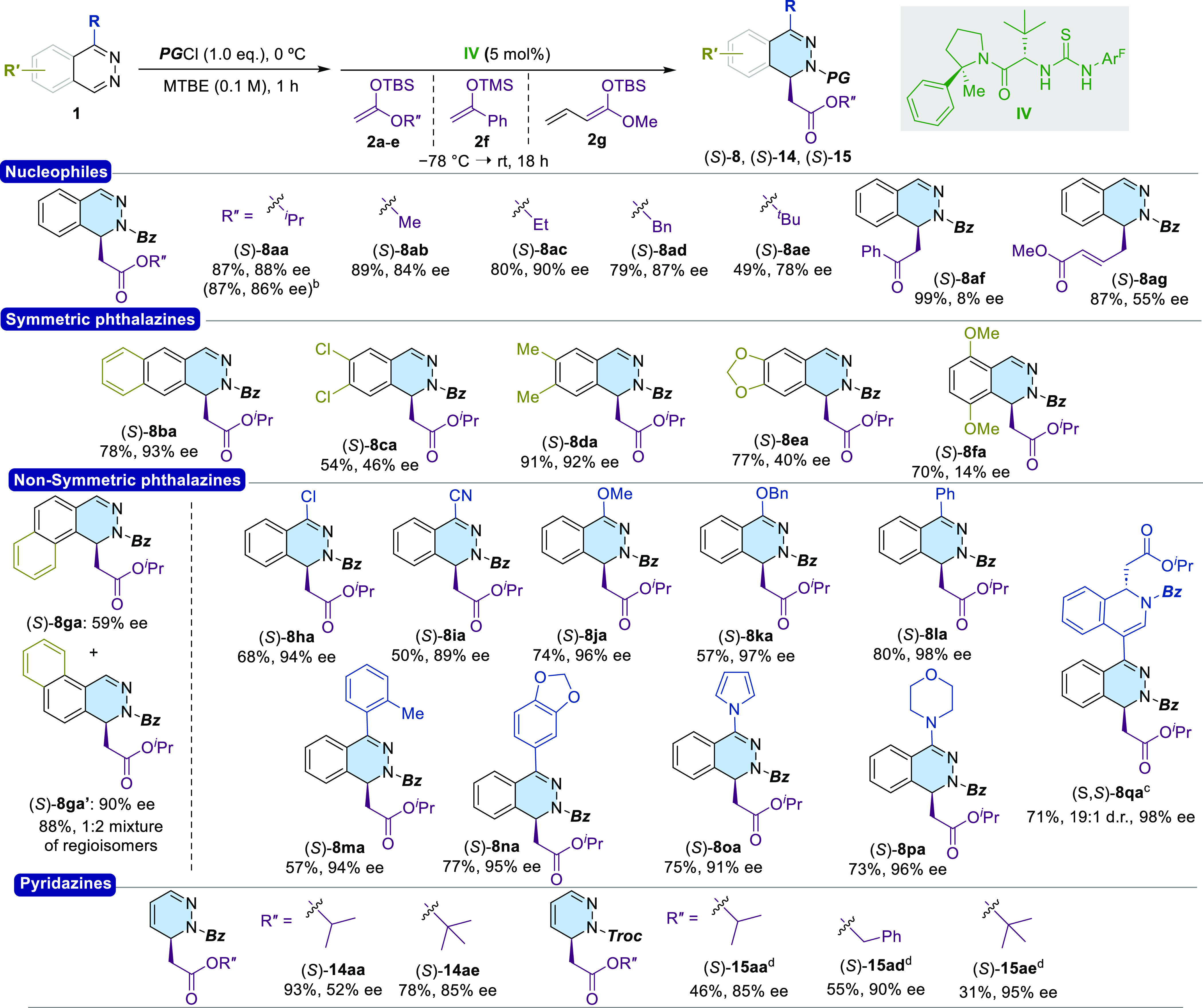
Substrate Scope (a) Reactions performed at 0.2 mmol scale. (b) Reaction performed at 1 mmol scale. (c) 2.5 equiv of **2a**. (d) 10 mol % of catalyst loading.

Silyl ketene acetals bearing isopropyl, methyl, ethyl, and benzyl ester moieties (**2a**–**d**) performed efficiently, affording dihydrophthalazines (*S*)-**8aa**–**8ad** in generally good yields (79–89%) and enantioselectivities (84–90% ee). However, the use of *tert*-butyl ketene acetal **2e** led to a moderate yield (49%) and lower enantioselectivity (78% ee).

Silyl enol ethers derived from acetone and cyclohexanone were not reactive in this transformation, while acetophenone-derived **2f** afforded **8af** in quantitative yield, albeit in low enantioselectivity (8% ee). Finally, vinylogous reagent **2g** was also tolerated, affording **8ag** in good yield (87%) with moderate enantioselectivity (55% ee). Next we carried out the dearomatization reaction of different phthalazines using **2a** as a representative nucleophile. Within the symmetric series, the product (*S*)-**8ba**, bearing an additional fused aromatic ring, was obtained in 93% ee. 6,7-Disubstitution was further evaluated: 6,7-dimethyl phthalazine (**1d**) proved to be also a suitable substrate, affording the corresponding product (*S*)-**8da** in excellent yield (91%) and enantioselectivity (92%). On the other hand, the introduction of chloro or alkoxy groups at these positions afforded products in poorer yields and enantioselectivities [(*S*)-**8ca**, 54%, 46% ee; (*S*)-**8ea**, 77%, 40% ee]. The introduction of oxygenated substituents near the reactive center had a negative impact in the enantioselectivity. Thus, the dearomatization of 5,8-dimethoxyphthalazine (**1f**) afforded (*S*)-**8fa** in 70% yield and 14% ee. Benzo[*f*]phthalazine **1g** afforded an inseparable mixture of regioisomers **8ga**/**8ga**′ (1:2) in good yield (88%) and moderate to excellent ee values (59 and 90% ee, respectively). More interestingly, C4-monosubstituted phthalazines afforded adducts (*S*)-**8ha**–**8pa** in moderate-to-good yields (50–80%) and excellent enantioselectivities (89–98% ee), regardless of the nature of the substituents: chloro, cyano, alkoxy groups, with various substitution patterns, heteroaryls (exemplified by R = pyrrole), and a saturated heterocycle (R = morpholine). Finally, the challenging substrate **1q**, bearing both phthalazine and isoquinoline scaffolds, was evaluated. Remarkably, a double dearomatization process involving the corresponding dibenzoyl bis-chloride allowed the generation of two stereogenic centers in (*S*,*S*)-**8qa** with excellent stereocontrol [19:1 dr, 98% ee (major)] and 71% yield. Finally, the methodology was extended to the dearomatization of pyridazine **13a**. Under the optimized conditions, dihydropyridazines (*S*)-**14aa** and (*S*)-**14ae** were obtained in high yields and moderate-to-good ee values of 52 and 85% ee, respectively. It′s worth noting that Bz-pyridazinium chloride was more soluble in MTBE than the corresponding Bz-phthalazinium salt, thereby favoring the competing background reaction (82% NMR-yield in absence of catalyst). Additionally, other solvents and fixed temperatures (−78 °C) were unsuccessfully tested (see Supporting Information). On the contrary, Troc-pyridazinium chloride was less soluble than Bz-protected one and (*S*)-**15aa**, (*S*)-**15ad**, and (*S*)-**15ae** were synthesized with superior enantioselectivities (85–95% ee), albeit in moderate yields (46%, 55%, and 31%, respectively) even when using 10 mol % of catalyst loadings. Remarkably, the best enantioselectivity in this case was obtained when combined with *tert*-butyl ketene acetal **2e**, in contrast with the trend observed in the dearomatization of **1a**. Finally, the synthetic usefulness of dihydrophthalazines **8** was demonstrated by accessing several selected targets (Scheme 4). Under standard hydrogenation conditions [Pd(C)/ 1 atm (H_2_)], **8aa** underwent a chemoselective hydrogenation of the C=N double bond to afford cyclic hydrazino ester (*S*)-**16**. Under the same conditions, (*S*)-**8ka** was efficiently transformed into the phthalazone derivative (*S*)-**17** in high yields and without noticeable racemization.

**Scheme 4:**
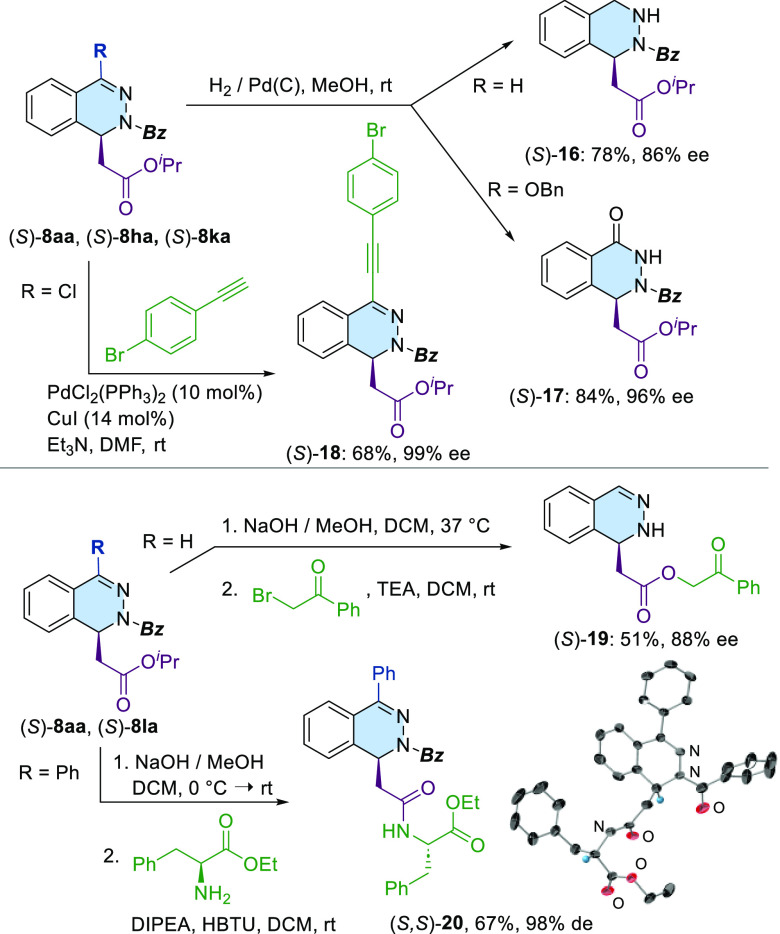
Transformations of Dihydrophthalazines **8**

Additionally, 4-chloro substituted **8ha** was subjected to Sonogashira coupling to yield product (*S*)-**18** (77%). To illustrate the synthetic potential of dihydrophthalazines **8** as cyclic β-hydrazino acid precursors, hydrolysis of both the ^*i*^Pr-ester and *Bz*-amide was performed under basic conditions [NaOH (3M), 37 °C]. Subsequent chemoselective *O*-alkylation through S_N_2 of 2-bromoacetophenone yielded **19** without compromising the stereochemical integrity of the starting material (**8aa**). Additionally, milder basic conditions [NaOH (1M), from 0 °C to rt] allowed selective saponification of the ^*i*^Pr-ester. Peptide-type coupling of the corresponding carboxylic acid with enantiopure l-phenylalanine ethyl ester, promoted by HBTU, afforded (*S,S*)-**20** in good overall yield (67%, 2 steps) and without a loss of enantiomeric purity (98% of diastereomeric excess). Crystals of (*S,S*)-**20** suitable for X-ray diffraction analysis served to determine the absolute *S* configuration of the newly created stereogenic center in (*S*)-**8la**. Within the Troc-protected series, the absolute *S* configuration of dihydrophthalazine **3aa** and dihydropyridazine **15aa** was assigned by a chemical correlation. Assuming a uniform stereochemical pathway, the absolute configurations of all other products were assigned by analogy.

In summary, anion-binding catalysis has enabled a three-component enantioselective dearomatization reaction of phthalazines, employing benzoyl chloride as the optimal acylating reagent and silyl ketene acetals as nucleophiles. This general methodology afforded 1,2-dihydrophthalazines in moderate-to-good yields and high enantioselectivities in most cases. Subsequent derivatizations provide direct access to key building blocks for the synthesis of dihydro- and tetrahydrophthalazines, phthalazones, and piperazic acid homologues.

## Data Availability

The data underlying this study are available in the published article and its Supporting Information.

## Supporting Information Available

Experimental details, optimizations, spectroscopic and analytical data for new compounds, HPLC traces, crystallographic data of (*S*,*S*)-**20** (PDF)
