# Supplementary material for: Asymmetric Dearomatization of Phthalazines by Anion-Binding Catalysis
Source: Org Lett. 2023 Dec 1;25(49):8797–802. doi: 10.1021/acs.orglett.3c03325 (PMC10729020; doi:10.1021/acs.orglett.3c03325)
Supplement: Supplementary file 1 — ol3c03325_si_001.pdf [file ol3c03325_si_001.pdf]

Supporting Information

Asymmetric Dearomatization of Phthalazines by Anion-Binding Catalysis

Marta Velázquez,[a] Rosario Fernández,[a,*] José M. Lassaletta[b,*] and David Monge[a,*]

[a]Departamento de Química Orgánica, Facultad de Química, Universidad de Sevilla and Centro de Innovación en Química Avanzada (ORFEO-CINQA), C/ Prof. García González, 1, 41012 Sevilla, Spain. E-mail: dmonge@us.es, ffernan@us.es

[b]Instituto de Investigaciones Químicas (CSIC-US) and Centro de Innovación en Química Avanzada (ORFEO-CINQA), Avda. Américo Vespucio, 49, 41092 Sevilla, Spain. E-mail: jmlassa@iiq.csic.es

CONTENTS

1. General information…………………………………………………………………….....S2
2. Synthesis of 1-methoxyphthalazine (1j)………………………………..…………………...S3
3. Synthesis of 1-(benzyloxy)phthalazine (1k)……………………..………..…………...............S3
4. General procedure for the Suzuki-Miyaura coupling between 1h and boronic acid derivatives S2-4……………………………………………………………..…………………..….....S4
5. Synthesis of 1-(1*H*-pyrrol-1-yl)phthalazine (1o)………………………….....…………..….........S5
6. Synthesis of organocatalyst VII………………………………..……….................................S6
7. Preliminary screening of chiral organocatalysts………………...………………..……..……....S7
8. Optimization of other reaction parameters…………………....……………………..…...…...S8
9. Modifications in the pyridazine dearomatization reaction conditions………....……………………S10
10. Kinetic study of the model reaction…………………………………………..………................S11
11. Screening of acylating reagents…………………………………..…………............................S12
12. General procedure for the enantioselective dearomatization of phthalazine derivatives 1a-q………………………………..………...S17
13. General procedure for the enantioselective dearomatization of pyridazine (13a)...........................S33
14. Derivatizations…………………………………..………...S37
14.1. Synthesis of isopropyl (*S*)-2-(2-benzoyl-1,2,3,4-tetrahydrophthalazin-1-yl)acetate [(*S*)-16]...S37
14.2. Synthesis of isopropyl (*S*)-2-(2-benzoyl-4-oxo-1,2,3,4-tetrahydrophthalazin-1-yl)acetate [(*S*)-17]...S38
14.3. Synthesis of isopropyl (*S*)-2-{2-benzoyl-4-[(4-bromophenyl)ethynyl]-1,2-dihydrophthalazin-1-yl}acetate [(*S*)-18]..S39
14.4. Synthesis of 2-oxo-2-phenylethyl (*S*)-2-(1,2-dihydrophthalazin-1-yl)acetate [(*S*)-19]...S40
14.5. Synthesis of ethyl {2-[(*S*)-2-benzoyl-4-phenyl-1,2-dihydrophthalazin-1-yl]acetyl}-L-phenylalaninate [(*S*)-20]…..S41
15. NMR spectra of new compounds..S43
16. References...S91

1. General information

^{1}H NMR spectra were recorded at 300 MHz or 500 MHz (internal reference; $CDCl_3$ = 7.26; Acetone-d^6 = 2.05); ^{13}C NMR spectra were recorded at 75.5 MHz or 126 MHz (internal reference; $CDCl_3$ = 77.16; Acetone-d^6 = 29.84). ^{19}F NMR spectra were recorded at 471 MHz. Multiplicities were given as: s (singlet), br s (broad singlet), d (doublet), t (triplet), q (quartet) hept (heptet), dd (doublet of doublets), dq (doublet of quartets), ddd (doublet of doublet of doublets) and m (multiplet). Column chromatography was performed on silica gel (Merck Kieselgel 40-60). Analytical TLC was performed on aluminum backed plates (1.5 × 5 cm) pre-coated (0.25 mm) with silica gel (Merck, Silica Gel 60 F_{254}). Semipreparative TLC was performed on glass backed plates (5 x 10 cm) pre-coated (0.25 mm) with silica gel (Merck, Silica Gel 60 F_{254}). Compounds were visualized by exposure to UV light or/and by dipping the plates in solutions of ninhydrin, vainilline or phosphomolibdic acid stains following by heating. The melting point of crystalline solid (*S*)-20 was recorded in a metal block and is uncorrected. Optical rotations were measured on a JASCO P-2000 polarimeter. The enantiomeric excess (ee) of the products was determined by chiral stationary phase HPLC (Daicel Chiralpack IA, IB, IC, ID). High resolution mass spectrometry (HRMS) was performed using a Thermo Fisher Orbitrap Elite with an orbitrap mass analyzer. Unless otherwise noted, commercially available reagents were used without further purification. Solvents for catalytic reactions (MTBE, Et_2O, THF and toluene) were distilled and dried over Na at 760 Torr. Phthalazine derivatives **1b**,[1] **1c**,[1] **1d**,[1] **1e**,[2] **1f**,[3] **1g**,[3] **1h**,[4] **1i**,[5] **1l**,[3] **1p**[4] silyl ketene acetals **2a**,[6] **2c**,[6] **2d**,[7] **2e**,[6] **2g**[8] and organocatalysts **O2**,[9] **O3**,[10] **O4**,[11] **O5**,[12] **I**,[13] **II**,[14] **III**,[12] **IV**,[15] **V**,[16] **VI**[17] were synthesized according to literature procedures.

2. Synthesis of 1-methoxyphthalazine (1j)

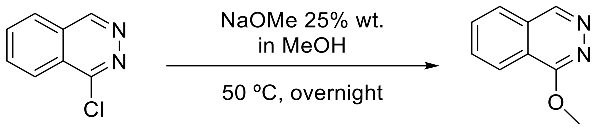

1h **1j**

1-Chlorophthalazine (**1h**) (388 mg, 2.3 mmol) was dissolved in a solution of NaOMe 25% wt. in MeOH (5.3 mL, 23 mmol) and the mixture was stirred at 50 °C in an oil bath overnight. Then, the solvent was removed under reduced pressure and the residue purified by flash chromatography (cyhex/Acetone 3/2) to afford **1j** as a yellow solid (198 mg, 54%). **^{1}H-NMR** (300 MHz, $CDCl_3$): δ 9.15 – 9.14 (m, 1H), 8.19 – 8.14 (m, 1H), 7.86 – 7.79 (m, 3H), 4.26 – 4.25 (m, 3H). **^{13}C-NMR** (75.5 MHz, $CDCl_3$): δ 160.7, 148.1, 132.3, 132.2, 128.8, 125.9, 123.0, 120.0, 55.0. **HRMS** (ESI) *m/z*: $[M+H]^+$ Calcd for $C_9H_9ON_2$ 161.0709; found 161.0706.

3. Synthesis of 1-(benzyloxy)phthalazine (1k)

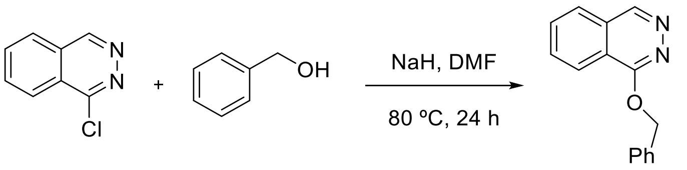

1h **S1** **1k**

In a flame-dried Schlenk flask, benzyl alcohol (**S1**) (0.3 mL, 3 mmol) was added dropwise to a mixture of NaH (107 mg, 4 mmol) in DMF (20 mL). The mixture was stirred 30 min at room temperature. Then, 1-chlorophthalazine (**1h**) (329 mg, 2 mmol) was added and the corresponding mixture was stirred at 80 °C in an oil bath for 24 h. After that time, the reaction was cooled to room temperature and EtOAc (15 mL) and H_2O (15 mL) were added. The organic layer was washed with NaCl (3 x 15 mL), dried over $MgSO_4$ and the solvent was removed under reduced pressure. The resulting residue was purified by flash chromatography (*n*-hexane/EtOAc 1/1) to afford **1k** as an orange oil (235 mg, 50%). **^{1}H-NMR** (300 MHz, $CDCl_3$): δ 9.16 (d, J = 0.8 Hz, 1H), 8.24 – 8.17 (m, 1H), 7.86 – 7.75 (m, 3H), 7.57 – 7.53 (m, 2H), 7.43 – 7.31 (m, 3H), 5.71 (s, 2H). **^{13}C-NMR** (75.5 MHz, $CDCl_3$): δ 160.2, 148.1, 136.7, 132.3, 132.1, 128.8, 128.6, 128.3, 128.2, 125.8, 122.9, 119.9, 69.1. **HRMS** (ESI) *m/z*: $[M+H]^+$ Calcd for $C_{15}H_{13}ON_2$ 237.1022; found 237.1017.

4. General procedure for the Suzuki-Miyaura coupling between 1h and boronic acid derivatives S2-4

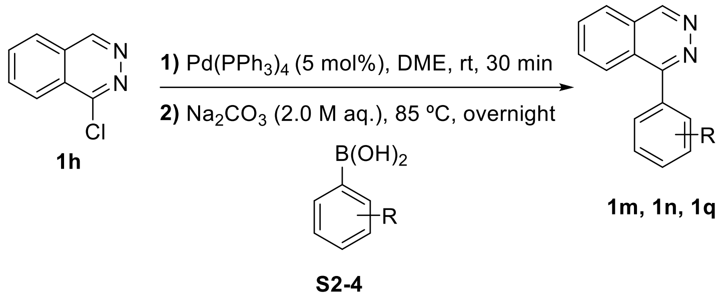

A flame-dried Schlenk flask was charged with $Pd(PPh_3)_4$ (89 mg, 0.075 mmol) and 1-chlorophthalazine (**1h**) (250 mg, 1.5 mmol). After three cycles of vacuum-argon, DME (3 mL) was added and the reaction mixture was stirred for 30 min at room temperature. Then, the corresponding boronic acid derivative **S2-4** (1.2 eq, 1.8 mmol) and Na_2CO_3 2.0 M (1.8 mL) were added and the reaction mixture was stirred at 85 °C in an oil bath overnight. After that time, the reaction was cooled to room temperature and H_2O (2.5 mL) was added. The aqueous layer was extracted with DCM (3 x 7.5 mL). The combined organic layers were washed with NaCl (1 x 7.5 mL), dried over $MgSO_4$ and the solvent was removed under reduced pressure. The resulting residue was purified by flash chromatography to afford **1m, 1n and 1q**.

1-(*o*-Tolyl)phthalazine (1m): Following the general procedure **4**, using *o*-tolylboronic acid (**S2**) (246 mg, 1.8 mmol), **1m** was obtained after purification by flash chromatography (cyhex/Acetone 3/1) as a brown oil (234 mg, 71%). **^{1}H-NMR** (300 MHz, $CDCl_3$): 9.52 – 9.51 (m, 1H), 7.99 – 7.97 (m, 1H), 7.88 – 7.83 (m, 1H), 7.79 – 7.73 (m, 1H), 7.59 – 7.56 (m, 1H), 7.40 – 7.28 (m, 4H), 2.05 (s, 3H). **^{13}C-NMR** (75.5 MHz, $CDCl_3$): δ 160.7, 150.6, 136.7, 135.5, 132.7, 132.4, 130.4, 129.8, 129.1, 126.6, 126.5, 126.1, 125.7, 19.8. **HRMS** (ESI) *m/z*: $[M+H]^+$ Calcd for $C_{15}H_{13}N_2$ 221.1073; found 221.1073.

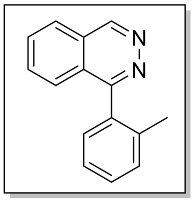

1-(Benzo[d][1,3]dioxol-5-yl)phthalazine (1n): Following the general procedure **4**, using 3,4-methylenedioxyphenylboronic acid (**S3**) (300 mg, 1.8 mmol), **1n** was obtained after purification by flash chromatography (cyhex/Acetone 2/1) as a yellow solid (125 mg, 33%). **^{1}H-NMR** (300 MHz, $CDCl_3$): δ 9.49 (s, 1H), 8.15 – 8.11 (m, 1H), 8.02 – 7.99 (m, 1H), 7.94 – 7.84 (m, 2H), 7.27 – 7.23 (m, 2H), 7.01 – 6.98 (m, 1H), 6.07 (s, 2H). **^{13}C-NMR** (75.5 MHz, $CDCl_3$): δ 159.5, 150.5, 148.9, 148.1, 132.7, 132.3, 130.1, 127.3, 126.8, 126.3, 126.5, 124.5, 110.6, 108.6, 101.6. **HRMS** (ESI) *m/z*: $[M+H]^+$ Calcd for $C_{15}H_{11}O_2N_2$ 251.0815; found 251.0817.

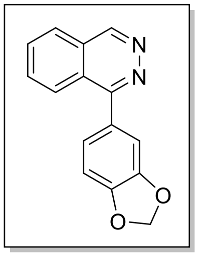

1-(Isoquinolin-4-yl)phthalazine (1q): Following the general procedure **4**, using isoquinolin-4-ylboronic acid (**S4**) (318 mg, 1.8 mmol), **1q** was obtained after purification by flash chromatography (cyhex/Acetone 1/1) as a brown solid (243 mg, 63%). **^{1}H-NMR** (300 MHz, $CDCl_3$): 9.67 – 9.66 (m, 1H), 9.44 (s, 1H), 8.70 (s, 1H), 8.13 – 8.08 (m, 2H), 7.98 – 7.92 (m, 1H), 7.82 – 7.76 (m, 1H), 7.69 – 7.58 (m, 3H), 7.54 – 7.50 (m, 1H). **^{13}C-NMR** (126 MHz, $CDCl_3$): δ 157.4, 154.0, 151.3, 144.3, 134.9, 133.1, 132.9, 131.3, 128.5, 128.1, 127.9, 127.3, 127.0, 126.8, 126.0, 124.8. **HRMS** (ESI) *m/z*: $[M+H]^+$ Calcd for $C_{17}H_{12}N_3$ 258.1026; found 258.1029.

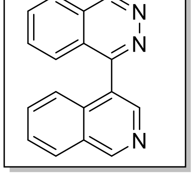

5. Synthesis of 1-(1*H*-pyrrol-1-yl)phthalazine (1o)

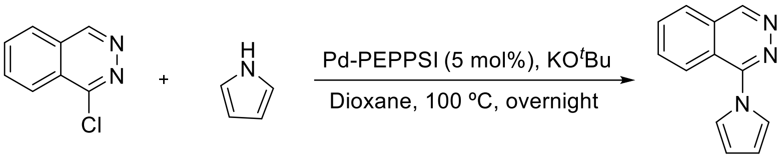

Pyrrole (**S5**) (85 µL, 1.2 mmol) was added to a suspension of 1-chlorophthalazine (**1h**) (165 mg, 1 mmol), Pd-PEPPSI (35 mg, 0.05 mmol) and KO^{t}Bu (178 mg, 1.5 mmol) in dioxane (4 mL). The mixture was stirred at 100 °C in an oil bath overnight. Then, the reaction was cooled to room temperature and H_2O (20 mL) was added. The aqueous layer was extracted with DCM (3 x 5 mL). The combined organic layers were washed with NaCl (1 x 5 mL), dried over $MgSO_4$ and the solvent was removed under reduced pressure. The resulting residue was purified by flash chromatography (cyhex/Acetone 4/1) to afford **1o** as a yellow solid (72 mg, 37%). **^{1}H-NMR** (300 MHz, $CDCl_3$): δ 9.45 – 9.45 (m, 1H), 8.29 – 8.23 (m, 1H), 8.08 – 8.01 (m, 1H), 7.99 – 7.91 (m, 2H), 7.40 – 7.39 (m, 2H), 6.49 – 6.48 (m, 2H). **^{13}C-NMR** (75.5 MHz, $CDCl_3$): δ 152.5, 150.5, 133.3, 132.9, 129.1, 126.9, 124.4, 122.2, 121.9, 111.4. **HRMS** (ESI) *m/z*: $[M+H]^+$ Calcd for $C_{12}H_{10}N_3$ 196.0869; found 196.0867.

6. Synthesis of organocatalyst VII

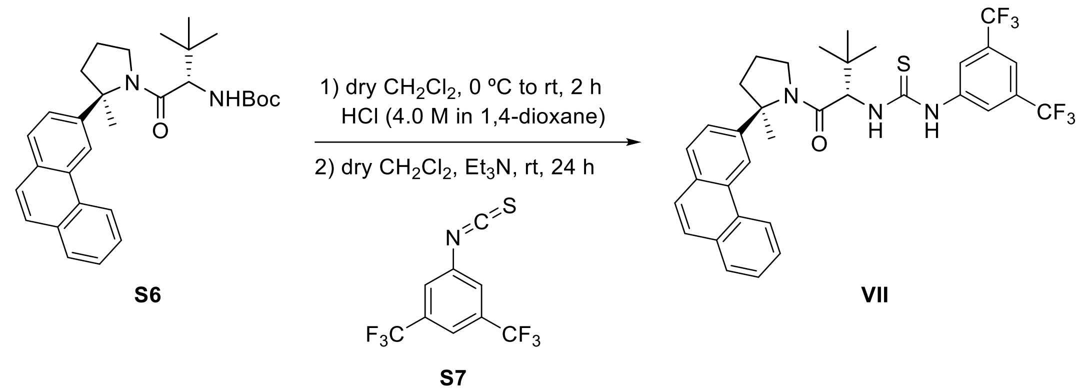

Step 1 (*Synthesis of S6´*): HCl (4.0 M in 1,4-dioxane, 2.5 mL, 10 mmol) was dropwise added to a solution of **S6**[18] (237 mg, 0.5 mmol) in dry CH_2Cl_2 (5 mL) under argon atmosphere at 0 °C. The reaction mixture was allowed to warm to room temperature and stirred for 2 hours. After this time, the solvent was removed under reduced pressure to afford the corresponding salt **S6´**as a yellow foam, which was used in the next step without further purification.

Step 2: Et_3N (209 µL, 1.5 mmol) was added to a solution of **S6´** (~0.5 mmol) in dry CH_2Cl_2 (5 mL) at 0 °C. After 15 min, 1-isothiocyanato-3,5-bis(trifluoromethyl)benzene (**S7**) (103 µL, 0.55 mmol) was added and the reaction mixture was allowed to warm to room temperature and stirred for 24 h. Then, the solvent was removed under reduced pressure and the residue purified by flash chromatography (*n*-hexane/EtOAc 6/1) to afford **VII** as a beige solid (189 mg, 59% in 2 steps). **$[\alpha]_D^{30}$** = +54.1 (*c* 1, $CHCl_3$). **^{1}H-NMR** (300 MHz, $CDCl_3$): δ 9.37 (br s, 1H), 8.55 – 8.52 (m, 1H), 8.19 – 8.18 (m, 1H), 7.80 – 7.77 (m, 1H), 7.59 – 7.31 (m, 10H), 5.66 (d, *J* = 9.6 Hz, 1H), 4.76 – 4.69 (m, 1H), 4.00 – 3.92 (m, 1H), 2.19 – 2.15 (m, 2H), 2.05 (s, 3H), 1.96 – 1.81 (m, 2H), 1.13 (s, 9H). **^{13}C-NMR** (126 MHz, $CDCl_3$): δ 180.8, 170.2, 142.6, 139.0, 132.2, 132.1 (q, *J* = 33.5 Hz), 132.12, 130.2 (d, *J* = 3.8 Hz), 129.5, 128.7, 128.6, 126.8, 126.5, 126.4, 126.2, 129.3, 123.7, 123.1, 122.6, 121.8, 118.7 – 118.5 (m), 118.4, 68.5, 63.6, 50.8, 44.7, 36.3, 27.0, 25.8, 22.5. **^{19}F NMR** (471 MHz, $CDCl_3$): δ −62.82 (s, 6F). **HRMS** (ESI) *m/z*: $[M+H]^+$ Calcd for $C_{34}H_{34}ON_3F_6S$ 646.2321; found 646.2317.

7. Preliminary screening of chiral organocatalysts

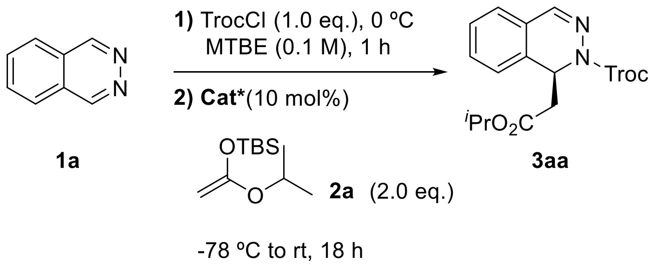

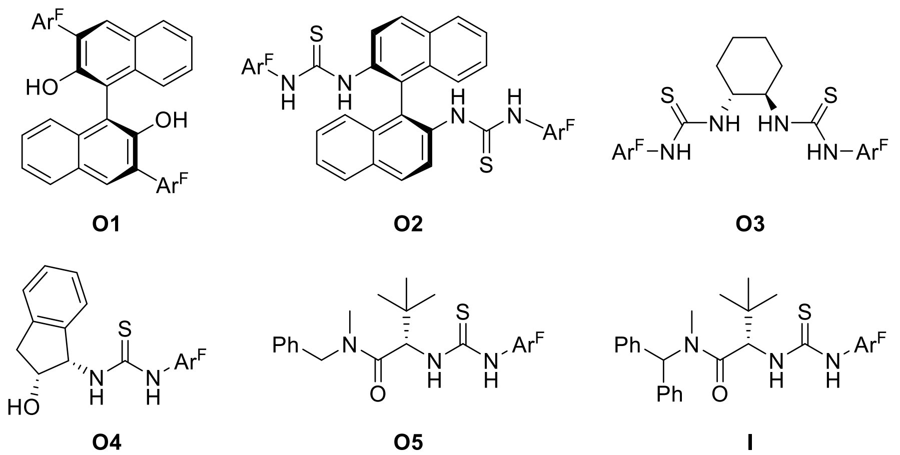

In a flame-dried Schlenk flask, 2,2,2-trichloroethyl chloroformate (14 µL, 0.1 mmol) was added to a solution of phthalazine (**1a**) (13 mg, 0.1 mmol) in freshly distilled anhydrous MTBE (1 mL, 0.1 M) at 0 ºC. The resulting suspension was stirred for 1 h at room temperature. Then, the corresponding organocatalyst (0.01 mmol, 10 mol%) was added and the reaction was cooled to −78 ºC (dry ice/acetone bath). Silyl ketene acetal (**2a**) (51 µL, 0.2 mmol) was added and the reaction mixture was stirred for 18 h and allowed to warm slowly to room temperature during that time. Then, the solvent was removed under reduced pressure. NMR yield was determined by ^{1}H-NMR analysis of the crude reaction mixture and enantiomeric ratios were determined by HPLC analysis.

Table S1

Entry	Cat*	yield (%)[a]	ee (%)[b]
1	**O1**	56	5
2	**O2**	49	5[c]
3	**O3**	49	8[c]
4	**O4**	47	6
5	**O5**	55	75
6	**I**	57	81

[a]Determined by ^{1}H-NMR using mesitylene as internal standard. [b]Determined by HPLC analysis after isolation of the product by semipreparative TLC (*n*-hexane/EtOAc 5/1). [c](*R*)-Enantiomer.

8. Optimization of other reaction parameters

TrocCl (2,2,2-trichloroethyl chloroformate)

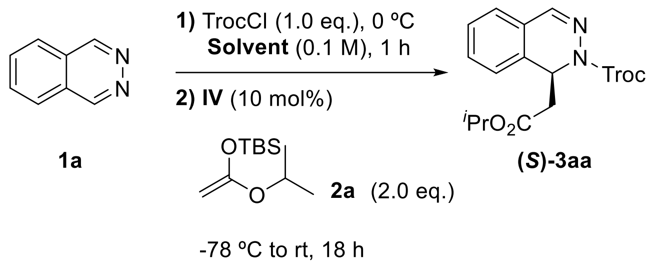

In a flame-dried Schlenk flask, 2,2,2-trichloroethyl chloroformate (14 μL, 0.1 mmol) was added to a solution of phthalazine (**1a**) (13 mg, 0.1 mmol) in the freshly distilled corresponding solvent (1 mL, 0.1 M) at 0 ºC. The resulting suspension was stirred for 1 h at room temperature. Then, catalyst **IV** (5 mg, 0.01 mmol, 10 mol%) was added and the reaction was cooled to −78 ºC (dry ice/acetone bath). Silyl ketene acetal **2a** (51 μL, 0.2 mmol) was added and the reaction mixture was stirred for 18 h and allowed to warm slowly to room temperature during that time. Then, the solvent was removed under reduced pressure. NMR yield was determined by ^{1}H-NMR analysis of the crude reaction mixture and enantiomeric ratios were determined by HPLC analysis.

Table S2

Entry	Solvent	yield (%)[a]	ee (%)[b]
**1**	**MTBE**	**75**	**89**
2	Et_2O	68	87
3	THF	62	83
4	Toluene	64	85
5	DCM	50	52
6[c]	C_6F_6	66	58
7[d]	MTBE	77	88
8[e]	MTBE	70	90
9[f]	MTBE	63	88
10[g]	MTBE	59	89

[a]Determined by ^{1}H-NMR using mesitylene as internal standard. [b]Determined by HPLC analysis after isolation of the product by semipreparative TLC (*n*-hexane/EtOAc 5/1). [c]The reaction was performed at 4 °C. [d][0.2 M]. [e][0.067 M]. [f]Cat IV loading: 5 mol%. [g] Cat IV loading: 3 mol%.

BzCl (benzoyl chloride)

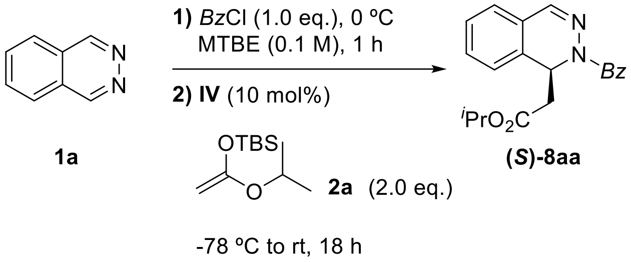

In a flame-dried Schlenk flask, benzoyl chloride (12 μL, 0.1 mmol) was added to a solution of phthalazine (**1a**) (13 mg, 0.1 mmol) in freshly distilled MTBE (1 mL, 0.1 M) at 0 ºC. The resulting suspension was stirred for 1 h at room temperature. Then, catalyst **IV** (5 mg, 0.01 mmol, 10 mol%) was added and the reaction was cooled to −78 ºC (dry ice/acetone bath). Silyl ketene acetal **2a** (51 μL, 0.2 mmol) was added and the reaction mixture was stirred for 18 h and allowed to warm slowly to room temperature during that time. Then, the solvent was removed under reduced pressure. NMR yield was determined by ^{1}H-NMR analysis of the crude reaction mixture and enantiomeric ratios were determined by HPLC analysis.

Table S3

Entry	Modification	yield (%)[a]	ee (%)[b]
**1**	**None**	**91**	**88**
2	0.067 M	80	87
3	5 mol % IV	92	88
4	3 mol% IV	89	88

[a]Determined by ^{1}H-NMR using mesitylene as internal standard. [b]Determined by HPLC analysis after isolation of the product by semipreparative TLC (*n*-hexane/EtOAc 5/1).

9. Modifications in the pyridazine dearomatization reaction conditions

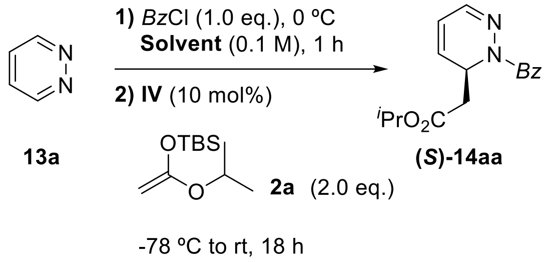

In a flame-dried Schlenk flask, benzoyl chloride (12 μL, 0.1 mmol) was added to a solution of pyridazine (**13a**) (7 μL, 0.1 mmol) in the freshly distilled corresponding solvent (1 mL, 0.1 M) at 0 ºC. The resulting suspension was stirred for 1 h at room temperature. Then, catalyst **IV** (5 mg, 0.01 mmol, 10 mol%) was added and the reaction was cooled to −78 ºC (dry ice/acetone bath). Silyl ketene acetal **2a** (51 μL, 0.2 mmol) was added and the reaction mixture was stirred for 18 h and allowed to warm slowly to room temperature during that time. Then, the solvent was removed under reduced pressure. NMR yield was determined by ^{1}H-NMR analysis of the crude reaction mixture and enantiomeric ratios were determined by HPLC analysis.

Table S4

Entry	Solvent	yield (%)[a]	ee (%)[b]
**1**	**MTBE**	**94**	**52**
2	Et_2O	99	30
3	Bu_2O	93	17
4	2-MeTHF	99	4
5	Anisole	53	28
6	Toluene	90	29
7	*n*-hexane	63	13
8[c]	MTBE	74	51
9[d]	MTBE	67	6
10[e]	MTBE	91	40

[a]Determined by ^{1}H-NMR using mesitylene as internal standard. [b]Determined by HPLC analysis after isolation of the product by semipreparative TLC (*n*-hexane/EtOAc 5/1). [c]The reaction was performed at -78 °C. [d]Cat I. [e]Cat VII.

10. Kinetic study of the model reaction

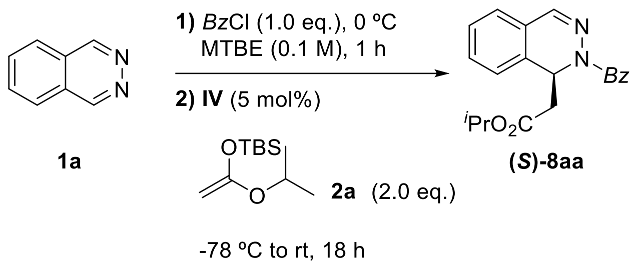

In a flame-dried Schlenk flask, benzoyl chloride (12 µL, 0.1 mmol) was added to a solution of phthalazine (**1a**) (13 mg, 0.1 mmol) in freshly distilled MTBE (1 mL, 0.1 M) at 0 ºC. The resulting suspension was stirred for 1 h at room temperature. Then, for enantioselective reactions, catalyst **IV** (3 mg, 0.005 mmol, 5 mol%) was added and the reaction was cooled to −78 ºC (dry ice/acetone bath). Silyl ketene acetal **2a** (51 µL, 0.2 mmol) was added and the reaction mixture was stirred for the indicate time and allowed to warm slowly to room temperature. The reaction was quenched by the addition of H_2O (0.5 mL) and was extracted with DCM (2 x 1 mL), dried over $MgSO_4$ and the solvent was removed under reduced pressure.

NMR yield was determined by ^{1}H-NMR analysis of the crude reaction mixture using mesitylene (0.1 mmol, 14 µL) as internal standard. Enantiomeric ratios were determined by HPLC analysis after isolation of the product by semipreparative TLC (*n*-hexane/EtOAc 5/1). **[**(Chiralpak IA, *n*-hexane/2-propanol 90:10, flow 1 mL/min) t_R 13.4 min (minor) and 19.9 min (major)].

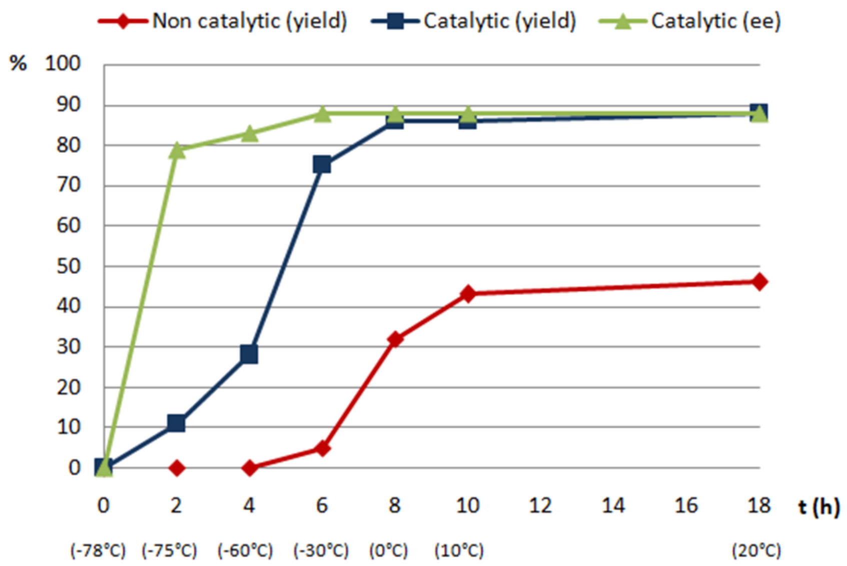

Figure 1. Non-catalyzed *vs* catalyzed reaction kinetics.

11. Screening of acylating reagents

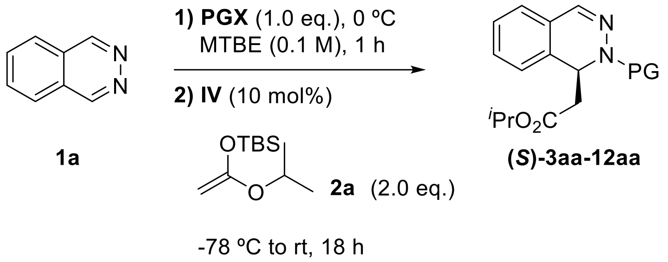

In a flame-dried Schlenk flask, the acylating reagent (1.0 eq, 0.2 mmol) was added to a solution of phthalazine (**1a**) (27 mg, 0.2 mmol) in freshly distilled anhydrous MTBE (2 mL, 0.1 M) at 0 ºC. The resulting suspension was stirred for 1 h at room temperature. Then, catalyst **IV** (11 mg, 0.02 mmol, 10 mol%) was added and the reaction was cooled to −78 ºC (dry ice/acetone bath). Silyl ketene acetal **2a** (102 µL, 0.4 mmol) was added and the reaction mixture was stirred for 18 h and allowed to warm slowly to room temperature during that time. Then, the solvent was removed under reduced pressure and the residue purified by flash chromatography to afford the corresponding products **(*S*)-3aa-12aa**. Enantiomeric ratios were determined by HPLC analysis.

Racemic samples were prepared without catalyst following the general procedure describe above.

2,2,2-Trichloroethyl (*S*)-1-(2-isopropoxy-2-oxoethyl)phthalazine-2(1H)-carboxylate [(*S*)-3aa]: Following the general procedure **11**, employing 2,2,2-trichloroethyl chloroformate (28 µL, 0.2 mmol) as acylating reagent, **(*S*)-3aa** was obtained after purification by flash chromatography (*n*-hexane/EtOAc 5/1) as a colorless oil (57 mg, 70%, 89% ee). **$[\alpha]_D^{23}$** = +304.0 (*c* 0.5, $CHCl_3$). Lit. **$[\alpha]_D^{20}$** = -145.0 [*c* 0.1, $CHCl_3$, 52% ee (*R*)]. **^{1}H-NMR** (300 MHz, $CDCl_3$): δ 7.80 (br s, 1H), 7.47 – 7.36 (m, 2H), 7.32 – 7.29 (m, 2H), 5.95 (t, *J* = 7.0 Hz, 1H), 5.06 – 4.83 (m, 3H), 2.68 – 2.67 (m, 2H), 1.18 (d, *J* = 6.3 Hz, 3H), 1.10 (d, *J* = 6.3 Hz, 3H). **^{13}C-NMR** (75.5 MHz, $CDCl_3$): δ 168.9, 152.4, 144.5, 132.5, 132.0, 129.0, 126.6, 126.3, 123.3, 95.1, 75.6, 68.5, 50.9, 39.6, 21.80, 21.77. The spectroscopic data match those reported in the literature.[19] **HPLC** (Chiralpak IA, *n*-hexane/2-propanol 90:10, flow 1 mL/min) t_R 12.1 min (major) and 15.4 min (minor).

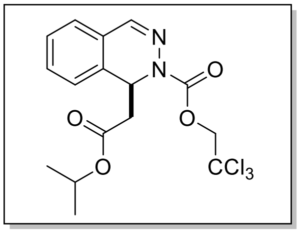

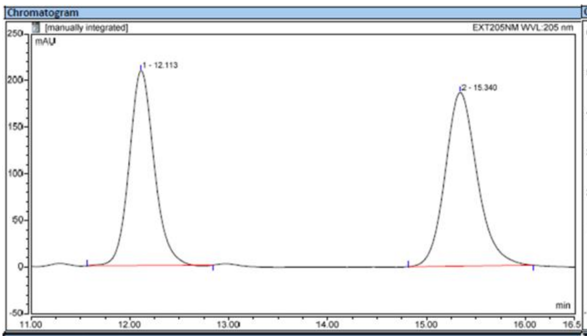

Integration Results

No.	Retention Time min	Area mAU*min	Relative Area %
1	12.113	61.060	46.59
2	15.340	69.984	53.41

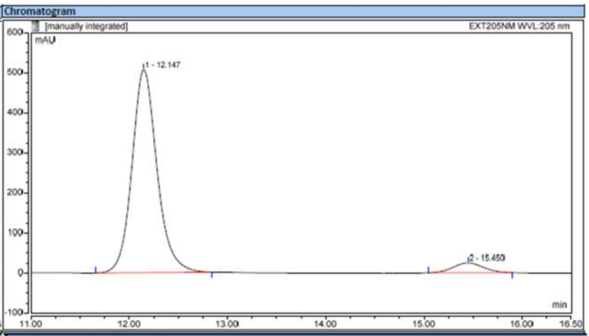

Integration Results

No.	Retention Time min	Area mAU*min	Relative Area %
1	12.147	149.385	94.50
2	15.450	8.691	5.50

2-Chloroethyl (*S*)-1-(2-isopropoxy-2-oxoethyl)phthalazine-2(1H)-carboxylate [(*S*)-4aa]: Following the general procedure **11**, employing 2-chloroethyl chloroformate (21 µL, 0.2 mmol) as acylating reagent, **(*S*)-4aa** was obtained after purification by flash chromatography (*n*-hexane/EtOAc 5/1) as a colorless oil (48 mg, 71%, 66% ee). **$[\alpha]_D^{23}$ =** +277.6 (*c* 1, $CHCl_3$). **^{1}H-NMR** (300 MHz, $CDCl_3$): δ 7.74 (br s, 1H), 7.46 – 7.35 (m, 2H), 7.31 – 7.27 (m, 2H), 5.92 (t, *J* = 6.9 Hz, 1H), 4.92 (hept, *J* = 6.3 Hz, 1H), 4.61 – 4.45 (m, 2H), 3.77 (t, *J* = 6.0 Hz, 2H), 2.69 – 2.57 (m, 2H), 1.19 (d, *J* = 6.3 Hz, 3H), 1.11 (d, *J* = 6.3 Hz, 3H). **^{13}C-NMR** (75.5 MHz, $CDCl_3$): δ 169.2, 153.4, 143.7, 132.5, 131.9, 128.9, 126.6, 126.1, 123.5, 68.4, 66.1, 50.6, 41.4, 39.7, 21.81, 21.78. **HRMS** (ESI) *m/z:* $[M+H]^+$ Calcd for $C_{16}H_{20}O_4N_2Cl$ 339.1106; found 339.1104. **HPLC** (Chiralpak IA, *n*-hexane/2-propanol 90:10, flow 1 mL/min) t_R 20.8 min (major) and 24.6 min (minor).

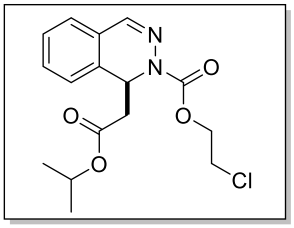

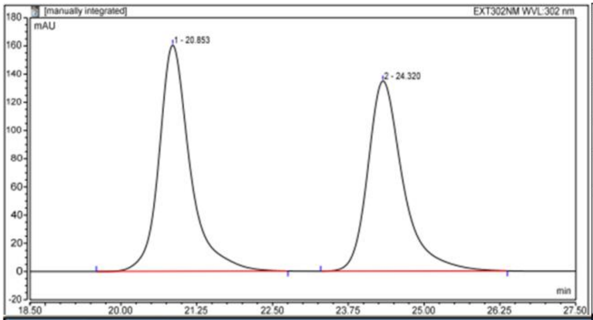

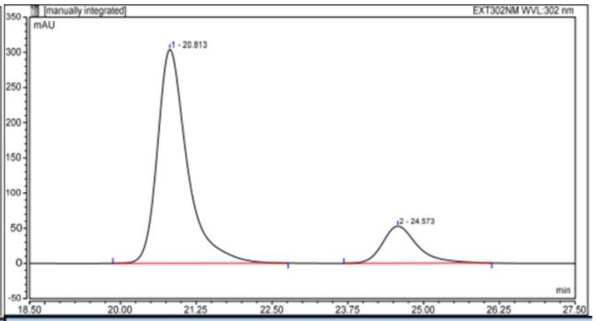

Integration Results

No.	Retention Time min	Area mAU*min	Relative Area %
1	20.853	95.337	51.20
2	24.320	90.871	48.80

Integration Results

No.	Retention Time min	Area mAU*min	Relative Area %
1	20.813	173.687	83.14
2	24.573	35.212	16.86

Benzyl (*S*)-1-(2-isopropoxy-2-oxoethyl)phthalazine-2(1H)-carboxylate [(*S*)-6aa]: Following the general procedure **11**, employing benzyl chloroformate (29 µL, 0.2 mmol) as acylating reagent, **(*S*)-6aa** was obtained after purification by flash chromatography (*n*-hexane/EtOAc 5/1) as a colorless oil (55 mg, 75%, 55% ee). **$[\alpha]_D^{23}$ =** +201.8 (*c* 1, $CHCl_3$). **^{1}H-NMR** (300 MHz, $CDCl_3$): δ 7.73 (br s, 1H), 7.46 – 7.25 (m, 9H), 5.95 (t, *J* = 7.0 Hz, 1H), 5.42 – 5.27 (m, 2H), 4.91 (hept, *J* = 6.3 Hz, 1H), 2.64 – 2.61 (m, 2H), 1.18 (d, *J* = 6.3 Hz, 3H), 1.10 (d, *J* = 6.3 Hz, 3H). **^{13}C-NMR** (75.5 MHz, $CDCl_3$): δ 169.2, 153.8, 143.3, 136.0, 132.6, 131.8, 128.8, 128.6, 128.42, 128.39, 126.6, 126.0, 123.6, 68.5, 68.4, 50.6, 39.7, 21.80, 21.77. **HRMS** (ESI) *m/z:* $[M+Na]^+$ Calcd for $C_{21}H_{22}O_4N_2Na$ 389.1472; found 389.1470. **HPLC** (Chiralpak IB, *n*-hexane/2-propanol 90:10, flow 1 mL/min) t_R 9.7 min (minor) and 10.8 min (major).

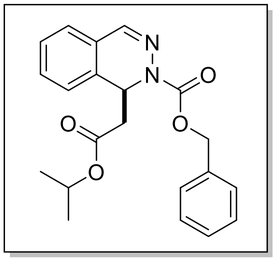

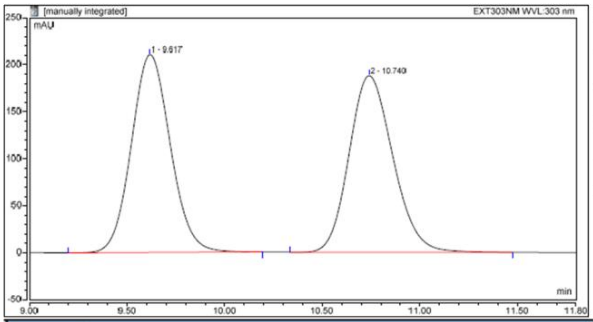

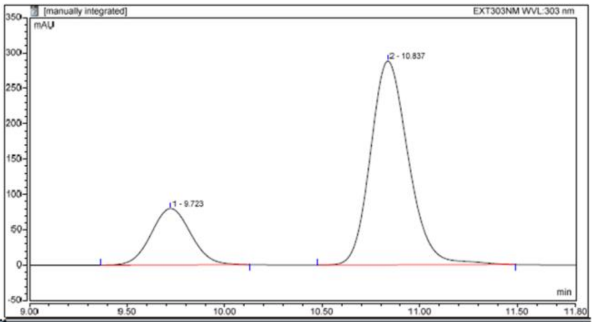

Integration Results

No.	Retention Time min	Area mAU*min	Relative Area %
1	9.617	48.703	49.89
2	10.740	48.920	50.11

Integration Results

No.	Retention Time min	Area mAU*min	Relative Area %
1	9.723	18.819	22.49
2	10.837	64.862	77.51

Isopropyl (*S*)-2-(2-acetyl-1,2-dihydrophthalazin-1-yl)acetate [(*S*)-7aa]: Following the general procedure

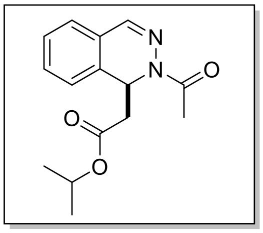

11, employing acetyl chloride (15 µL, 0.2 mmol) as acylating reagent, **(*S*)-7aa** was obtained after purification by flash chromatography (*n*-hexane/EtOAc 5/1) as a colorless oil (54 mg, 98%, 64% ee). **$[\alpha]_D^{23}$ =** +298.1 (*c* 1, $CHCl_3$). **^{1}H-NMR** (500 MHz, $CDCl_3$): δ 7.60 (s, 1H), 7.43 – 7.40 (m, 1H), 7.37 – 7.34 (m, 1H), 7.29 – 7.27 (m, 2H), 6.21 (t, *J* = 6.8 Hz, 1H), 4.90 (hept, *J* = 6.3 Hz, 1H), 2.55 (d, *J* = 6.8 Hz, 2H), 2.34 (s, 3H), 1.17 (d, *J* = 6.3 Hz, 3H), 1.13 (d, *J* = 6.3 Hz, 3H). **^{13}C-NMR** (126 MHz, $CDCl_3$): δ 172.3, 169.3, 142.0, 132.6, 131.8, 128.7, 126.8, 125.9, 123.9, 68.4, 47.9, 40.0, 21.81, 21.79, 21.4. **HRMS** (ESI) *m/z*: $[M+H]^+$ Calcd for $C_{15}H_{19}O_3N_2$ 275.1392; found 275.1388. **HPLC** (Chiralpak IA, *n*-hexane/2-propanol 90:10, flow 1 mL/min) t_R 7.6 min (minor) and 8.7 min (major).

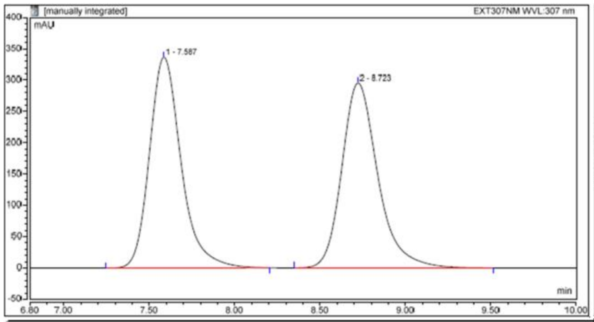

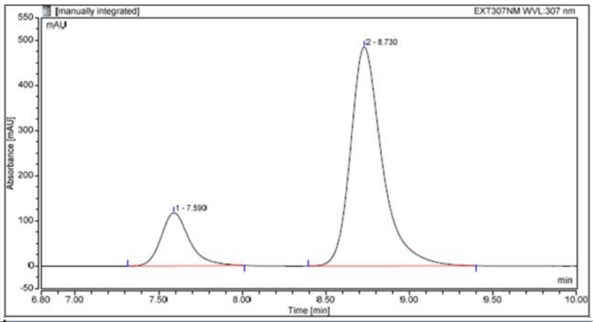

Integration Results

No.	Retention Time min	Area mAU*min	Relative Area %
1	7.587	72.126	49.97
2	8.723	72.223	50.03

Integration Results

No.	Retention Time min	Area mAU*min	Relative Area %
1	7.590	22.995	17.82
2	8.730	106.056	82.18

Isopropyl (*S*)-2-[2-(2-chlorobenzoyl)-1,2-dihydrophthalazin-1-yl]acetate [(*S*)-9aa]: Following the general

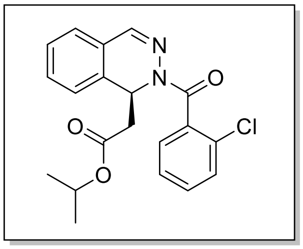

procedure **11**, employing *o*-chlorobenzoyl chloride (27 µL, 0.2 mmol) as acylating reagent, **(*S*)-9aa** was obtained after purification by flash chromatography (*n*-hexane/EtOAc 5/1) as a colorless oil (44 mg, 59%, 80% ee). **$[\alpha]_D^{23}$ =** +401.3 (*c* 1, $CHCl_3$). **^{1}H-NMR** (500 MHz, $CDCl_3$): δ 7.56 (s, 1H), 7.48 – 7.45 (m, 1H), 7.40 - 7.26 (m, 7H), 6.38 – 6.35 (m, 1H), 4.96 (hept, *J* = 6.3 Hz, 1H), 2.81 (dd, *J* = 14.6, 5.2 Hz, 1H), 2.73 (dd, *J* = 14.6, 8.5 Hz, 1H), 1.21 (d, *J* = 6.3 Hz, 3H), 1.15 (d, *J* = 6.3 Hz, 3H). **^{13}C-NMR** (126 MHz, $CDCl_3$): δ 169.2, 169.0, 143.5, 136.2, 132.5, 132.1, 131.1, 130.3, 129.4, 128.9, 128.5, 127.1, 126.7, 126.3, 123.7, 68.6, 48.6, 40.0, 21.92, 21.86. **HRMS** (ESI) *m/z*: $[M+Na]^+$ Calcd for

$C_{20}H_{19}O_3N_2ClNa$ 393.0976; found 393.0974. **HPLC** (Chiralpak IA, *n*-hexane/2-propanol 90:10, flow 1 mL/min) t_R 12.7 min (minor) and 18.7 min (major).

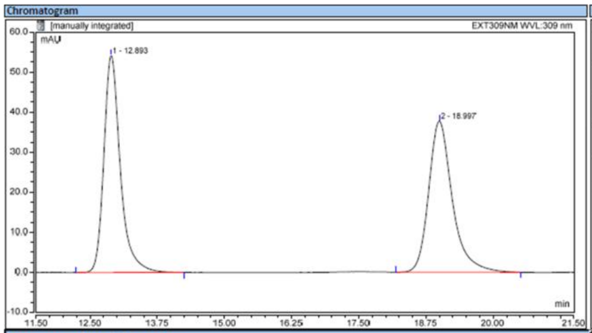

Integration Results

No.	Retention Time min	Area mAU*min	Relative Area %
1	12.893	19.212	49.91
2	18.997	19.279	50.09

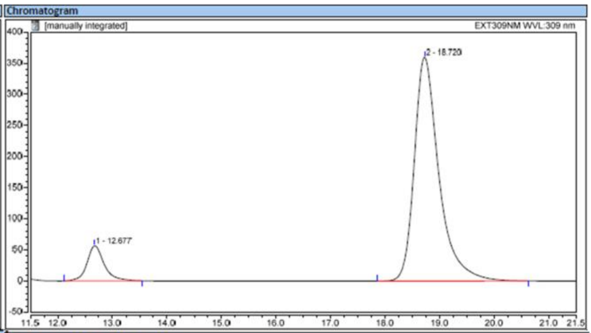

Integration Results

No.	Retention Time min	Area mAU*min	Relative Area %
1	12.677	20.516	9.85
2	18.720	187.691	90.15

Isopropyl (*S*)-2-[2-(4-chlorobenzoyl)-1,2-dihydrophthalazin-1-yl]acetate [(*S*)-10aa]: Following the general procedure **11**, employing *p*-chlorobenzoyl chloride (26 µL, 0.2 mmol) as acylating reagent, **(*S*)-10aa** was obtained after purification by flash chromatography (toluene/EtOAc 20/1) as a colorless oil (62 mg, 83%, 86% ee). $[\alpha]_D^{23}$ = +478.6 (*c* 1, $CHCl_3$). **^{1}H-NMR** (500 MHz, $CDCl_3$): δ 7.65 – 7.63 (m, 3H), 7.50 – 7.46 (m, 1H), 7.42 – 7.36 (m, 4H), 7.32 (d, *J* = 7.2 Hz, 1H), 6.31 – 6.28 (m, 1H), 4.95 (hept, *J* = 6.3 Hz, 1H), 2.76 (dd, *J* = 14.4, 5.4 Hz, 1H), 2.67 (dd, *J* = 14.4, 8.4 Hz, 1H), 1.20 (d, *J* = 6.3 Hz, 3H), 1.14 (d, *J* = 6.3 Hz, 3H). **^{13}C-NMR** (126 MHz, $CDCl_3$): δ 169.3, 169.2, 143.1, 137.0, 132.8, 132.7, 132.1, 131.4, 128.9, 128.1, 127.0, 126.0, 123.8, 68.5, 49.0, 39.5, 21.9, 21.8. **HRMS** (ESI) *m/z:* $[M+Na]^+$ Calcd for $C_{20}H_{19}O_3N_2ClNa$ 393.0976; found 393.0974. **HPLC** (Chiralpak IA, *n*-hexane/2-propanol 80:20, flow 1 mL/min) t_R 9.8 min (minor) and 14.9 min (major).

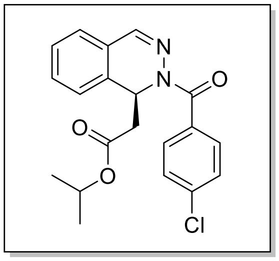

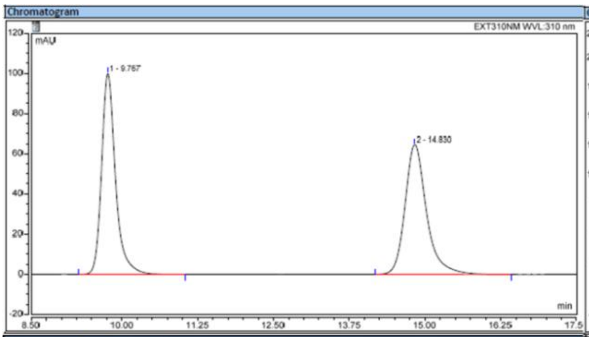

Integration Results

No.	Retention Time min	Area mAU*min	Relative Area %
1	9.767	26.545	50.00
2	14.830	26.549	50.00

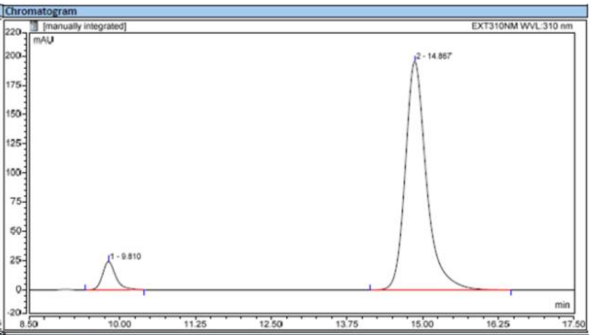

Integration Results

No.	Retention Time min	Area mAU*min	Relative Area %
1	9.810	6.129	7.15
2	14.867	79.603	92.85

Isopropyl (*S*)-2-[2-(4-nitrobenzoyl)-1,2-dihydrophthalazin-1-yl]acetate [(*S*)-11aa]: Following the general procedure **11**, employing *p*-nitrobenzoyl chloride (38 mg, 0.2 mmol) as acylating reagent, **(*S*)-11aa** was obtained after purification by flash chromatography (*n*-hexane/EtOAc 5/1) as a yellow oil (59 mg, 77%, 80% ee). **$[\alpha]_D^{23}$ =** +429.8 (*c* 1, $CHCl_3$). **^{1}H NMR** (500 MHz, $CDCl_3$): δ 8.27 – 8.24 (m, 2H), 7.82 – 7.80 (m, 2H), 7.63 (s, 1H), 7.53 – 7.50 (m, 1H), 7.45 – 7.39 (m, 2H), 7.33 (d, *J* = 7.2 Hz, 1H), 6.33 – 6.30 (m, 1H), 4.96 (hept, *J* = 6.3 Hz, 1H), 2.75 (dd, *J* = 14.3, 6.0 Hz, 1H), 2.69 (dd, *J* = 14.3, 7.7 Hz, 1H), 1.20 (d, *J* = 6.3 Hz, 3H), 1.17 (d, *J* = 6.3 Hz, 3H). **^{13}C-NMR** (126 MHz, $CDCl_3$): δ 169.1, 168.7, 148.8, 143.6, 140.9, 132.6, 132.4, 130.5, 129.1, 126.9, 126.4, 123.6, 123.0, 68.7, 49.0, 39.8, 21.9, 21.8. **HRMS** (ESI) *m/z*: $[M+Na]^+$ Calcd for $C_{20}H_{19}O_5N_3Na$ 404.1217; found 404.1214. **HPLC** (Chiralpak IA, *n*-hexane/2-propanol 80:20, flow 1 mL/min) t_R 14.8 min (minor) and 25.4 min (major).

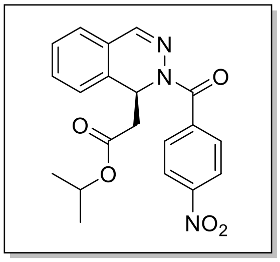

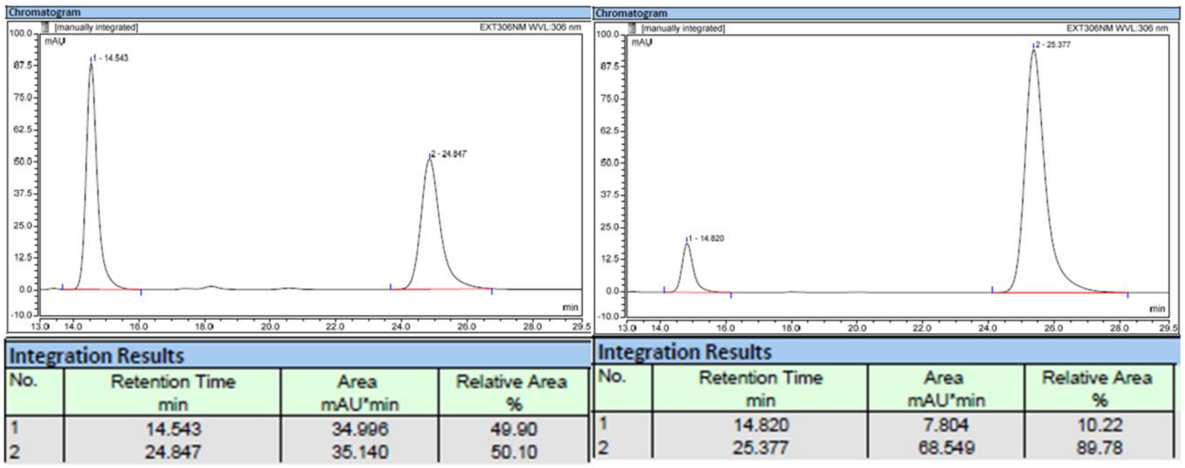

Integration Results

No.	Retention Time min	Area mAU*min	Relative Area %
1	14.543	34.996	49.90
2	24.847	35.140	50.10

Integration Results

No.	Retention Time min	Area mAU*min	Relative Area %
1	14.820	7.804	10.22
2	25.377	68.549	89.78

Isopropyl (*S*)-2-[2-(4-methylbenzoyl)-1,2-dihydrophthalazin-1-yl]acetate [(*S*)-12aa]: Following the general procedure **11**, employing *p*-toluoyl chloride (27 μL, 0.2 mmol) as acylating reagent, **(*S*)-12aa** was obtained after purification by flash chromatography (toluene/EtOAc 20/1) as a colorless oil (69 mg, 99%, 70% ee). **$[\alpha]_D^{23}$ =** +381.9 (*c* 1, $CHCl_3$). **^{1}H-NMR** (300 MHz, $CDCl_3$): δ 7.66 (s, 1H), 7.61 – 7.58 (m, 2H), 7.50 – 7.37 (m, 3H), 7.32 – 7.29 (m, 1H), 7.21 (d, *J* = 7.9 Hz, 2H), 6.33 – 6.28 (m, 1H), 4.95 (hept, *J* = 6.2 Hz, 1H), 2.79 (dd, *J* = 14.4, 5.2 Hz, 1H), 2.66 (dd, *J* = 14.4, 8.7 Hz, 1H), 2.39 (s, 3H), 1.21 (d, *J* = 6.3 Hz, 3H), 1.14 (d, *J* = 6.3 Hz, 3H). **^{13}C-NMR** (75.5 MHz, $CDCl_3$): δ 170.4, 169.3, 142.7, 141.2, 132.8, 131.8, 131.5, 130.0, 128.8, 128.5, 127.0, 125.8, 124.0, 68.4, 48.9, 39.3, 21.9, 21.8, 21.6. **HRMS** (ESI) *m/z*: $[M+H]^+$ Calcd for $C_{21}H_{23}O_3N_2$ 351.1703; found 351.1702. **HPLC** (Chiralpak IA, *n*-hexane/2-propanol 80:20, flow 1 mL/min) t_R 9.3 min (minor) and 15.8 min (major).

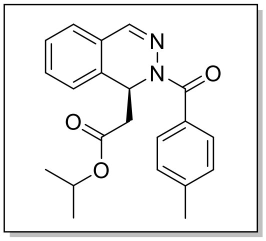

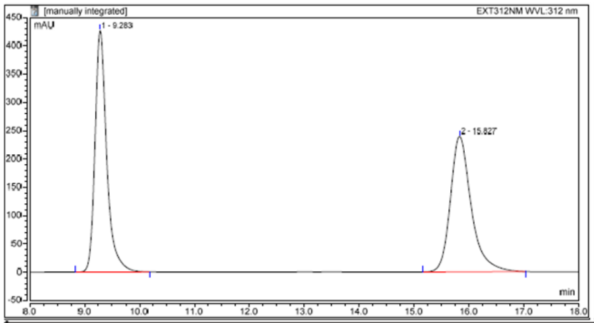

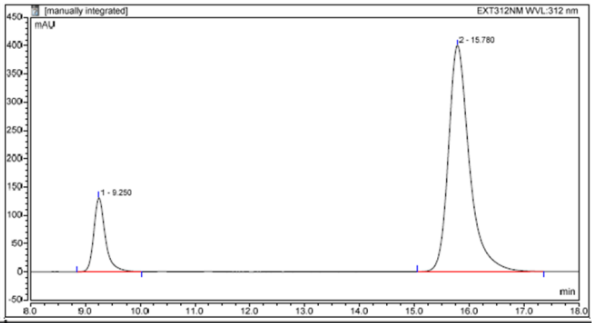

Integration Results			
No.	Retention Time min	Area mAU*min	Relative Area %
1	9.283	105.437	50.08
2	15.827	105.101	49.92

Integration Results			
No.	Retention Time min	Area mAU*min	Relative Area %
1	9.250	31.764	15.19
2	15.780	177.324	84.81

12. General procedure for the enantioselective dearomatization of phthalazine derivatives 1a-q

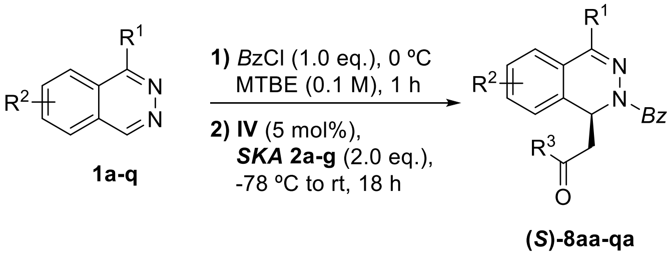

In a flame-dried Schlenk flask, benzoyl chloride (24 µL, 0.2 mmol) was added to a solution of the corresponding phthalazine derivative **1a-q** (0.2 mmol) in freshly distilled anhydrous MTBE (2 mL, 0.1 M) at 0 ºC. The resulting suspension was stirred for 1 h at room temperature. Then, catalyst **IV** (5 mg, 0.01 mmol, 5 mol%) was added and the reaction was cooled to −78 ºC (dry ice/acetone bath). Silyl ketene acetal **2a-g** (0.4 mmol) was added and the reaction mixture was stirred for 18 h and allowed to warm slowly to room temperature during that time. Then, the solvent was removed under reduced pressure and the residue purified by flash chromatography to afford the corresponding products **(*S*)-8aa-qa**. Enantio- and diastereomeric ratios were determined by HPLC analysis.

Racemic samples were prepared without catalyst following the general procedure describe above.

Isopropyl (*S*)-2-(2-benzoyl-1,2-dihydrophthalazin-1-yl)acetate [(*S*)-8aa]: Following the general procedure 12, starting from phthalazine (**1a**) (27 mg, 0.2 mmol) and *tert*-butyl[(1-isopropoxyvinyl)oxy]dimethylsilane (**2a**) (102 µL, 0.4 mmol), **(*S*)-8aa** was obtained after purification by flash chromatography (*n*-hexane/EtOAc 5/1) as a colorless oil (58 mg, 87%, 88% ee).

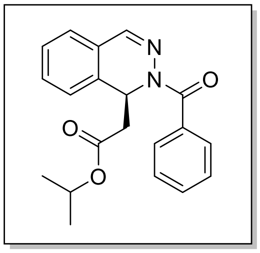

1 mmol scale: In a flame-dried Schlenk flask, benzoyl chloride (118 µL, 1 mmol) was added to a solution of phthalazine (**1a**) (133 mg, 1 mmol) in freshly distilled anhydrous MTBE (10 mL, 0.1 M) at 0 ºC. The resulting suspension was stirred for 1 h at room temperature. Then, catalyst **IV** (27 mg, 0.05 mmol, 5 mol%) was added and the reaction was cooled to −78 ºC (dry ice/acetone bath). Silyl ketene acetal **2a** (515 µL, 2 mmol) was added and the reaction mixture was stirred for 18 h and allowed to warm slowly to room temperature during that time. Then, the solvent was removed under reduced pressure and the residue purified by flash chromatography (*n*-hexane/EtOAc 5/1) to afford **(*S*)-8aa** as a colorless oil (292 mg, 87%, 86% ee). **$[\alpha]_D^{23}$ =** +504.1 (*c* 1, $CHCl_3$). **^{1}H-NMR** (300 MHz, $CDCl_3$): δ 7.69 – 7.65 (m, 3H), 7.51 – 7.30 (m, 7H), 6.34 – 6.29 (m, 1H), 4.96 (hept, *J* = 6.1 Hz, 1H), 2.79 (dd, *J* = 14.4, 5.3 Hz, 1H), 2.67 (dd, *J* = 14.4, 8.6 Hz, 1H), 1.21 (d, *J* = 6.3 Hz, 3H), 1.15 (d, *J* = 6.3 Hz, 3H). **^{13}C-NMR** (75.5 MHz, $CDCl_3$): δ 170.5, 169.3, 142.8, 134.6, 132.8, 131.9, 130.8, 129.8, 128.8, 127.8, 127.0, 126.0, 123.9, 68.5, 48.9, 39.4, 21.9, 21.8. **HRMS** (ESI) *m/z*: $[M+Na]^+$ Calcd for $C_{20}H_{20}O_3N_2Na$ 359.1366; found 359.1365. **HPLC** (Chiralpak IA, *n*-hexane/2-propanol 90:10, flow 1 mL/min) t_R 13.4 min (minor) and 19.9 min (major).

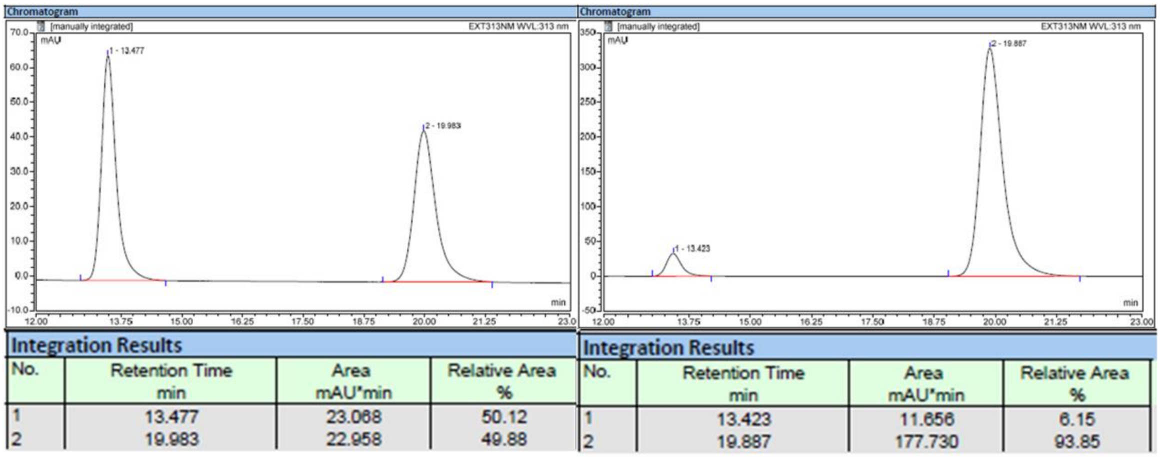

Integration Results

No.	Retention Time min	Area mAU*min	Relative Area %
1	13.477	23.068	50.12
2	19.983	22.958	49.88

Integration Results

No.	Retention Time min	Area mAU*min	Relative Area %
1	13.423	11.656	6.15
2	19.887	177.730	93.85

Methyl (*S*)-2-(2-benzoyl-1,2-dihydrophthalazin-1-yl)acetate [(*S*)-8ab]: Following the general procedure 12, starting from phthalazine (**1a**) (27 mg, 0.2 mmol) and *tert*-butyl[(1-methoxyvinyl)oxy]dimethylsilane (**2b**) (90 µL, 0.4 mmol), **(*S*)-8ab** was obtained after purification by flash chromatography (*n*-hexane/EtOAc 5/1) as a colorless oil (55 mg, 89%, 84% ee). **$[\alpha]_D^{23}$ =** +498.3 (*c* 1, $CHCl_3$). **^{1}H-NMR** (300 MHz, $CDCl_3$): δ 7.69 – 7.65 (m, 3H), 7.52 – 7.31 (m, 7H), 6.35 – 6.31 (m, 1H), 3.65 (s, 3H), 2.82 (dd, *J* = 14.3, 5.8 Hz, 1H), 2.68 (dd, *J* = 14.3, 8.1 Hz, 1H). **^{13}C-NMR** (75.5 MHz, $CDCl_3$): δ 170.5, 170.2, 142.8, 134.4, 132.7, 132.0, 130.8, 129.8, 128.9, 127.7, 126.7, 126.0, 123.8, 52.0, 48.9, 38.9. **HRMS** (ESI) *m/z*: $[M+Na]^+$ Calcd for $C_{18}H_{16}O_3N_2Na$ 331.1053; found 331.1050. **HPLC** (Chiralpak IB, *n*-hexane/2-propanol 90:10, flow 1 mL/min) t_R 11.3 min (minor) and 13.6 min (major).

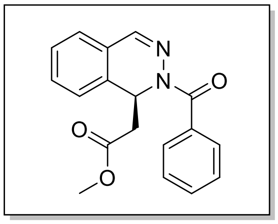

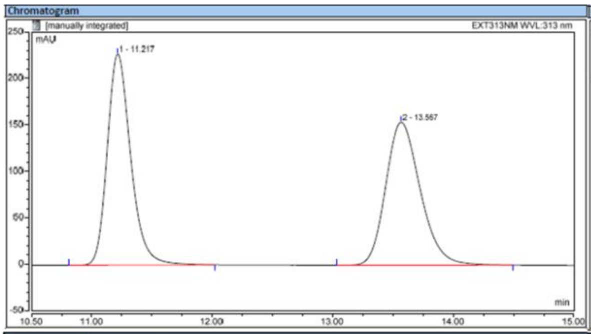

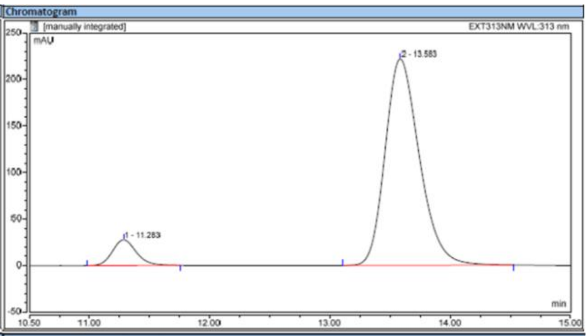

Integration Results

No.	Retention Time min	Area mAU*min	Relative Area %
1	11.217	51.204	50.04
2	13.567	51.114	49.96

Integration Results

No.	Retention Time min	Area mAU*min	Relative Area %
1	11.283	6.361	7.99
2	13.583	73.212	92.01

Ethyl (*S*)-2-(2-benzoyl-1,2-dihydrophthalazin-1-yl)acetate [(*S*)-8ac]: Following the general procedure **12**, starting from phthalazine (**1a**) (27 mg, 0.2 mmol) and *tert*-butyl[(1-ethoxyvinyl)oxy]dimethylsilane (**2c**) (83 µL, 0.4 mmol), **(*S*)-8ac** was obtained after purification by flash chromatography (toluene/EtOAc 15/1) as a colorless oil (52 mg, 80%, 90% ee). **$[\alpha]_D^{28}$ =** +505.4 (*c* 1, $CHCl_3$). **^{1}H-NMR** (300 MHz, $CDCl_3$): δ 7.68 – 7.66 (m, 3H), 7.51 – 7.30 (m, 7H), 6.35 – 6.31 (m, 1H), 4.10 (q, *J* = 7.1 Hz, 2H), 2.81 (dd, *J* = 14.4, 5.6 Hz, 1H), 2.69 (dd, *J* = 14.3, 8.3 Hz, 1H), 1.21 (t, *J* = 7.1 Hz, 3H). **^{13}C-NMR** (75.5 MHz, $CDCl_3$): δ 170.5, 169.8, 142.8, 134.5, 132.8, 132.0, 130.8, 129.8, 128.8, 127.8, 126.9, 126.0, 123.9, 61.0, 48.9, 38.2, 14.2. **HRMS** (ESI) *m/z*: $[M+Na]^+$ Calcd for $C_{19}H_{18}O_3N_2Na$ 345.1210; found 345.1208. **HPLC** (Chiralpak IA, *n*-hexane/2-propanol 90:10, flow 1 mL/min) t_R 14.5 min (minor) and 23.2 min (major).

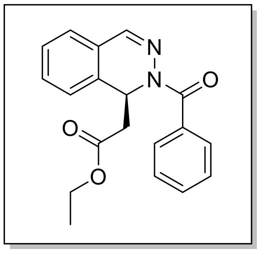

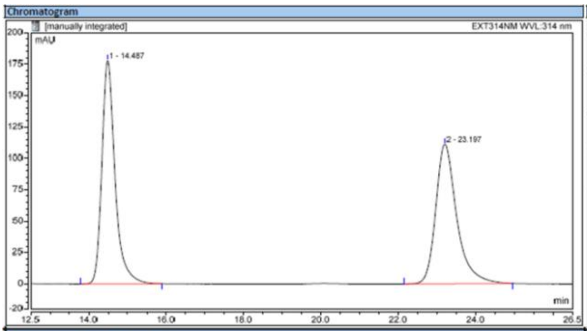

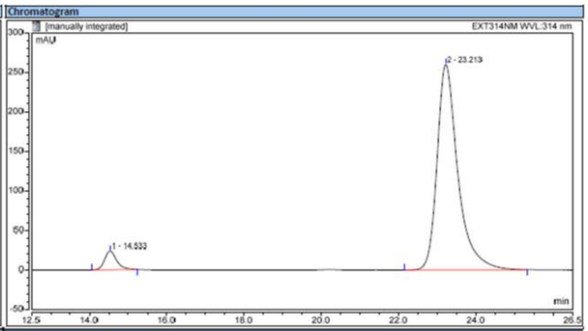

Integration Results

No.	Retention Time min	Area mAU*min	Relative Area %
1	14.487	70.046	50.11
2	23.197	69.735	49.89

Integration Results

No.	Retention Time min	Area mAU*min	Relative Area %
1	14.533	8.559	5.04
2	23.213	161.395	94.96

Benzyl (*S*)-2-(2-benzoyl-1,2-dihydrophthalazin-1-yl)acetate [(*S*)-8ad]: Following the general procedure **12**, starting from phthalazine (**1a**) (27 mg, 0.2 mmol) and {[1-(benzyloxy)vinyl]oxy}(*tert*-butyl)dimethylsilane (**2d**) (99 µL, 0.4 mmol), **(*S*)-8ad** was obtained after purification by flash chromatography (*n*-hexane/EtOAc 5/1) as a colorless oil (61 mg, 79%, 87% ee). **$[\alpha]_D^{23}$** = +515.6 (*c* 1, $CHCl_3$). **^{1}H-NMR** (300 MHz, $CDCl_3$): δ 7.67 – 7.63 (m, 3H), 7.50 – 7.28 (m, 12H), 6.36 – 6.32 (m, 1H), 5.13 – 5.03 (m, 2H), 2.87 (dd, *J* = 14.3, 5.5 Hz, 1H), 2.75 (dd, *J* = 14.3, 8.3 Hz, 1H). **^{13}C-NMR** (75.5 MHz, $CDCl_3$): δ 170.6, 169.6, 142.8, 135.6, 134.4, 132.6, 132.0, 130.9, 129.8, 128.9, 128.70, 128.66, 128.5, 127.8, 126.9, 126.0, 123.9, 66.9, 49.0, 39.1. **HRMS** (ESI) *m/z*: $[M+Na]^+$ Calcd for $C_{24}H_{20}O_3N_2Na$ 407.1366; found 407.1364. **HPLC** (Chiralpak IA, *n*-hexane/2-propanol 90:10, flow 1 mL/min) t_R 18.3 min (minor) and 33.7 min (major).

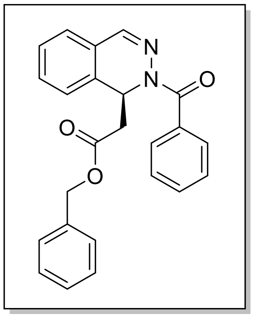

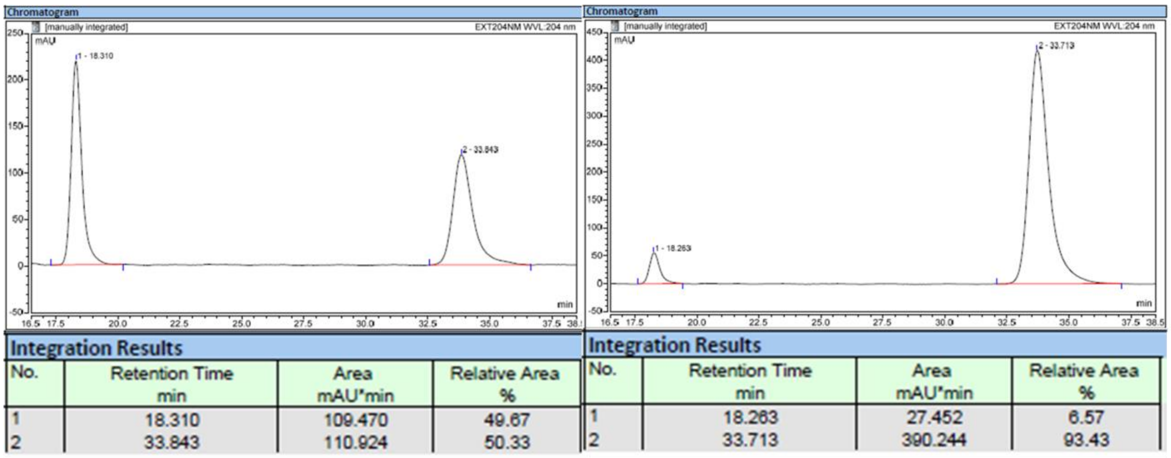

Integration Results

No.	Retention Time min	Area mAU*min	Relative Area %
1	18.310	109.470	49.67
2	33.843	110.924	50.33

Integration Results

No.	Retention Time min	Area mAU*min	Relative Area %
1	18.263	27.452	6.57
2	33.713	390.244	93.43

***tert*-Butyl (*S*)-2-(2-benzoyl-1,2-dihydrophthalazin-1-yl)acetate [(*S*)-8ae]:** Following the general procedure **12**, starting from phthalazine (**1a**) (27 mg, 0.2 mmol) and {[1-(*tert*-butoxy)vinyl]oxy}(*tert*-butyl)dimethylsilane (**2e**) (100 µL, 0.4 mmol), **(*S*)-8ae** was obtained after purification by flash chromatography (*n*-hexane/EtOAc 5/1) as a colorless oil (35 mg, 49%, 78% ee). **$[\alpha]_D^{23}$** = +418.2 (*c* 1, $CHCl_3$). **^{1}H-NMR** (300 MHz, $CDCl_3$): δ 7.68 – 7.65 (m, 3H), 7.50 – 7.30 (m, 7H), 6.32 – 6.27 (m, 1H), 2.75 (dd, *J* = 14.4, 5.2 Hz, 1H), 2.62 (dd, *J* = 14.4, 8.7 Hz, 1H), 1.39 (s, 9H). **^{13}C-NMR** (75.5 MHz, $CDCl_3$): δ 170.4, 169.0, 142.9, 134.6, 133.0, 131.8, 130.8, 129.8, 128.8, 127.8, 127.1, 125.9, 123.9, 81.3, 49.0, 40.3, 28.1. **HRMS** (ESI) *m/z*: $[M+Na]^+$ Calcd for $C_{21}H_{22}O_3N_2Na$ 373.1523; found 373.1520. **HPLC** (Chiralpak IA, *n*-hexane/2-propanol 90:10, flow 1 mL/min) t_R 10.2 min (minor) and 16.6 min (major).

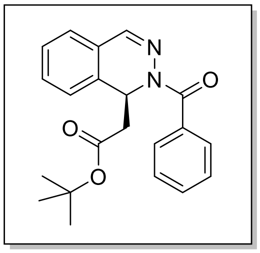

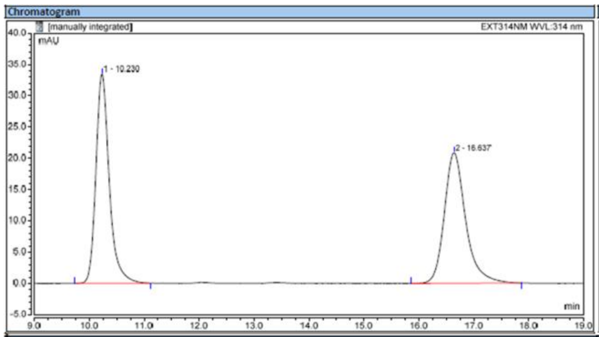

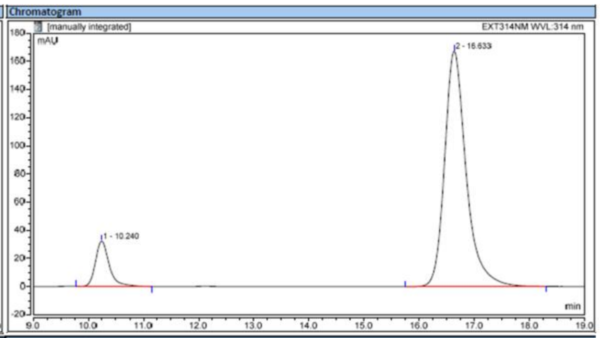

Integration Results

No.	Retention Time min	Area mAU*min	Relative Area %
1	10.230	9.400	50.10
2	16.637	9.361	49.90

Integration Results

No.	Retention Time min	Area mAU*min	Relative Area %
1	10.240	9.258	10.88
2	16.633	75.806	89.12

(*S*)-2-(2-Benzoyl-1,2-dihydrophthalazin-1-yl)-1-phenylethan-1-one [(*S*)-8af]: Following the general procedure **12**, starting from phthalazine (**1a**) (27 mg, 0.2 mmol) and trimethyl[(1-phenylvinyl)oxy]silane (**2f**) (85 µL, 0.4 mmol), **(*S*)-8af** was obtained after purification by flash chromatography (*n*-hexane/EtOAc 6/1) as a colorless oil (70 mg, 99%, 8% ee). **^{1}H-NMR** (300 MHz, $CDCl_3$): δ 7.99 – 7.96 (m, 2H), 7.71 (s, 1H), 7.67 – 7.64 (m, 2H), 7.58 – 7.53 (m, 1H), 7.50 – 7.30 (m, 9H), 6.53 – 6.48 (m, 1H), 3.50 (dd, J = 15.0, 4.4 Hz, 1H), 3.39 (dd, J = 15.0, 9.0 Hz, 1H). **^{13}C-NMR** (75.5 MHz, $CDCl_3$): δ 196.6, 170.6, 143.2, 136.8, 134.4, 133.4, 133.1, 132.0, 130.9, 129.9, 128.8, 128.7, 128.4, 127.8, 127.4, 126.0, 123.8, 49.2, 43.2. **HRMS** (ESI) *m/z*: $[M+Na]^+$ Calcd for $C_{23}H_{18}O_2N_2Na$ 377.1260; found 377.1255. **HPLC** (Chiralpak IA, *n*-hexane/2-propanol 85:15, flow 1 mL/min) t_R 11.7 min (minor) and 19.4 min (major).

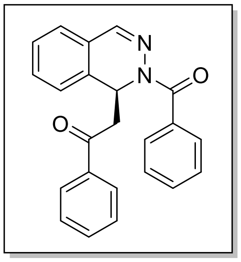

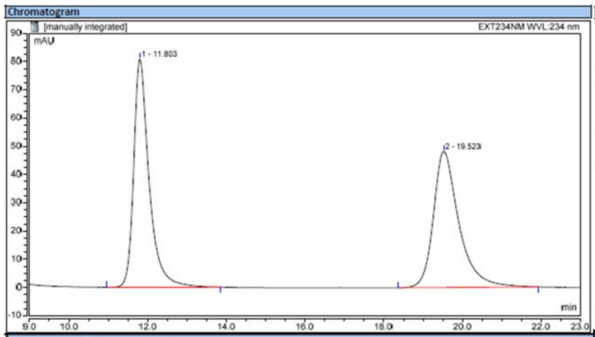

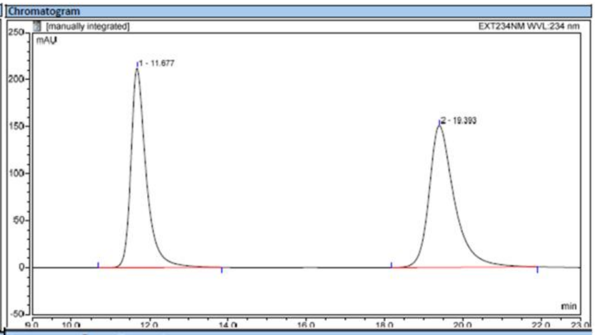

Integration Results

No.	Retention Time min	Area mAU*min	Relative Area %
1	11.803	36.702	50.34
2	19.523	36.201	49.66

Integration Results

No.	Retention Time min	Area mAU*min	Relative Area %
1	11.677	96.267	46.00
2	19.393	113.003	54.00

Methyl (*S*,*E*)-4-(2-benzoyl-1,2-dihydrophthalazin-1-yl)but-2-enoate [(*S*)-8ag]: Following the general procedure **12**, starting from phthalazine (**1a**) (27 mg, 0.2 mmol) and (*E*)-*tert*-butyl[(1-methoxypenta-2,4-dien-1-yl)oxy]dimethylsilane (**2g**) (94 µL, 0.4 mmol), **(*S*)-8ag** was obtained after purification by flash chromatography (*n*-hexane/EtOAc 5/1) as a yellow oil (58 mg, 87%, 55% ee). **$[\alpha]_D^{29}$ =** +348.6 (*c* 1, $CHCl_3$). **^{1}H-NMR** (300 MHz, $CDCl_3$): δ 7.66 – 7.62 (m, 3H), 7.51 – 7.37 (m, 5H), 7.32 – 7.30 (m, 1H), 7.21 – 7.19 (m, 1H), 6.90 – 6.79 (m, 1H), 6.06 – 6.01 (m, 1H), 5.71 (d, *J* = 15.5, 1H), 3.69 (s, 3H), 2.75 – 2.57 (m, 2H). **^{13}C-NMR** (75.5 MHz, $CDCl_3$): δ 170.5, 166.4, 142.9, 142.8, 134.6, 132.7, 131.9, 130.7, 129.7, 128.8, 127.8, 126.6, 126.0, 124.6, 124.0, 51.6, 51.3, 37.9. **HRMS** (ESI) *m/z*: $[M+Na]^+$ Calcd for $C_{20}H_{18}O_3N_2Na$ 357.1210; found 357.1205. **HPLC** (Chiralpak IA, *n*-hexane/2-propanol 85:15, flow 1 mL/min) t_R 11.5 min (minor) and 21.5 min (major).

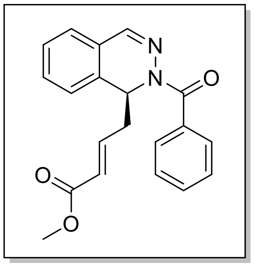

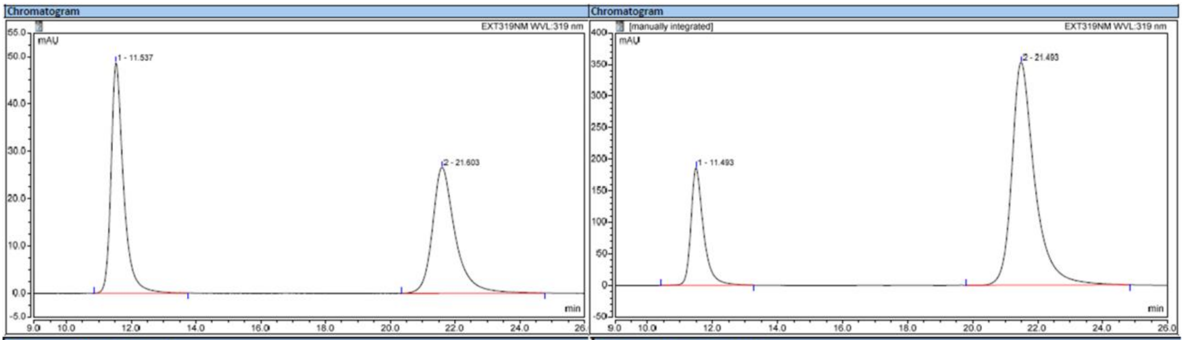

Integration Results

No.	Retention Time min	Area mAU*min	Relative Area %
1	11.537	21.791	50.17
2	21.603	21.639	49.83

Integration Results

No.	Retention Time min	Area mAU*min	Relative Area %
1	11.493	83.295	22.42
2	21.493	288.177	77.58

Isopropyl (*S*)-2-(2-benzoyl-1,2-dihydrobenzo[g]phthalazin-1-yl)acetate [(*S*)-8ba]: Following the general procedure **12**, starting from benzo[g]phthalazine (**1b**) (36 mg, 0.2 mmol) and *tert*-butyl[(1-isopropoxyvinyl)oxy]dimethylsilane (**2a**) (102 µL, 0.4 mmol), **(*S*)-8ba** was obtained after purification by flash chromatography (*n*-hexane/EtOAc 5/1) as a white solid (60 mg, 78%, 93% ee). **$[\alpha]_D^{28}$ =** +289.6 (*c* 1, $CHCl_3$). **^{1}H-NMR** (300 MHz, $CDCl_3$): δ 7.92 – 7.81 (m, 5H), 7.72 – 7.69 (m, 2H), 7.60 – 7.39 (m, 5H), 6.50 – 6.45 (m, 1H), 4.95 (hept, *J* = 6.3 Hz, 1H), 2.90 (dd, *J* = 14.5, 5.1 Hz, 1H), 2.74 (dd, *J* = 14.5, 9.0 Hz, 1H), 1.21 (d, *J* = 6.3 Hz, 3H), 1.08 (d, *J* = 6.3 Hz, 3H). **^{13}C-NMR** (75.5 MHz, $CDCl_3$): δ 170.4, 169.3, 142.8, 135.0, 134.6, 133.1, 130.9, 129.9, 129.7, 128.9, 128.2, 128.1, 127.8, 127.2, 126.3, 126.1, 121.7, 68.5, 49.3, 39.6, 21.9, 21.8. **HRMS** (ESI) *m/z*: $[M+Na]^+$ Calcd for $C_{24}H_{22}O_3N_2Na$ 409.1523; found 409.1519. **HPLC** (Chiralpak IB, *n*-hexane/2-propanol 90:10, flow 1 mL/min) t_R 9.3 min (minor) and 10.3 min (major).

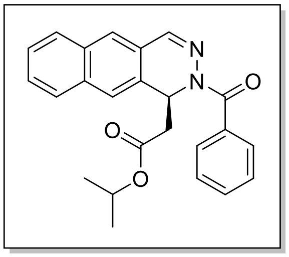

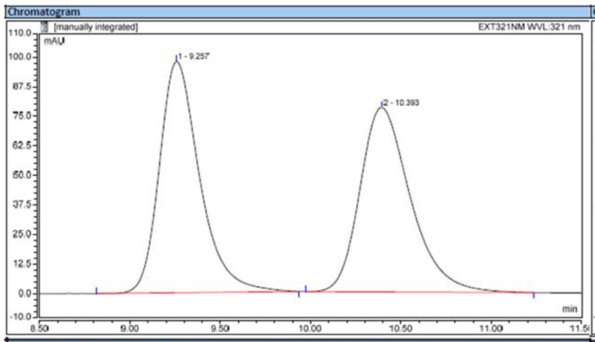

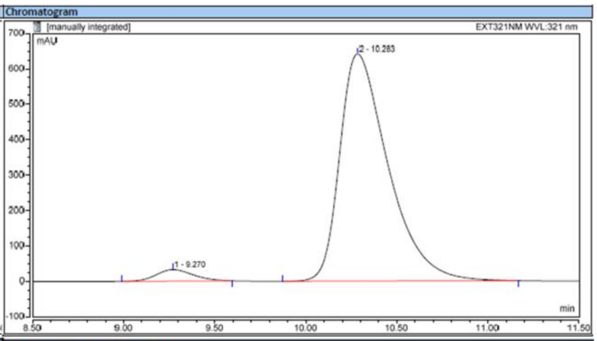

Integration Results			
No.	Retention Time min	Area mAU*min	Relative Area %
1	9.257	25.177	49.92
2	10.393	25.256	50.08

Integration Results			
No.	Retention Time min	Area mAU*min	Relative Area %
1	9.270	7.625	3.72
2	10.283	197.559	96.28

Isopropyl (*S*)-2-(2-benzoyl-6,7-dichloro-1,2-dihydrophthalazin-1-yl)acetate [(*S*)-8ca]: Following the general procedure **12**, starting from 6,7-dichlorophthalazine (**1c**) (39 mg, 0.2 mmol) and *tert*-butyl[(1-isopropoxyvinyl)oxy]dimethylsilane (**2a**) (102 µL, 0.4 mmol), **(*S*)-8ca** was obtained after purification by flash chromatography (*n*-hexane/EtOAc 7/1) as a colorless oil (44 mg, 54%, 46% ee). $[\alpha]_D^{29}$ **=** +205.0 (*c* 1, $CHCl_3$). **^{1}H-NMR** (300 MHz, $CDCl_3$): δ 7.67 – 7.64 (m, 2H), 7.58 (s, 1H), 7.53 (s, 1H), 7.49 – 7.38 (m, 4H), 6.28 – 6.23 (m, 1H), 4.99 (hept, *J* = 6.3 Hz, 1H), 2.79 (dd, *J* = 14.8, 5.0 Hz, 1H), 2.67 (dd, *J* = 14.8, 8.9 Hz, 1H), 1.23 (d, *J* = 6.3 Hz, 3H), 1.18 (d, *J* = 6.3 Hz, 3H). **^{13}C-NMR** (75.5 MHz, $CDCl_3$): δ 170.4, 169.0, 140.2, 135.9, 134.0, 133.2, 132.1, 131.2, 129.9, 129.3, 127.9, 127.4, 123.5, 68.9, 48.1, 39.0, 21.9. **HRMS** (ESI) *m/z*: $[M+Na]^+$ Calcd for $C_{20}H_{18}O_3N_2Cl_2Na$ 427.0587; found 427.0580. **HPLC** (Chiralpak IA, *n*-hexane/2-propanol 90:10, flow 1 mL/min) t_R 9.5 min (minor) and 15.7 min (major).

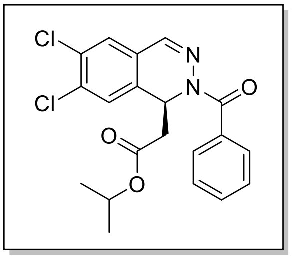

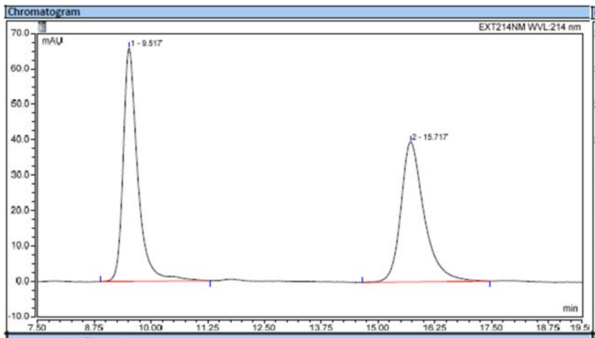

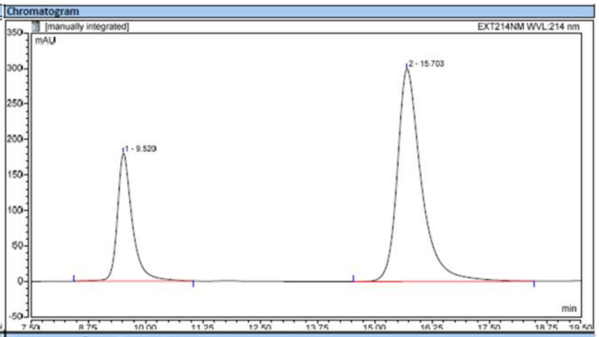

Integration Results			
No.	Retention Time min	Area mAU*min	Relative Area %
1	9.517	24.488	50.95
2	15.717	23.577	49.05

Integration Results			
No.	Retention Time min	Area mAU*min	Relative Area %
1	9.520	68.011	27.24
2	15.703	181.674	72.76

Isopropyl (*S*)-2-(2-benzoyl-6,7-dimethyl-1,2-dihydrophthalazin-1-yl)acetate [(*S*)-8da]: Following the general procedure **12**, starting from 6,7-dimethylphthalazine (**1d**) (32 mg, 0.2 mmol) and *tert*-butyl[(1-isopropoxyvinyl)oxy]dimethylsilane (**2a**) (102 μL, 0.4 mmol), **(*S*)-8da** was obtained after purification by flash chromatography (*n*-hexane/EtOAc 5/1) as a colorless oil (67 mg, 91%, 92% ee). **$[\alpha]_D^{23}$ =** +460.5 (*c* 1, $CHCl_3$). **^{1}H-NMR** (300 MHz, $CDCl_3$): δ 7.67 – 7.64 (m, 2H), 7.60 (s, 1H), 7.47 – 7.36 (m, 3H), 7.14 – 7.08 (m, 2H), 6.27 – 6.22 (m, 1H), 4.96 (hept, *J* = 6.3 Hz, 1H), 2.77 (dd, *J* = 14.2, 5.3 Hz, 1H), 2.63 (dd, *J* = 14.2, 8.5 Hz, 1H), 2.30 – 2.29 (m, 6H), 1.22 (d, *J* = 6.3 Hz, 3H), 1.16 (d, *J* = 6.3 Hz, 3H). **^{13}C-NMR** (75.5 MHz, $CDCl_3$): δ 170.5, 169.5, 143.2, 141.3, 137.3, 134.8, 130.7, 130.5, 129.8, 128.0, 127.8, 127.0, 121.8, 68.4, 48.8, 39.6, 21.93, 21.87, 20.2, 19.6. **HRMS** (ESI) *m/z*: $[M+Na]^+$ Calcd for $C_{22}H_{24}O_3N_2Na$ 387.1679; found 387.1677. **HPLC** (Chiralpak IC, *n*-hexane/2-propanol 90:10, flow 1 mL/min) t_R 30.2 min (major) and 34.6 min (minor).

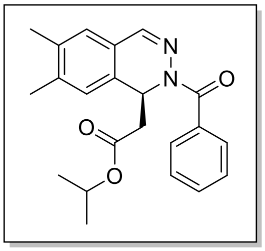

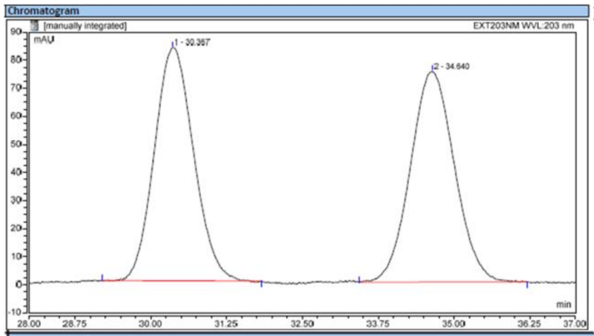

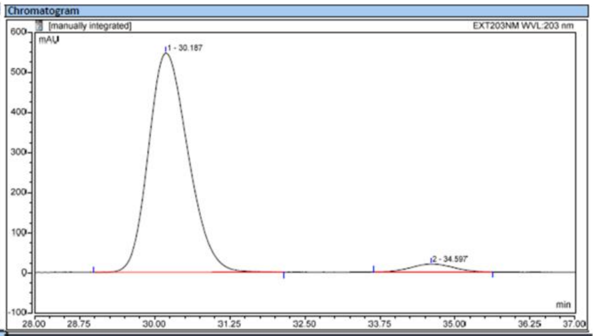

Integration Results

No.	Retention Time min	Area mAU*min	Relative Area %
1	30.367	62.239	49.65
2	34.640	63.107	50.35

Integration Results

No.	Retention Time min	Area mAU*min	Relative Area %
1	30.187	418.305	96.28
2	34.597	16.183	3.72

Isopropyl (*S*)-2-(6-benzoyl-5,6-dihydro-[1,3]dioxolo[4,5-g]phthalazin-5-yl)acetate [(*S*)-8ea]: Following the general procedure **12**, starting from [1,3]dioxolo[4,5-g]phthalazine (**1e**) (35 mg, 0.2 mmol) and *tert*-butyl[(1-isopropoxyvinyl)oxy]dimethylsilane (**2a**) (102 μL, 0.4 mmol), **(*S*)-8ea** was obtained after purification by flash chromatography (*n*-hexane/EtOAc 5/1) as a colorless oil (59 mg, 77%, 40% ee). **$[\alpha]_D^{29}$ =** +171.6 (*c* 1, $CHCl_3$). **^{1}H-NMR** (300 MHz, $CDCl_3$): δ 7.68 – 7.64 (m, 2H), 7.49 (s, 1H), 7.47 – 7.36 (m, 3H), 6.87 (s, 1H), 6.75 (s, 1H), 6.24 – 6.19 (m, 1H), 6.02 – 6.00 (m, 2H), 4.98 (hept, *J* = 6.3 Hz, 1H), 2.74 (dd, *J* = 14.4, 5.2 Hz, 1H), 2.63 (dd, *J* = 14.4, 8.6 Hz, 1H), 1.23 (d, *J* = 6.3 Hz, 3H), 1.18 (dd, *J* = 6.3 Hz, 3H). **^{13}C-NMR** (75.5 MHz, $CDCl_3$): δ 170.3, 169.4, 150.4, 148.0, 142.6, 134.5, 130.8, 129.8, 128.2, 127.7, 118.3, 107.6, 105.9, 101.9, 68.5, 48.9, 39.3, 21.87, 21.86. **HRMS** (ESI) *m/z*: $[M+Na]^+$ Calcd for $C_{21}H_{20}O_5N_2Na$ 403.1264; found 403.1257. **HPLC** (Chiralpak IA, *n*-hexane/2-propanol 80:20, flow 1 mL/min) t_R 9.4 min (minor) and 18.3 min (major).

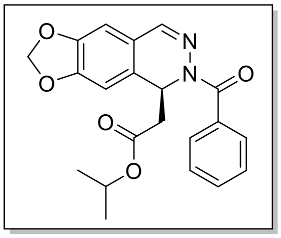

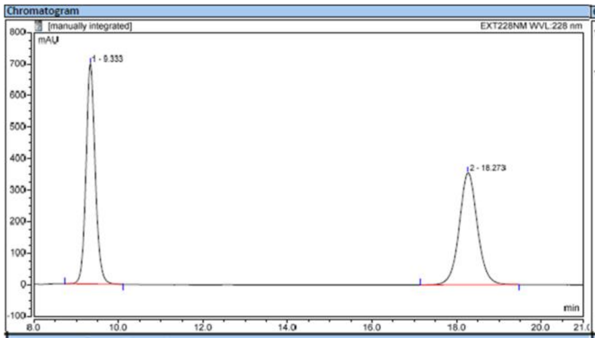

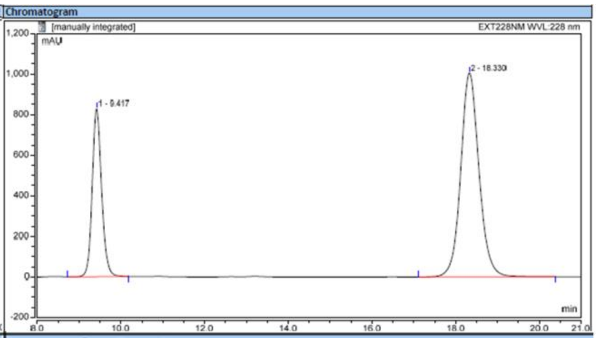

Integration Results

No.	Retention Time min	Area mAU*min	Relative Area %
1	9.333	176.521	50.02
2	18.273	176.376	49.98

Integration Results

No.	Retention Time min	Area mAU*min	Relative Area %
1	9.417	215.554	29.79
2	18.330	507.904	70.21

Isopropyl (*S*)-2-(2-benzoyl-5,8-dimethoxy-1,2-dihydrophthalazin-1-yl)acetate [(*S*)-8fa]: Following the general procedure **12**, starting from 5,8-dimethoxyphthalazine (**1f**) (38 mg, 0.2 mmol) and *tert*-butyl[(1-isopropoxyvinyl)oxy]dimethylsilane (**2a**) (102 μL, 0.4 mmol), **(*S*)-8fa** was obtained after purification by flash chromatography (*n*-hexane/EtOAc 5/1) as a yellow solid (56 mg, 70%, 14% ee).

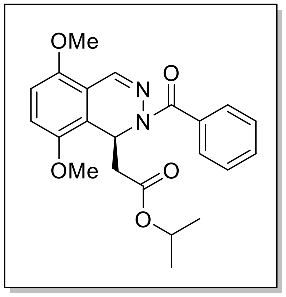

^{1}H-NMR (300 MHz, $CDCl_3$): δ 7.97 (s, 1H), 7.69 – 7.65 (m, 2H), 7.45 – 7.35 (m, 3H), 6.94 (d, *J* = 9.0 Hz, 1H), 6.80 (d, *J* = 9.0 Hz, 1H), 6.51 – 6.47 (m, 1H), 4.89 (hept, *J* = 6.3 Hz, 1H), 3.85 – 3.82 (m, 6H), 2.71 – 2.60 (m, 2H), 1.19 – 1.16 (m, 6H). **^{13}C-NMR** (75.5 MHz, $CDCl_3$): δ 170.6, 169.8, 149.7, 148.6, 138.6, 135.1, 130.4, 129.6, 127.6, 122.2, 114.3, 114.0, 110.5, 68.2, 56.0, 55.9, 44.4, 38.3, 21.81, 21.75. **HRMS** (ESI) *m/z*: $[M+Na]^+$ Calcd for $C_{22}H_{24}O_5N_2Na$ 419.1577; found 419.1571. **HPLC** (Chiralpak IA, *n*-hexane/2-propanol 90:10, flow 1 mL/min) t_R 14.8 min (major) and 17.1 min (minor).

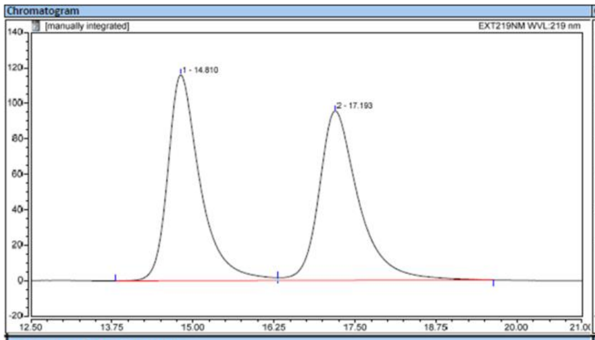

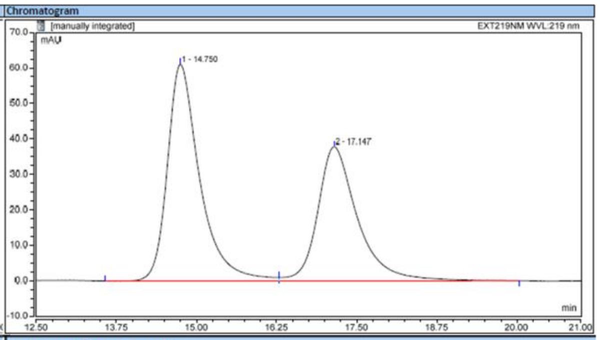

Integration Results

No.	Retention Time min	Area mAU*min	Relative Area %
1	14.810	67.122	49.83
2	17.193	67.588	50.17

Integration Results

No.	Retention Time min	Area mAU*min	Relative Area %
1	14.750	35.684	56.83
2	17.147	27.111	43.17

Isopropyl (*S*)-2-(3-benzoyl-3,4-dihydrobenzo[f]phthalazin-4-yl)acetate/ Isopropyl (*S*)-2-(2-benzoyl-1,2-dihydrobenzo[f]phthalazin-1-yl)acetate [(*S*)-8ga/8ga´]: Following the general procedure **12**, starting from benzo[f]phthalazine (**1g**) (36 mg, 0.2 mmol) and *tert*-butyl[(1-isopropoxyvinyl)oxy]dimethylsilane (**2a**) (102 µL, 0.4 mmol), **(*S*)-8ga/8ga´** was obtained after purification by flash chromatography (*n*-hexane/EtOAc 7/1) as a colorless oil (68 mg, 88%, 90% ee major, 59% ee minor, *regioisomer ratio* 2/1). **$[\alpha]_D^{28}$** = +428.0 (*c* 1, $CHCl_3$). **^{1}H-NMR** (300 MHz, $CDCl_3$): The compound exists as a ~2:1 mixture of regioisomers. *Signals corresponding to the major regioisomer:* δ 8.48 (s, 1H), 8.24 (d, *J* = 8.0 Hz, 1H), 7.98 (d, *J* = 8.4 Hz, 1H), 7.91 – 7.87 (m, 2H), 7.75 – 7.71 (m, 3H), 7.48 – 7.40 (m, 4H), 6.50 – 6.45 (m, 1H), 5.05 – 4.96 (m, 1H), 2.85 (dd, *J* = 14.4, 5.6 Hz, 1H), 2.75 (dd, *J* = 14.3, 8.1 Hz, 1H), 1.22 (d, *J* = 6.3 Hz, 3H), 1.16 (d, *J* = 6.3 Hz, 3H). *Representative signals corresponding to the minor regioisomer:* δ 8.32 (d, *J* = 8.5 Hz, 1H), 7.81 (s, 1H), 7.69 – 7.48 (m, 9H), 7.18 – 7.14 (m, 1H), 4.96 – 4.85 (m, 1H), 2.83 – 2.71 (m, 1H), 2.64 (dd, *J* = 13.3, 4.9 Hz, 1H), 1.20 (d, *J* = 6.2 Hz, 3H), 1.12 (d, *J* = 6.3 Hz, 3H). **^{13}C-NMR** (75.5 MHz, $CDCl_3$): *Signals corresponding to both regioisomers:* δ 170.8, 170.5, 169.6, 169.3, 143.6, 139.6, 134.6, 134.4, 133.4, 132.4, 131.8, 130.9, 130.7, 129.8, 129.7, 129.1, 129.10, 129.05, 128.2, 128.1, 127.8, 127.7, 126.7, 124.6, 123.2, 122.6, 121.5, 118.8, 68.7, 68.5, 49.3, 46.0, 39.2, 38.8, 21.83, 21.73, 21.6. **HRMS** (ESI) *m/z*: $[M+Na]^+$ Calcd for $C_{24}H_{22}O_3N_2Na$ 409.1523; found 409.1518. **HPLC** (Chiralpak IA, *n*-hexane/2-propanol 90:10, flow 1 mL/min) *Major regioisomer:* t_R 17.5 min (minor) and 24.5 min (major). *Minor regioisomer:* t_R 14.9 min (minor) and 20.6 min (major).

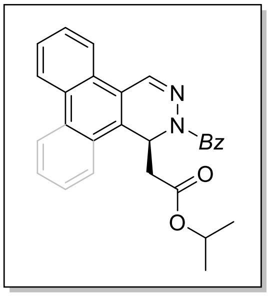

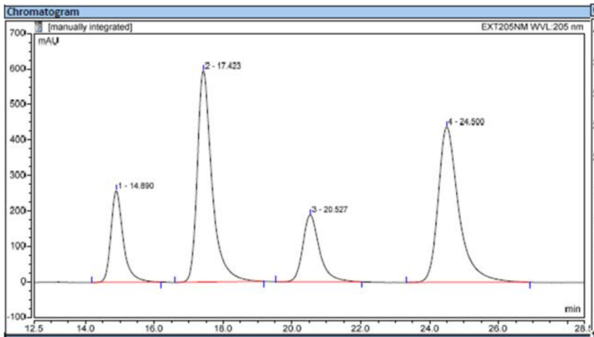

Integration Results			
No.	Retention Time min	Area mAU*min	Relative Area %
1	14.890	106.960	13.16
2	17.423	294.772	36.26
3	20.527	105.755	13.01
4	24.500	305.490	37.58

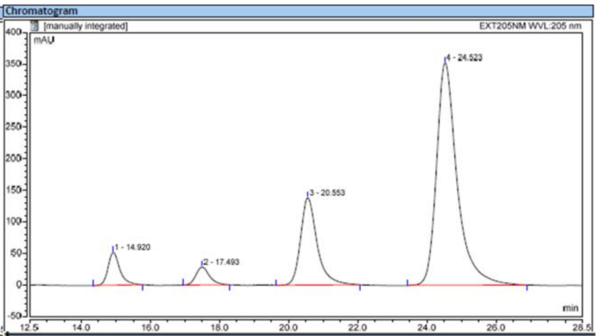

Integration Results			
No.	Retention Time min	Area mAU*min	Relative Area %
1	14.920	20.654	5.81
2	17.493	12.911	3.63
3	20.553	77.229	21.72
4	24.523	244.741	68.84

Isopropyl (*S*)-2-(2-benzoyl-7-chloro-1,2-dihydrophthalazin-1-yl)acetate [(*S*)-8ha]: Following the general procedure **12**, starting from 1-chlorophthalazine (**1h**) (33 mg, 0.2 mmol) and *tert*-butyl[(1-isopropoxyvinyl)oxy]dimethylsilane (**2a**) (102 µL, 0.4 mmol), **(*S*)-8ha** was obtained after purification by flash chromatography (*n*-hexane/EtOAc 7/1) as a colorless oil (50 mg, 68%, 94% ee). **$[\alpha]_D^{25}$** = +538.9 (*c* 1, $CHCl_3$). **^{1}H-NMR** (300 MHz, $CDCl_3$): δ 7.75 – 7.70 (m, 3H), 7.58 – 7.38 (m, 6H), 6.35 – 6.31 (m, 1H), 4.96 (hept, *J* = 6.3 Hz, 1H), 2.82 (dd, *J* = 14.6, 5.3 Hz, 1H), 2.72 (dd, *J* = 14.6, 8.4 Hz, 1H), 1.21 (d, *J* = 6.3 Hz, 3H), 1.14 (d, *J* = 6.3 Hz, 3H). **^{13}C-NMR** (126 MHz, $CDCl_3$): δ 169.4, 169.1, 142.6, 134.0, 133.5, 133.1, 131.3, 130.3, 129.1, 127.8, 127.0, 125.5, 123.6, 68.7, 49.9, 39.6, 21.9, 21.8. **HRMS** (ESI) *m/z*: $[M+Na]^+$ Calcd for $C_{20}H_{19}O_3N_2ClNa$ 393.0976; found 393.0974. **HPLC** (Chiralpak IC, *n*-hexane/2-propanol 90:10, flow 1 mL/min) t_R 14.4 min (minor) and 20.3 min (major).

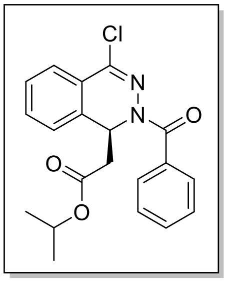

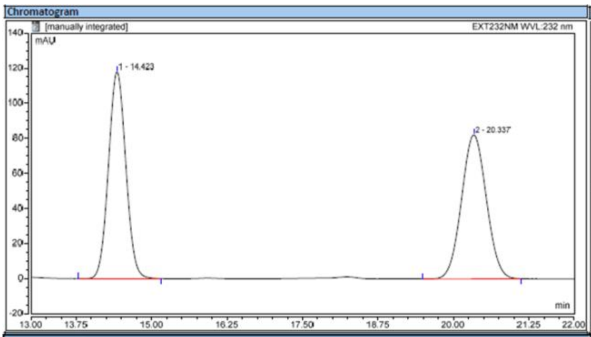

Integration Results

No.	Retention Time min	Area mAU*min	Relative Area %
1	14.423	39.247	50.05
2	20.337	39.163	49.95

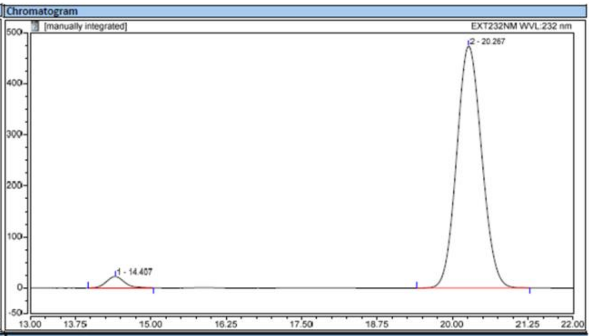

Integration Results

No.	Retention Time min	Area mAU*min	Relative Area %
1	14.407	7.605	3.23
2	20.267	228.021	96.77

Isopropyl (*S*)-2-(2-benzoyl-4-cyano-1,2-dihydrophthalazin-1-yl)acetate [(*S*)-8ia]: Following the general procedure **12**, starting from phthalazine-1-carbonitrile (**1i**) (31 mg, 0.2 mmol) and *tert*-butyl[(1-isopropoxyvinyl)oxy]dimethylsilane (**2a**) (102 µL, 0.4 mmol), **(*S*)-8ia** was obtained after purification by flash chromatography (*n*-hexane/EtOAc 5/1) as a colorless oil (36 mg, 50%, 89% ee). **$[\alpha]_D^{28}$** = +637.1 (*c* 1, $CHCl_3$). **^{1}H-NMR** (300 MHz, $CDCl_3$): δ 7.71 – 7.41 (m, 9H), 6.36 – 6.32 (m, 1H), 4.94 (hept, *J* = 6.3 Hz, 1H), 2.82 – 2.69 (m, 2H), 1.18 (d, *J* = 6.3 Hz, 3H), 1.13 (d, *J* = 6.3 Hz, 3H). **^{13}C-NMR** (75.5 MHz, $CDCl_3$): δ 170.1, 168.8, 133.5, 132.7, 131.9, 131.2, 130.3, 129.5, 128.0, 127.2, 126.7, 124.5, 121.8, 113.6, 68.9, 50.0, 40.1, 21.81, 21.78. **HRMS** (ESI) *m/z*: $[M+Na]^+$ Calcd for $C_{21}H_{19}O_3N_3Na$ 384.1319; found 384.1314. **HPLC** (Chiralpak IB, *n*-hexane/2-propanol 95:5, flow 1 mL/min) t_R 9.0 min (major) and 9.7 min (minor).

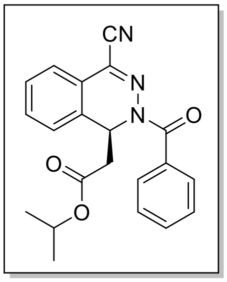

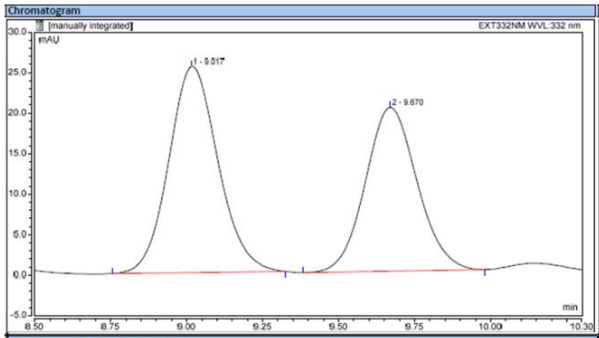

Integration Results

No.	Retention Time min	Area mAU*min	Relative Area %
1	9.017	4.776	54.56
2	9.670	3.978	45.44

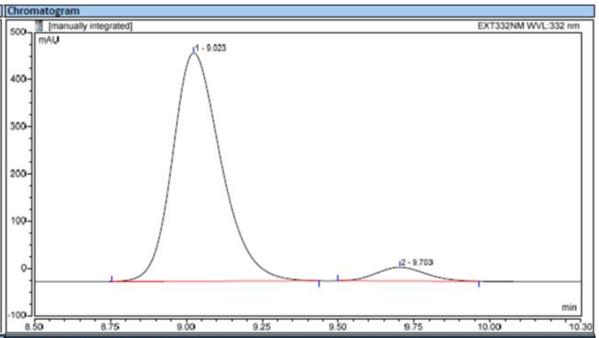

Integration Results

No.	Retention Time min	Area mAU*min	Relative Area %
1	9.023	89.668	94.33
2	9.703	5.390	5.67

Isopropyl (*S*)-2-(2-benzoyl-4-methoxy-1,2-dihydrophthalazin-1-yl)acetate [(*S*)-8ja]: Following the general procedure **12**, starting from 1-methoxyphthalazine (**1j**) (32 mg, 0.2 mmol) and *tert*-butyl[(1-isopropoxyvinyl)oxy]dimethylsilane (**2a**) (102 µL, 0.4 mmol), **(*S*)-8ja** was obtained after purification by flash chromatography (*n*-hexane/EtOAc 7/1) as a colorless oil (54 mg, 74%, 96% ee). **$[\alpha]_D^{28}$ =** +472.9 (*c* 1, $CHCl_3$). **^{1}H-NMR** (500 MHz, $CDCl_3$): δ 7.73 (d, *J* = 7.0 Hz, 2H), 7.67 (d, *J* = 7.6 Hz, 1H), 7.51 – 7.48 (m, 1H), 7.43 – 7.35 (m, 5H), 6.38 – 6.35 (m, 1H), 4.95 (dq, *J* = 12.5, 6.3 Hz, 1H), 3.75 (s, 3H), 2.81 (dd, *J* = 14.3, 5.7 Hz, 1H), 2.69 (dd, *J* = 14.3, 8.4 Hz, 1H), 1.21 (d, *J* = 6.3 Hz, 3H), 1.15 (d, *J* = 6.3 Hz, 3H). **^{13}C-NMR** (126 MHz, $CDCl_3$): δ 169.5, 169.0, 154.6, 135.8, 135.1, 132.2, 130.3, 129.7, 128.5, 127.4, 126.7, 123.9, 120.9, 68.5, 54.2, 49.4, 39.4, 21.91, 21.87. **HRMS** (ESI) *m/z*: $[M+Na]^+$ Calcd for $C_{21}H_{22}O_4N_2Na$ 389.1472; found 389.1468. **HPLC** (Chiralpak IB, *n*-hexane/2-propanol 95:5, flow 0.5 mL/min) t_R 14.8 min (minor) and 16.0 min (major).

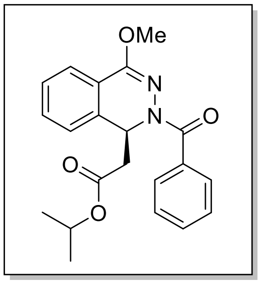

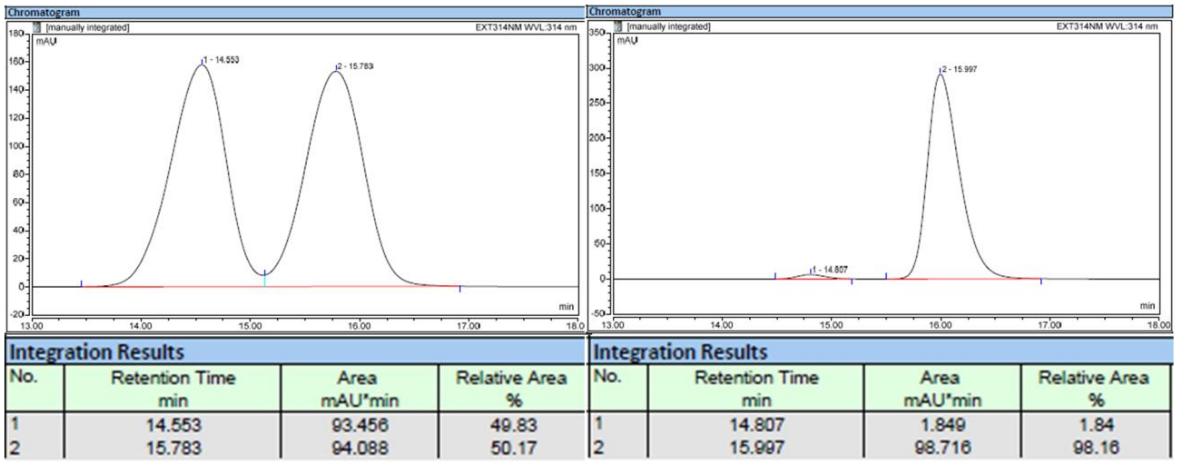

Integration Results

No.	Retention Time min	Area mAU*min	Relative Area %
1	14.553	93.456	49.83
2	15.783	94.088	50.17

Integration Results

No.	Retention Time min	Area mAU*min	Relative Area %
1	14.807	1.849	1.84
2	15.997	98.716	98.16

Isopropyl (*S*)-2-[2-benzoyl-4-(benzyloxy)-1,2-dihydrophthalazin-1-yl]acetate [(*S*)-8ka]: Following the general procedure **12**, starting from 1-(benzyloxy)phthalazine (**1k**) (47 mg, 0.2 mmol) and *tert*-butyl[(1-isopropoxyvinyl)oxy]dimethylsilane (**2a**) (102 µL, 0.4 mmol), **(*S*)-8ka** was obtained after purification by flash chromatography (*n*-hexane/EtOAc 6/1) as a yellow oil (50 mg, 57%, 97% ee). **$[\alpha]_D^{25}$ =** +396.2 (*c* 1, $CHCl_3$). **^{1}H-NMR** (300 MHz, $CDCl_3$): δ 7.75 – 7.67 (m, 3H), 7.54 – 7.48 (m, 1H), 7.45 – 7.27 (m, 10H), 6.41 – 6.37 (m, 1H), 5.12 (s, 2H), 4.97 (hept, *J* = 6.3 Hz, 1H), 2.83 (dd, *J* = 14.3, 5.8 Hz, 1H), 2.70 (dd, *J* = 14.3, 8.2 Hz, 1H), 1.22 (d, *J* = 6.3 Hz, 3H), 1.17 (d, *J* = 6.3 Hz, 3H). **^{13}C-NMR** (75.5 MHz, $CDCl_3$): δ 169.4, 169.1, 153.7, 136.5, 135.7, 135.1, 132.2, 130.2, 129.5, 128.6, 128.5, 128.1, 128.0, 127.5, 126.6, 124.0, 120.9, 68.5, 68.3, 49.3, 39.4, 21.9, 21.8. **HRMS** (ESI) *m/z*: $[M+Na]^+$ Calcd for $C_{27}H_{26}O_4N_2Na$ 465.1785; found 465.1776. **HPLC** (Chiralpak IA, *n*-hexane/2-propanol 90:10, flow 1 mL/min) t_R 10.9 min (major) and 13.2 min (minor).

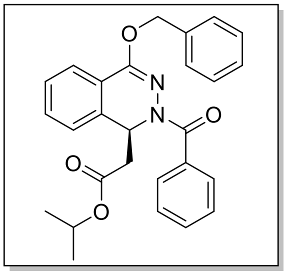

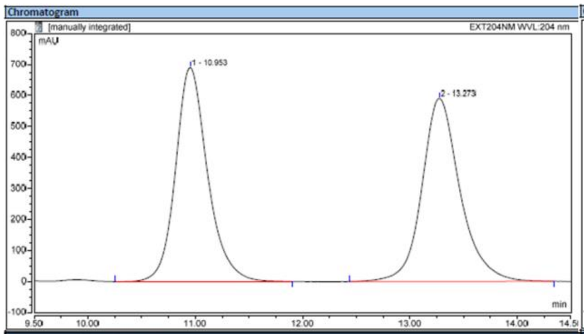

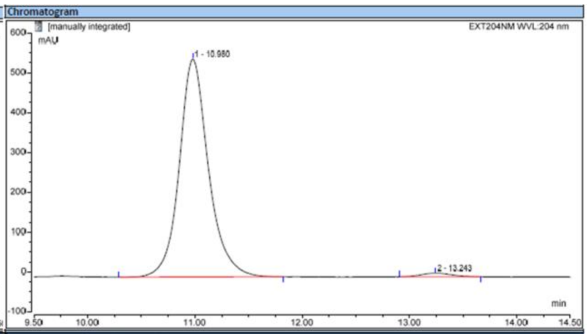

Integration Results

No.	Retention Time min	Area mAU*min	Relative Area %
1	10.953	238.542	49.60
2	13.273	242.432	50.40

Integration Results

No.	Retention Time min	Area mAU*min	Relative Area %
1	10.980	172.708	98.34
2	13.243	2.916	1.66

Isopropyl (*S*)-2-(2-benzoyl-4-phenyl-1,2-dihydrophthalazin-1-yl)acetate [(*S*)-8la]: Following the general procedure **12**, starting from 1-phenylphthalazine (**1l**) (41 mg, 0.2 mmol) and *tert*-butyl[(1-isopropoxyvinyl)oxy]dimethylsilane (**2a**) (102 μL, 0.4 mmol), **(*S*)-8la** was obtained after purification by flash chromatography (*n*-hexane/EtOAc 5/1) as a white solid (66 mg, 80%, 98% ee). **$[\alpha]_D^{25}$ =** +578.6 (*c* 1, $CHCl_3$). **^{1}H-NMR** (300 MHz, $CDCl_3$) δ 7.79 – 7.75 (m, 2H), 7.61 – 7.57 (m, 2H), 7.53 – 7.35 (m, 10H), 6.39 – 6.35 (m, 1H), 4.98 (hept, *J* = 6.2 Hz, 1H), 2.85 (dd, *J* = 14.5, 5.2 Hz, 1H), 2.71 (dd, *J* = 14.5, 9.1 Hz, 1H), 1.25 (d, *J* = 6.4 Hz, 3H), 1.16 (d, *J* = 6.3 Hz, 3H). **^{13}C-NMR** (126 MHz, $CDCl_3$): δ 170.4, 169.5, 152.5, 134.95, 134.88, 134.5, 131.8, 131.0, 130.4, 129.6, 129.0, 128.7, 128.5, 127.7, 127.6, 126.9, 124.4, 68.6, 49.3, 38.5, 22.0, 21.8. **HRMS** (ESI) *m/z*: $[M+Na]^+$ Calcd for $C_{26}H_{24}O_3N_2Na$ 435.1679; found 435.1674. **HPLC** (Chiralpak IA, *n*-hexane/2-propanol 90:10, flow 1 mL/min) t_R 8.8 min (major) and 10.3 min (minor).

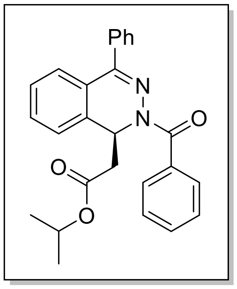

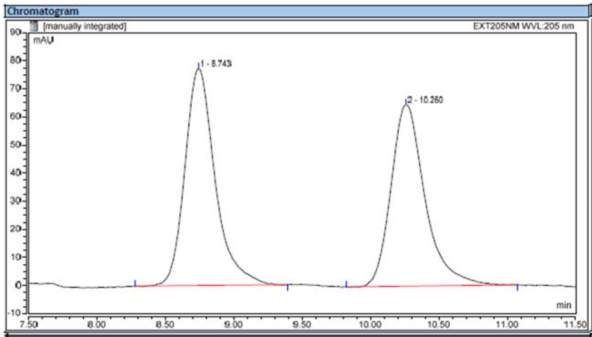

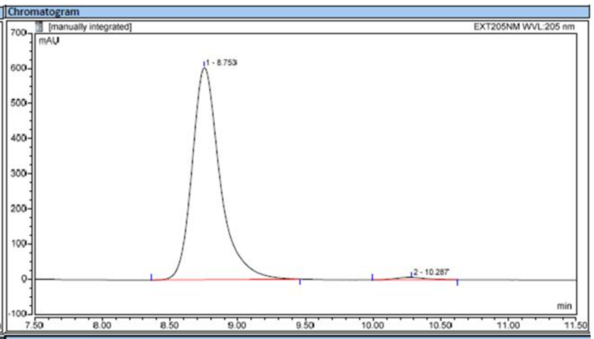

Integration Results

No.	Retention Time min	Area mAU*min	Relative Area %
1	8.743	19.749	51.49
2	10.260	18.609	48.51

Integration Results

No.	Retention Time min	Area mAU*min	Relative Area %
1	8.753	144.422	98.91
2	10.287	1.590	1.09

Isopropyl (*S*)-2-[2-benzoyl-4-(*o*-tolyl)-1,2-dihydrophthalazin-1-yl]acetate [(*S*)-8ma]: Following the general procedure **12**, starting from 1-(*o*-tolyl)phthalazine (**1m**) (44 mg, 0.2 mmol) and *tert*-butyl[(1-isopropoxyvinyl)oxy]dimethylsilane (**2a**) (102 μL, 0.4 mmol), **(*S*)-8ma** was obtained after purification by flash chromatography (*n*-hexane/EtOAc 7/1) as a colorless oil (49 mg, 57%, 94% ee). **$[\alpha]_D^{28}$ =** +467.6 (*c* 1, $CHCl_3$). **^{1}H-NMR** (300 MHz, $CDCl_3$) δ 7.75 – 7.70 (m, 2H), 7.49 – 7.29 (m, 8H), 7.28 – 7.23 (m, 2H), 6.97 (d, *J* = 7.5 Hz, 1H), 6.42 – 6.37 (m, 1H), 4.98 (hept, *J* = 6.3 Hz, 1H), 2.92 (dd, *J* = 14.2, 5.5 Hz, 1H), 2.76 (dd, *J* = 14.2, 9.1 Hz, 1H), 2.18 (s, 3H), 1.25 (d, *J* = 6.3 Hz, 3H), 1.15 (d, *J* = 6.3 Hz, 3H). **^{13}C-NMR** (75.5 MHz, $CDCl_3$): δ 170.6, 169.4, 153.2, 137.2, 134.6, 134.5, 133.6, 131.8, 130.9, 130.7, 130.1, 129.7, 129.3, 128.8, 127.7, 127.2, 126.24, 126.16, 125.5, 68.5, 49.2, 38.5, 22.0, 21.9, 20.5. **HRMS** (ESI) *m/z*: $[M+Na]^+$ Calcd for $C_{27}H_{26}O_3N_2Na$ 449.1836; found 449.1827. **HPLC** (Chiralpak IC, *n*-hexane/2-propanol 90:10, flow 1 mL/min) t_R 19.8 min (minor) and 25.2 min (major).

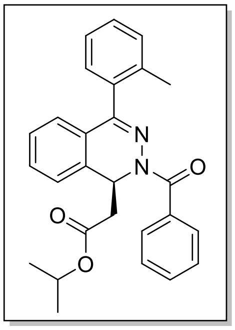

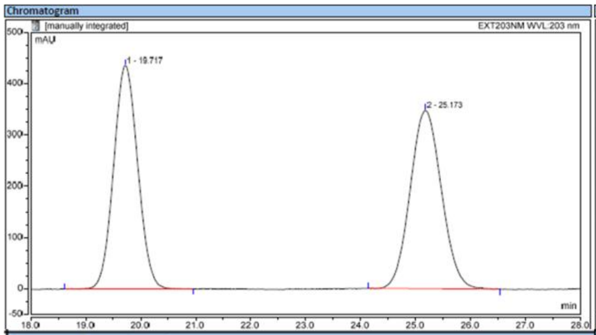

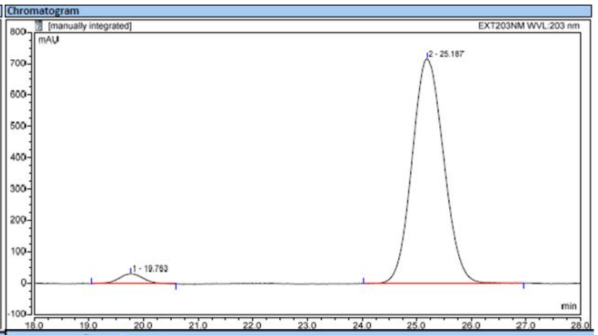

Integration Results

No.	Retention Time min	Area mAU*min	Relative Area %
1	19.717	220.690	49.05
2	25.173	229.226	50.95

Integration Results

No.	Retention Time min	Area mAU*min	Relative Area %
1	19.763	16.004	3.24
2	25.187	477.817	96.76

Isopropyl (*S*)-2-[4-(benzo[d][1,3]dioxol-5-yl)-2-benzoyl-1,2-dihydrophthalazin-1-yl]acetate [(*S*)-8na]: Following the general procedure **12**, starting from 1-(benzo[d][1,3]dioxol-5-yl)phthalazine (**1n**) (50 mg, 0.2 mmol) and *tert*-butyl[(1-isopropoxyvinyl)oxy]dimethylsilane (**2a**) (102 μL, 0.4 mmol), **(*S*)-8na** was obtained after purification by flash chromatography (*n*-hexane/EtOAc 5/1) as a white solid (71 mg, 77%, 95% ee). **$[\alpha]_D^{28}$ =** +677.5 (*c* 1, $CHCl_3$). **^{1}H-NMR** (300 MHz, $CDCl_3$) δ 7.75 – 7.72 (m, 2H), 7.52 – 7.36 (m, 7H), 7.12 – 7.09 (m, 1H), 7.06 – 7.05 (m, 1H), 6.85 (d, *J* = 8.0 Hz, 1H), 6.37 – 6.33 (m, 1H), 6.00 – 5.98 (m, 2H), 4.98 (hept, *J* = 6.2 Hz, 1H), 2.83 (dd, *J* = 14.6, 5.2 Hz, 1H), 2.69 (dd, *J* = 14.6, 9.1 Hz, 1H), 1.25 (d, *J* = 6.3 Hz, 3H), 1.16 (d, *J* = 6.3 Hz, 3H). **^{13}C-NMR** (75.5 MHz, $CDCl_3$): δ 170.3, 169.4, 152.1, 148.9, 148.0, 134.9, 131.7, 131.0, 130.2, 128.4, 127.7, 127.6, 127.0, 123.5, 109.1, 108.3, 101.5, 68.5, 49.2, 38.4, 21.94, 21.86. **HRMS** (ESI) *m/z*: $[M+Na]^+$ Calcd for $C_{27}H_{24}O_5N_2Na$ 479.1577; found 479.1573. **HPLC** (Chiralpak IB, *n*-hexane/2-propanol 90:10, flow 1 mL/min) t_R 7.5 min (major) and 9.3 min (minor).

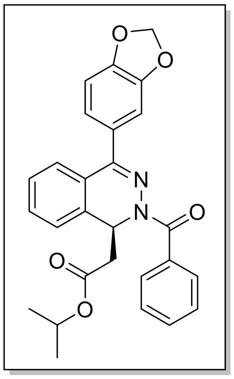

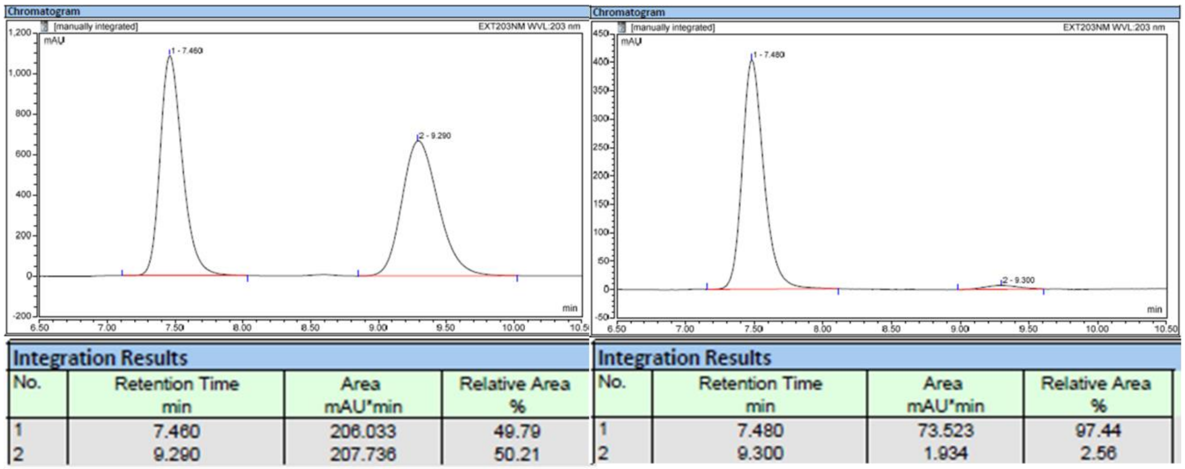

Integration Results

No.	Retention Time min	Area mAU*min	Relative Area %
1	7.460	206.033	49.79
2	9.290	207.736	50.21

Integration Results

No.	Retention Time min	Area mAU*min	Relative Area %
1	7.480	73.523	97.44
2	9.300	1.934	2.56

Isopropyl (*S*)-2-[2-benzoyl-4-(1H-pyrrol-1-yl)-1,2-dihydrophthalazin-1-yl]acetate [(*S*)-8oa]: Following the general procedure **12**, starting from 1-(1*H*-pyrrol-1-yl)phthalazine (**1o**) (39 mg, 0.2 mmol) and *tert*-butyl[(1-isopropoxyvinyl)oxy]dimethylsilane (**2a**) (102 μL, 0.4 mmol), **(*S*)-8oa** was obtained after purification by flash chromatography (*n*-hexane/EtOAc 5/1) as a colorless oil (60 mg, 75%, 91% ee). $[\alpha]_D^{28}$ **=** +576.9 (*c* 1, $CHCl_3$). **^{1}H-NMR** (300 MHz, $CDCl_3$) δ 7.74 – 7.70 (m, 2H), 7.59 – 7.40 (m, 7H), 7.07 – 7.06 (m, 2H), 6.42 – 6.37 (m, 1H), 6.33 – 6.32 (m, 2H), 4.97 (hept, *J* = 6.3 Hz, 1H), 2.85 (dd, *J* = 14.6, 5.4 Hz, 1H), 2.73 (dd, *J* = 14.7, 8.9 Hz, 1H), 1.24 (d, *J* = 6.3 Hz, 3H), 1.16 (d, *J* = 6.3 Hz, 3H). **^{13}C-NMR** (75.5 MHz, $CDCl_3$): δ 169.8, 169.3, 145.6, 135.6, 134.2, 132.7, 131.1, 130.0, 128.8, 127.8, 127.7, 126.0, 121.1, 120.8, 111.0, 68.7, 49.4, 38.3, 21.9, 21.8. **HRMS** (ESI) *m/z*: $[M+Na]^+$ Calcd for $C_{24}H_{23}O_3N_3Na$ 424.1632; found 424.1625. **HPLC** (Chiralpak IC, *n*-hexane/2-propanol 85:15, flow 1 mL/min) t_R 13.8 min (minor) and 20.3 min (major).

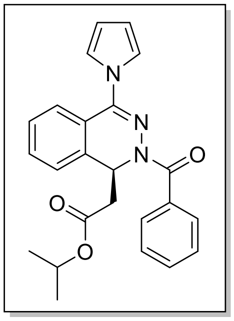

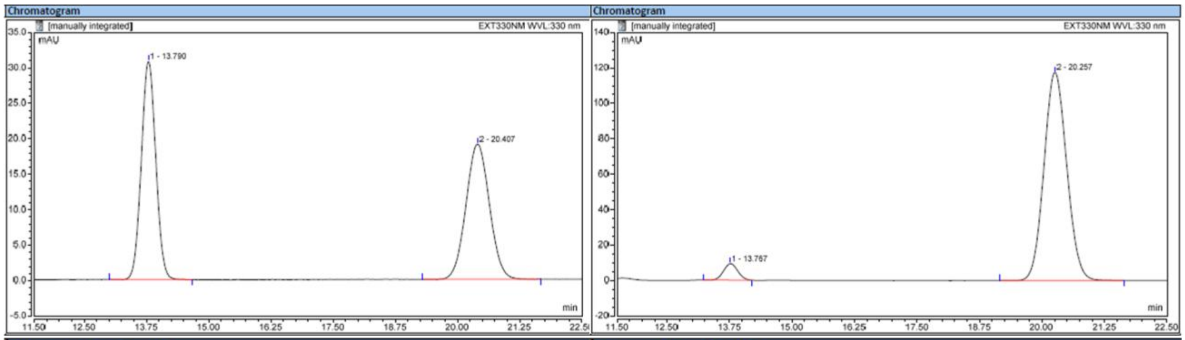

Integration Results

No.	Retention Time min	Area mAU*min	Relative Area %
1	13.790	10.349	49.89
2	20.407	10.394	50.11

Integration Results

No.	Retention Time min	Area mAU*min	Relative Area %
1	13.767	3.010	4.63
2	20.257	61.988	95.37

Isopropyl (*S*)-2-(2-benzoyl-4-morpholino-1,2-dihydrophthalazin-1-yl)acetate [(*S*)-8pa]: Following the general procedure **12**, starting from 4-(phthalazin-1-yl)morpholine (**1p**) (43 mg, 0.2 mmol) and *tert*-butyl[(1-isopropoxyvinyl)oxy]dimethylsilane (**2a**) (102 µL, 0.4 mmol), **(*S*)-8pa** was obtained after purification by flash chromatography (*n*-hexane/EtOAc 5/1) as a yellow oil (61 mg, 73%, 96% ee). **$[\alpha]_D^{28}$ =** +466.4 (*c* 1, $CHCl_3$). **^{1}H-NMR** (300 MHz, $CDCl_3$): δ 7.67 – 7.64 (m, 2H), 7.52 – 7.31 (m, 7H), 6.31 – 6.26 (m, 1H), 4.97 (hept, *J* = 6.3 Hz, 1H), 3.90 – 3.83 (m, 2H), 3.79 – 3.72 (m, 2H), 3.33 – 3.25 (m, 2H), 2.98 – 2.91 (m, 2H), 2.80 (dd, *J* = 14.5, 5.5 Hz, 1H), 2.66 (dd, *J* = 14.5, 9.0 Hz, 1H), 1.24 (d, *J* = 6.3 Hz, 3H), 1.18 (d, *J* = 6.3 Hz, 3H). **^{13}C-NMR** (126 MHz, $CDCl_3$): δ 169.6, 169.3, 155.0, 136.5, 135.0, 131.5, 130.4, 129.8, 128.4, 127.8, 127.4, 125.5, 121.4, 68.5, 66.6, 49.7, 48.9, 37.6, 22.0, 21.9. **HRMS** (ESI) *m/z*: $[M+Na]^+$ Calcd for $C_{24}H_{27}O_4N_3Na$ 444.1894; found 444.1892. **HPLC** (Chiralpak IA, *n*-hexane/2-propanol 90:10, flow 1 mL/min) t_R 14.8 min (minor) and 20.8 min (major).

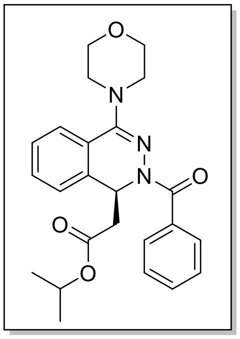

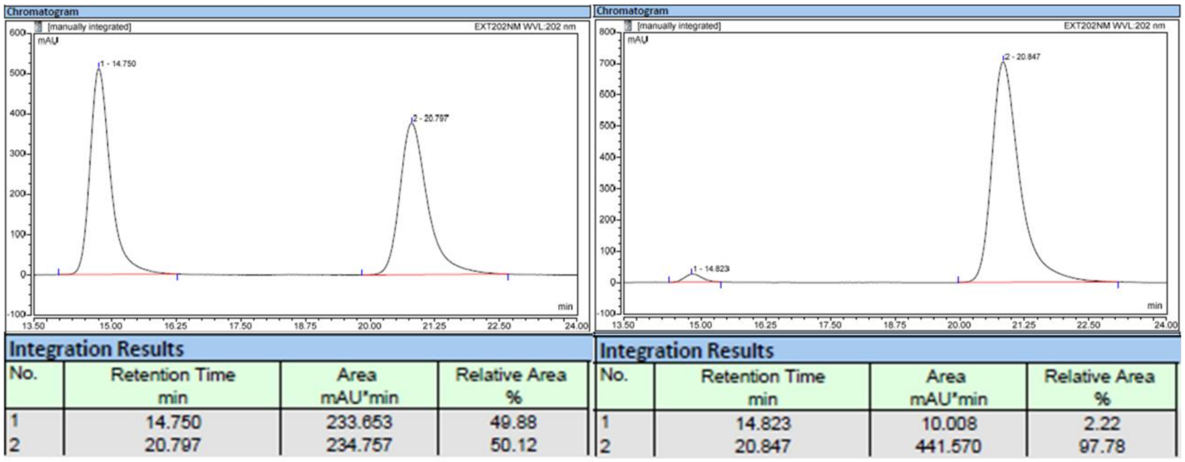

Integration Results

No.	Retention Time min	Area mAU*min	Relative Area %
1	14.750	233.653	49.88
2	20.797	234.757	50.12

Integration Results

No.	Retention Time min	Area mAU*min	Relative Area %
1	14.823	10.008	2.22
2	20.847	441.570	97.78

Isopropyl 2-{(*S*)-2-benzoyl-4-[(*S*)-2-benzoyl-1-(2-isopropoxy-2-oxoethyl)-1,2-dihydroisoquinolin-4-yl]-1,2-dihydrophthalazin-1-yl}acetate [(*S*,*S*)-8qa]: Following the general procedure **12**, starting from 1-(isoquinolin-4-yl)phthalazine (**1q**) (51 mg, 0.2 mmol), benzoyl chloride (48 µL, 0.4 mmol) and *tert*-butyl[(1-isopropoxyvinyl)oxy]dimethylsilane (**2a**) (127 µL, 0.5 mmol), **(*S*,*S*)-8qa** was obtained after purification by flash chromatography (*n*-hexane/EtOAc 3/1) as a white solid (97 mg, 71%, 98% ee major, 21% ee minor, dr = 19/1). *The product contains traces of the minor diastereoisomer*. **$[\alpha]_D^{28}$ =** +340.0 (*c* 1, $CHCl_3$). **^{1}H-NMR** (300 MHz, $CDCl_3$) δ 7.53 – 7.41 (m, 7H), 7.35 – 7.28 (m, 3H), 7.24 – 6.95 (m, 8H), 6.35 – 6.30 (m, 1H), 6.18 – 6.14 (m, 1H), 5.04 – 4.87 (m, 2H), 2.99 – 2.69 (m, 4H), 1.28 – 1.24 (m, 6H), 1.17 (d, *J* = 6.2 Hz, 3H), 1.12 (d, *J* = 6.3 Hz, 3H). **^{13}C-NMR** (75.5 MHz, $CDCl_3$): δ 170.3, 169.5, 169.3, 149.0, 134.1, 133.6, 133.4, 131.8, 131.7, 131.4, 131.1, 130.1, 129.8, 129.1, 128.7, 128.6, 128.3, 127.9, 127.5, 127.3, 127.2, 126.5, 124.8, 124.7, 116.6, 68.5, 52.4, 49.4, 39.1, 38.2, 22.03, 21.99, 21.9. **HRMS** (ESI) *m/z*: $[M+Na]^+$ Calcd for $C_{41}H_{39}O_6N_3Na$ 692.2731; found 692.2725. **HPLC** (Chiralpak IA, *n*-hexane/2-propanol 80:20, flow 1 mL/min) *Major diastereoisomer* t_R 18.9 min (major) and 36.8 min (minor). *Minor diastereoisomer* t_R 21.7 min (minor) and 30.6 min (major).

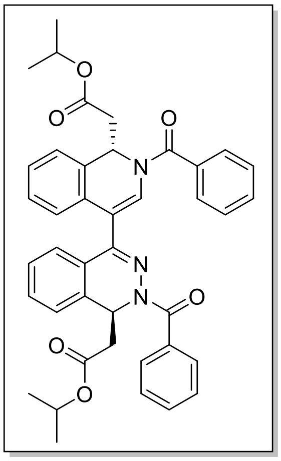

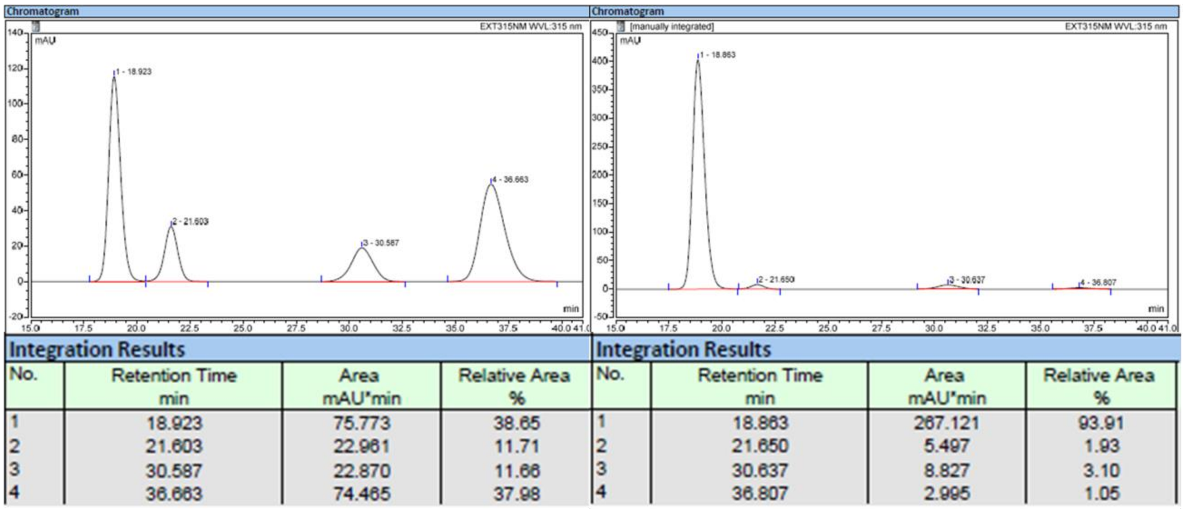

Integration Results

No.	Retention Time min	Area mAU*min	Relative Area %
1	18.923	75.773	38.65
2	21.603	22.961	11.71
3	30.587	22.870	11.66
4	36.663	74.465	37.98

Integration Results

No.	Retention Time min	Area mAU*min	Relative Area %
1	18.863	267.121	93.91
2	21.650	5.497	1.93
3	30.637	8.827	3.10
4	36.807	2.995	1.05

13. General procedure for the enantioselective dearomatization of pyridazine (13a)

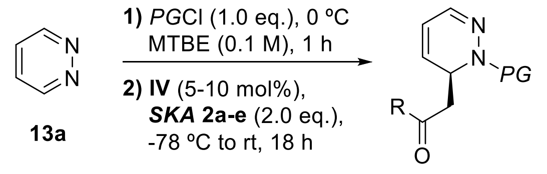

(*S*)-14aa-ae and (*S*)-15aa-ae

In a flame-dried Schlenk flask, the acylating reagent (0.2 mmol) was added to a solution of pyridazine (**13a**) (14 μL, 0.2 mmol) in freshly distilled anhydrous MTBE (2 mL, 0.1 M) at 0 ºC. The resulting suspension was stirred for 1 h at room temperature. Then, catalyst **IV** (5-10 mol%) was added and the reaction was cooled to −78 ºC (dry ice/acetone bath). Silyl ketene acetal **2a-e** (0.4 mmol) was added and the reaction mixture was stirred for 18 h and allowed to warm slowly to room temperature during that time. Then, the solvent was removed under reduced pressure and the residue purified by flash chromatography to afford the corresponding products **(*S*)-14aa-ae and (*S*)-15aa-ae**. Enantiomeric ratios were determined by HPLC analysis.

Racemic samples were prepared without catalyst following the general procedure describe above.

Isopropyl (*S*)-2-(2-benzoyl-2,3-dihydropyridazin-3-yl)acetate [(*S*)-14aa]: Following the general procedure **13**, employing benzoyl chloride (24 μL, 0.2 mmol), catalyst **IV** (5 mg, 5 mol%) and *tert*-butyl[(1-isopropoxyvinyl)oxy]dimethylsilane (**2a**) (102 μL, 0.4 mmol), **(*S*)-14aa** was obtained after purification by flash chromatography (*n*-hexane/EtOAc 5/1) as an orange oil (53 mg, 93%, 52% ee). **$[\alpha]_D^{23}$** = +441.2 (*c* 1, $CHCl_3$). **^{1}H-NMR** (300 MHz, $CDCl_3$): δ 7.66 – 7.62 (m, 2H), 7.48 – 7.35 (m, 3H), 7.08 – 7.07 (m, 1H), 6.47 – 6.41 (m, 1H), 6.00 – 5.96 (m, 1H), 5.72 – 5.66 (m, 1H), 5.02 (hept, *J* = 6.3 Hz, 1H), 2.76 – 2.62 (m, 2H), 1.24 (d, *J* = 6.3 Hz, 6H). **^{13}C-NMR** (75.5 MHz, $CDCl_3$): δ 170.8, 169.4, 140.2, 134.5, 132.2, 130.7, 129.7, 127.7, 117.7, 68.4, 46.2, 37.9, 21.9. **HRMS** (ESI) *m/z*: $[M+Na]^+$ Calcd for $C_{16}H_{18}O_3N_2Na$ 309.1210; found 309.1208. **HPLC** (Chiralpak IA, *n*-hexane/2-propanol 90:10, flow 1 mL/min) t_R 8.3 min (minor) and 9.5 min (major).

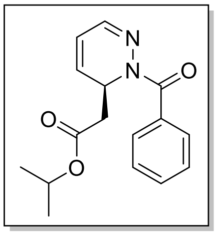

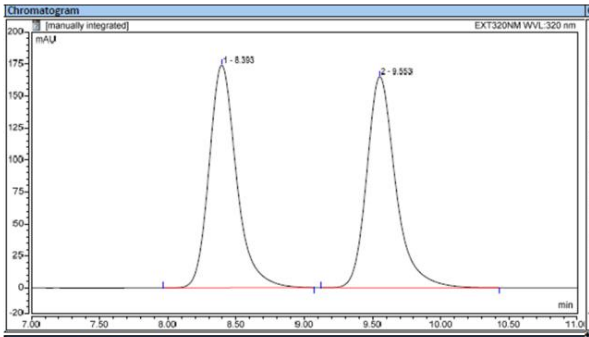

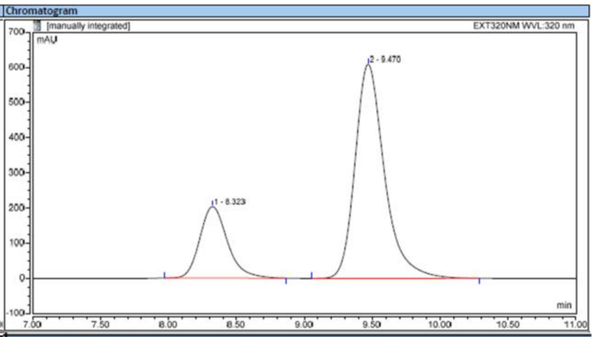

Integration Results

No.	Retention Time min	Area mAU*min	Relative Area %
1	8.393	40.576	49.97
2	9.553	40.626	50.03

Integration Results

No.	Retention Time min	Area mAU*min	Relative Area %
1	8.323	48.135	23.94
2	9.470	152.970	76.06

***tert*-Butyl (*S*)-2-(2-benzoyl-2,3-dihydropyridazin-3-yl)acetate [(*S*)-14ae]:** Following the general procedure **13**, employing benzoyl chloride (24 µL, 0.2 mmol), catalyst **IV** (5 mg, 5 mol%) and {[1-(*tert*-butoxy)vinyl]oxy}(*tert*-butyl)dimethylsilane (**2e**) (100 µL, 0.4 mmol), **(*S*)-14ae** was obtained after purification by flash chromatography (*n*-hexane/EtOAc 7/1) as a white solid (47 mg, 78%, 85% ee). **$[\alpha]_D^{28}$ =** +678.8 (*c* 1, $CHCl_3$). **^{1}H-NMR** (300 MHz, $CDCl_3$): δ 7.65 – 7.62 (m, 2H), 7.44 – 7.35 (m, 3H), 7.07 – 7.06 (m, 1H), 6.47 – 6.41 (m, 1H), 5.97 (dd, *J* = 9.6, 3.2 Hz, 1H), 5.69 – 5.62 (m, 1H), 2.72 – 2.58 (m, 2H), 1.46 (s, 9H). **^{13}C-NMR** (75.5 MHz, $CDCl_3$): δ 170.8, 169.2, 140.3, 134.6, 132.5, 130.7, 129.7, 127.7, 117.6, 81.3, 46.3, 38.8, 28.2. **HRMS** (ESI) *m/z*: $[M+Na]^+$ Calcd for $C_{17}H_{20}O_3N_2Na$ 323.1366; found 323.1365. **HPLC** (Chiralpak IA, *n*-hexane/2-propanol 90:10, flow 1 mL/min) t_R 6.7 min (minor) and 8.1 min (major).

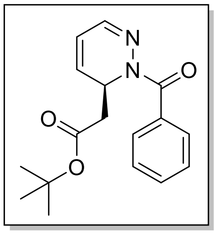

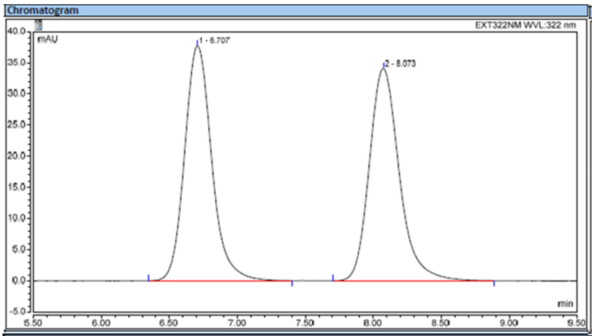

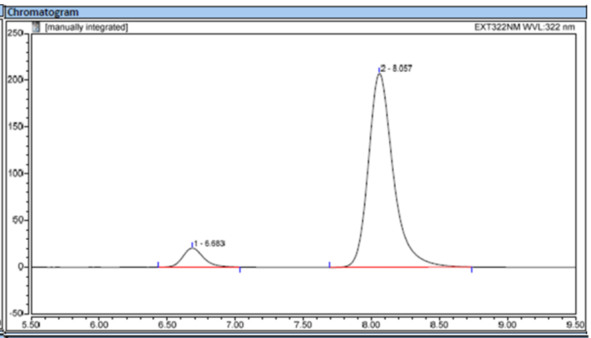

Integration Results

No.	Retention Time min	Area mAU*min	Relative Area %
1	6.707	8.720	50.03
2	8.073	8.709	49.97

Integration Results

No.	Retention Time min	Area mAU*min	Relative Area %
1	6.683	3.585	7.55
2	8.057	43.877	92.45

2,2,2-Trichloroethyl (*S*)-6-(2-isopropoxy-2-oxoethyl)pyridazine-1(6*H*)-carboxylate [(*S*)-15aa]: Following the general procedure **13**, employing 2,2,2-trichloroethyl chloroformate (28 µL, 0.2 mmol), catalyst **IV** (10 mg, 10 mol%) and *tert*-butyl[(1-isopropoxyvinyl)oxy]dimethylsilane (**2a**) (102 µL, 0.4 mmol), **(*S*)-15aa** was obtained after purification by flash chromatography (*n*-hexane/EtOAc 5/1) as a colorless oil (33 mg, 46%, 85% ee). $[\alpha]_D^{28}$ = +496.8 (*c* 1, $CHCl_3$). Lit. $[\alpha]_D^{20}$ = -245.0 [*c* 0.1, $CHCl_3$, 46% ee (*R*)]. **^{1}H-NMR** (300 MHz, $CDCl_3$): δ 7.23 (br s, 1H), 6.38 (ddd, *J* = 9.6, 6.1, 1.8 Hz, 1H), 5.96 (dd, *J* = 9.7, 3.2 Hz, 1H), 5.38 – 5.31 (m, 1H), 5.06 – 4.95 (m, 2H), 4.87 – 4.83 (m, 1H), 2.72 – 2.56 (m, 2H), 1.22 (d, *J* = 6.3 Hz, 6H). **^{13}C-NMR** (126 MHz, $CDCl_3$): δ 169.0, 152.8, 141.6, 132.1, 117.5, 95.1, 75.6, 68.6, 47.9, 38.1, 21.9. The spectroscopic data match those reported in the literature.[19] **HPLC** (Chiralpak IA, *n*-hexane/2-propanol 90:10, flow 1 mL/min) t_R 7.3 min (major) and 8.5 min (minor).

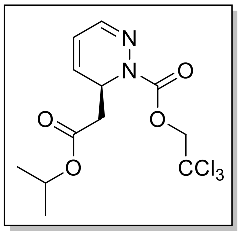

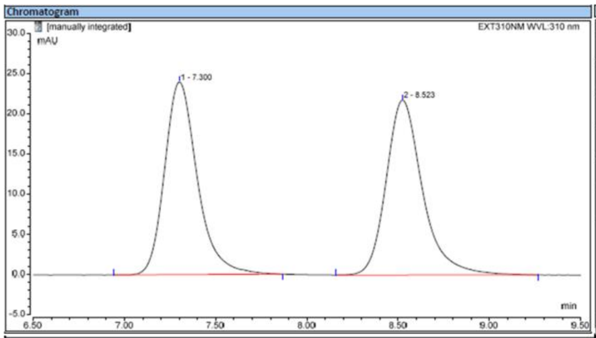

Integration Results

No.	Retention Time min	Area mAU*min	Relative Area %
1	7.300	4.981	49.76
2	8.523	5.030	50.24

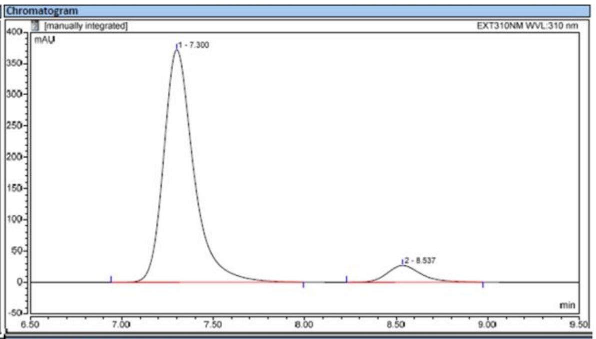

Integration Results

No.	Retention Time min	Area mAU*min	Relative Area %
1	7.300	70.875	92.53
2	8.537	5.725	7.47

2,2,2-Trichloroethyl (*S*)-6-(2-(benzyloxy)-2-oxoethyl)pyridazine-1(6*H*)-carboxylate [(*S*)-15ad]: Following the general procedure **13**, employing 2,2,2-trichloroethyl chloroformate (28 µL, 0.2 mmol), catalyst **IV** (10 mg, 10 mol%) and {[1-(benzyloxy)vinyl]oxy}(*tert*-butyl)dimethylsilane (**2d**) (200 µL, 0.8 mmol), **(*S*)-15ad** was obtained after purification by flash chromatography (*n*-hexane/EtOAc 4/1) as a yellow oil (45 mg, 55%, 90% ee). $[\alpha]_D^{25}$ = +418.7 (*c* 1, $CHCl_3$). **^{1}H-NMR** (300 MHz, $CDCl_3$): δ 7.41 – 7.32 (m, 5H), 7.25 (br s, 1H), 6.37 (ddd, *J* = 9.6, 6.1, 1.8 Hz, 1H), 5.97 (dd, *J* = 9.7, 3.2 Hz, 1H), 5.43 – 5.37 (m, 1H), 5.19 – 5.09 (m, 2H), 4.98 – 4.82 (m, 2H), 2.85 – 2.68 (m, 2H). **^{13}C-NMR** (126 MHz, $CDCl_3$): δ 169.4, 152.9, 141.5, 135.5, 131.8, 128.7, 128.6, 128.5, 117.7, 95.1, 75.6, 66.9, 47.9, 37.9. **HRMS** (ESI) *m/z*: $[M+Na]^+$ Calcd for $C_{16}H_{15}O_4N_2Cl_3Na$ 428.9990; found 428.9982. **HPLC** (Chiralpak IB, *n*-hexane/2-propanol 90:10, flow 1 mL/min) t_R 11.0 min (major) and 11.9 min (minor).

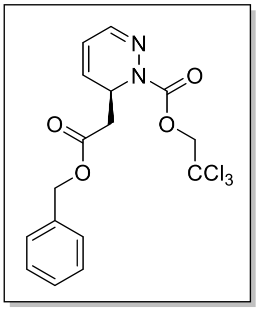

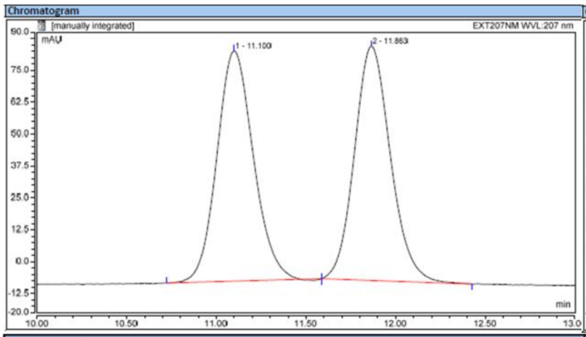

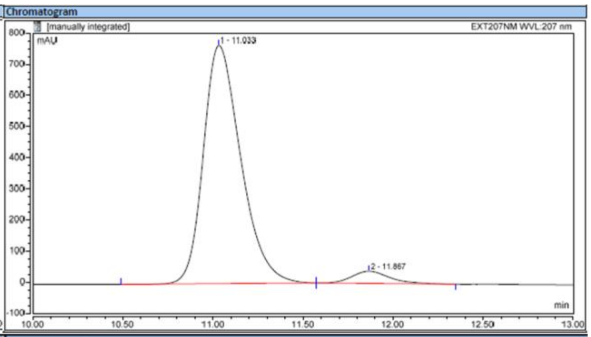

Integration Results

No.	Retention Time min	Area mAU*min	Relative Area %
1	11.100	21.283	49.86
2	11.863	21.405	50.14

Integration Results

No.	Retention Time min	Area mAU*min	Relative Area %
1	11.033	183.971	95.10
2	11.867	9.488	4.90

2,2,2-Trichloroethyl (*S*)-6-(2-(*tert*-butoxy)-2-oxoethyl)pyridazine-1(6*H*)-carboxylate [(*S*)-15ae]:

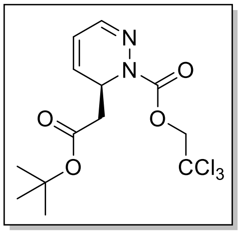

Following the general procedure **13**, employing 2,2,2-trichloroethyl chloroformate (28 μL, 0.2 mmol), catalyst **IV** (10 mg, 10 mol%) and {[1-(*tert*-butoxy)vinyl]oxy}(*tert*-butyl)dimethylsilane (**2e**) (200 μL, 0.8 mmol), **(*S*)-15ae** was obtained after purification by flash chromatography (*n*-hexane/EtOAc 5/1) as a white solid (23 mg, 31%, 95% ee). $\mathbf{[\alpha]_D^{25}}$ **=** +523.5 (*c* 1, $CHCl_3$). **^{1}H-NMR** (300 MHz, $CDCl_3$): δ 7.23 (br s, 1H), 6.39 (ddd, *J* = 9.7, 6.1, 1.8 Hz, 1H), 5.96 (dd, *J* = 9.8, 3.1 Hz, 1H), 5.34 – 5.28 (m, 1H), 4.99 – 4.80 (m, 2H), 2.72 – 2.53 (m, 2H), 1.44 (s, 9H). **^{13}C-NMR** (126 MHz, $CDCl_3$): δ 168.8, 141.6, 132.3, 117.5, 95.1, 81.6, 75.6, 48.2, 39.2, 28.2. **HRMS** (ESI) *m/z*: $[M+Na]^+$ Calcd for $C_{13}H_{17}O_4N_2Cl_3Na$ 393.0146; found 393.0147. **HPLC** (Chiralpak IA, *n*-hexane/2-propanol 90:10, flow 1 mL/min) t_R 6.5 min (major) and 8.3 min (minor).

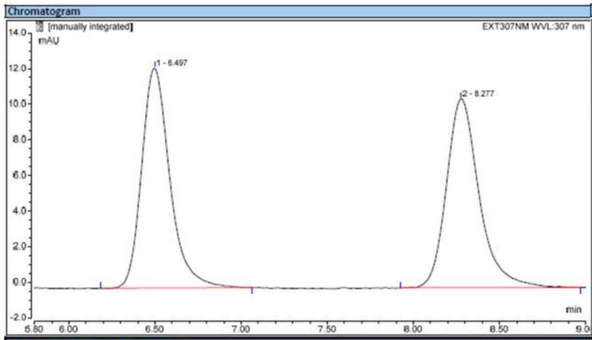

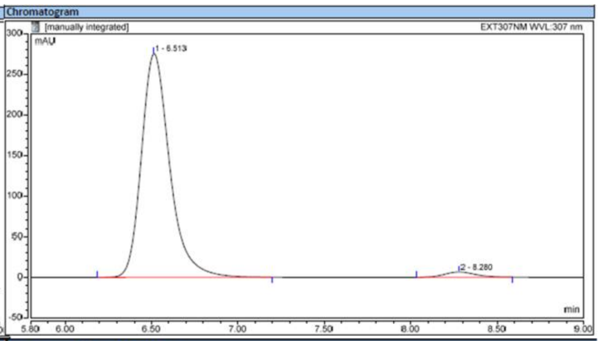

Integration Results

No.	Retention Time min	Area mAU*min	Relative Area %
1	6.497	2.338	49.99
2	8.277	2.338	50.01

Integration Results

No.	Retention Time min	Area mAU*min	Relative Area %
1	6.513	52.209	97.49
2	8.280	1.344	2.51

14. Derivatizations

14.1. Synthesis of isopropyl (*S*)-2-(2-benzoyl-1,2,3,4-tetrahydrophthalazin-1-yl)acetate [(*S*)-16]

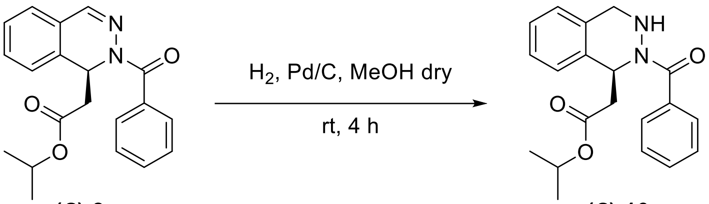

(*S*)-8aa (*S*)-16

A 25-mL round bottomed flask was charged with Pd/C (10% w/w) (43 mg, 0.04 mmol), methanol (2 mL), **(*S*)-8aa** (67 mg, 0.2 mmol, 88% ee) and sealed with a rubber septum. The head-space was evacuated and back-filled with hydrogen three times and then stirred under a balloon of hydrogen for 4 h at room temperature. After this time, the mixture was filtered through a celite pad, the solvent was removed under reduced pressure and the residue purified by flash chromatography (*n*-hex/EtOAc 3/1) to afford **(*S*)-16** as a colorless oil (53 mg, 78%, 86% ee). **$[\alpha]_D^{27}$ =** +152.2 (*c* 1, $CHCl_3$). **^{1}H-NMR** (300 MHz, Acetone-d^6) δ 7.68 – 7.54 (m, 2H), 7.47 – 7.31 (m, 4H), 7.29 – 7.20 (m, 2H), 7.14 – 7.11 (m, 1H), 5.91 (br s, 1H), 5.29 (br s, 1H), 4.92 (hept, *J* = 6.3 Hz, 1H), 4.16 – 4.00 (m, 1H), 3.95 – 3.88 (m, 1H), 3.12 – 3.04 (m, 1H), 2.99 – 2.91 (m, 1H), 1.19 (d, *J* = 6.3 Hz, 3H), 1.14 (d, *J* = 6.3 Hz, 3H). **^{13}C-NMR** (75.5 MHz, $CDCl_3$): δ 171.9, 171.5, 136.1, 133.5, 133.4, 129.9, 128.2, 127.7, 127.2, 126.7, 126.2, 68.6, 49.5, 47.7, 40.3, 21.7. **HRMS** (ESI) *m/z:* $[M+Na]^+$ Calcd for $C_{20}H_{22}O_3N_2Na$ 361.1523; found 361.1519. **HPLC** (Chiralpak IA, *n*-hexane/2-propanol 85:15, flow 1 mL/min) t_R 9.2 min (major) and 17.4 min (minor).

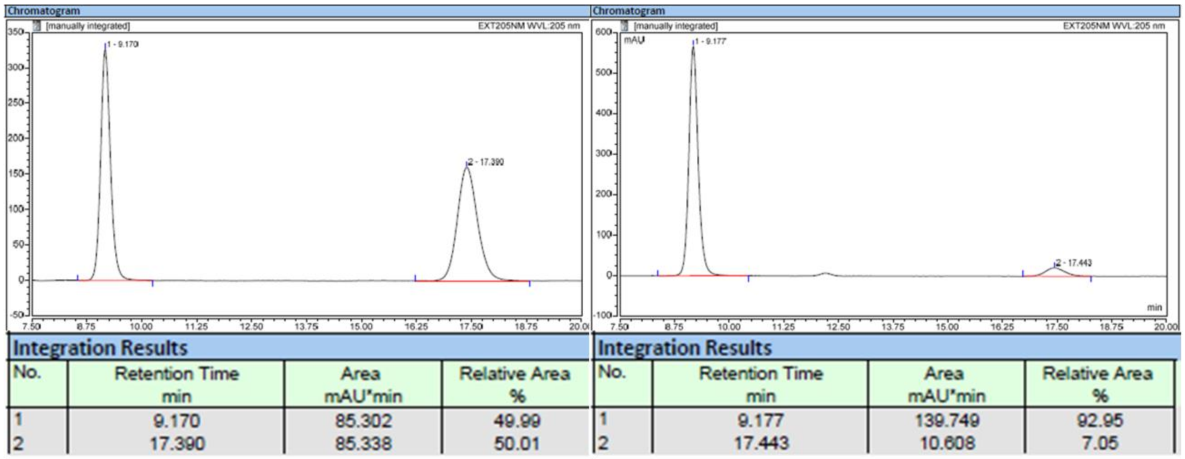

Integration Results

No.	Retention Time min	Area mAU*min	Relative Area %
1	9.170	85.302	49.99
2	17.390	85.338	50.01

Integration Results

No.	Retention Time min	Area mAU*min	Relative Area %
1	9.177	139.749	92.95
2	17.443	10.608	7.05

14.2. Synthesis of isopropyl (*S*)-2-(2-benzoyl-4-oxo-1,2,3,4-tetrahydrophthalazin-1-yl)acetate [(*S*)-17]

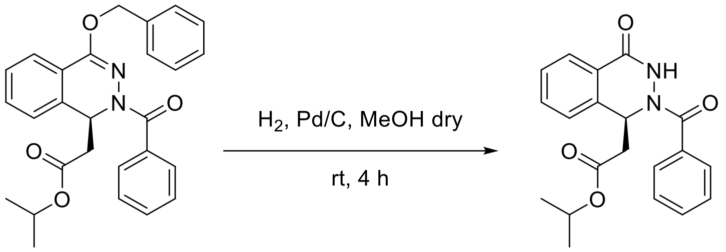

(*S*)-8ka **(*S*)-17**

A 25-mL round bottomed flask was charged with Pd/C (10% w/w) (43 mg, 0.04 mmol), methanol (2 mL), **(*S*)-8ka** (88 mg, 0.2 mmol, 97% ee) and sealed with a rubber septum. The head-space was evacuated and back-filled with hydrogen three times and then stirred under a balloon of hydrogen for 4 h at room temperature. After this time, the mixture was filtered through a celite pad, the solvent was removed under reduced pressure and the residue purified by flash chromatography (*n*-hex/EtOAc 1/1) to afford **(*S*)-17** as a white solid (59 mg, 84%, 96% ee). **$[\alpha]_D^{27}$ =** +294.5 (*c* 1, $CHCl_3$). **^{1}H-NMR** (300 MHz, $CDCl_3$) δ 8.83 (s, 1H), 8.10 (d, *J* = 8.8 Hz, 1H), 7.57 – 7.51 (m, 1H), 7.50 – 7.38 (m, 6H), 7.24 (d, *J* = 7.4 Hz, 1H), 5.84 (br s, 1H), 4.97 (hept, *J* = 6.3 Hz, 1H), 3.02 (dd, *J* = 15.3, 8.3 Hz, 1H), 2.72 (dd, *J* = 15.3, 6.2 Hz, 1H), 1.22 (d, *J* = 6.3 Hz, 3H), 1.14 (d, *J* = 6.3 Hz, 3H). **^{13}C-NMR** (75.5 MHz, $CDCl_3$): δ 169.1, 168.4, 163.6, 139.2, 133.6, 132.8, 131.4, 129.0, 128.9, 128.8, 128.0, 125.8, 125.0, 69.0, 55.1, 39.2, 21.85, 21.78. **HRMS** (ESI) *m/z*: $[M+Na]^+$ Calcd for $C_{20}H_{20}O_4N_2Na$ 375.1315; found 375.1311. **HPLC** (Chiralpak ID, *n*-hexane/2-propanol 75:25, flow 1 mL/min) t_R 29.0 min (minor) and 34.9 min (major).

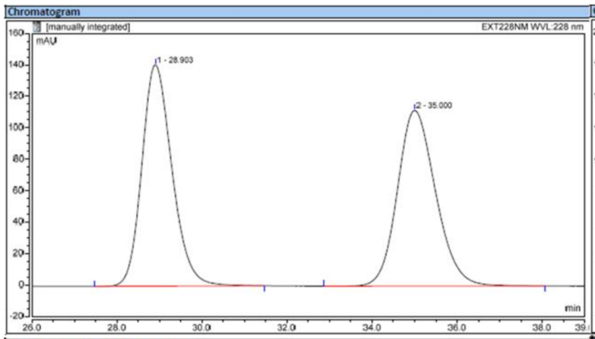

Integration Results

No.	Retention Time min	Area mAU*min	Relative Area %
1	28.903	115.493	49.87
2	35.000	116.083	50.13

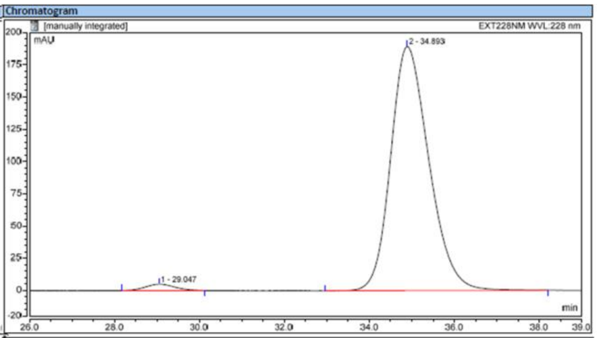

Integration Results

No.	Retention Time min	Area mAU*min	Relative Area %
1	29.047	3.888	1.93
2	34.893	197.132	98.07

14.3. Synthesis of isopropyl (*S*)-2-{2-benzoyl-4-[(4-bromophenyl)ethynyl]-1,2-dihydrophthalazin-1-yl}acetate [(*S*)-18]

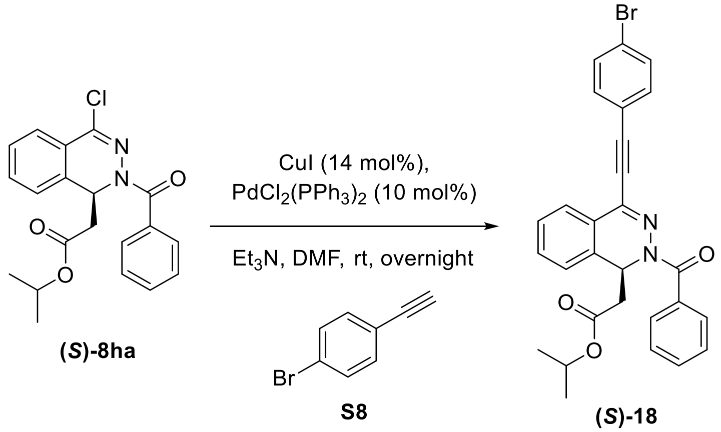

In a flame-dried schlenk flask, $PdCl_2(PPh_3)_2$ (27 mg, 0.038 mmol), CuI (10 mg, 0.053 mmol) and Et_3N (80 µL, 0.57 mmol) was added to a solution of **(*S*)-8ha** (142 mg, 0.38 mmol, 93% ee) in DMF (190 µL). Subsequently, addition of **S8** in three portions (0.55 mmol/2 h) was performed and the reaction mixture was stirred at room temperature overnight. Then, EtOAc (15 mL) and H_2O (10 mL) were added. The organic layer was washed with NaCl (3 x 10 mL), dried over $MgSO_4$ and the solvent was removed under reduced pressure. The resulting residue was purified by flash chromatography (*n*-hexane/EtOAc 5/1) (151 mg, 77%, 92% ee) to afford **(*S*)-18** as a yellow foam. Pure enantiomer of **(*S*)-18** was obtained after purification by chiral HPLC (Chiralpak IC, *n*-hexane/2-propanol 90:10, flow 1 mL/min) (134 mg, 68%, 99% ee). **$[\alpha]_D^{27}$** = +663.5 (*c* 1, $CHCl_3$). **^{1}H-NMR** (300 MHz, $CDCl_3$) δ 7.83 – 7.74 (m, 3H), 7.55 – 7.39 (m, 10H), 6.39 – 6.34 (m, 1H), 4.98 (hept, *J* = 6.3 Hz, 1H), 2.83 (dd, *J* = 14.5, 5.2 Hz, 1H), 2.71 (dd, *J* = 14.5, 8.6 Hz, 1H), 1.22 (d, *J* = 6.3 Hz, 3H), 1.16 (d, *J* = 6.3 Hz, 3H). **^{13}C-NMR** (75.5 MHz, $CDCl_3$): δ 169.7, 169.1, 136.5, 133.7, 133.5, 132.5, 132.2, 131.9, 131.2, 130.4, 128.9, 127.8, 126.8, 125.6, 124.3, 124.0, 120.7, 92.0, 84.7, 68.5, 49.5, 39.5, 21.84, 21.80. **HRMS** (ESI) *m/z*: $[M+Na]^+$ Calcd for $C_{28}H_{23}O_3N_2BrNa$ 537.0784; found 537.0778. **HPLC** (Chiralpak IC, *n*-hexane/2-propanol 90:10, flow 1 mL/min) t_R 19.4 min (minor) and 24.9 min (major).

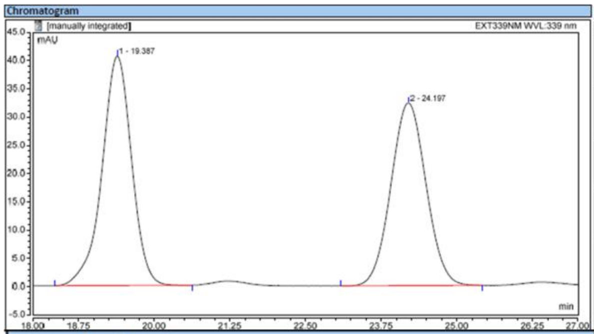

Integration Results

No.	Retention Time min	Area mAU*min	Relative Area %
1	19.387	22.996	51.40
2	24.197	21.742	48.60

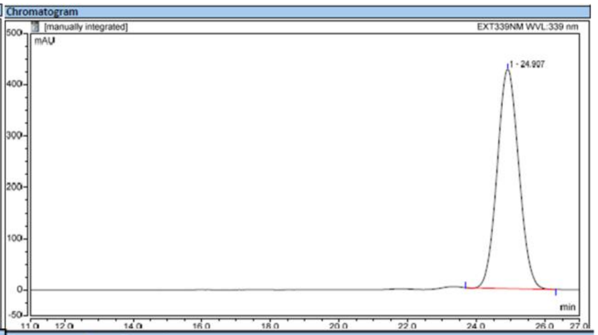

Integration Results

No.	Retention Time min	Area mAU*min	Relative Area %
1	24.907	311.931	100.00

14.4. Synthesis of 2-oxo-2-phenylethyl (*S*)-2-(1,2-dihydrophthalazin-1-yl)acetate [(*S*)-19]

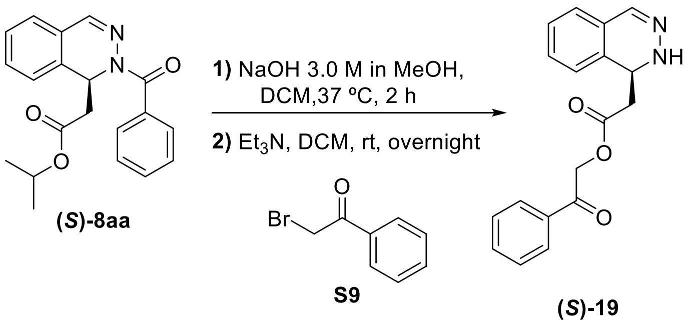

Step 1: NaOH 3.0 M in MeOH (0.4 mL) was added to a solution of **(*S*)-8aa** (134 mg, 0.4 mmol, 88% ee) in DCM (3.6 mL) and the mixture was stirred at 37 °C in an oil bath for 2 h. After this time, the solvent was removed under reduced pressure and EtOAc (2 mL) and H_2O (2 mL) were added. The aqueous layer was acidified with aq. $NaHSO_4$ (to pH ~2) and extracted with EtOAc (3 x 3 mL). The organic layer was dried over $MgSO_4$ and the solvent was removed under reduced pressure to afford the corresponding acid **(*S*)-8aa'**, which was used in the next step without further purification.

Step 2: To a solution of **(*S*)-8aa'** (~0.4 mmol) in DCM (4 mL) was added Et_3N (112 µL, 0.8 mmol) and **S9** (130 mg, 0.8 mmol). The reaction mixture was stirred at room temperature overnight. Then, the solvent was removed under reduced pressure and the residue was purified by flash chromatography (*n*-hex/EtOAc 2/1) to afford **(*S*)-19** as a yellow solid (63 mg, 51%, 88% ee). $[\alpha]_D^{27}$ = +21.6 (*c* 1, $CHCl_3$). **^{1}H-NMR** (300 MHz, $CDCl_3$) δ 7.95 – 7.92 (m, 2H), 7.66 – 7.60 (m, 1H), 7.53 – 7.48 (m, 3H), 7.40 – 7.30 (m, 2H), 7.19 – 7.13 (m, 2H), 5.44 (s, 2H), 4.90 – 4.85 (m, 1H), 3.17 (dd, J = 15.2, 10.6 Hz, 1H), 2.45 (dd, J = 15.3, 3.2 Hz, 1H). **^{13}C-NMR** (75.5 MHz, $CDCl_3$): δ 192.2, 170.7, 138.5, 134.3, 134.0, 132.6, 130.5, 129.1, 128.3, 128.0, 125.4, 125.1, 124.7, 66.5, 51.2, 37.9. **HRMS** (ESI) *m/z:* $[M+H]^+$ Calcd for $C_{18}H_{17}O_3N_2$ 309.1234; found 309.1235. **HPLC** (Chiralpak IB, *n*-hexane/2-propanol 70:30, flow 1 mL/min) t_R 14.3 min (minor) and 18.5 min (major).

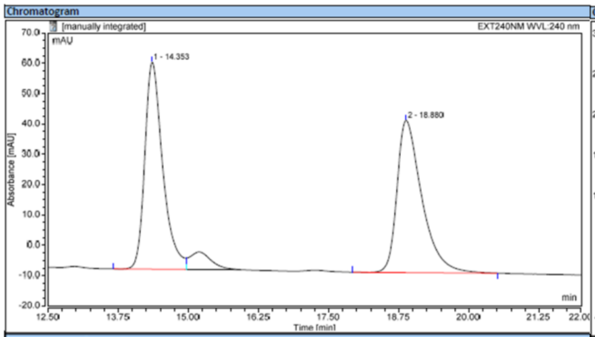

Integration Results

No.	Retention Time min	Area mAU*min	Relative Area %
1	14.353	25.291	49.55
2	18.880	25.751	50.45

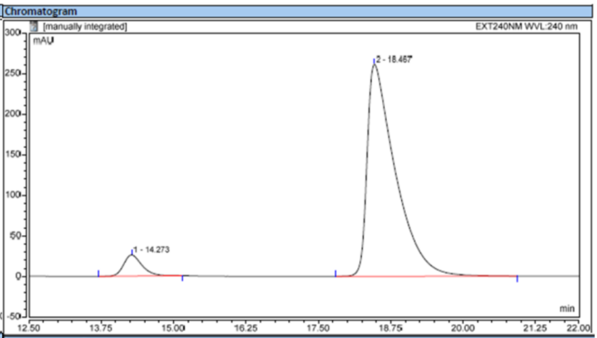

Integration Results

No.	Retention Time min	Area mAU*min	Relative Area %
1	14.273	9.534	6.08
2	18.467	147.253	93.92

14.5. Synthesis of ethyl {2-[(*S*)-2-benzoyl-4-phenyl-1,2-dihydrophthalazin-1-yl]acetyl}-L-phenylalaninate [(*S*,*S*)-20]

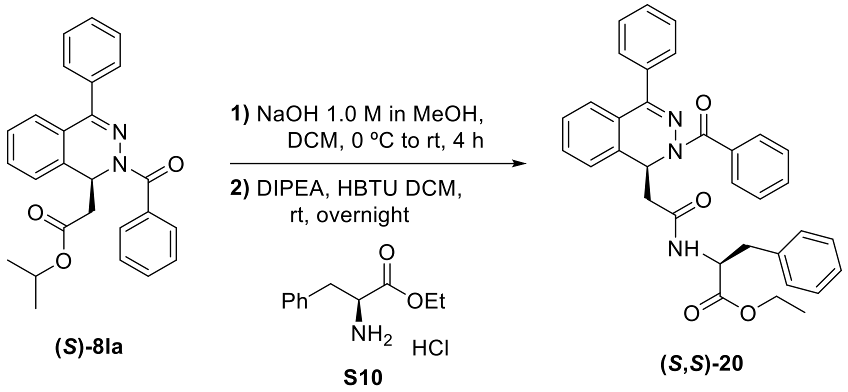

Step 1: NaOH 1.0 M in MeOH (0.3 mL) was added to a solution of **(*S*)-8la** (124 mg, 0.3 mmol, 98% ee) in DCM (2.7 mL) and the mixture was stirred from 0 °C to room temperature for 4 h. After this time, the solvent was evaporated under reduced pressure and EtOAc (2 mL) and H_2O (2 mL) were added. The aqueous layer was acidified with aq. $NaHSO_4$ (to pH ~2) and extracted with EtOAc (3 x 3 mL). The organic layer was dried over $MgSO_4$ and the solvent was removed under reduced pressure to afford the corresponding acid **(*S*)-8la'**, which was used in the next step without further purification.

Step 2: L-Phenylalanine ethyl ester hydrochloride (**S10**) (83 mg, 0.36 mmol) was added to a solution of HBTU (126 mg, 0.33 mmol), acid **(*S*)-8la'** (~0.3 mmol) and diisopropylethylamine (127 µL, 0.72 mmol) in dry CH_2Cl_2 (4.4 mL). The resulting mixture was stirred under Ar at room temperature for 16 h. The reaction was diluted with EtOAc (10 mL) and washed with HCl (0.5 M, 2 x 6 mL), saturated aqueous $NaHCO_3$ (2 x 6 mL) and brine (2 x 6 mL), dried over $MgSO_4$ and the solvent was removed under reduced pressure. The resulting residue was purified by flash chromatography on silica gel (DCM/Acetone 20/1) to give **(*S*,*S*)-20** as a white foam (109 mg, 67%, 98% dr). **$[\alpha]_D^{26}$ =** +508.7 (*c* 1, $CHCl_3$). **^{1}H-NMR** (300 MHz, $CDCl_3$) δ 7.80 – 7.77 (m, 2H), 7.60 – 7.57 (m, 2H), 7.52 – 7.27 (m, 11H), 7.26 – 7.19 (m, 3H), 7.13 – 7.10 (m, 1H), 6.41 (d, *J* = 8.0, 1H), 6.21 – 6.16 (m, 1H), 4.86 – 4.79 (m, 1H), 4.12 (q, *J* = 7.1 Hz, 1H), 3.20 (dd, *J* = 13.8, 5.8 Hz, 1H), 3.08 (dd, *J* = 13.8, 7.1 Hz, 1H), 2.80 (dd, *J* = 14.1, 4.6 Hz, 1H), 2.58 (dd, *J* = 14.1, 8.9 Hz, 1H), 1.20 (t, *J* = 7.1 Hz, 2H). **^{13}C-NMR** (75.5 MHz, $CDCl_3$): δ 171.6, 170.6, 168.6, 152.8, 136.3, 135.0, 134.8, 134.3, 131.9, 131.2, 130.5, 129.7, 129.6, 129.0, 128.7, 128.6, 128.4, 127.7, 127.5, 127.2, 126.9, 123.9, 61.5, 53.6, 50.0, 41.0, 38.2, 14.2. **HRMS** (ESI) *m/z*: $[M+Na]^+$ Calcd for $C_{34}H_{31}O_4N_3Na$ 568.2207; found 568.2197. **HPLC** (Chiralpak IA, *n*-hexane/2-propanol 90:10, flow 1 mL/min) t_R 14.9 min (major) and 18.7 min (minor).

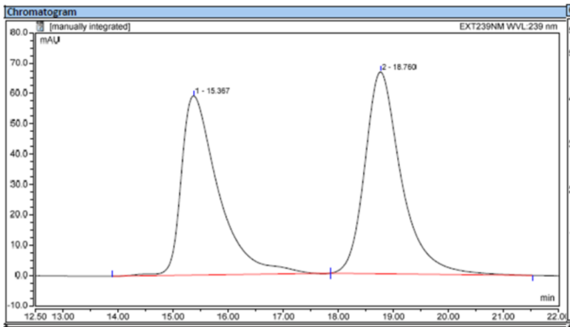

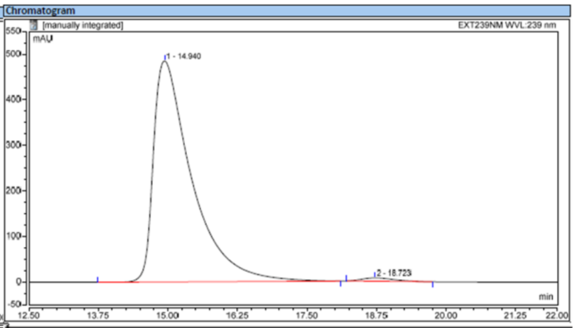

Integration Results

No.	Retention Time min	Area mAU*min	Relative Area %
1	15.367	45.898	47.39
2	18.760	50.958	52.61

Integration Results

No.	Retention Time min	Area mAU*min	Relative Area %
1	14.940	386.701	98.77
2	18.723	4.819	1.23

Crystallization of **(*S*,*S*)-20** by slow diffusion of *n*-pentane in a solution of **(*S*,*S*)-20** in DCM afforded crystals which are suitable of X-ray analysis. Mp = 135-137 °C.

Crystal Data for **(*S*,*S*)-20**

Low-temperature diffraction data were collected on a Bruker D8 Quest APEX-III single crystal diffractometer with a Photon III detector and a IμS 3.0 microfocus X-ray source at the Instituto de Investigaciones Químicas, Sevilla. Data were collected by means of ω and φ scans using monochromatic radiation λ(Mo Kα1) = 0.71073 Å. The diffraction images collected were processed and scaled using APEX-4 v2021.4-0 software. The structures were solved with SHELXT and was refined against F2 on all data by full-matrix least squares with SHELXL [1], using Olex2 [2] as graphical interface. All non-hydrogen atoms were refined anisotropically. Hydrogen atoms were included in the model at geometrically calculated positions and refined using a riding model, unless otherwise noted. The isotropic displacement parameters of all hydrogen atoms were fixed to 1.2 times the U value of the atoms to which they are linked (1.5 times for methyl groups).

$C_{137}H_{126}Cl_2N_{12}O_{16}$ (*M* =2267.39 g/mol): triclinic,
Space group P1 (no. 1),
a = 10.1882(6) Å,
b = 16.9193(9) Å,
c = 17.7221(11) Å,
α = 90.495(2)°,
β = 105.515(2)°,
γ = 100.234(2)°,
V = 2891.7(3) Å^3,
Z = 1, *T* = 193.00 K,
μ(MoKα) = 0.130 mm^{-1},
Dcalc = 1.302 g/cm^3,
119158 reflections measured (4.224° ≤ 2Θ ≤ 52.744°),
23578 unique (R_{int} = 0.0983, R_{sigma} = 0.0681) which were used in all calculations.
The final $R_1$was 0.0679 (I > 2σ(I)) and wR_2 was 0.1971 (all data).

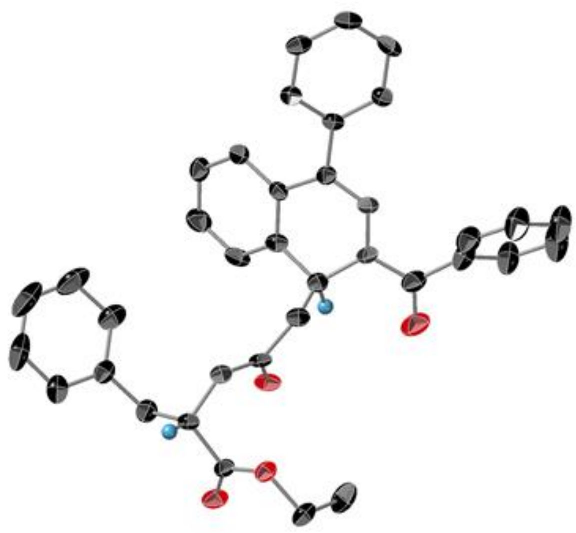

Figure 2. ORTEP plot of **(*S*,*S*)-20** with thermal ellipsoids set at the 50% probability level. Hydrogen atoms are omitted for clarity (except stereogenic centers).

15. NMR spectra of new compounds

^{1}H NMR ($CDCl_3$, 300 MHz) of **1j**

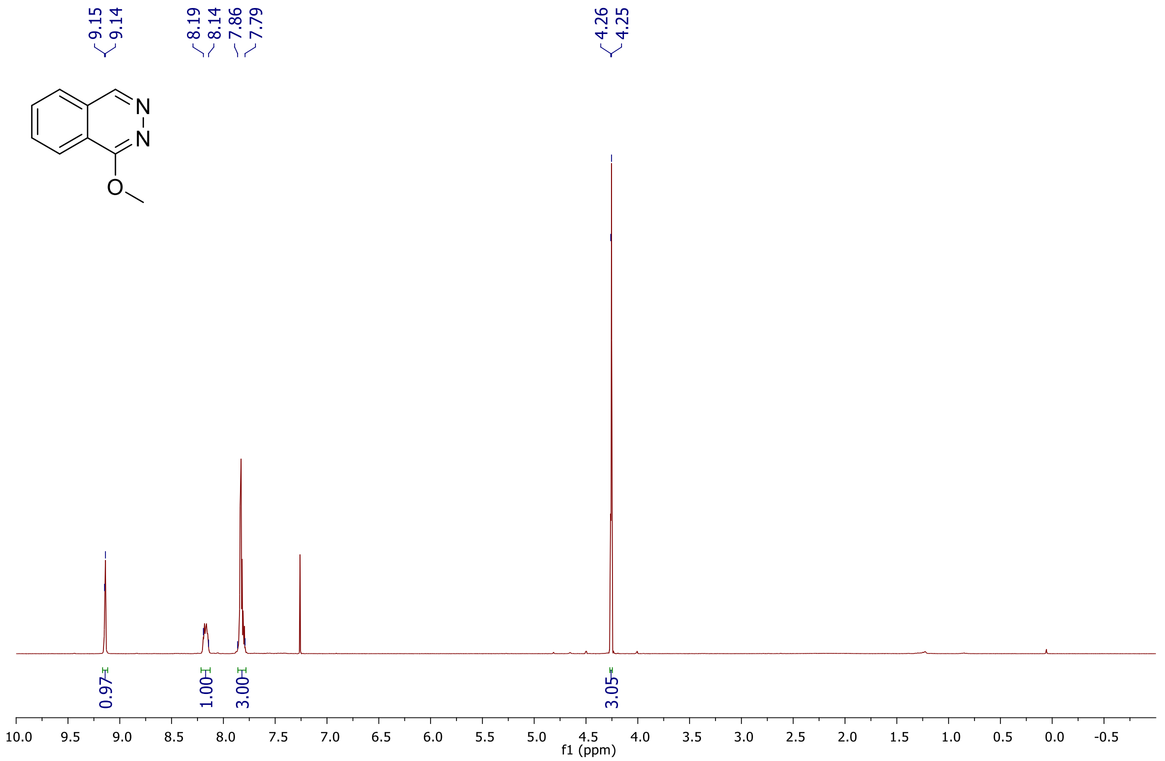

^{13}C NMR ($CDCl_3$, 75.5 MHz) of **1j**

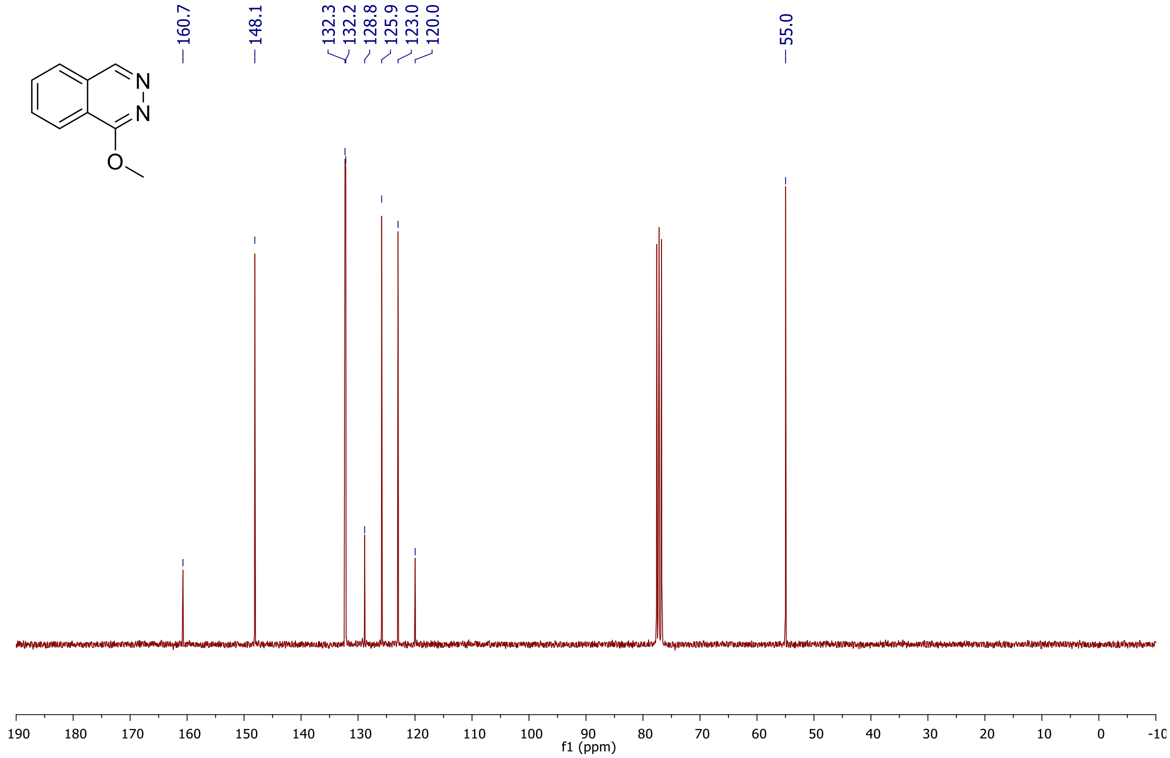

^{1}H NMR ($CDCl_3$, 300 MHz) of **1k**

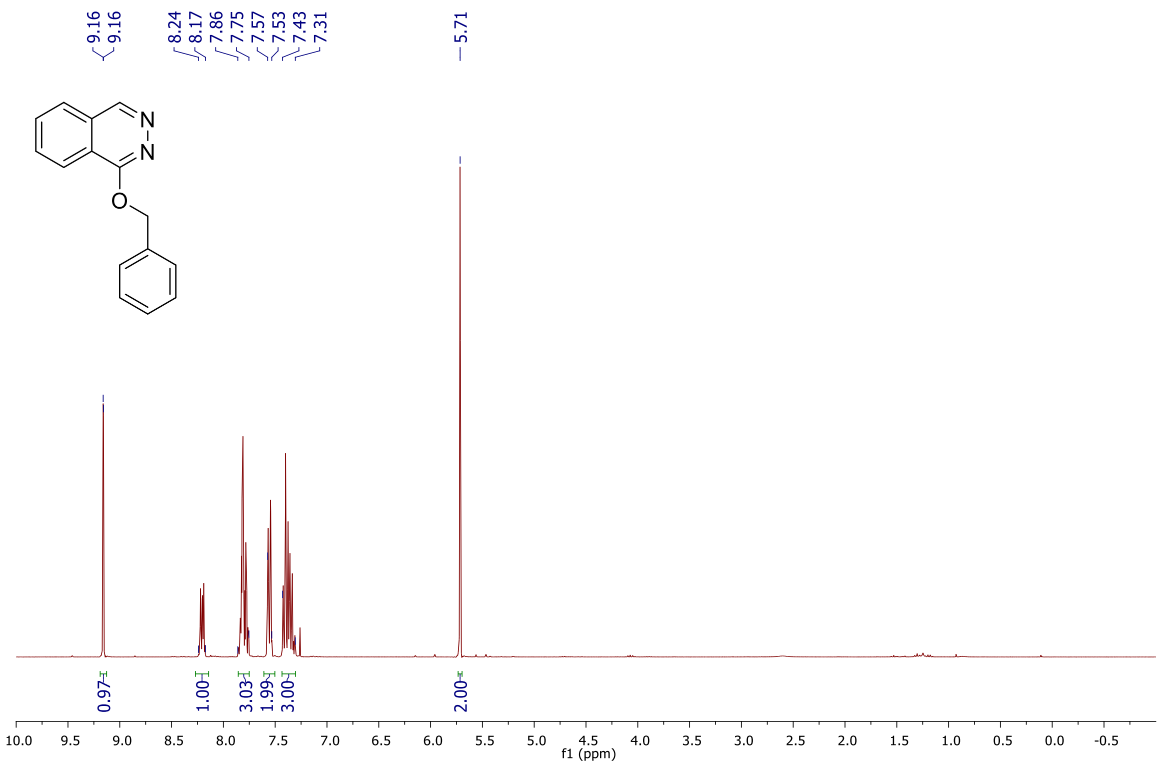

^{13}C NMR ($CDCl_3$, 75.5 MHz) of **1k**

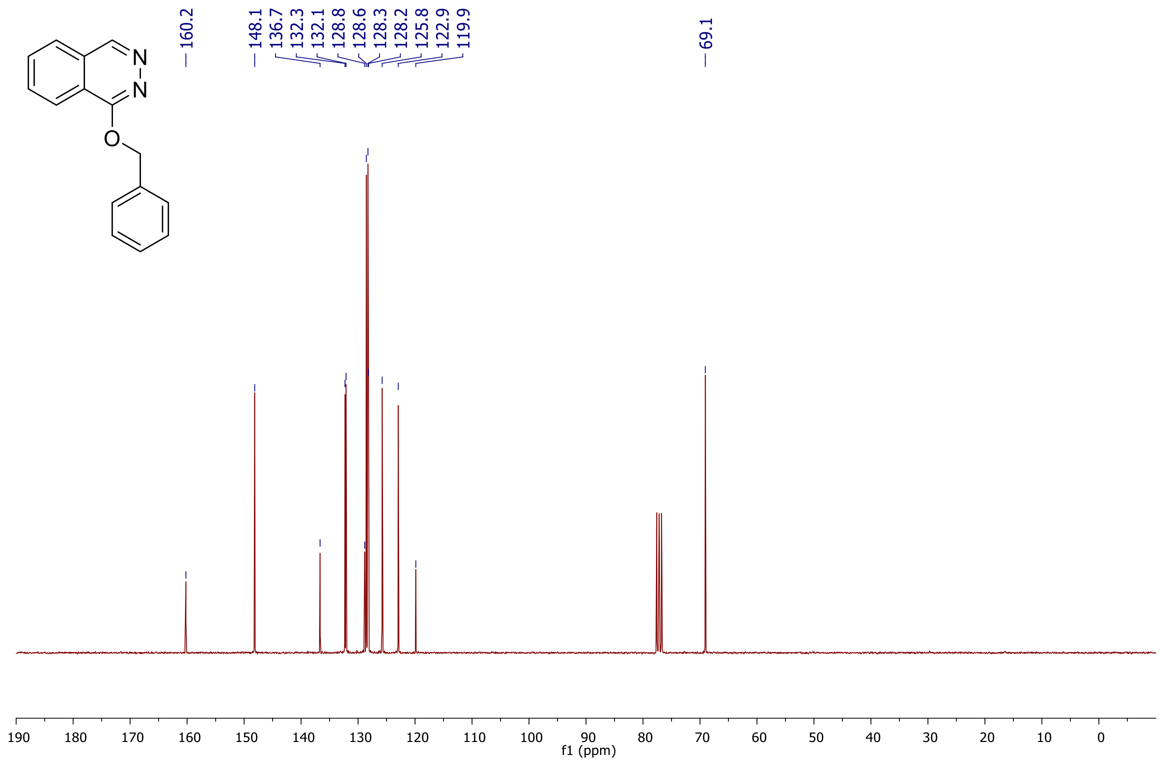

^{1}**H NMR** (CDCl$_3$, 300 MHz) of **1m**

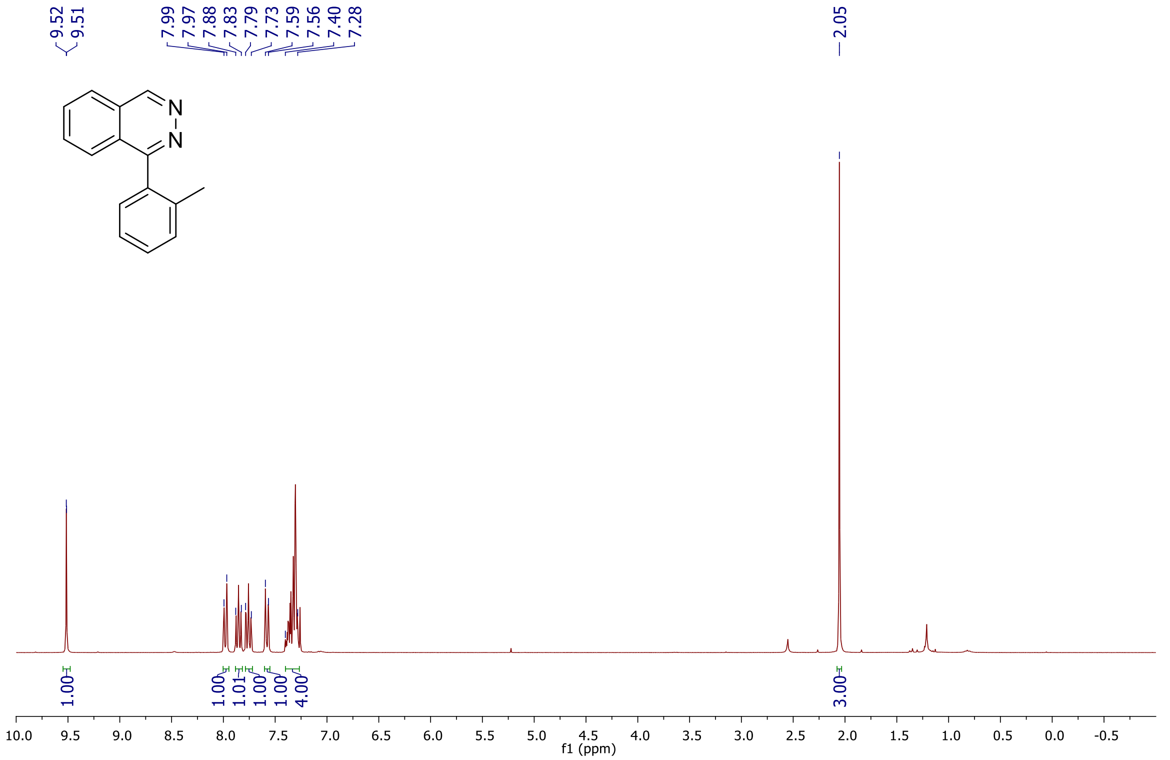

^{13}**C NMR** (CDCl$_3$, 75.5 MHz) of **1m**

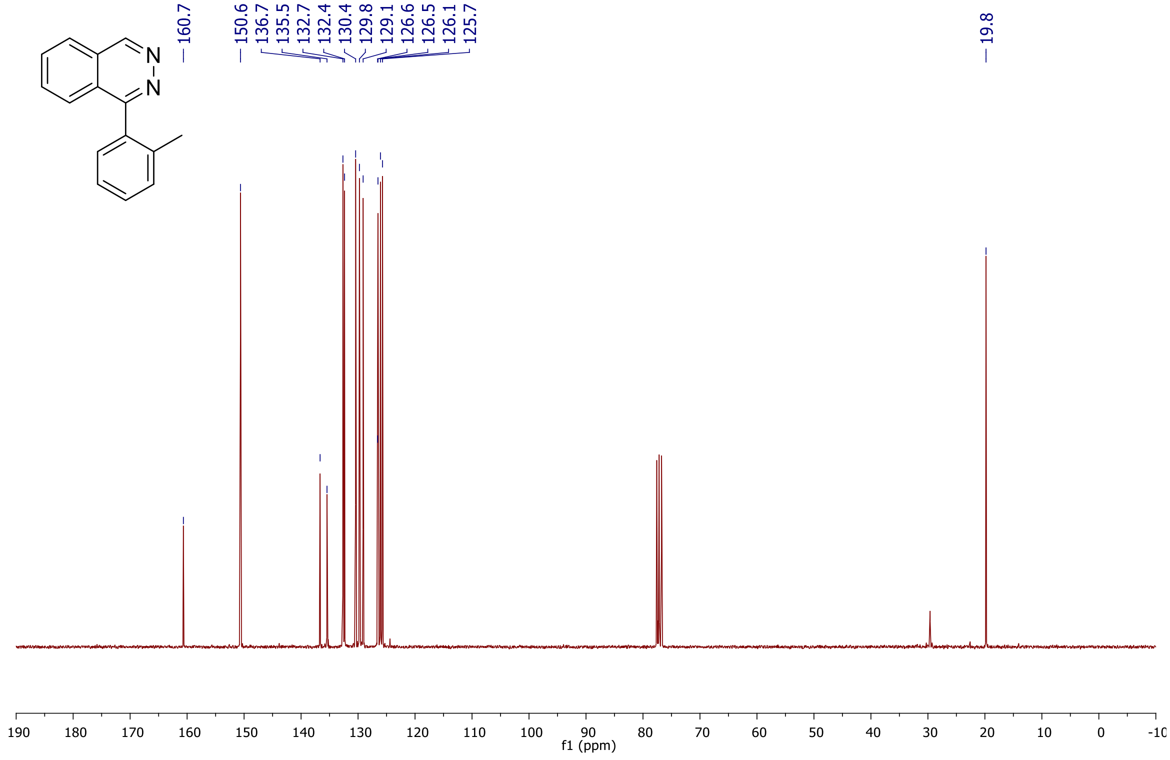

^{1}H NMR ($CDCl_3$, 300 MHz) of **1n**

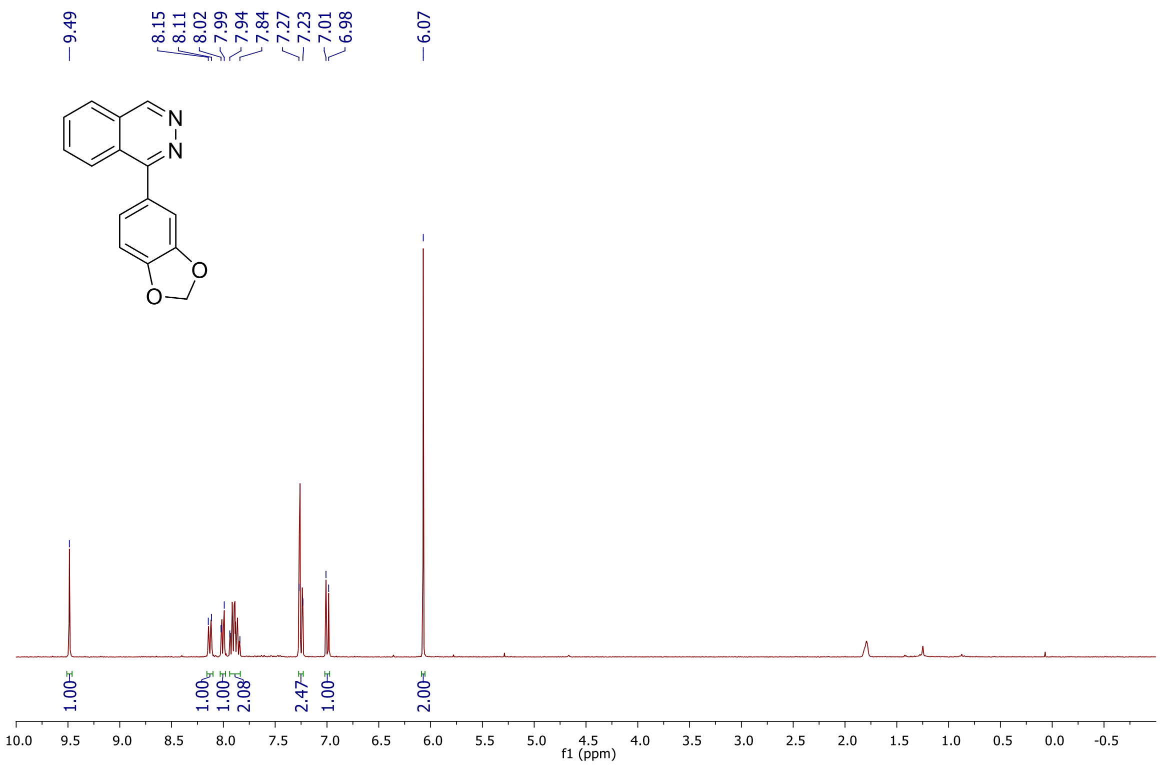

^{13}C NMR ($CDCl_3$, 75.5 MHz) of **1n**

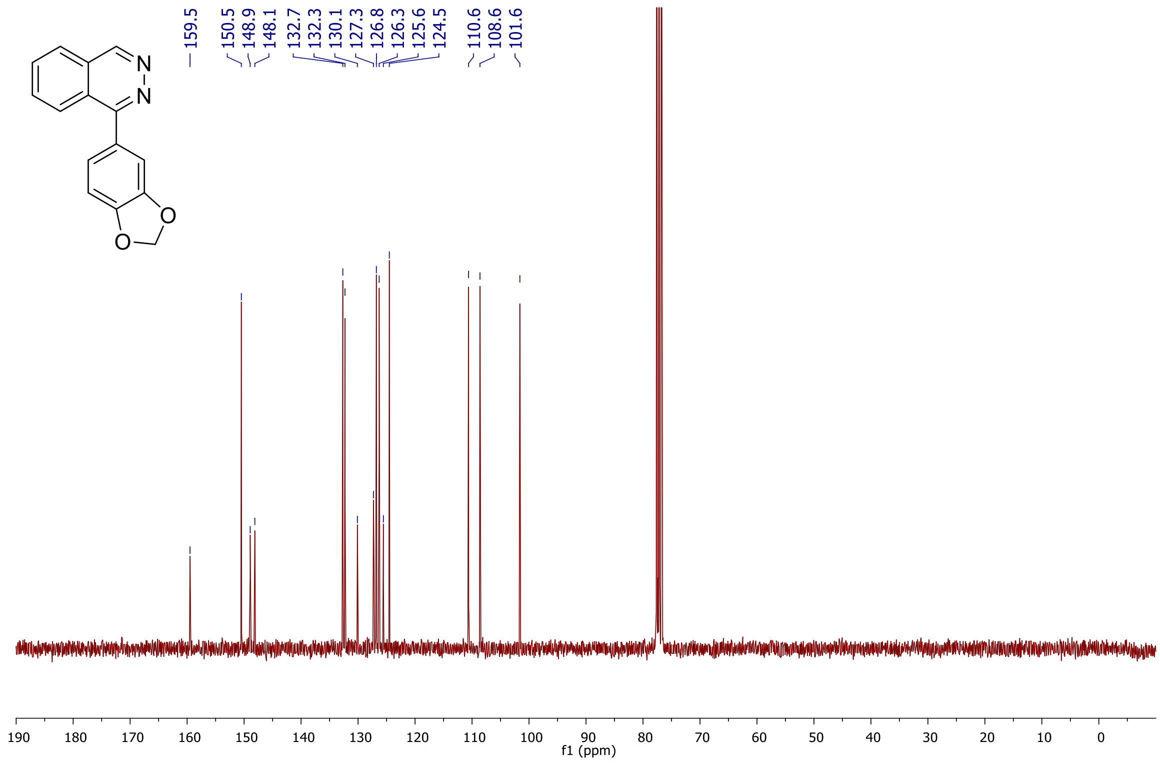

^{1}**H NMR** ($CDCl_3$, 300 MHz) of **1q**

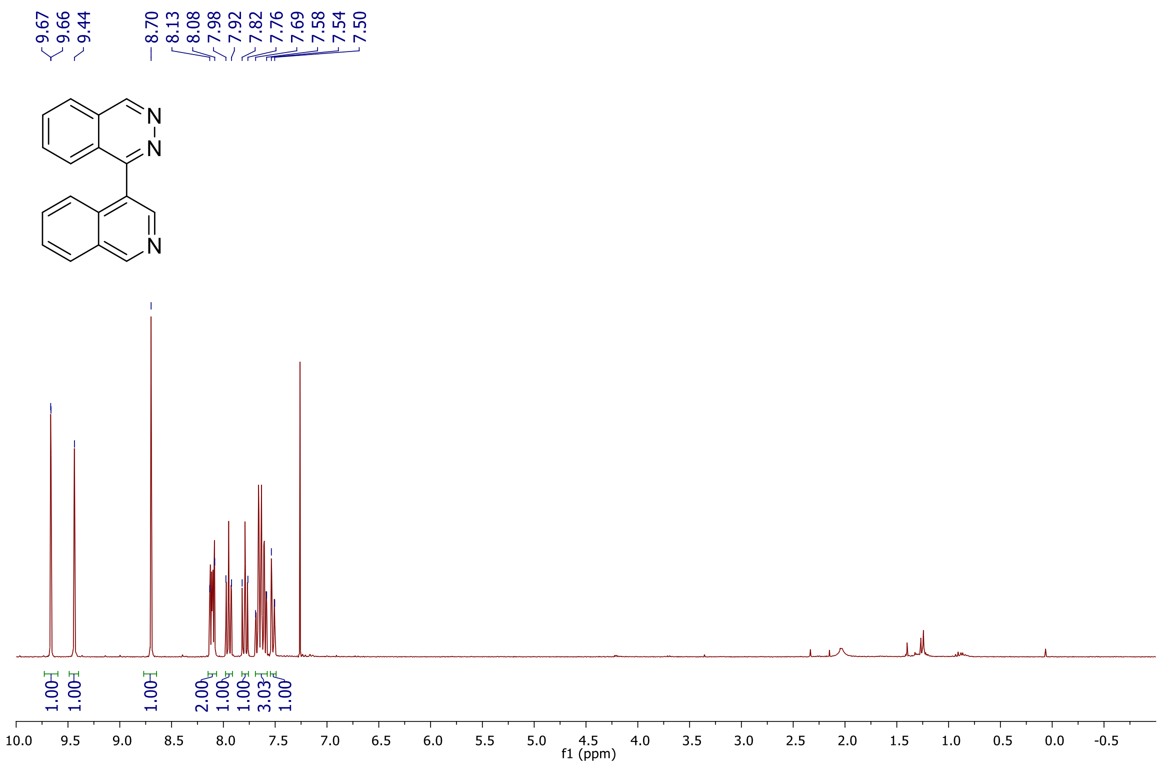

^{13}**C NMR** ($CDCl_3$, 126 MHz) of **1q**

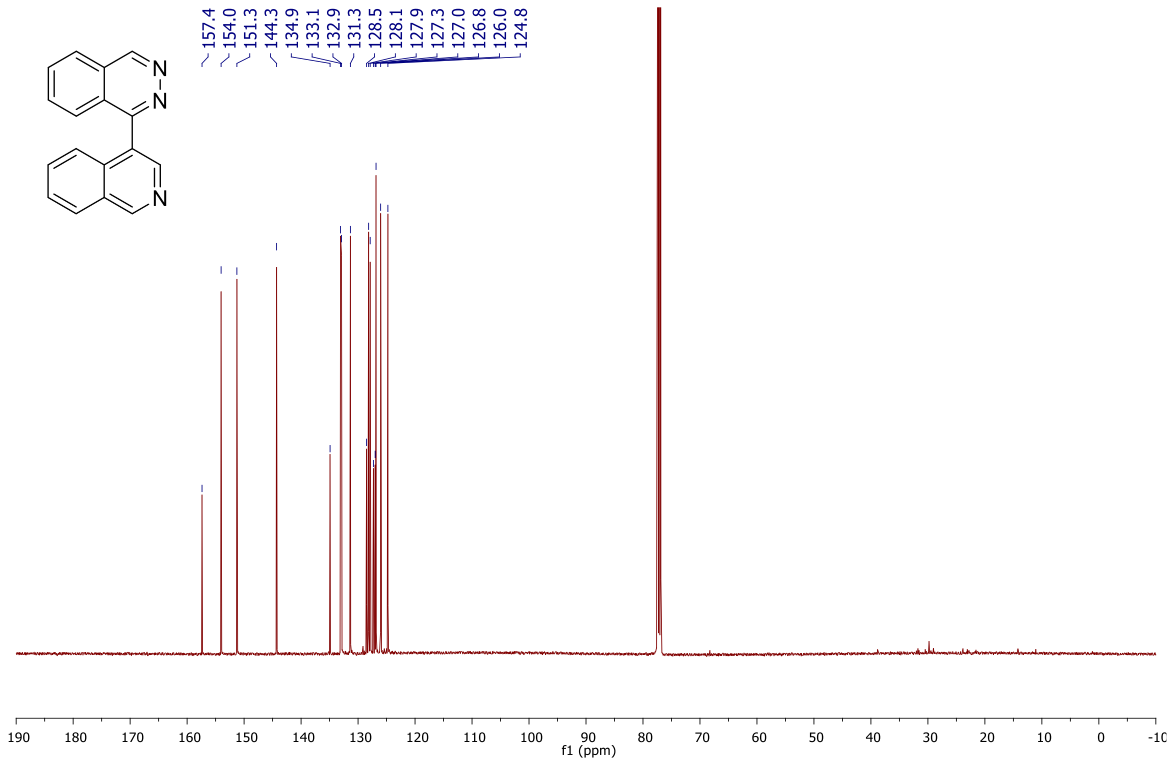

^{1}H NMR ($CDCl_3$, 300 MHz) of **1o**

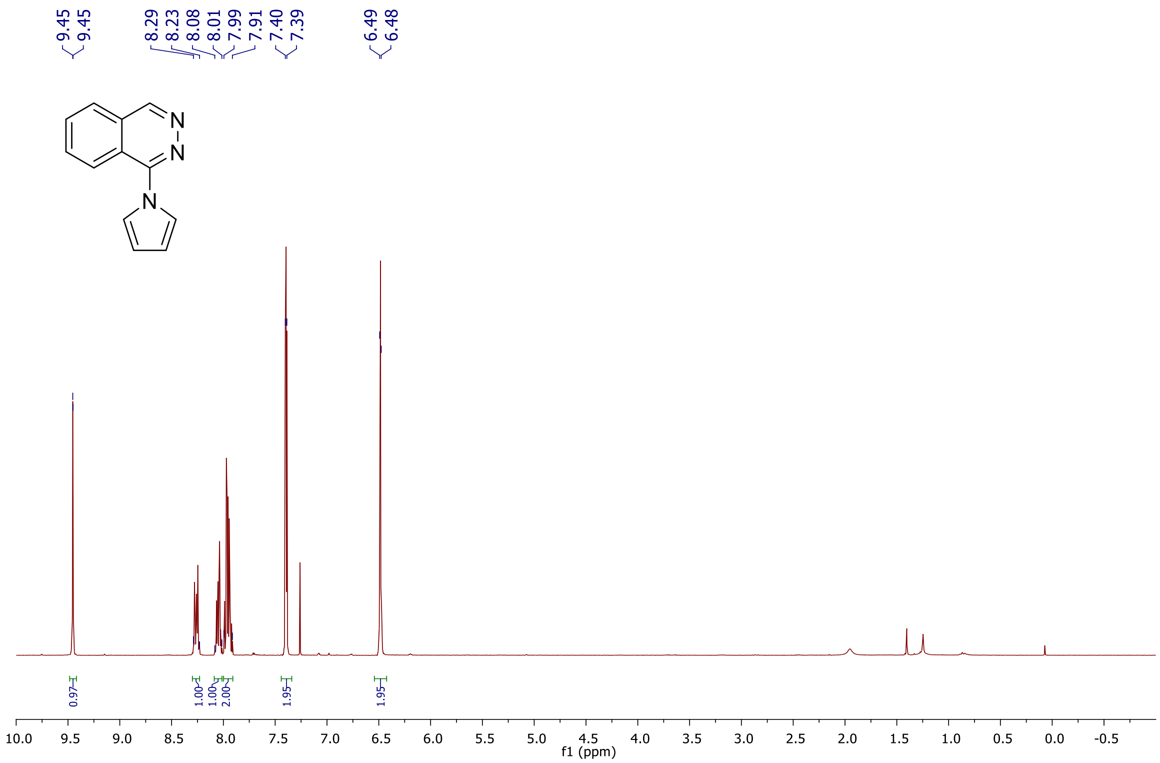

^{13}C NMR ($CDCl_3$, 75.5 MHz) of **1o**

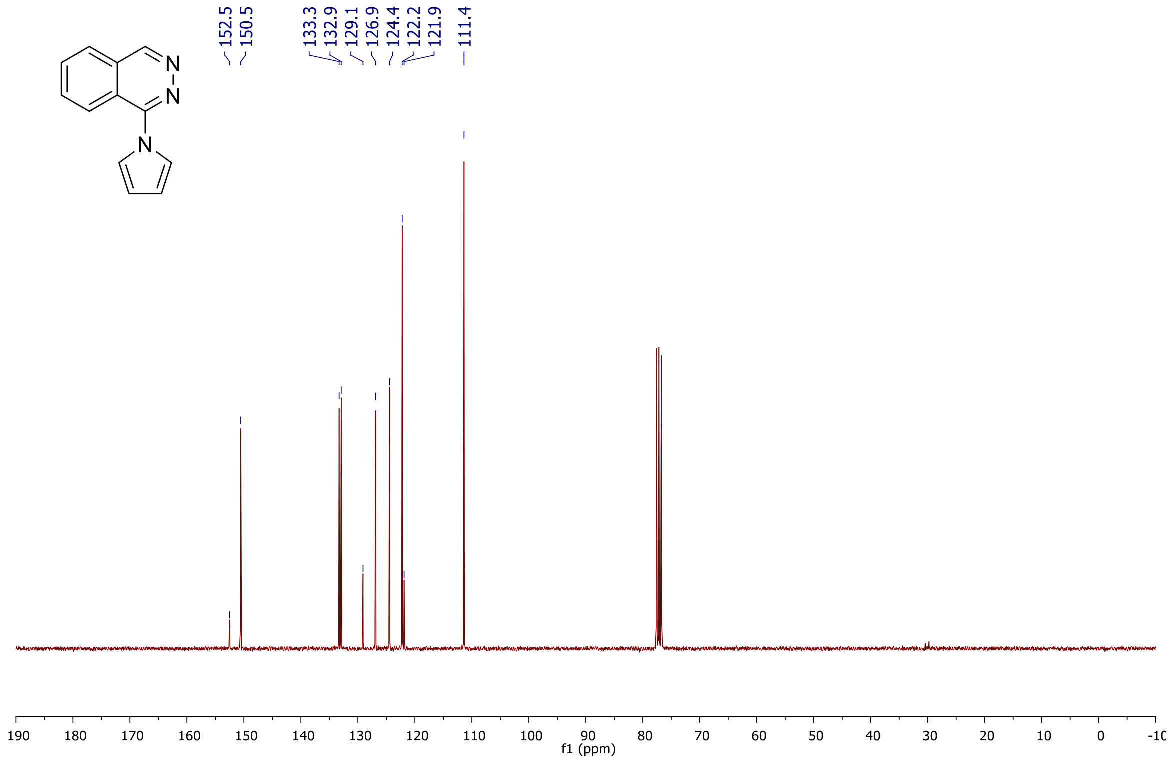

^{1}H NMR (CDCl$_3$, 300 MHz) of **VII**

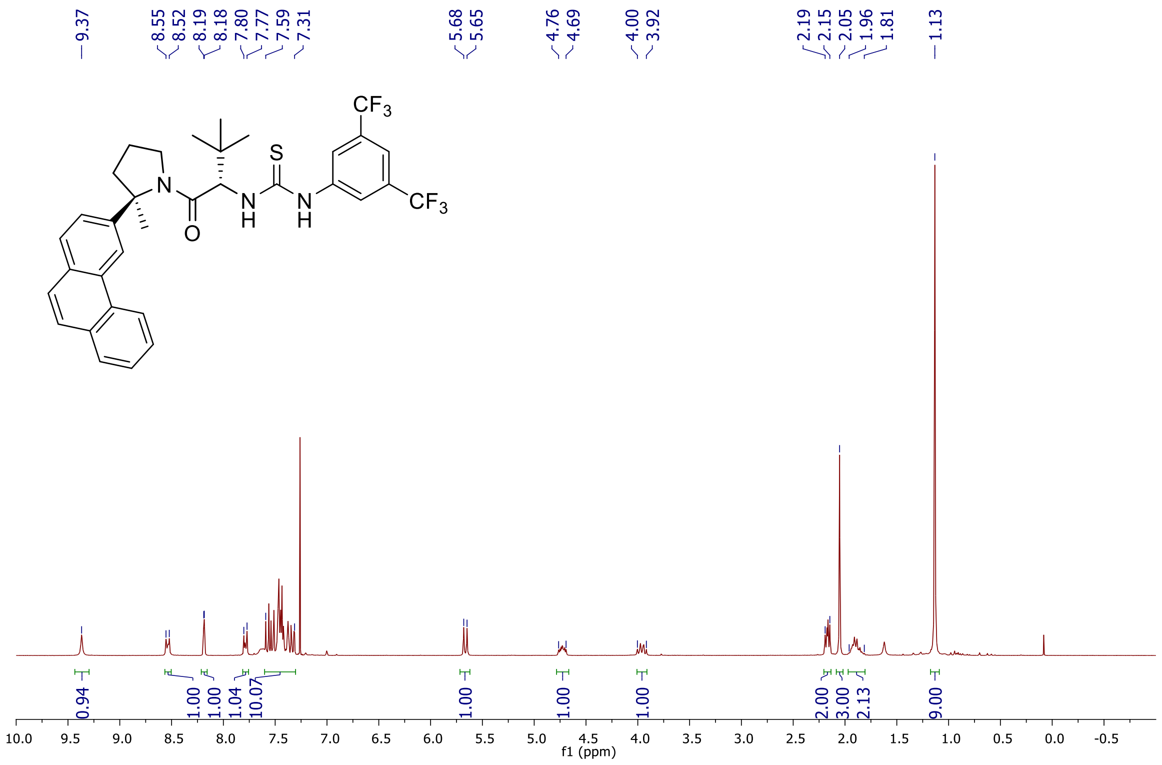

^{13}C NMR (CDCl$_3$, 126 MHz) of **VII**

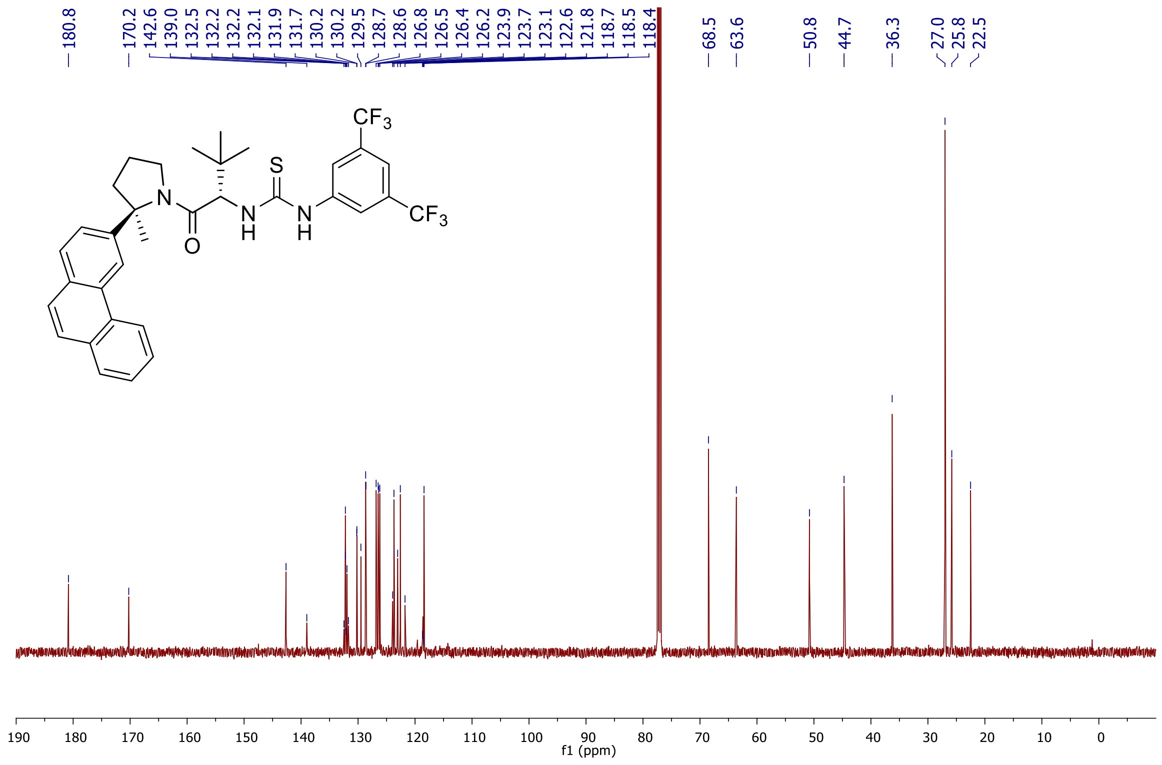

^{1}H NMR ($CDCl_3$, 300 MHz) of ***(S)*-3aa**

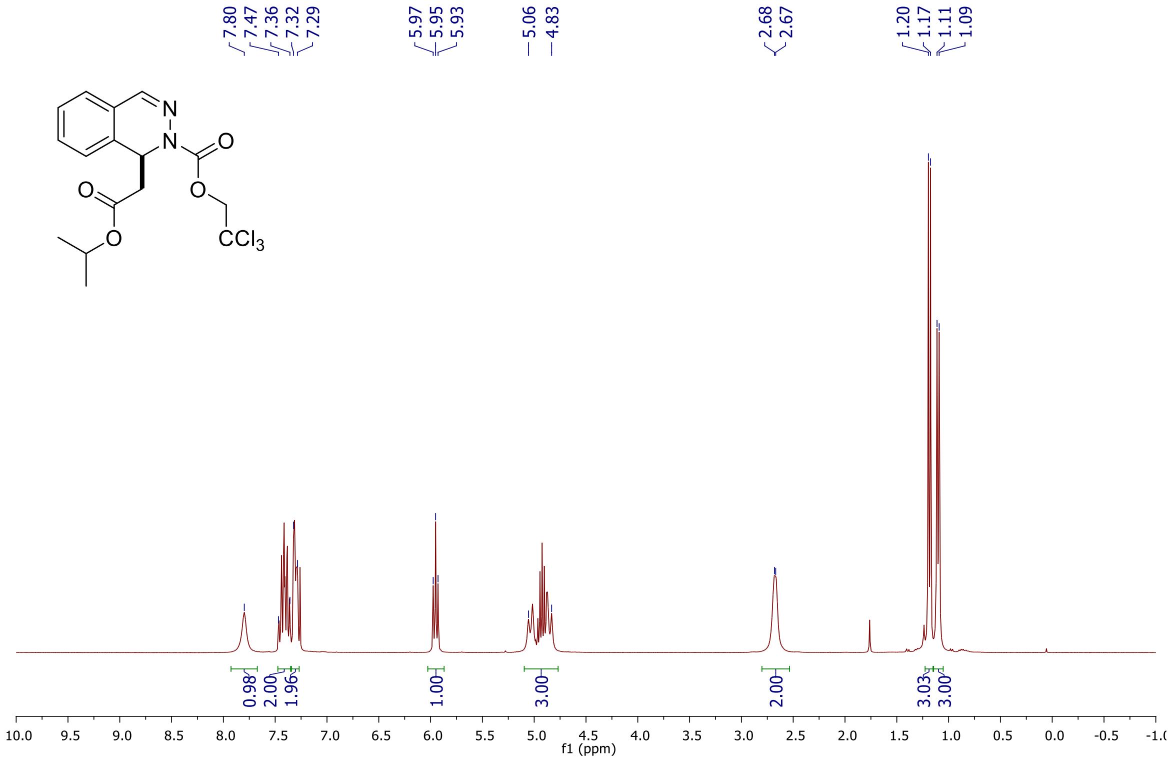

^{13}C NMR ($CDCl_3$, 75.5 MHz) of ***(S)*-3aa**

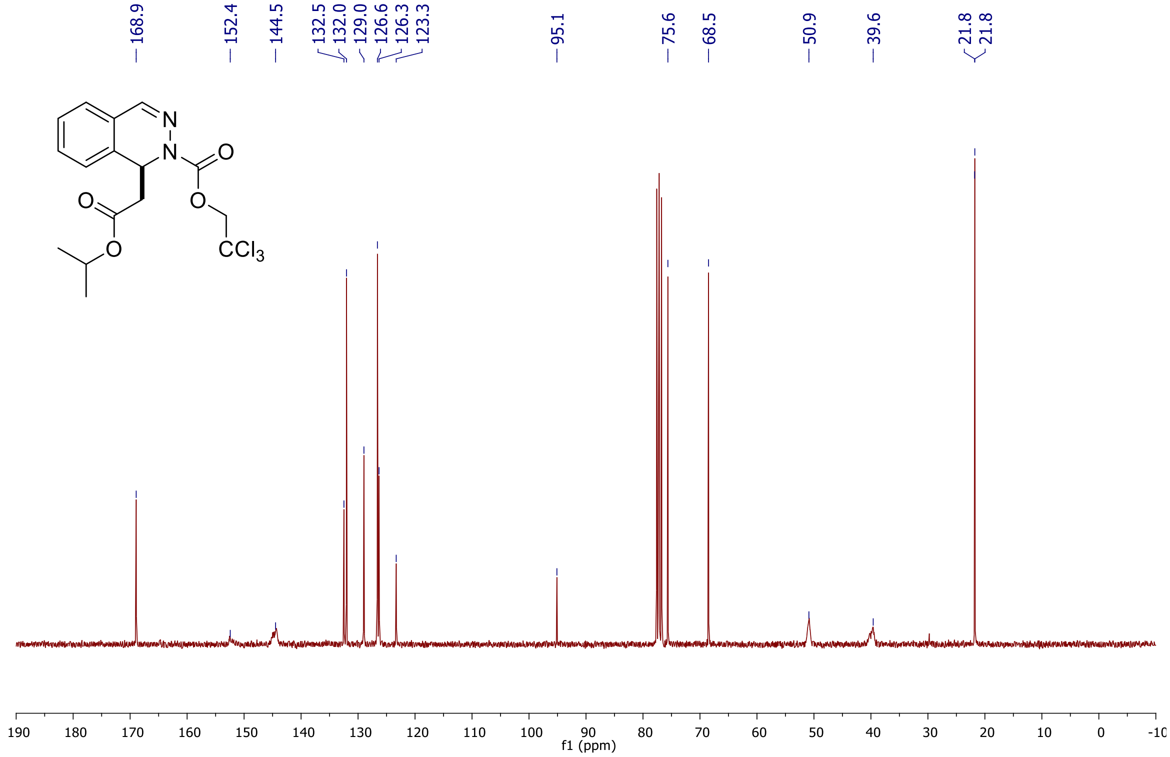

^{1}**H NMR** ($CDCl_3$, 300 MHz) of **(*S*)-4aa**

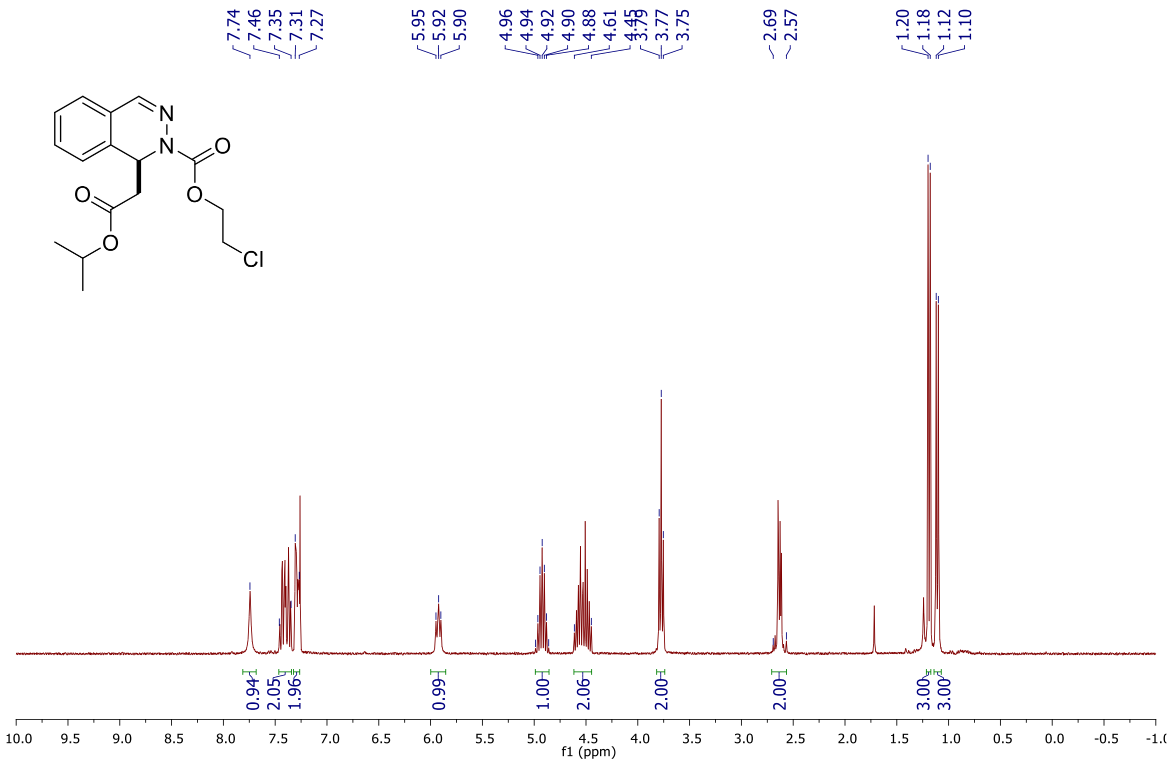

^{13}**C NMR** ($CDCl_3$, 75.5 MHz) of **(*S*)-4aa**

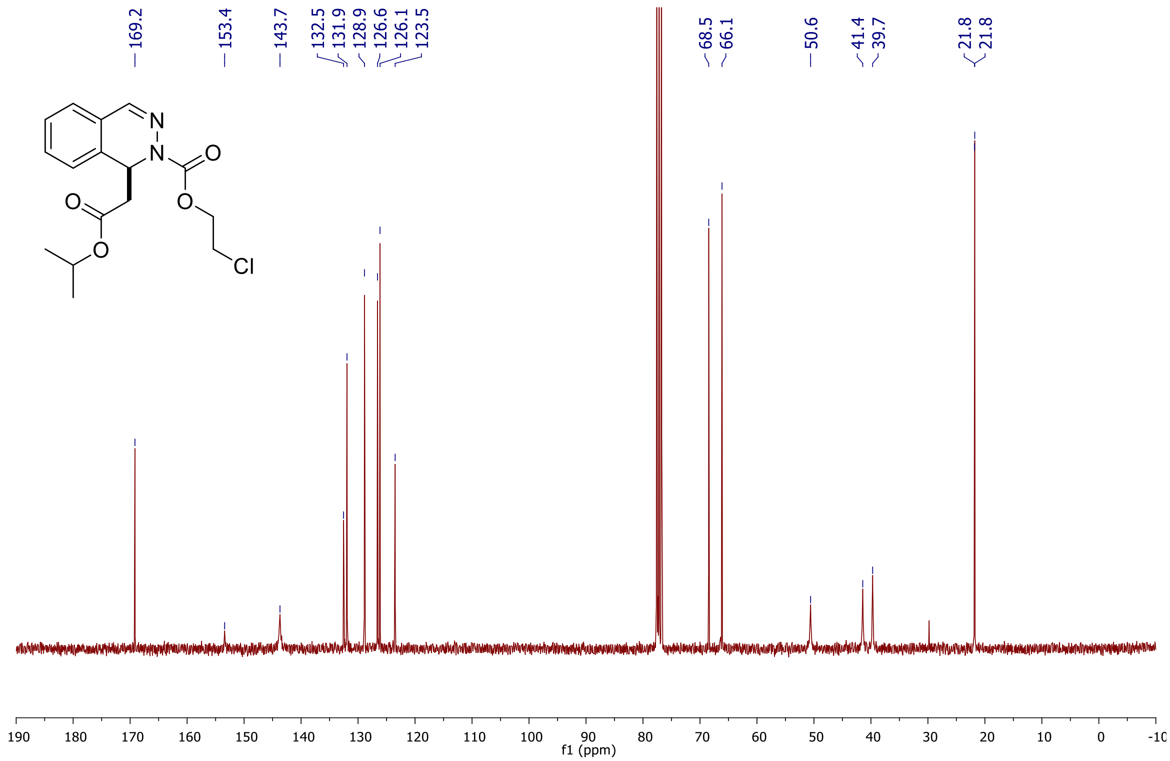

^{1}H NMR (CDCl$_3$, 300 MHz) of **(*S*)-6aa**

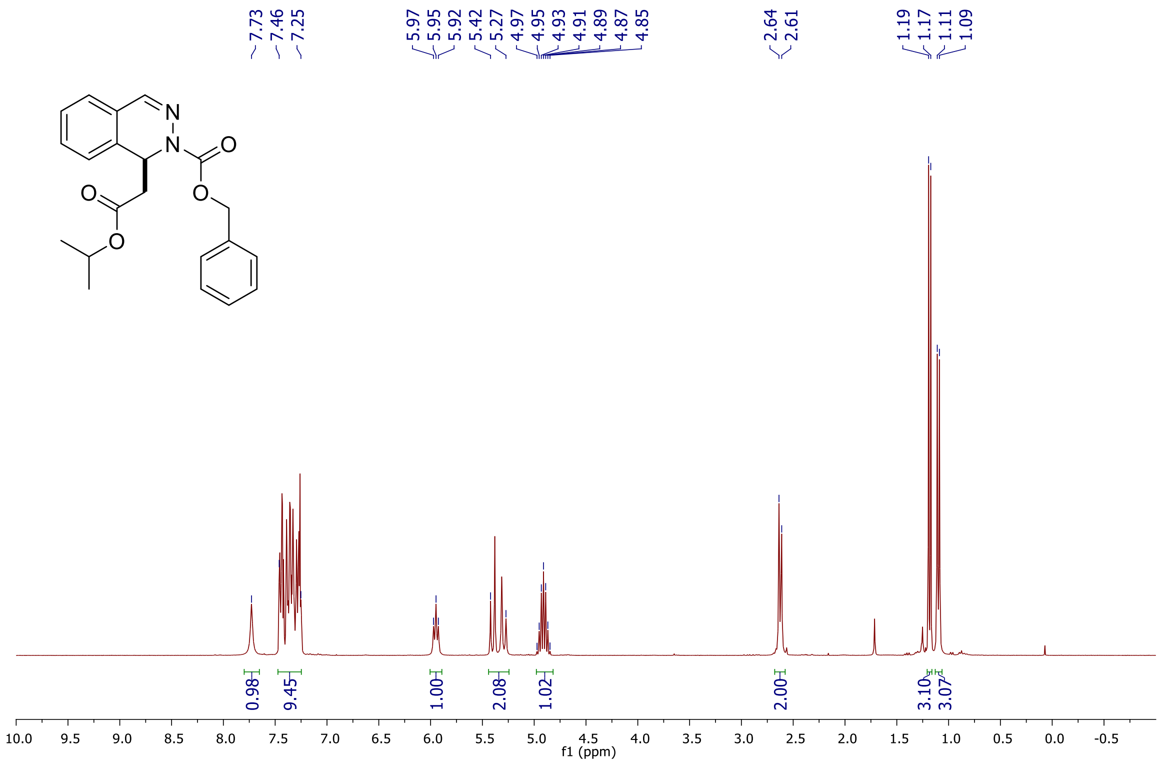

^{13}C NMR (CDCl$_3$, 75.5 MHz) of **(*S*)-6aa**

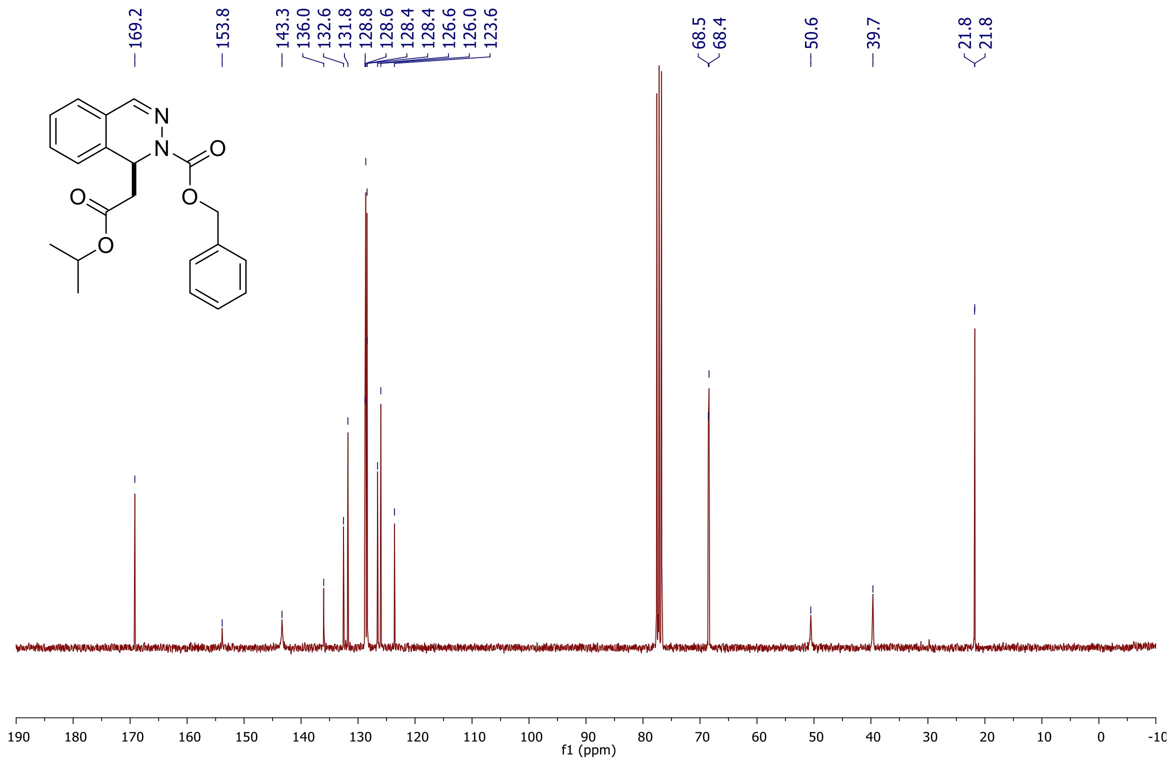

^{1}H NMR (CDCl$_3$, 500 MHz) of **(*S*)-7aa**

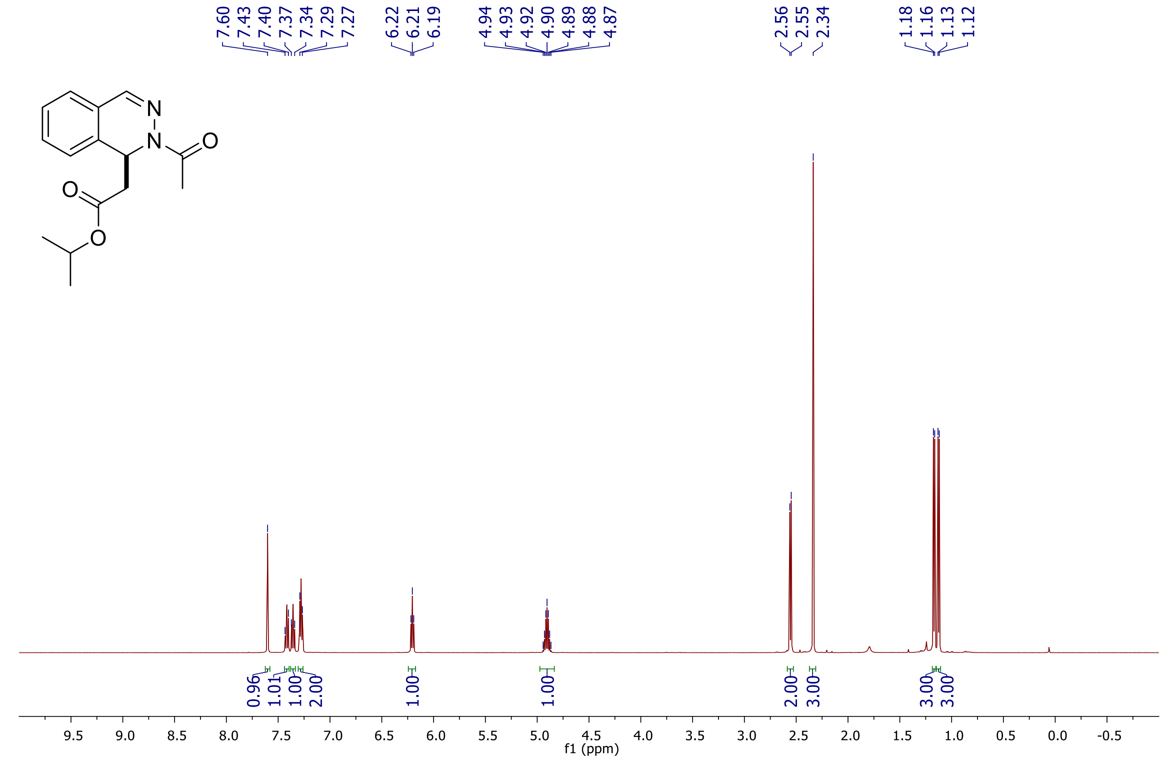

^{13}C NMR (CDCl$_3$, 126 MHz) of **(*S*)-7aa**

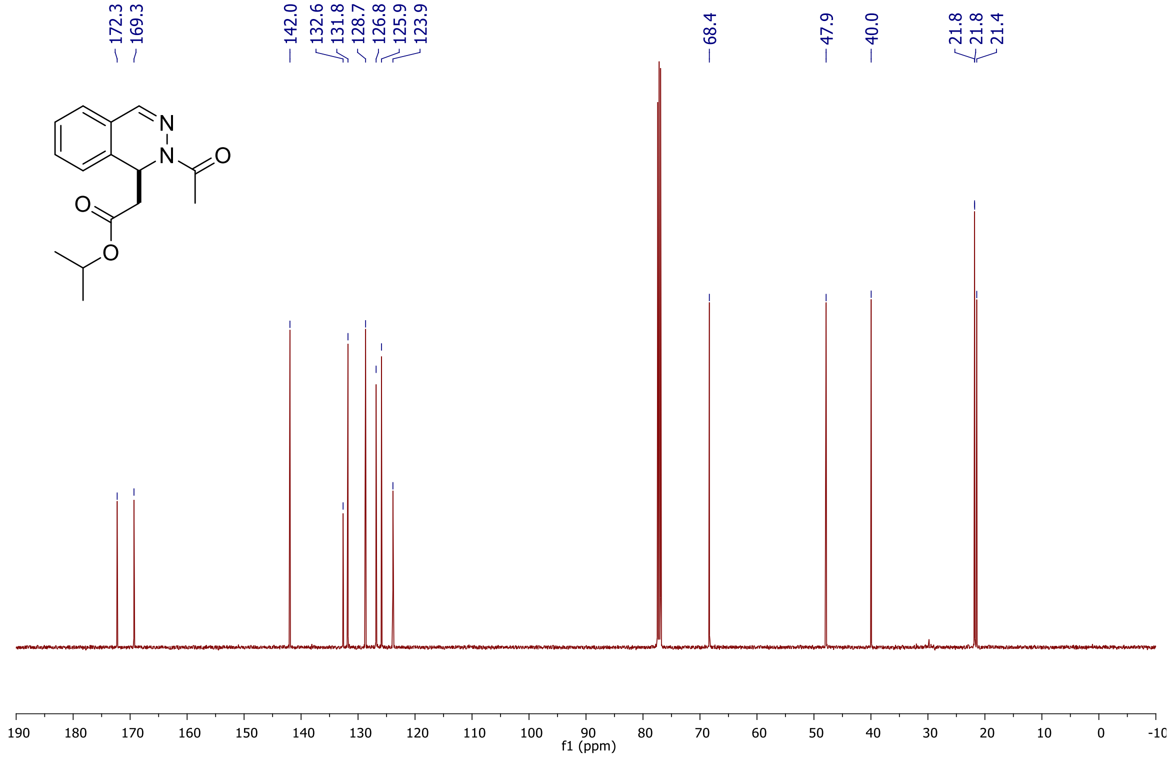

^{1}H NMR (CDCl$_3$, 500 MHz) of **(*S*)-9aa**

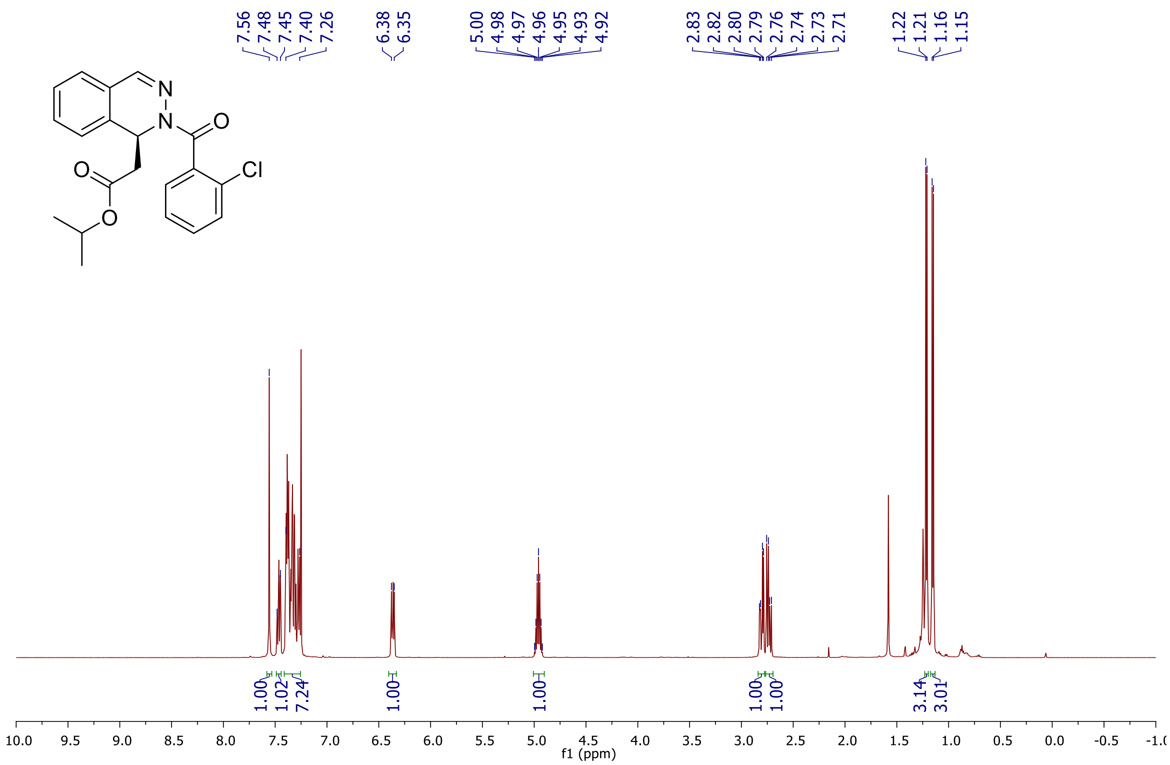

^{13}C NMR (CDCl$_3$, 126 MHz) of **(*S*)-9aa**

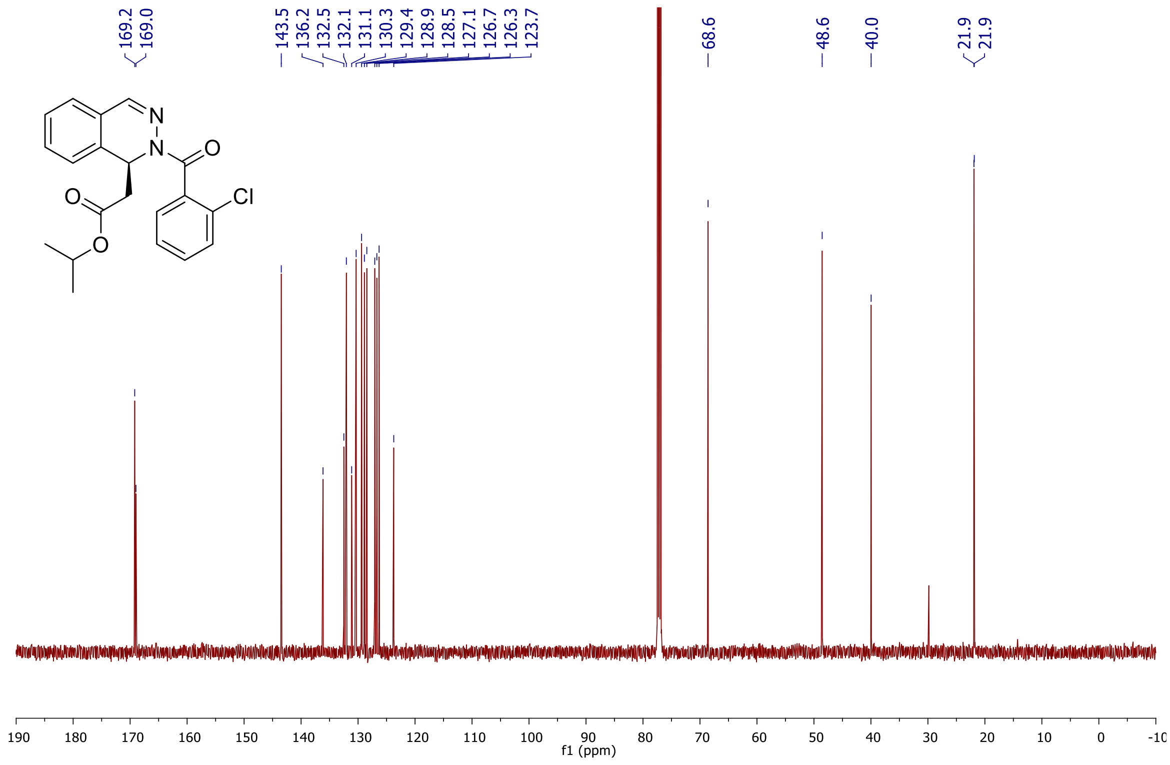

¹H NMR (CDCl$_3$, 500 MHz) of **(*S*)-10aa**

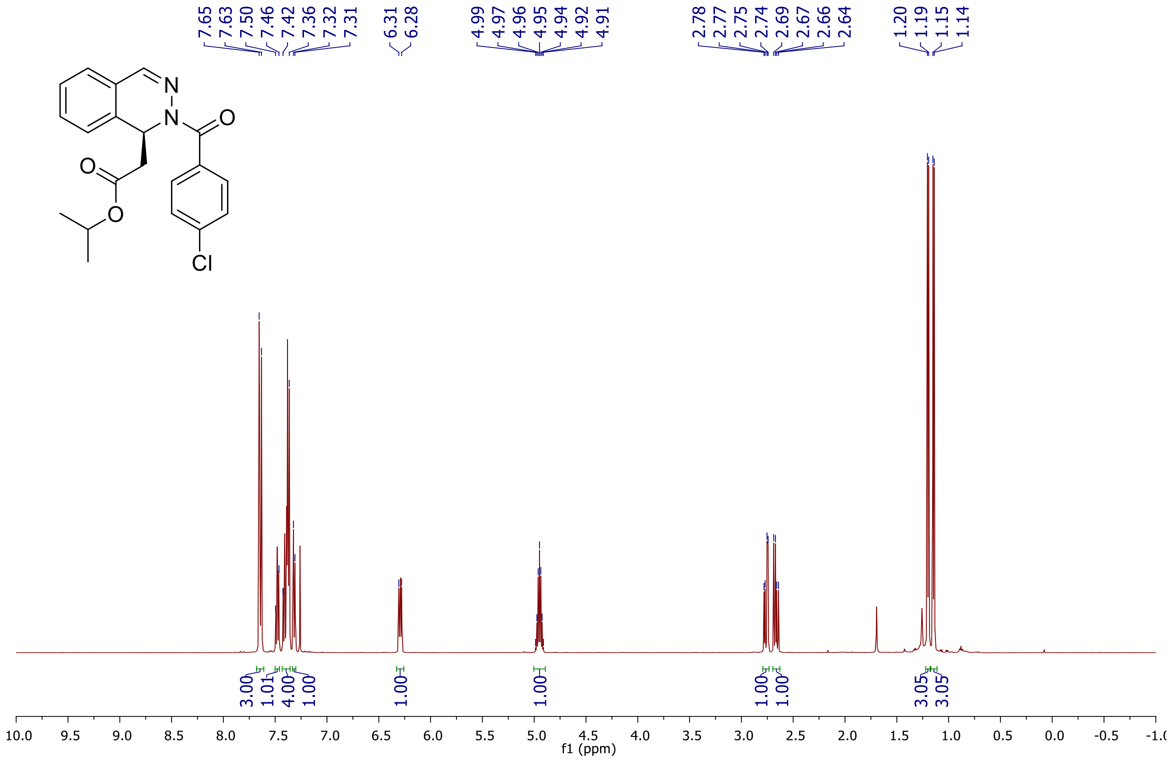

¹³C NMR (CDCl$_3$, 126 MHz) of **(*S*)-10aa**

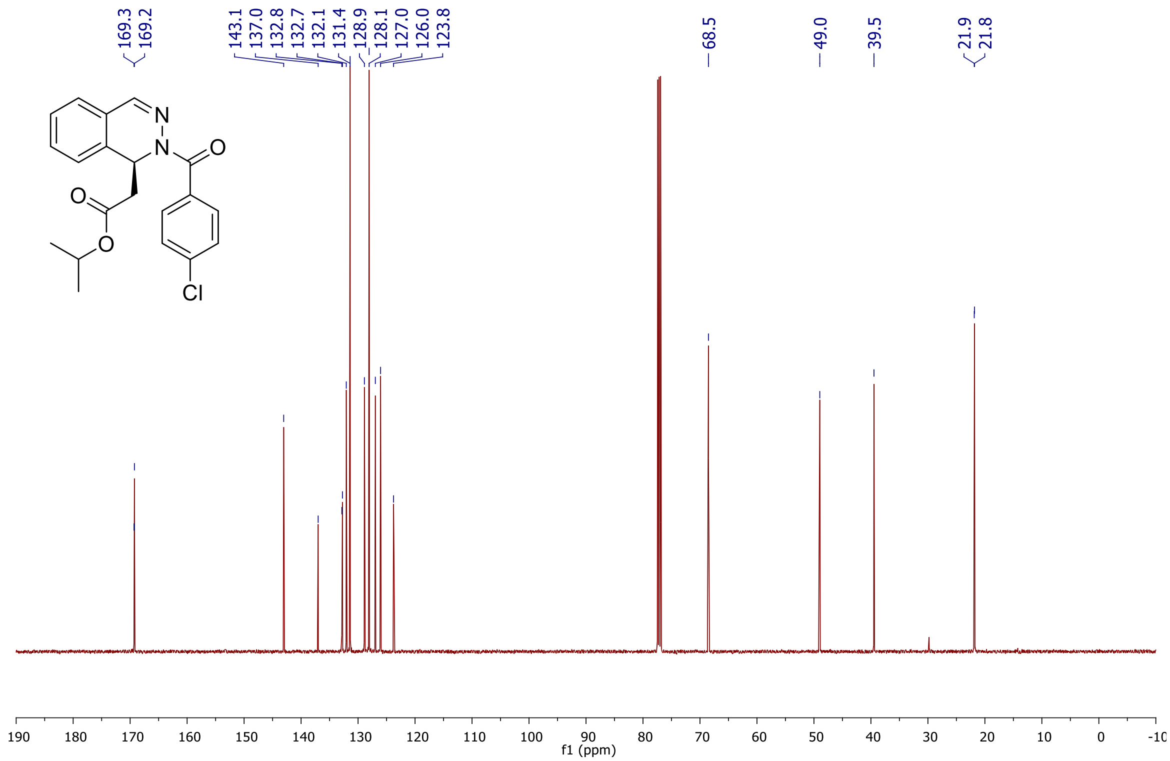

^{1}H NMR (CDCl$_3$, 500 MHz) of **(*S*)-11aa**

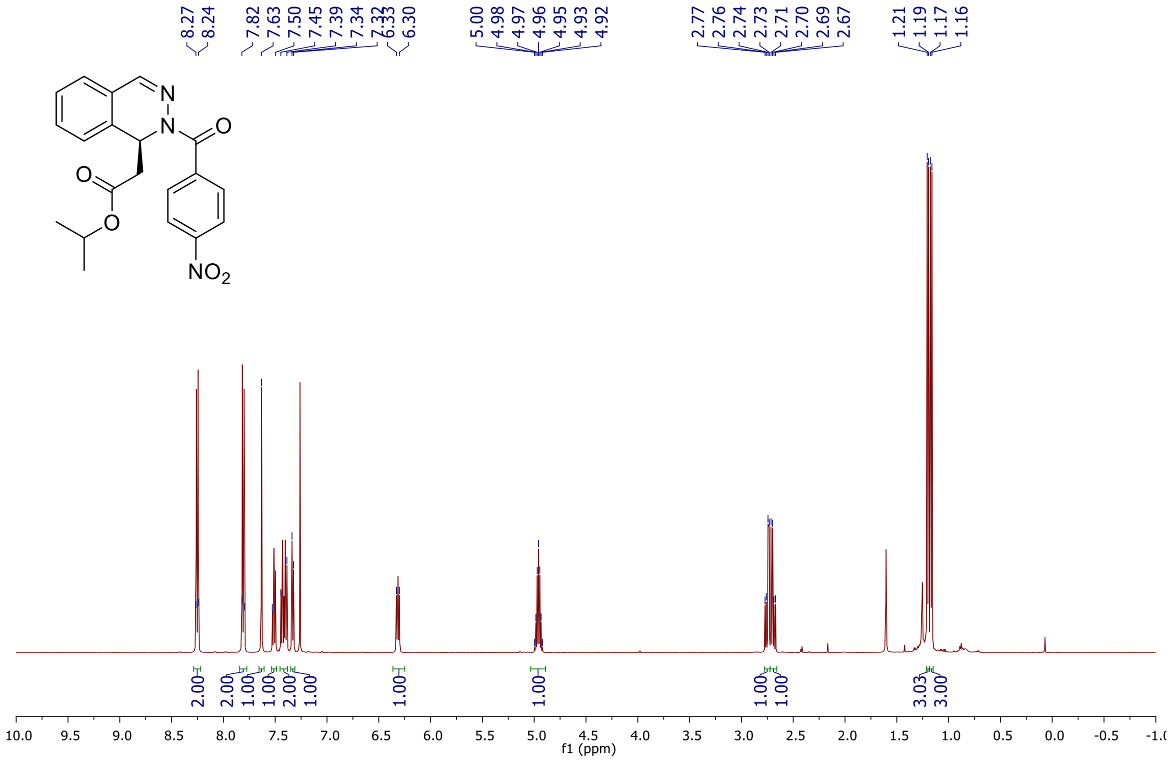

^{13}C NMR (CDCl$_3$, 126 MHz) of **(*S*)-11aa**

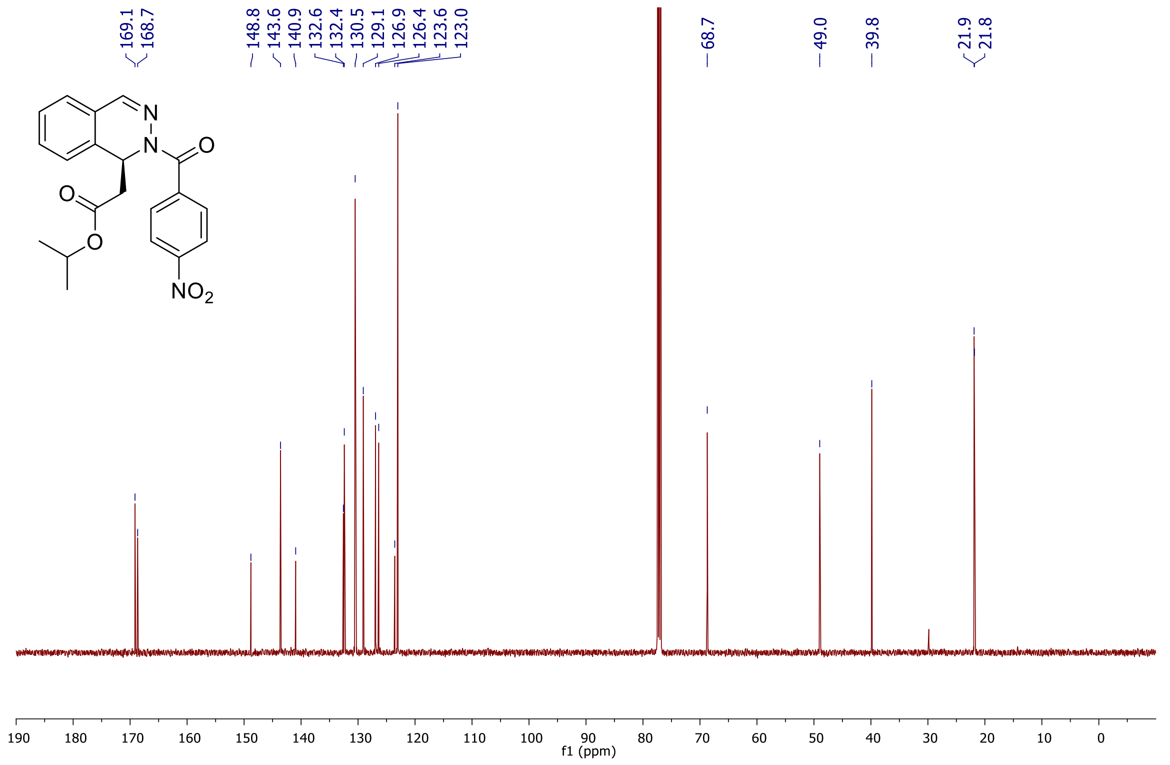

^{1}H NMR ($CDCl_3$, 500 MHz) of **(*S*)-12aa**

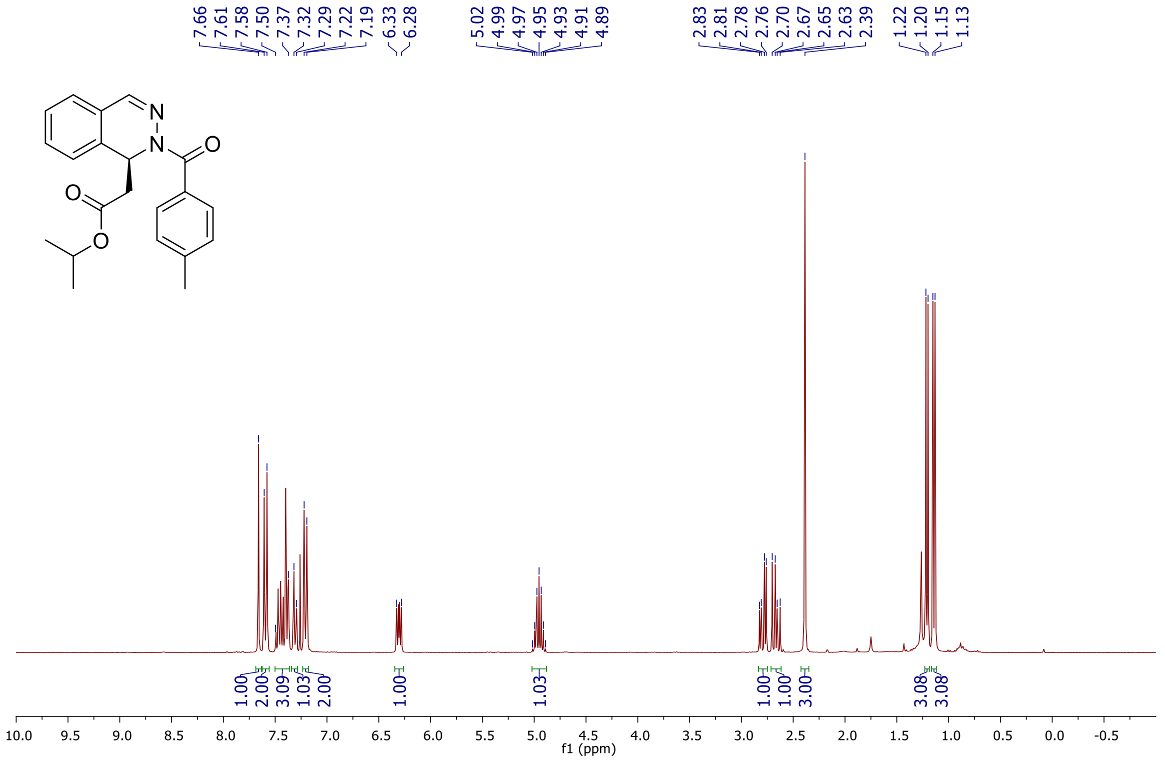

^{13}C NMR ($CDCl_3$, 126 MHz) of **(*S*)-12aa**

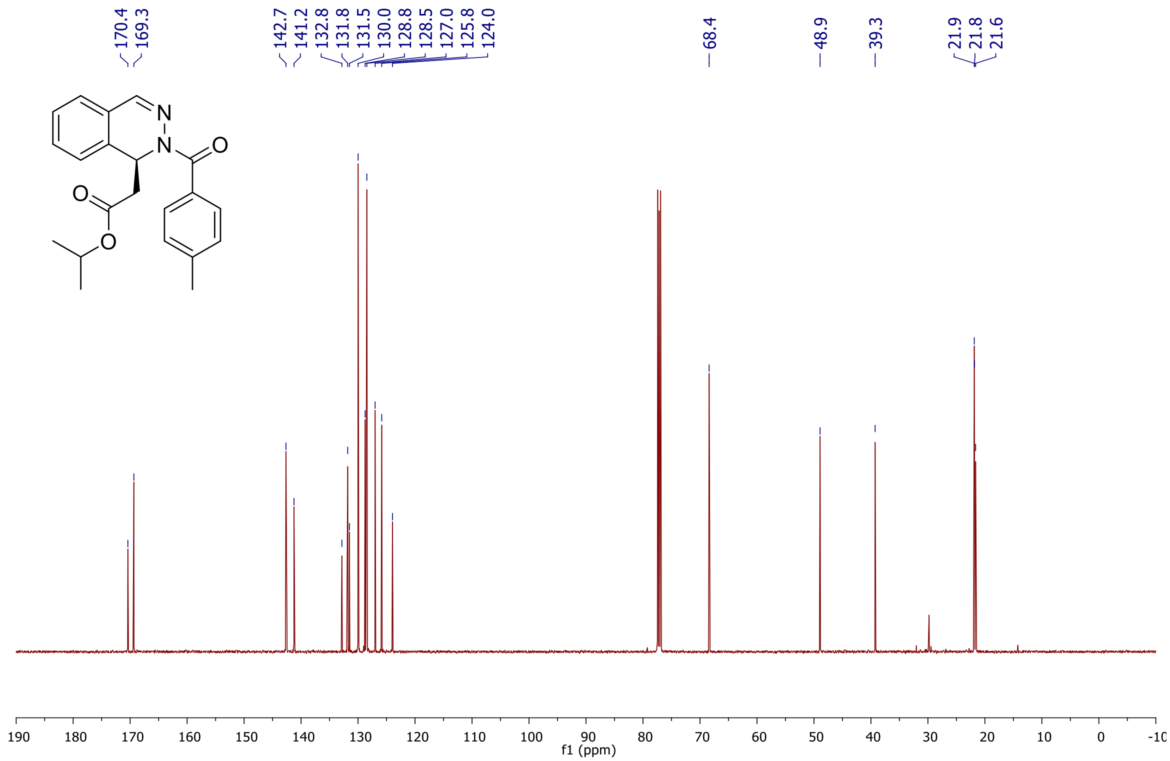

^{1}H NMR ($CDCl_3$, 300 MHz) of **(*S*)-8aa**

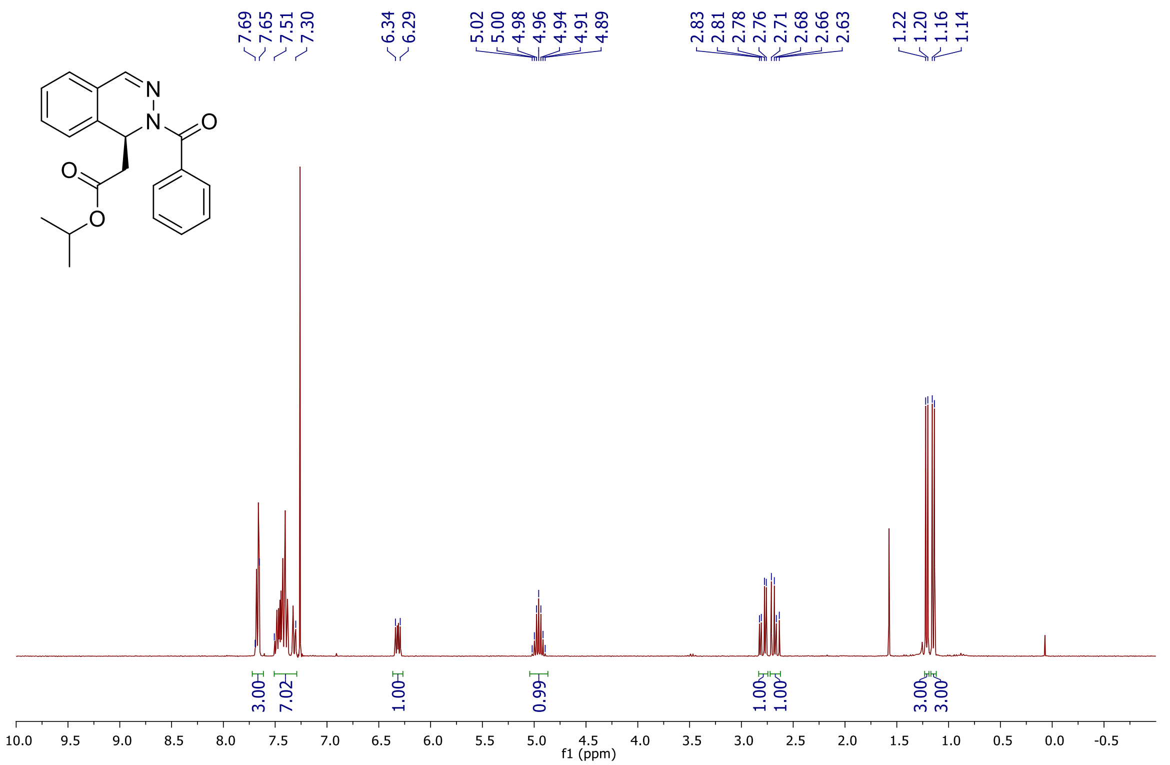

^{13}C NMR ($CDCl_3$, 75.5 MHz) of **(*S*)-8aa**

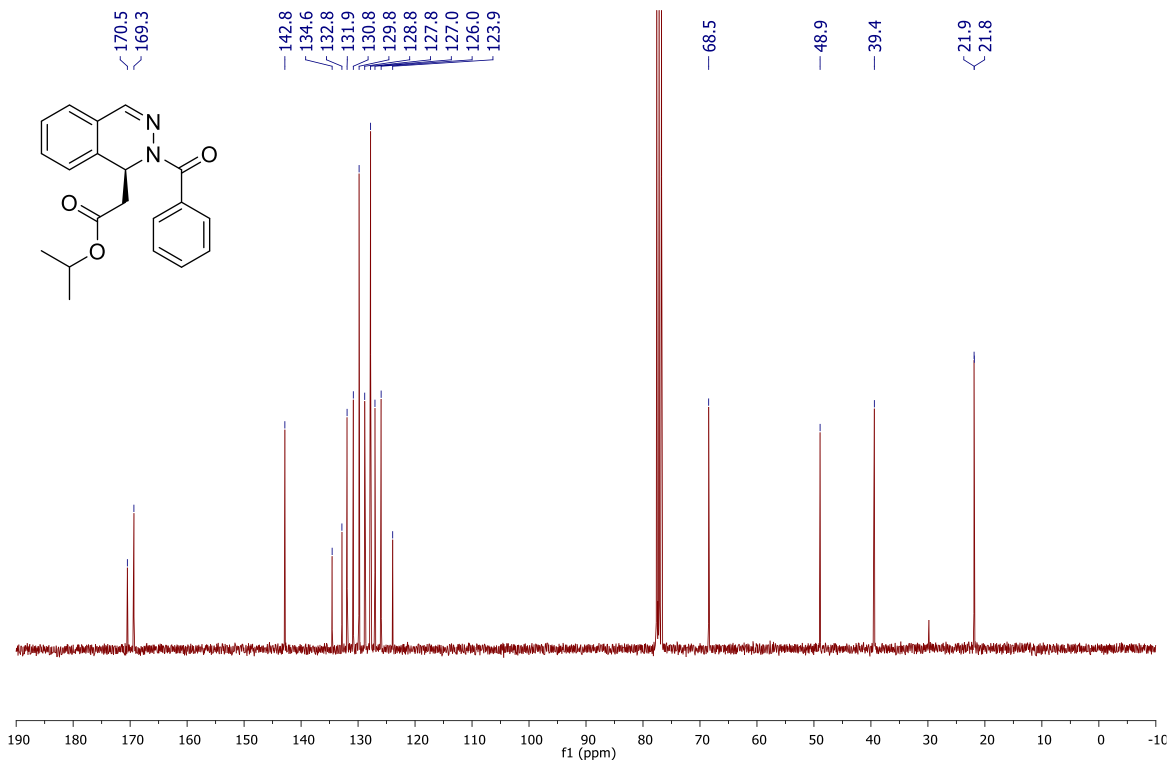

^{1}**H NMR** (CDCl$_3$, 300 MHz) of **(*S*)-8ab**

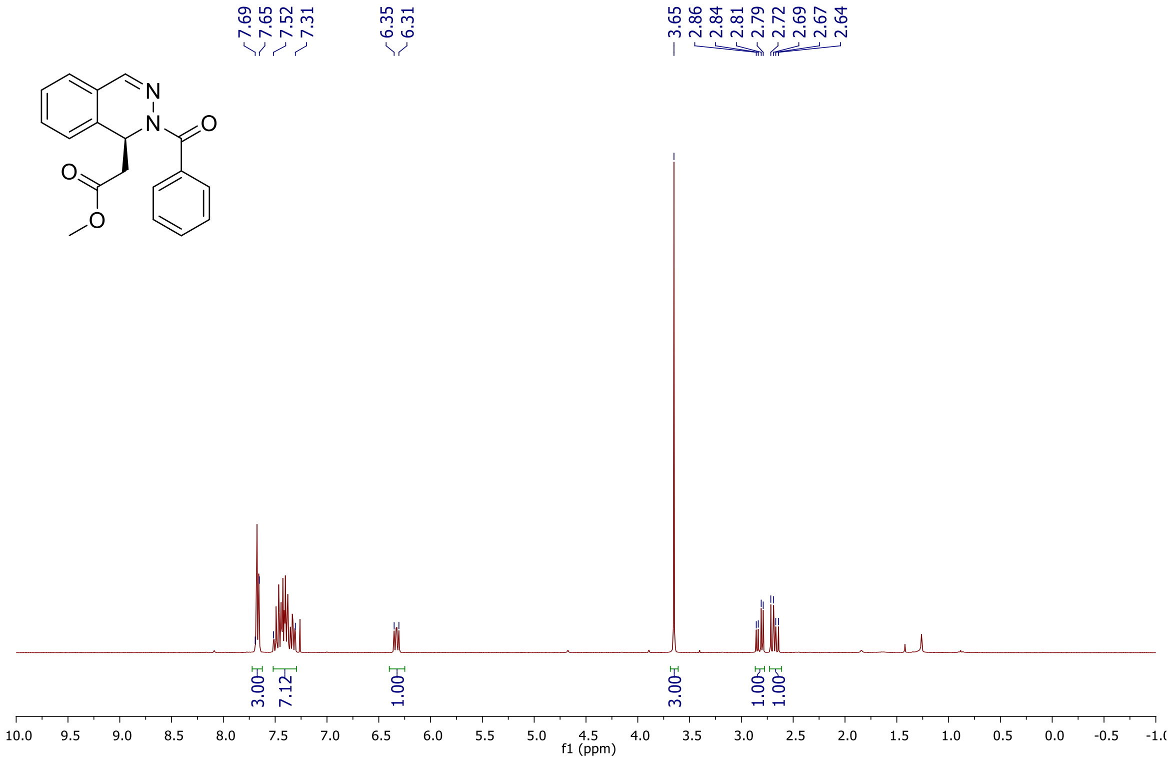

^{13}**C NMR** (CDCl$_3$, 75.5 MHz) of **(*S*)-8ab**

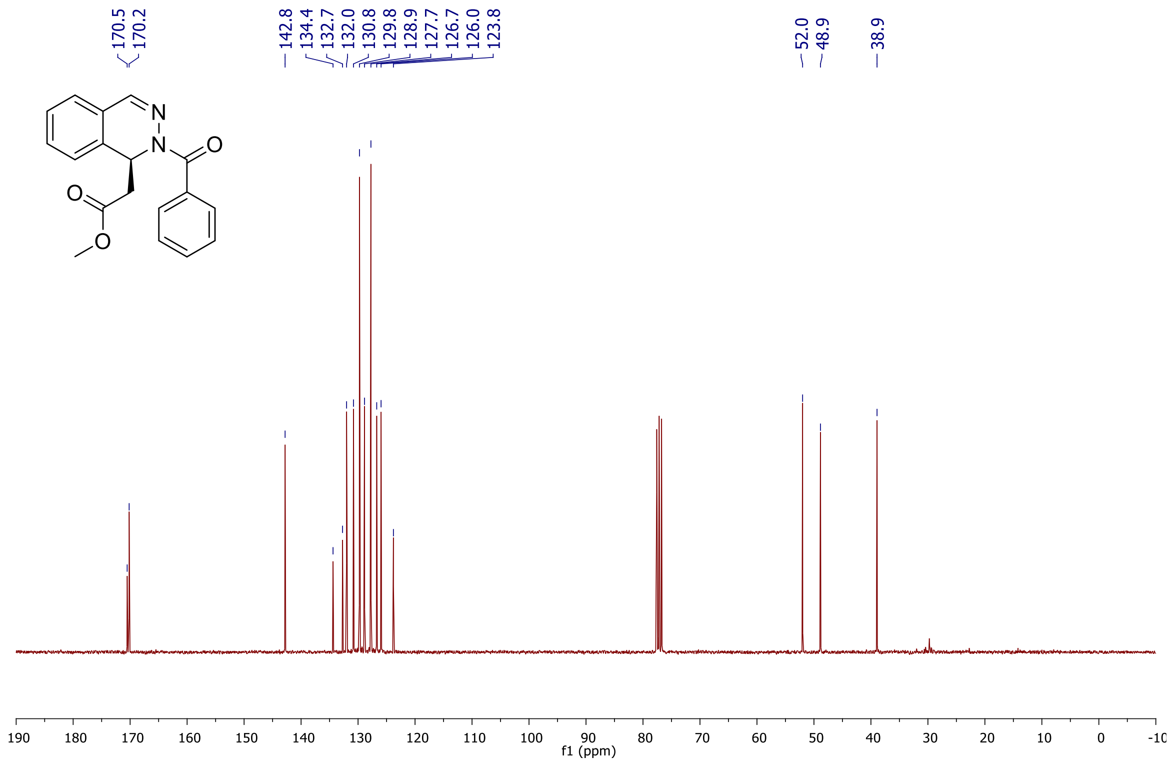

^{1}H NMR (CDCl$_3$, 300 MHz) of **(*S*)-8ac**

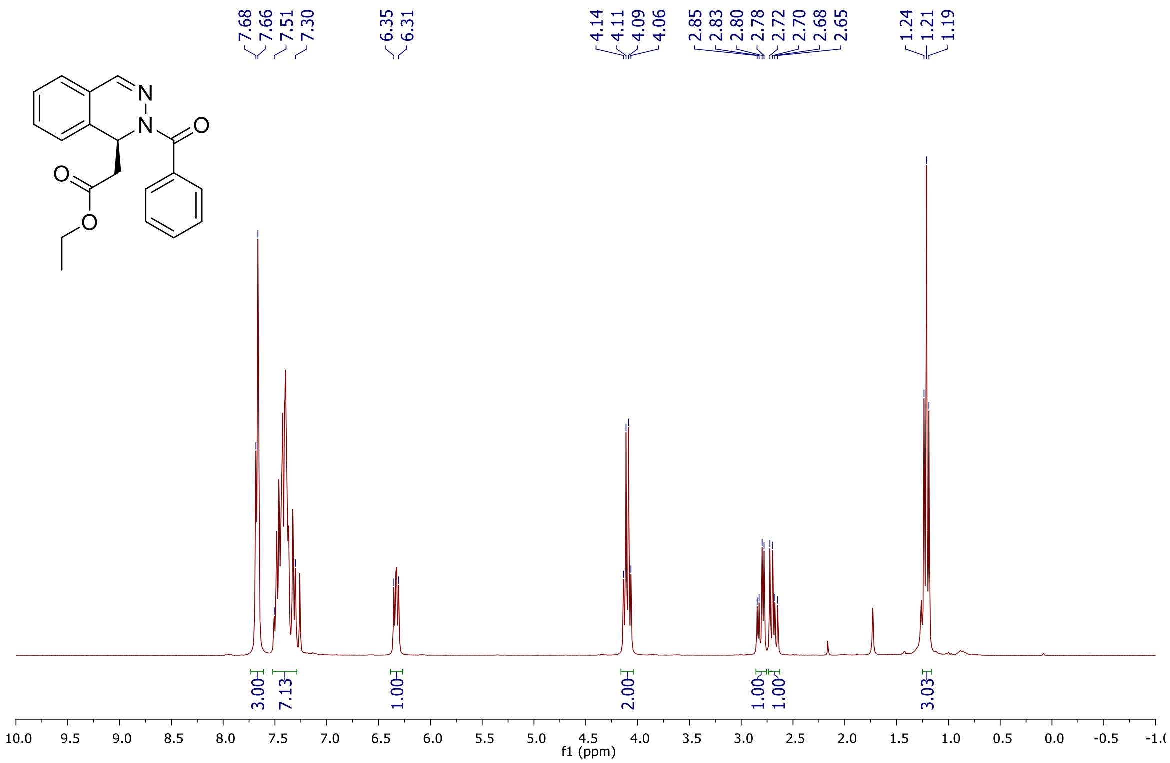

^{13}C NMR (CDCl$_3$, 75.5 MHz) of **(*S*)-8ac**

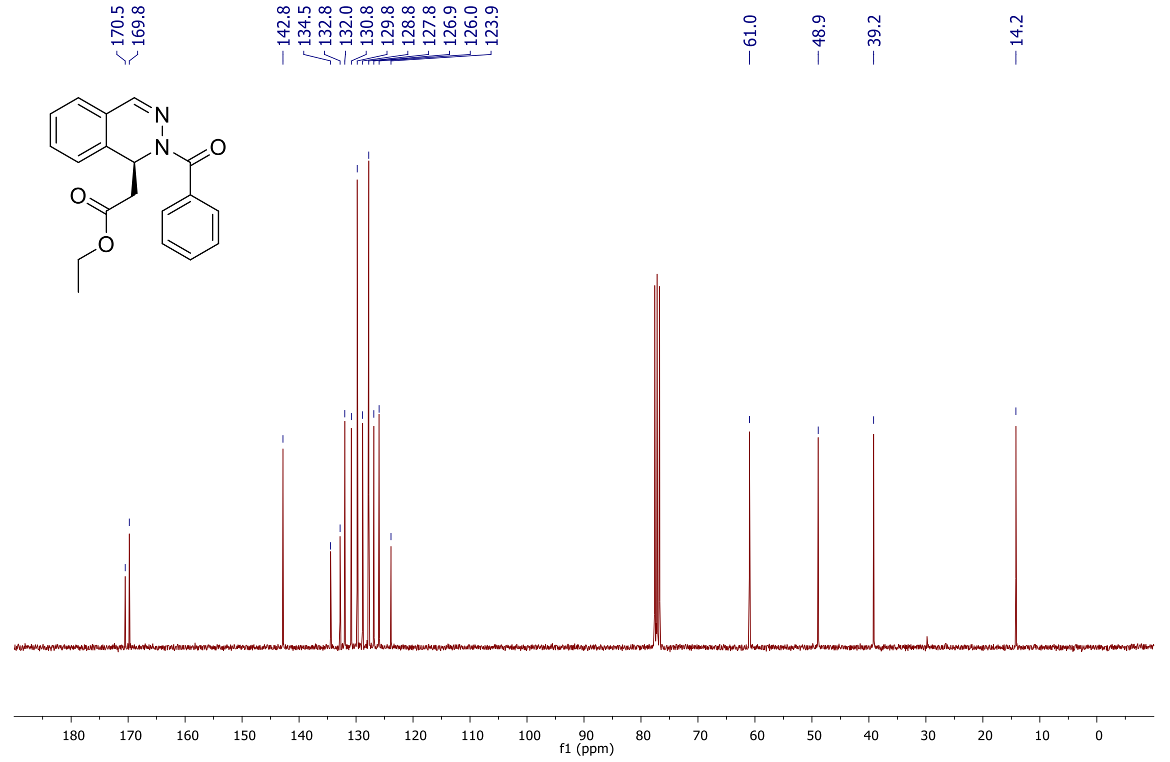

^{1}**H NMR** (CDCl$_3$, 300 MHz) of **(*S*)-8ad**

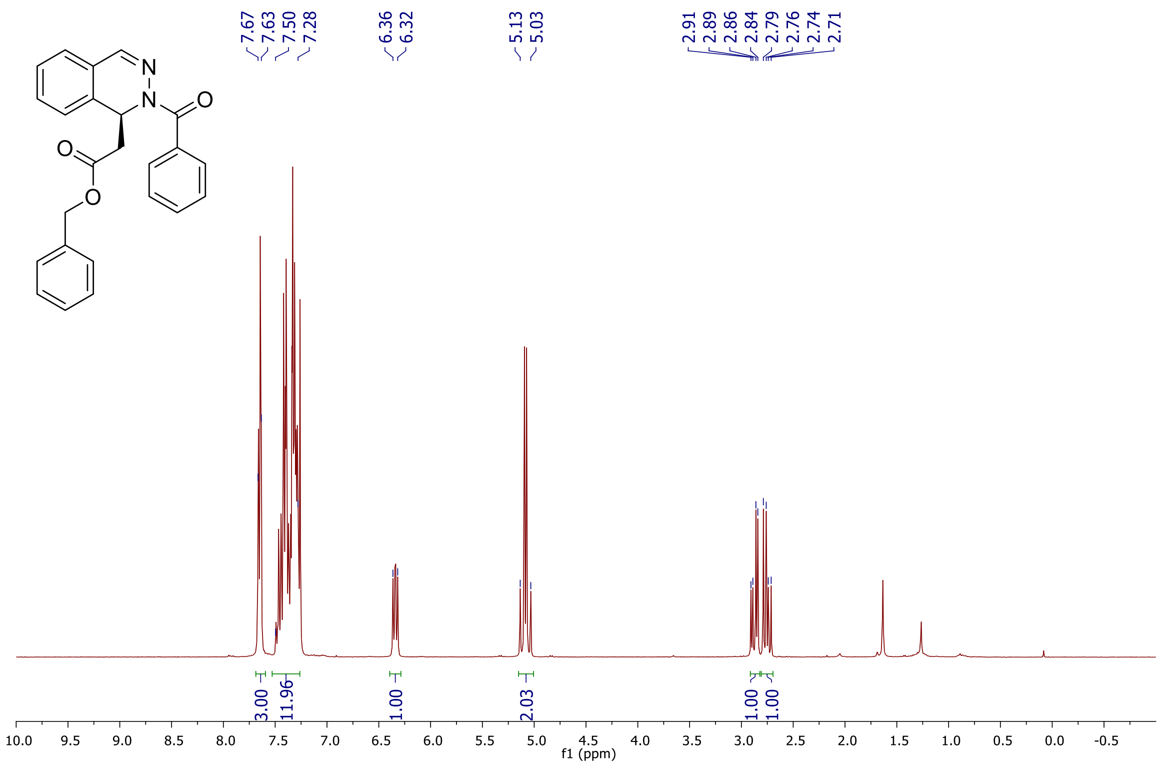

^{13}**C NMR** (CDCl$_3$, 75.5 MHz) of **(*S*)-8ad**

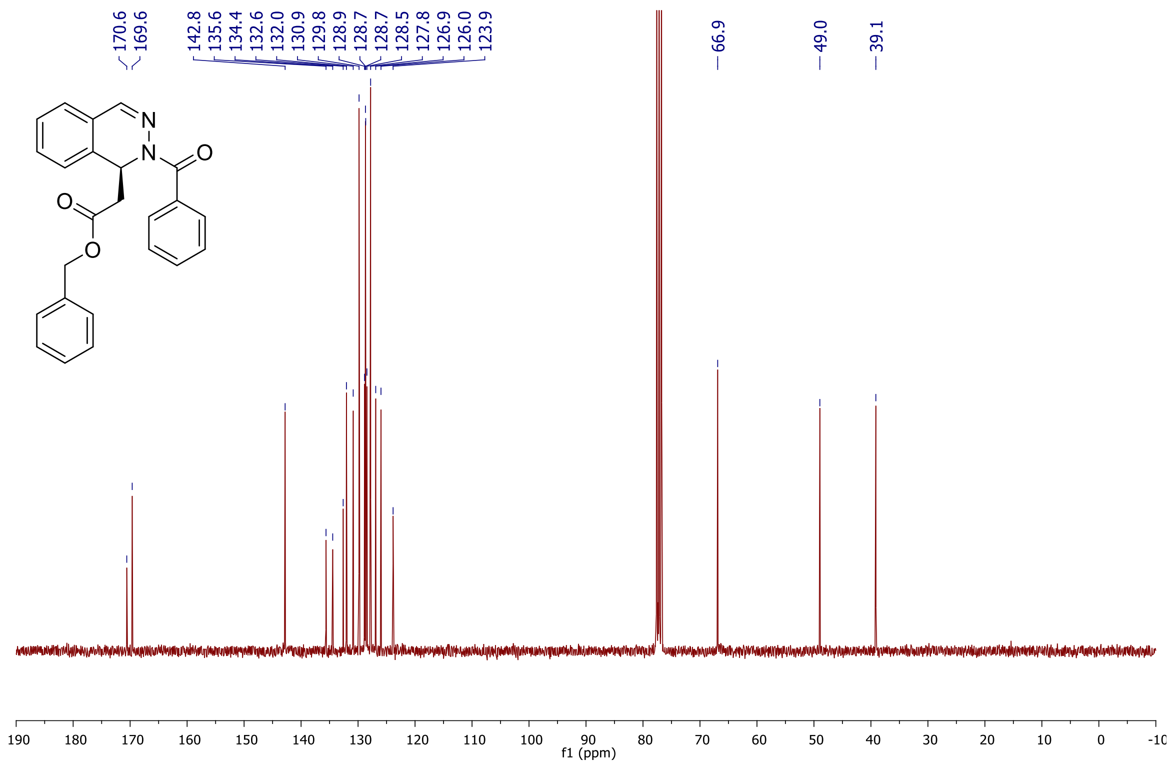

^{1}H NMR (CDCl$_3$, 300 MHz) of **(*S*)-8ae**

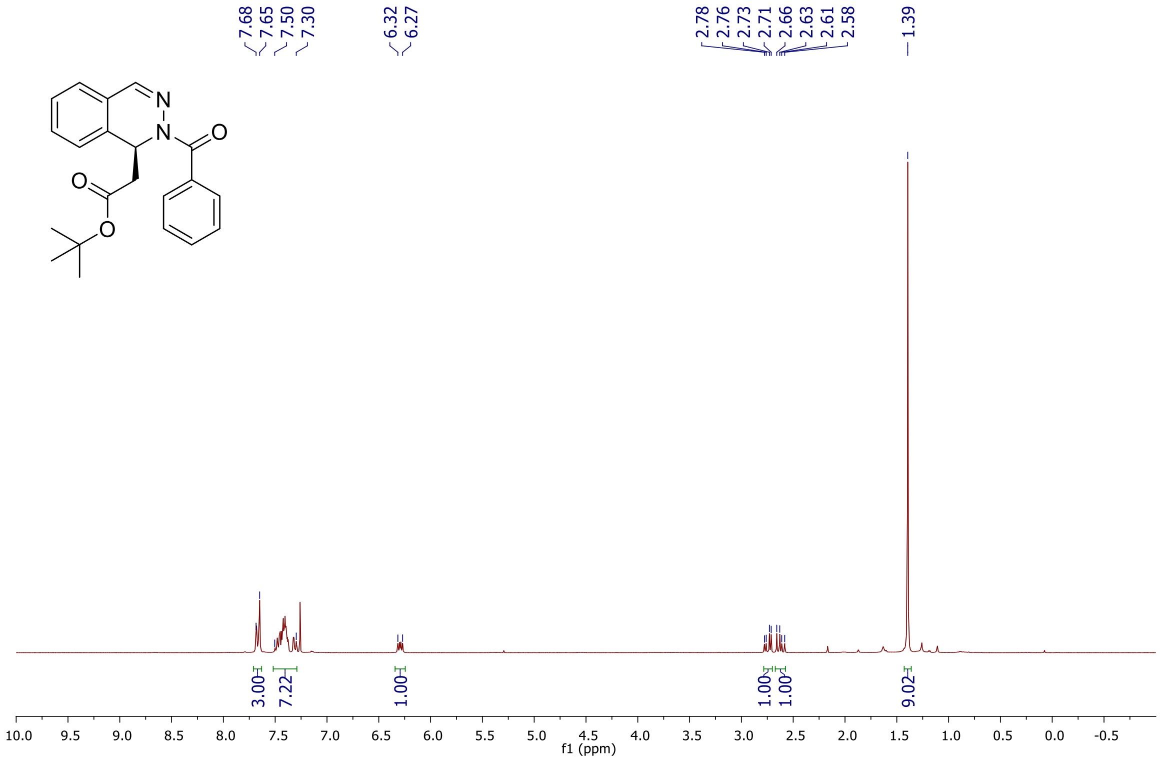

^{13}C NMR (CDCl$_3$, 75.5 MHz) of **(*S*)-8ae**

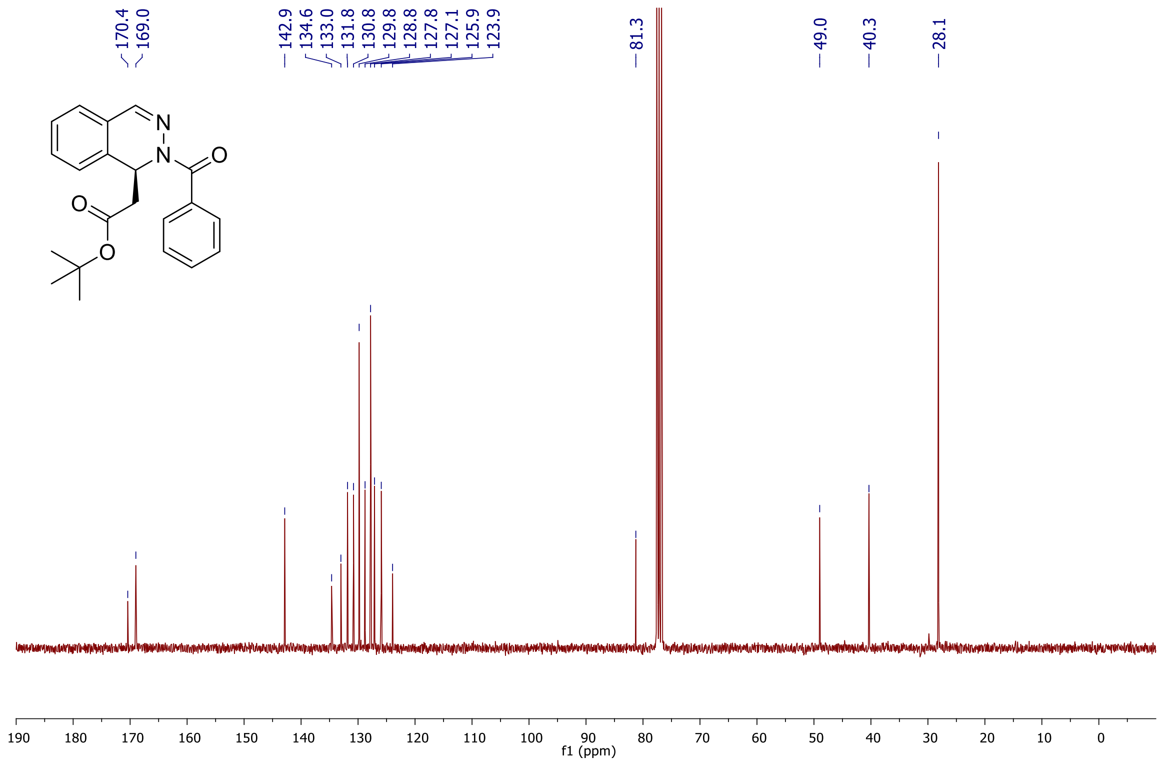

^{1}H NMR (CDCl$_3$, 300 MHz) of **(*S*)-8af**

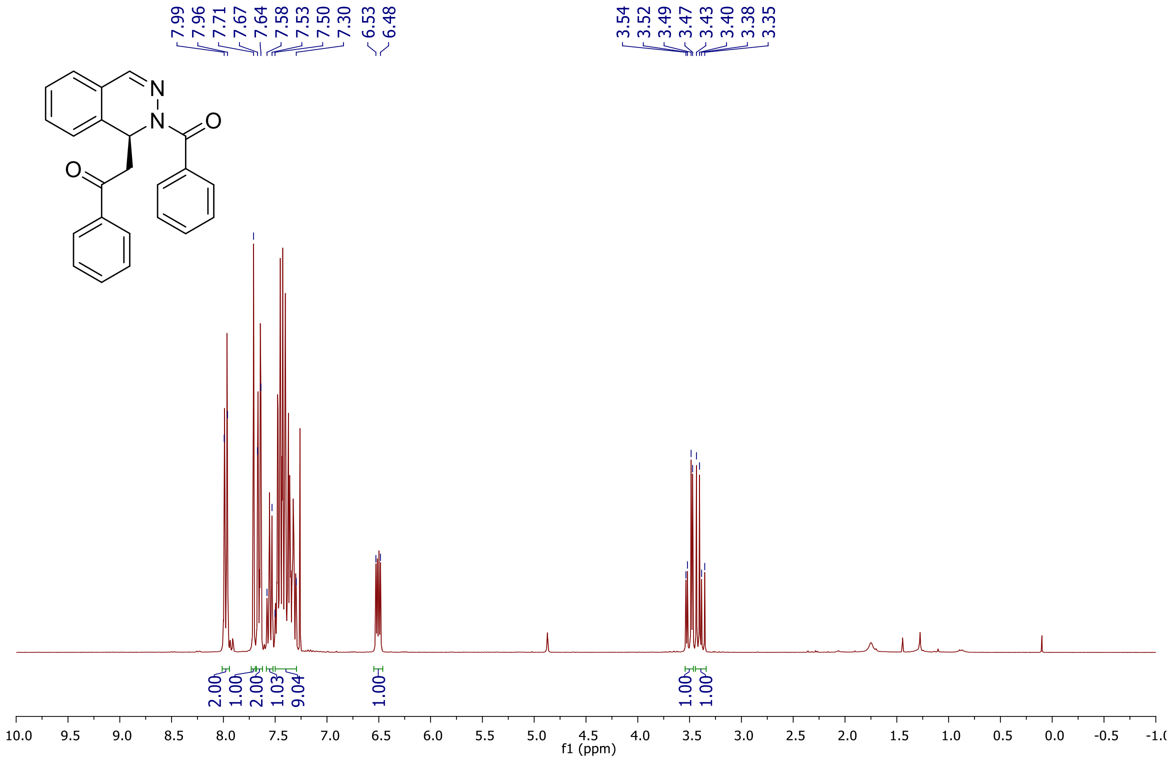

^{13}C NMR (CDCl$_3$, 75.5 MHz) of **(*S*)-8af**

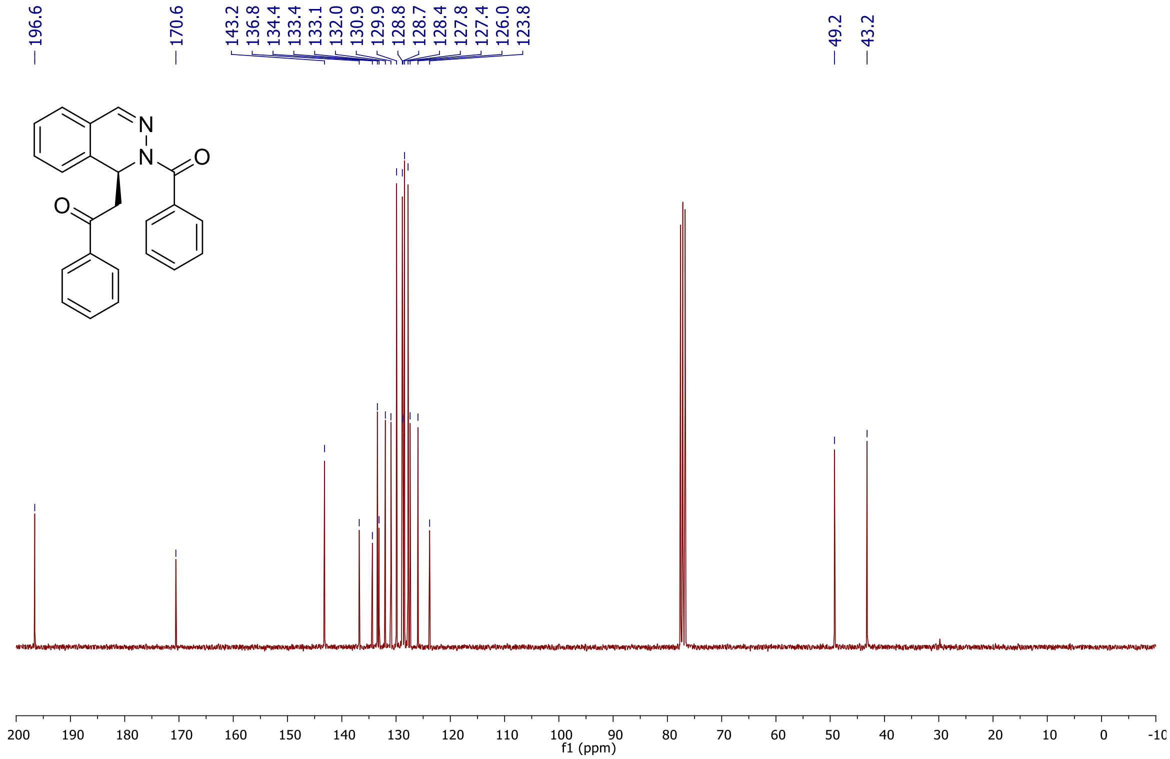

^{1}H NMR (CDCl$_3$, 300 MHz) of **(*S*)-8ag**

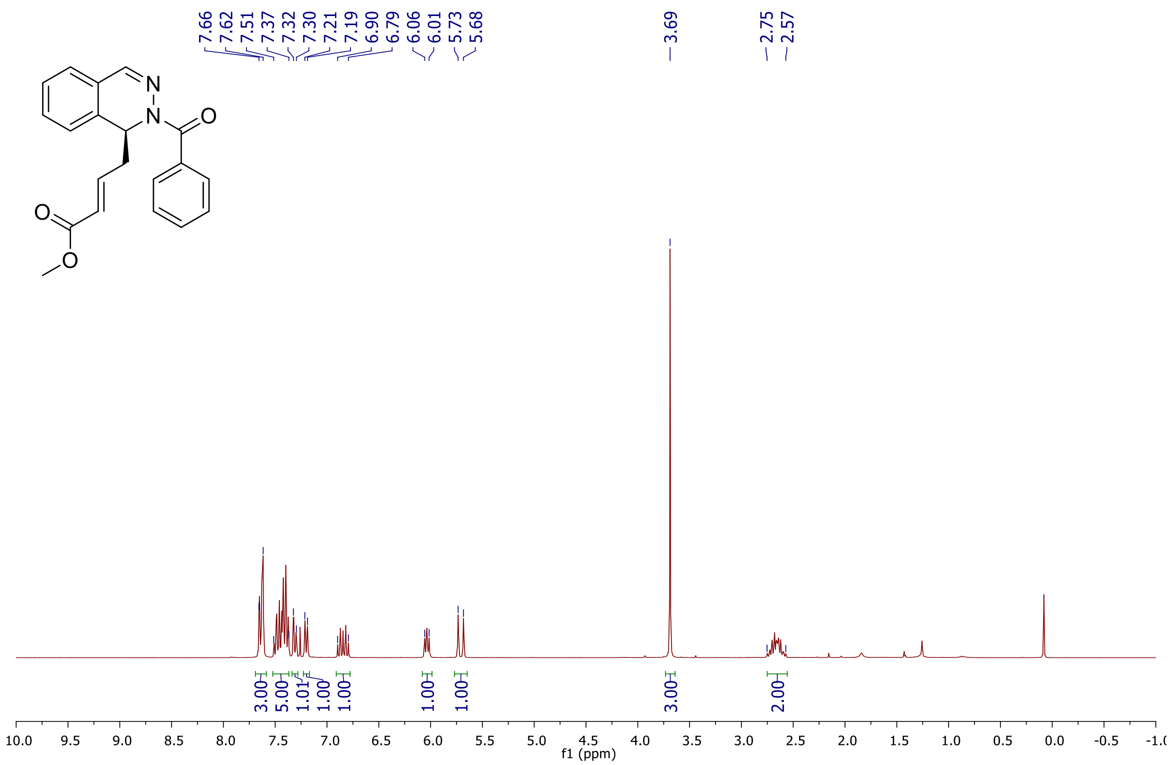

^{13}C NMR (CDCl$_3$, 75.5 MHz) of **(*S*)-8ag**

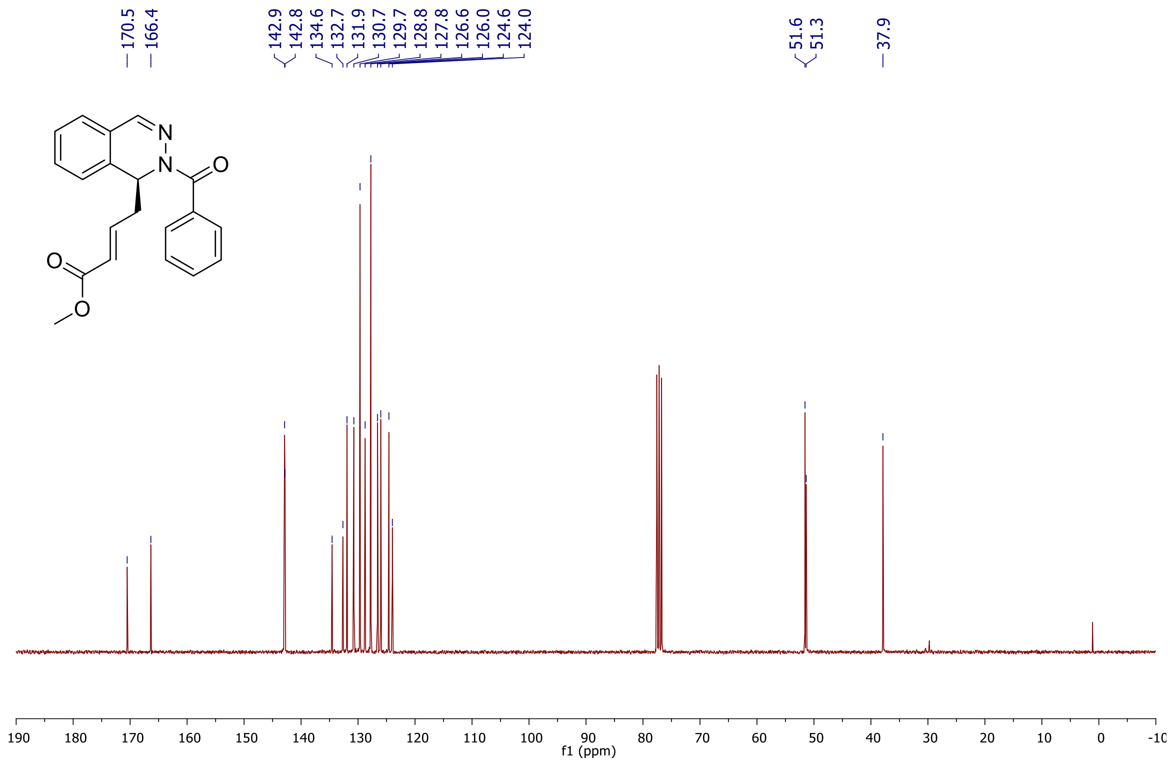

[1]**H NMR** (CDCl$_3$, 300 MHz) of **(*S*)-8ba**

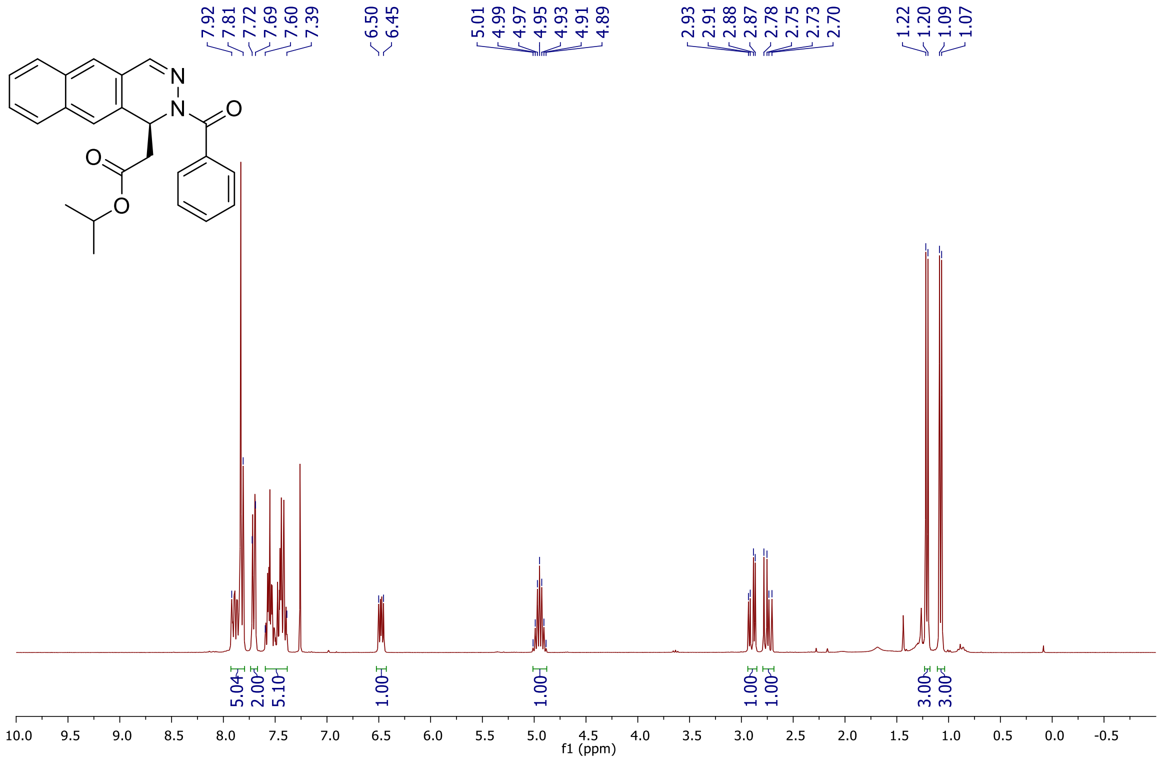

[13]**C NMR** (CDCl$_3$, 75.5 MHz) of **(*S*)-8ba**

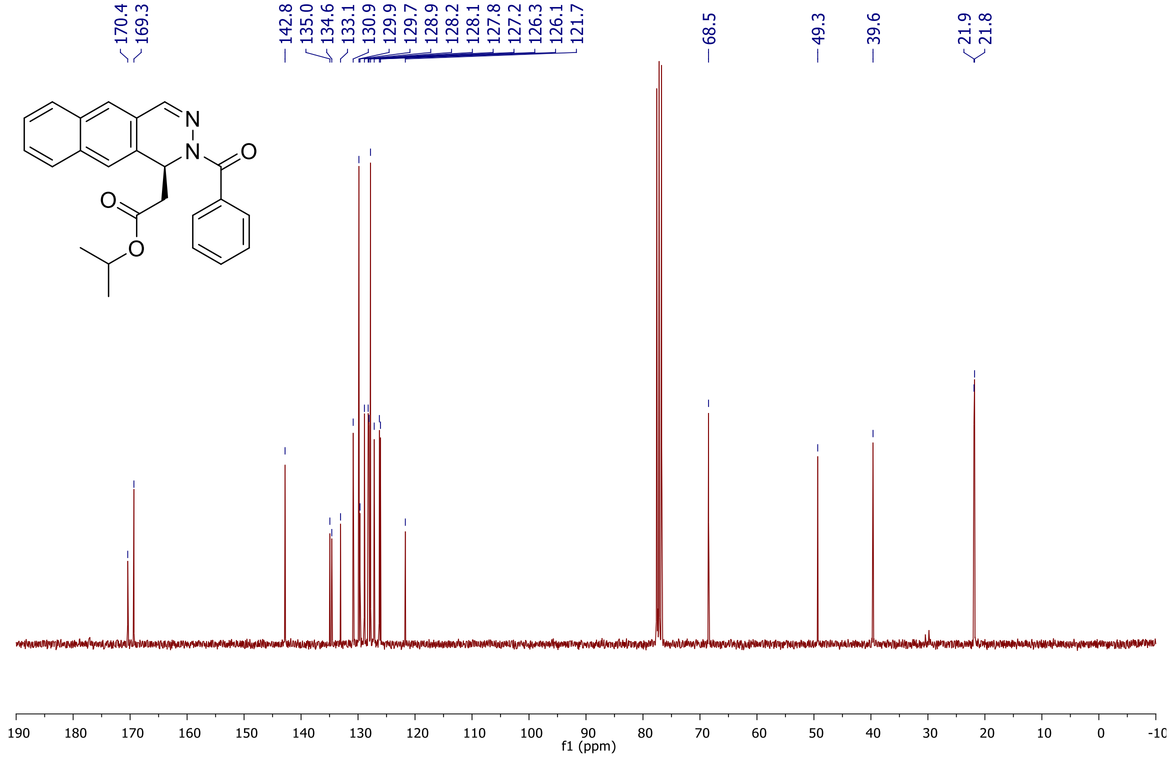

^{1}H NMR (CDCl$_3$, 300 MHz) of **(*S*)-8ca**

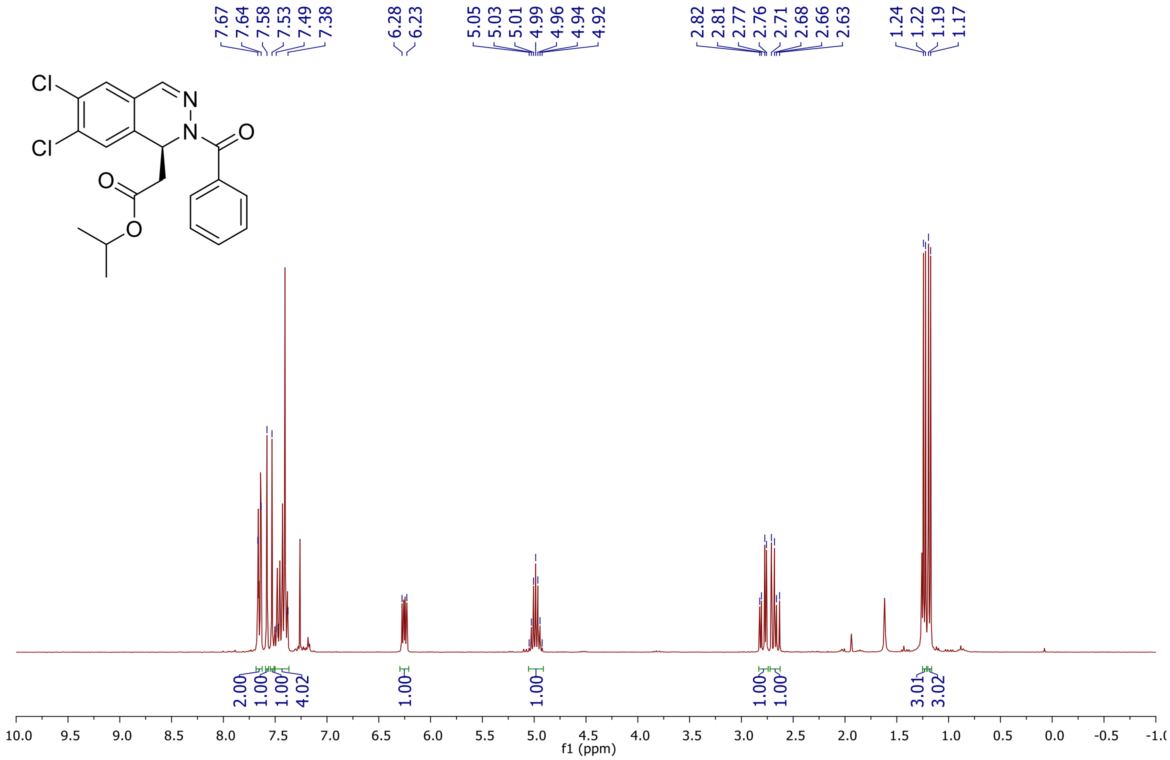

^{13}C NMR (CDCl$_3$, 75.5 MHz) of **(*S*)-8ca**

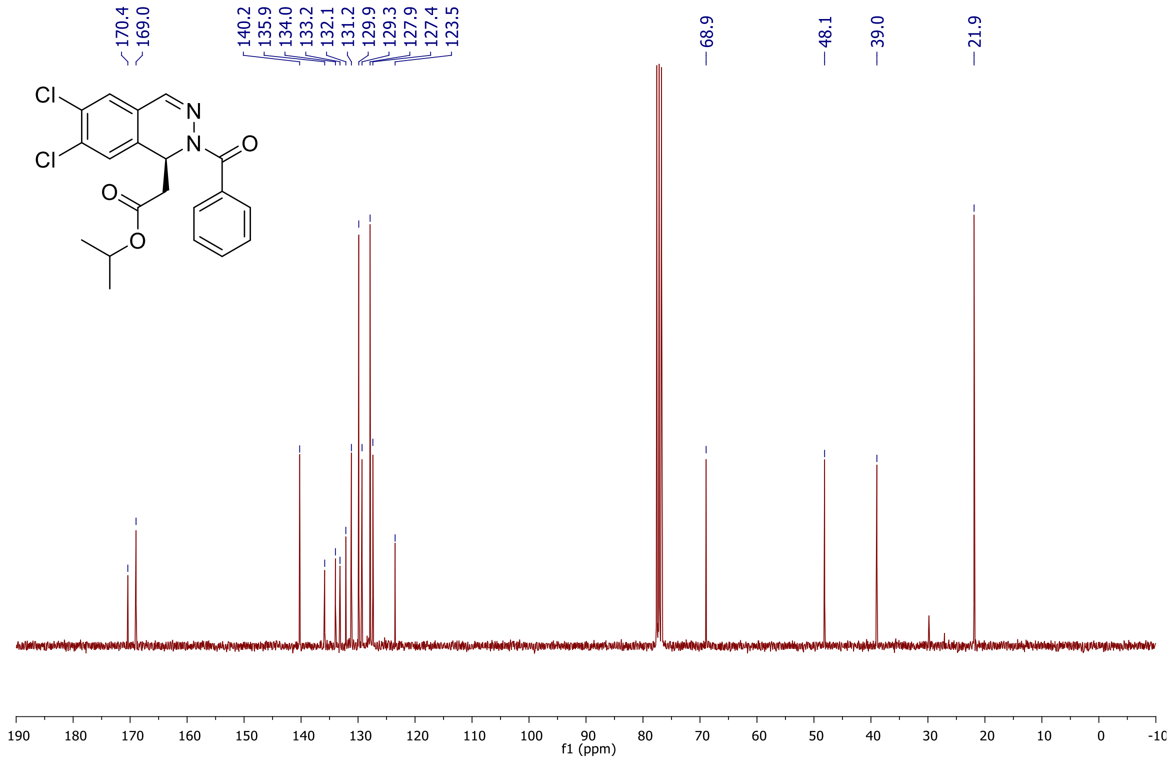

^{1}H NMR (CDCl$_3$, 300 MHz) of **(*S*)-8da**

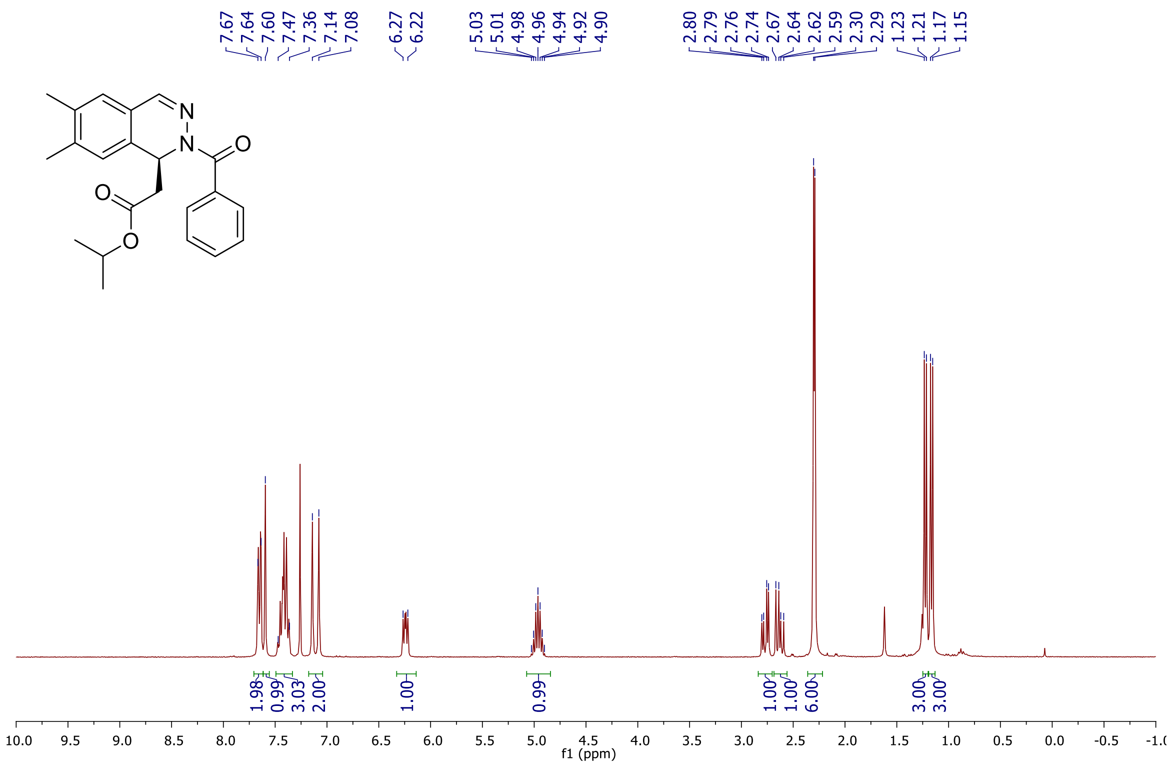

^{13}C NMR (CDCl$_3$, 75.5 MHz) of **(*S*)-8da**

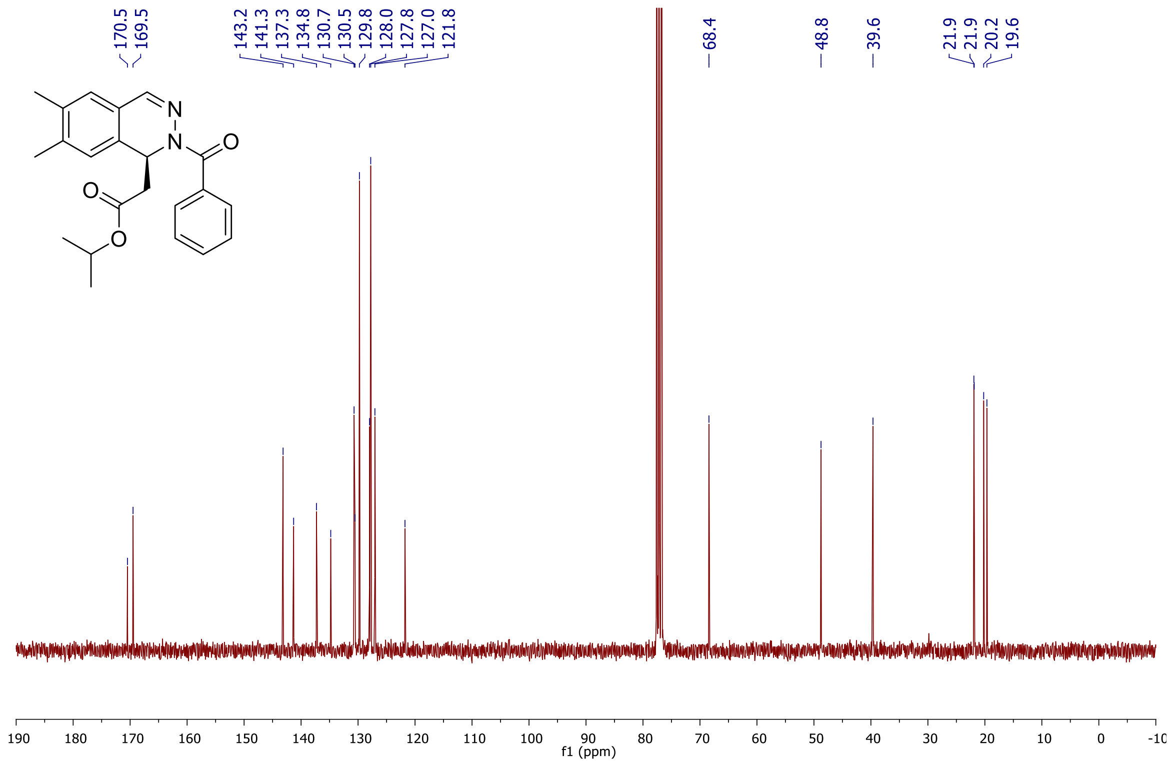

^{1}H NMR ($CDCl_3$, 300 MHz) of **(*S*)-8ea**

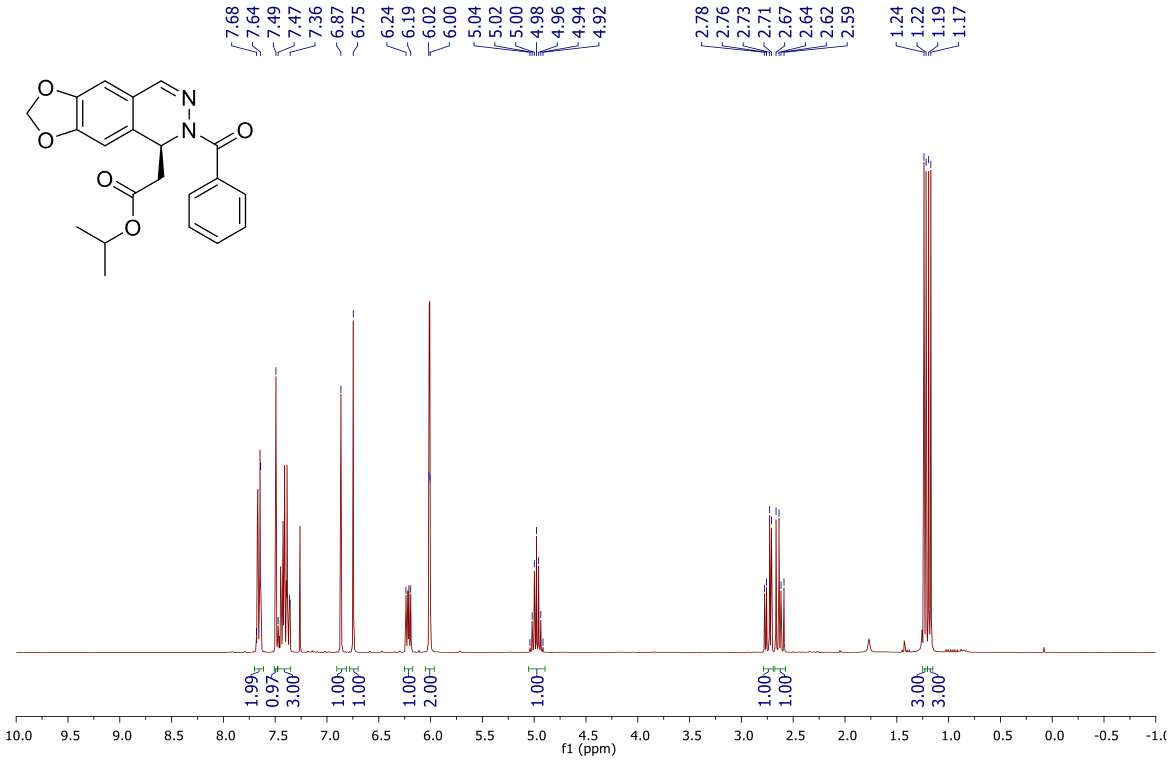

^{13}C NMR ($CDCl_3$, 75.5 MHz) of **(*S*)-8ea**

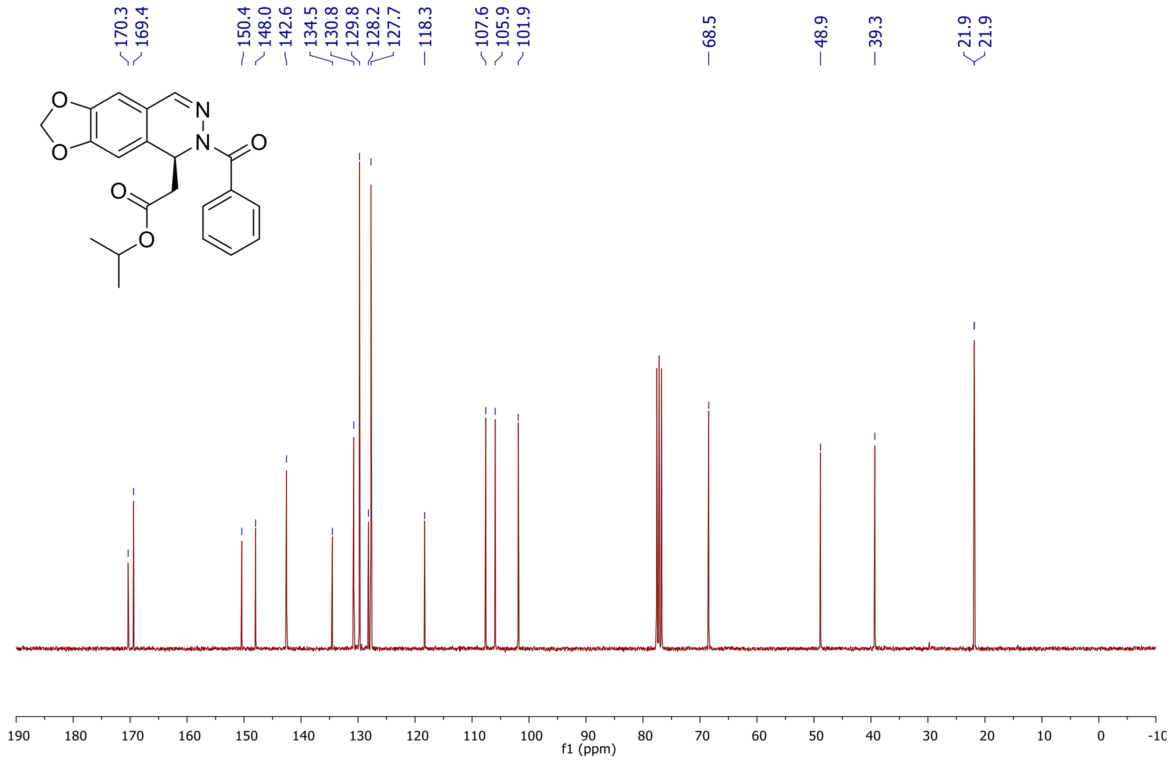

^{1}H NMR ($CDCl_3$, 300 MHz) of **(*S*)-8fa**

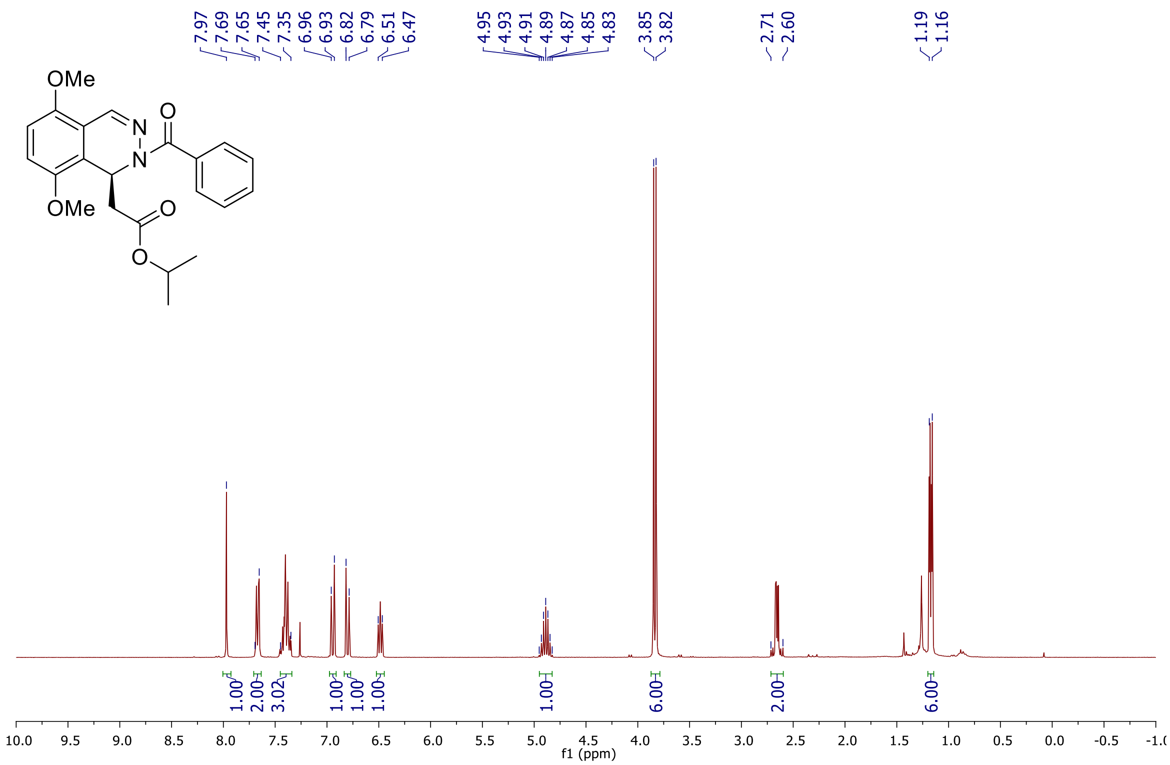

^{13}C NMR ($CDCl_3$, 75.5 MHz) of **(*S*)-8fa**

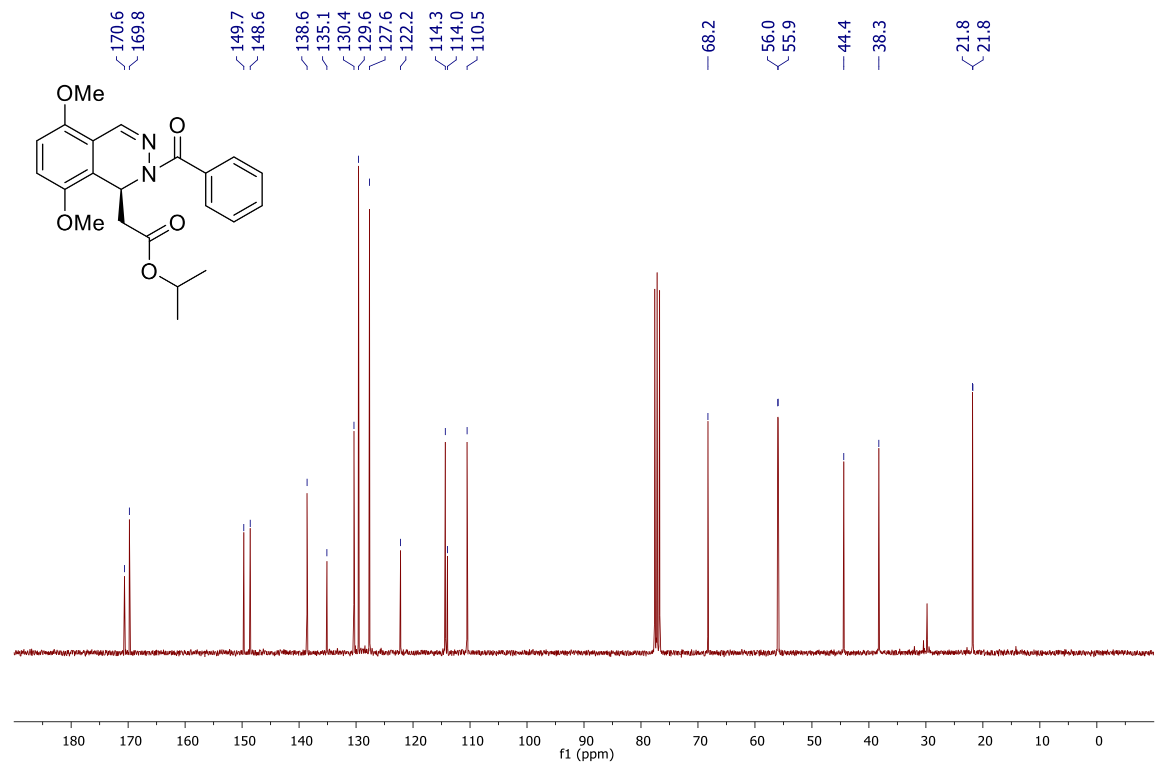

^{1}H NMR ($CDCl_3$, 300 MHz) of **(*S*)-8ga/8ga´**

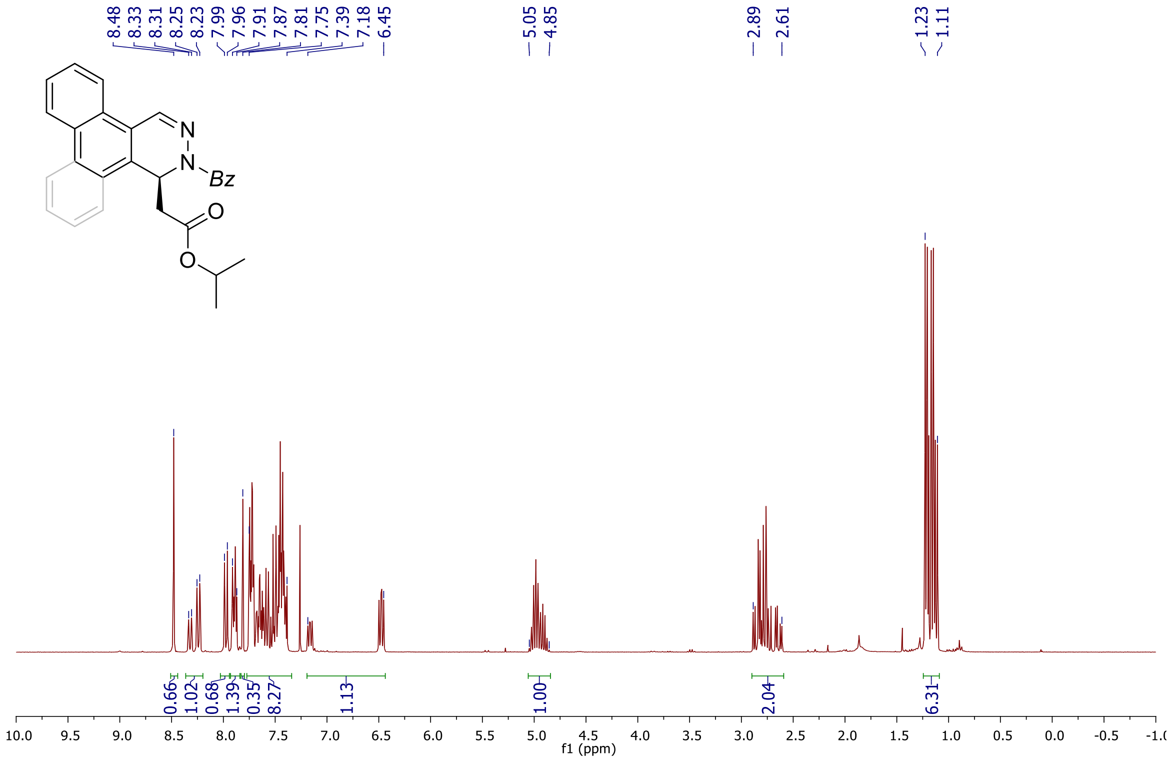

^{13}C NMR ($CDCl_3$, 75.5 MHz) of **(*S*)-8ga/8ga´**

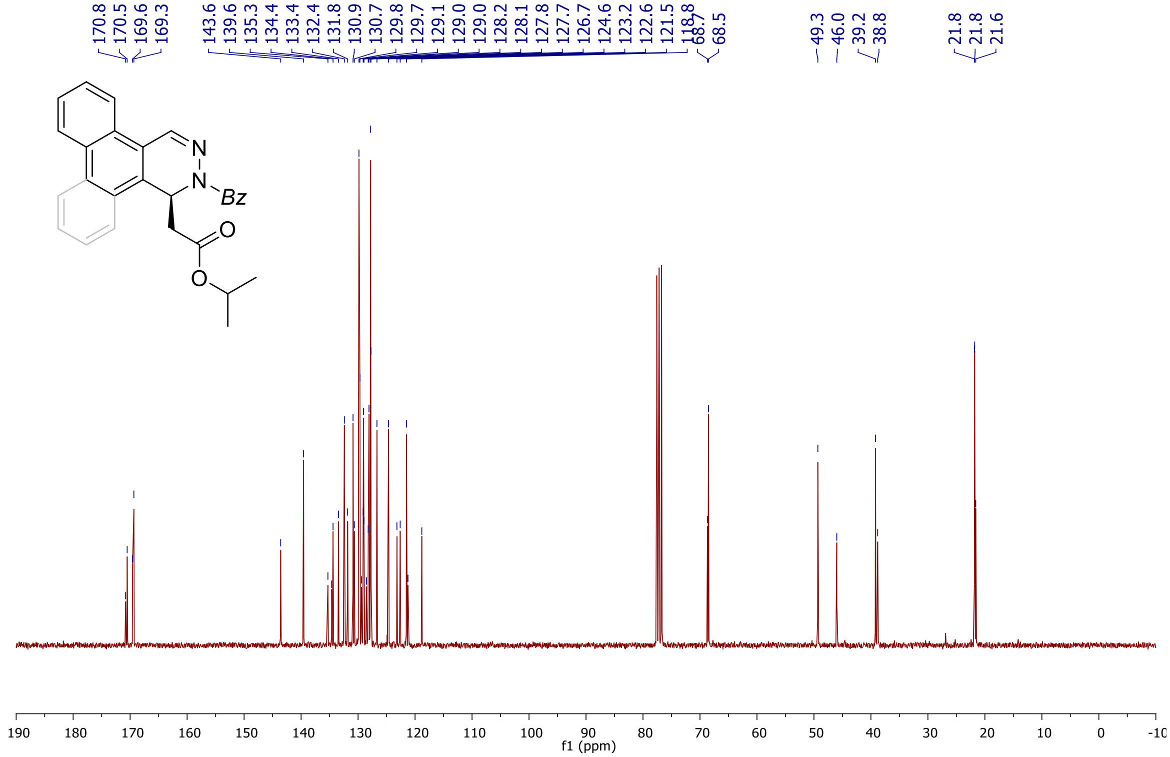

^{1}H NMR (CDCl$_3$, 300 MHz) of ***(S)*-8ha**

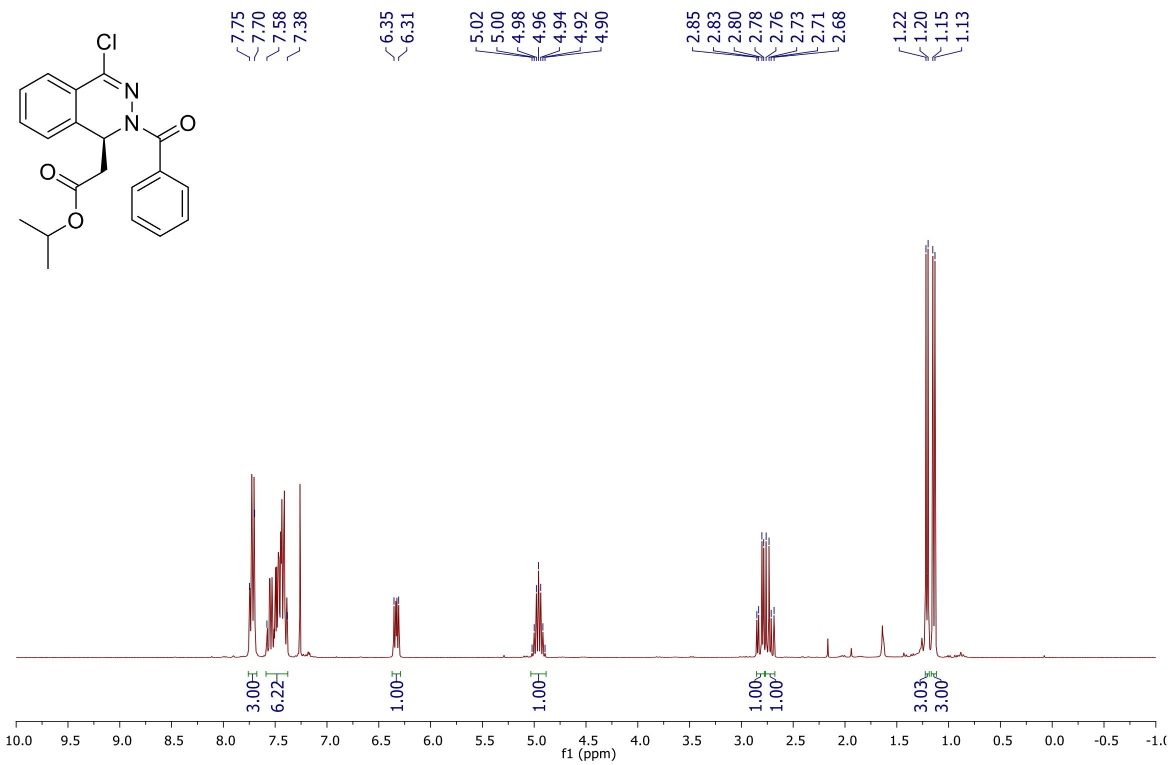

^{13}C NMR (CDCl$_3$, 126 MHz) of ***(S)*-8ha**

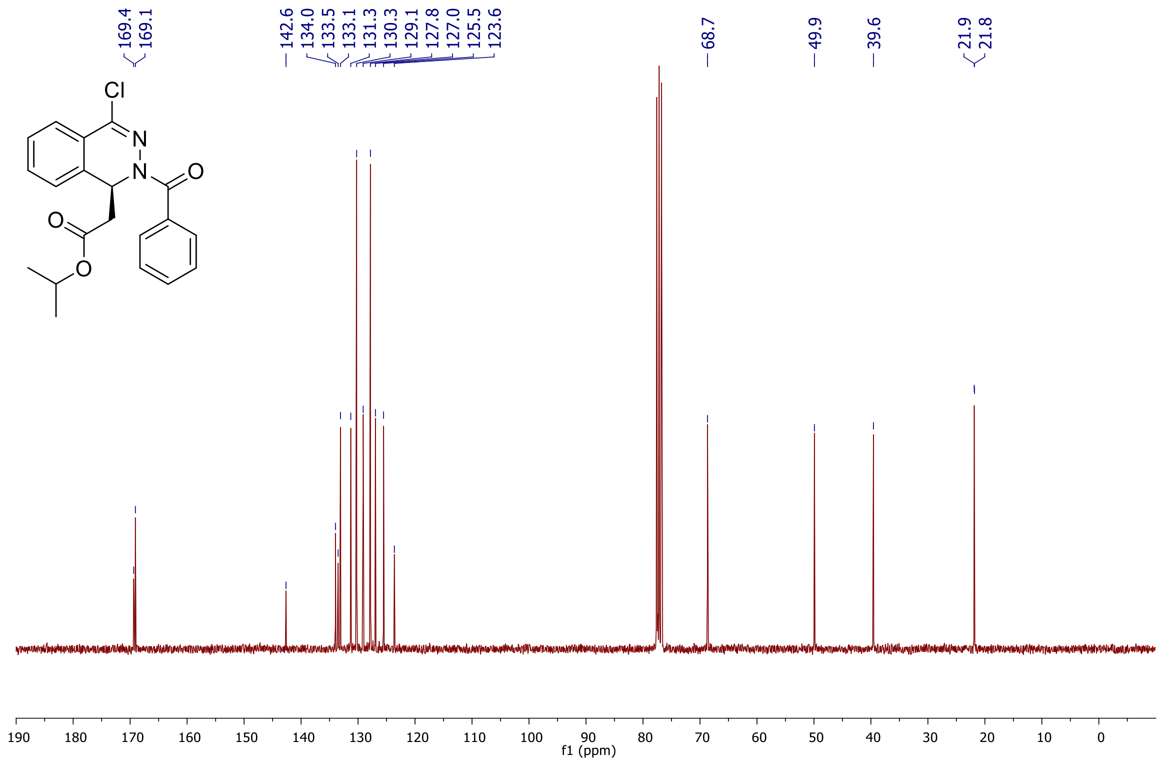

^{1}H NMR (CDCl$_3$, 300 MHz) of **(*S*)-8ia**

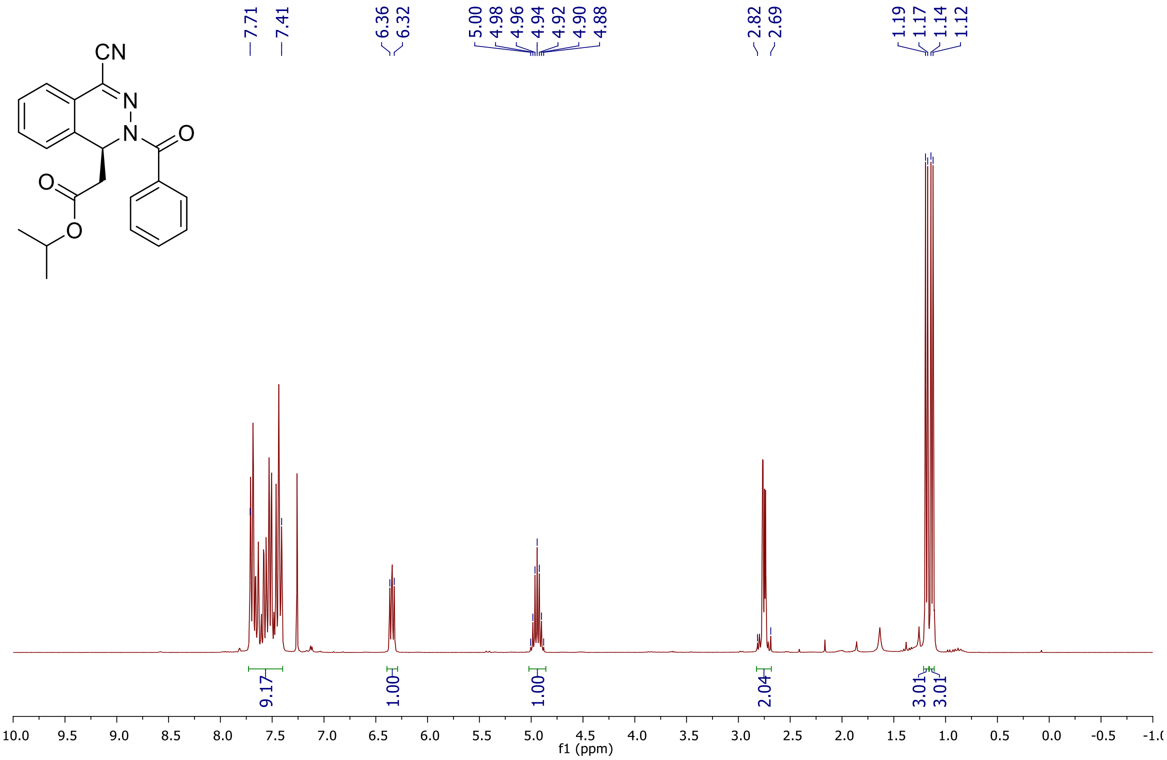

^{13}C NMR (CDCl$_3$, 75.5 MHz) of **(*S*)-8ia**

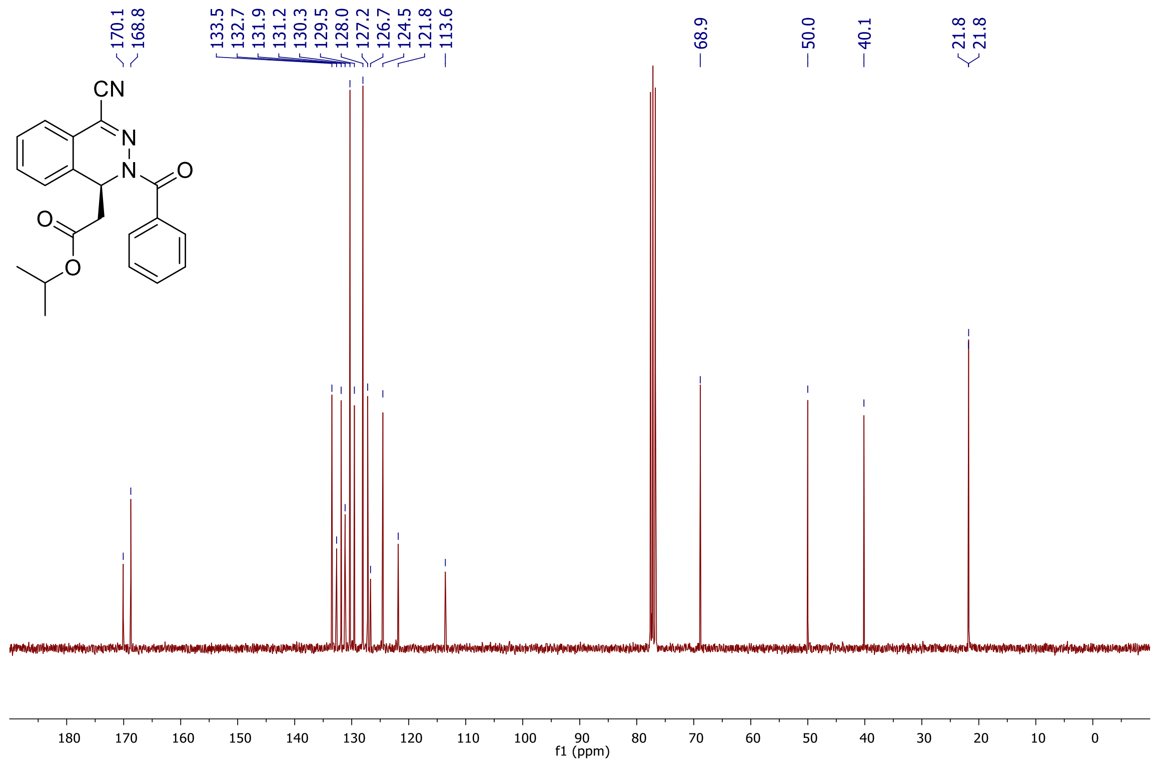

^{1}H NMR ($CDCl_3$, 500 MHz) of **(*S*)-8ja**

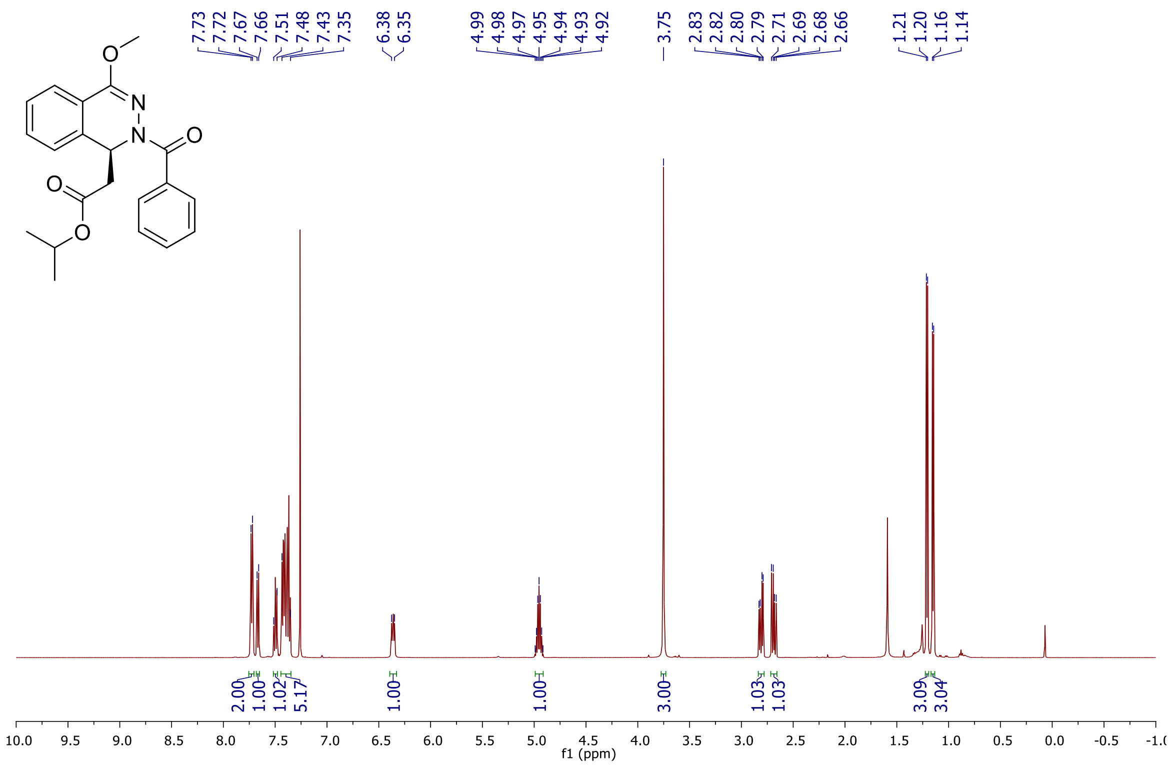

^{13}C NMR ($CDCl_3$, 126 MHz) of **(*S*)-8ja**

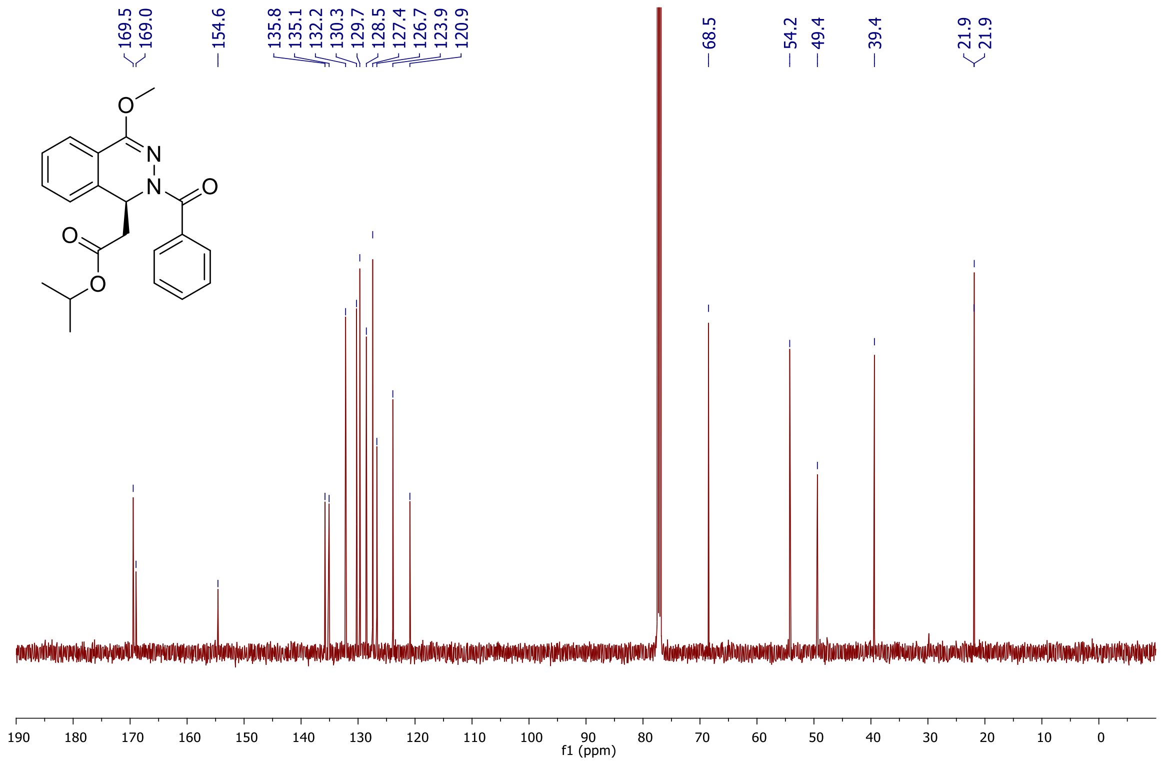

^{1}H NMR ($CDCl_3$, 300 MHz) of **(*S*)-8ka**

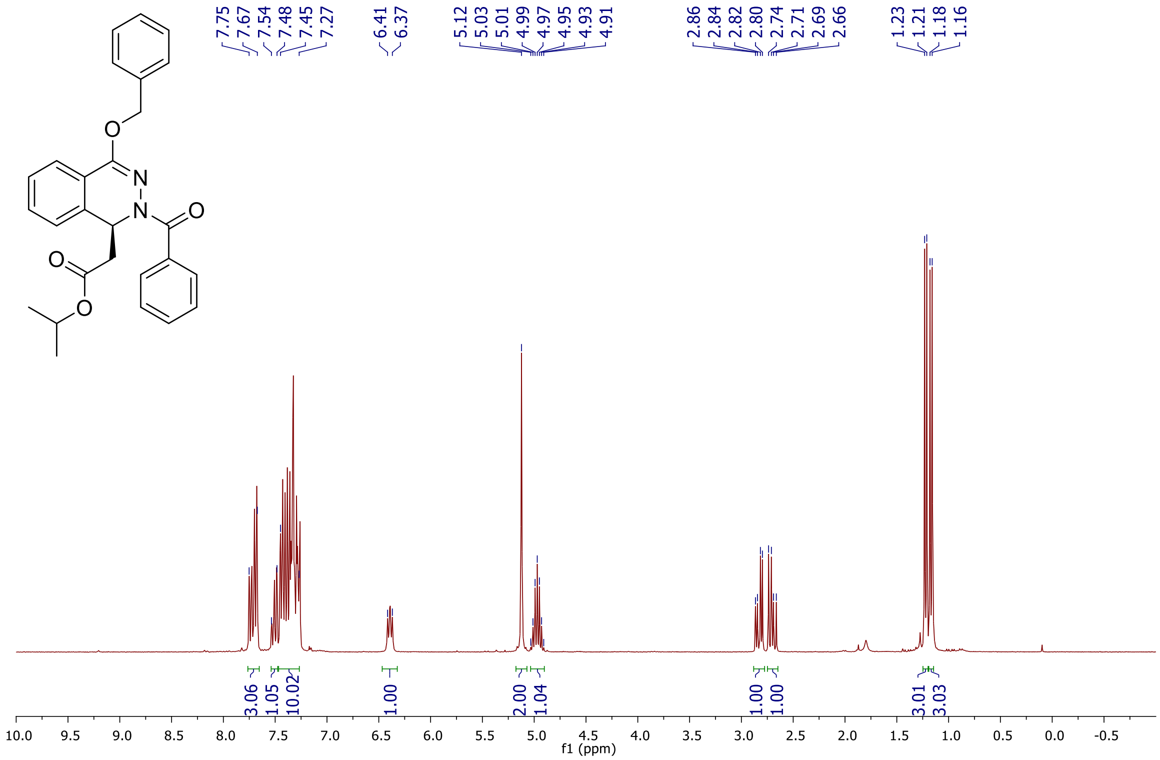

^{13}C NMR ($CDCl_3$, 75.5 MHz) of **(*S*)-8ka**

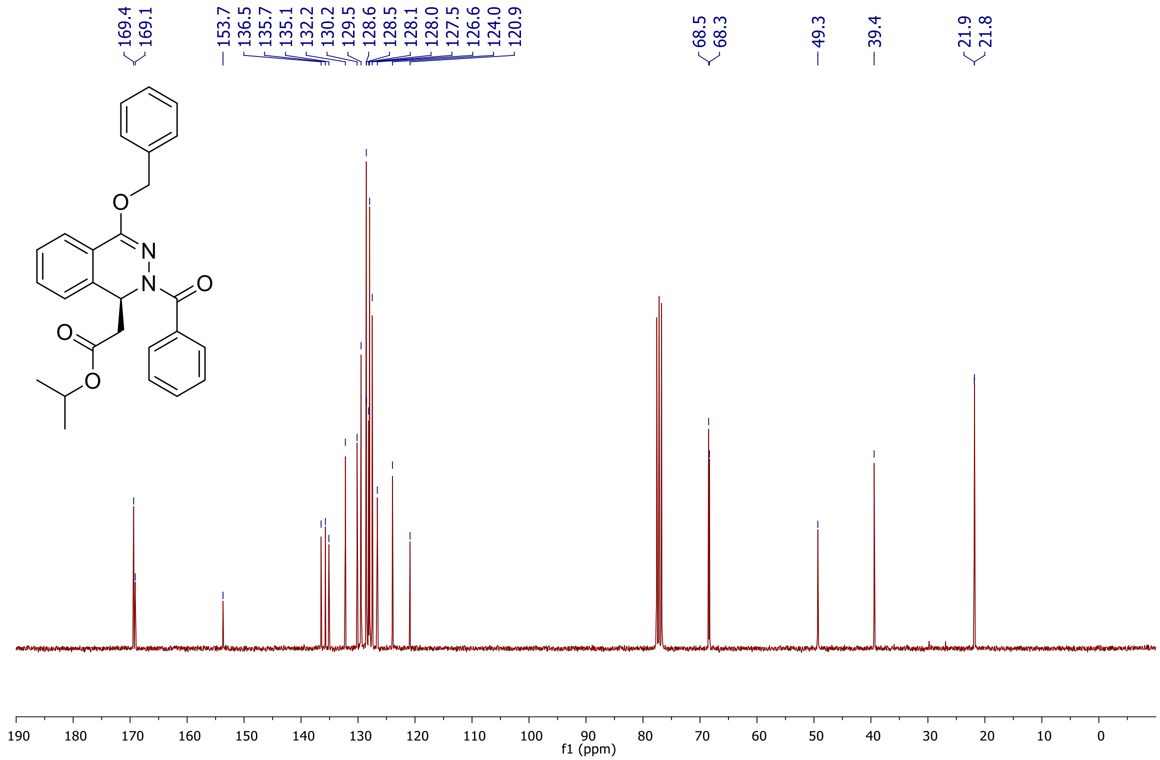

^{1}H NMR (CDCl$_3$, 300 MHz) of ***(S)*-8la**

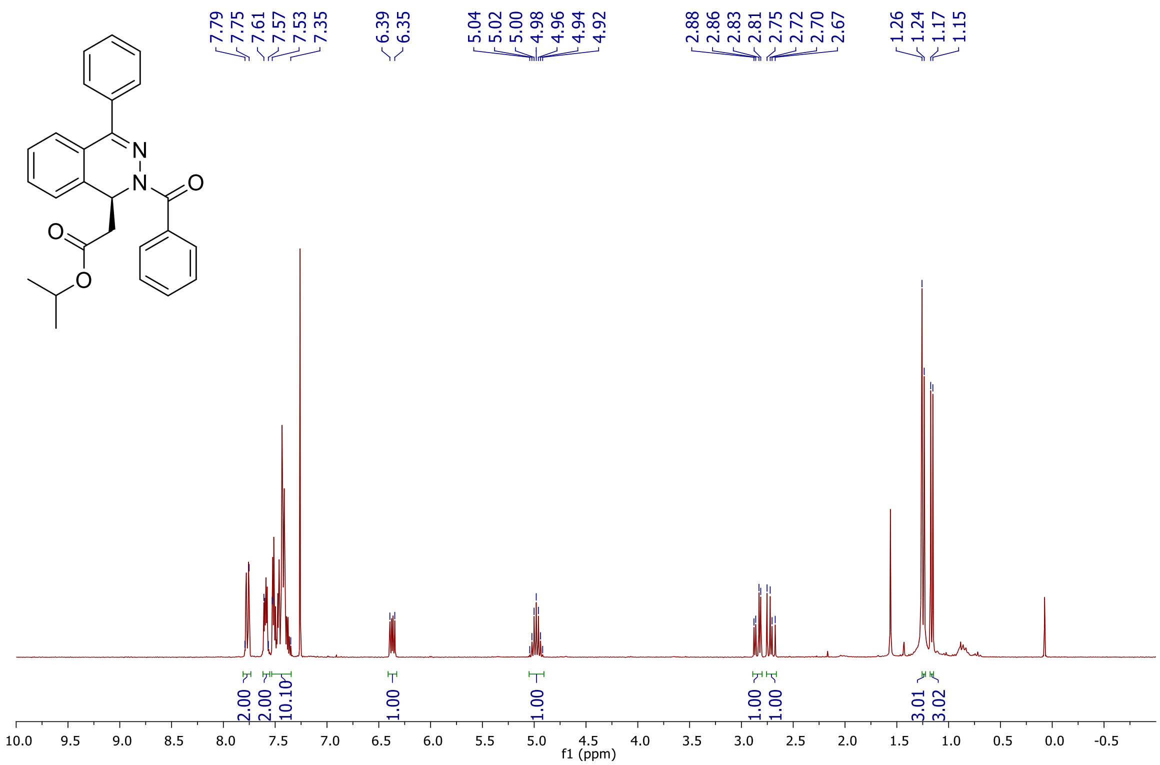

^{13}C NMR (CDCl$_3$, 126 MHz) of ***(S)*-8la**

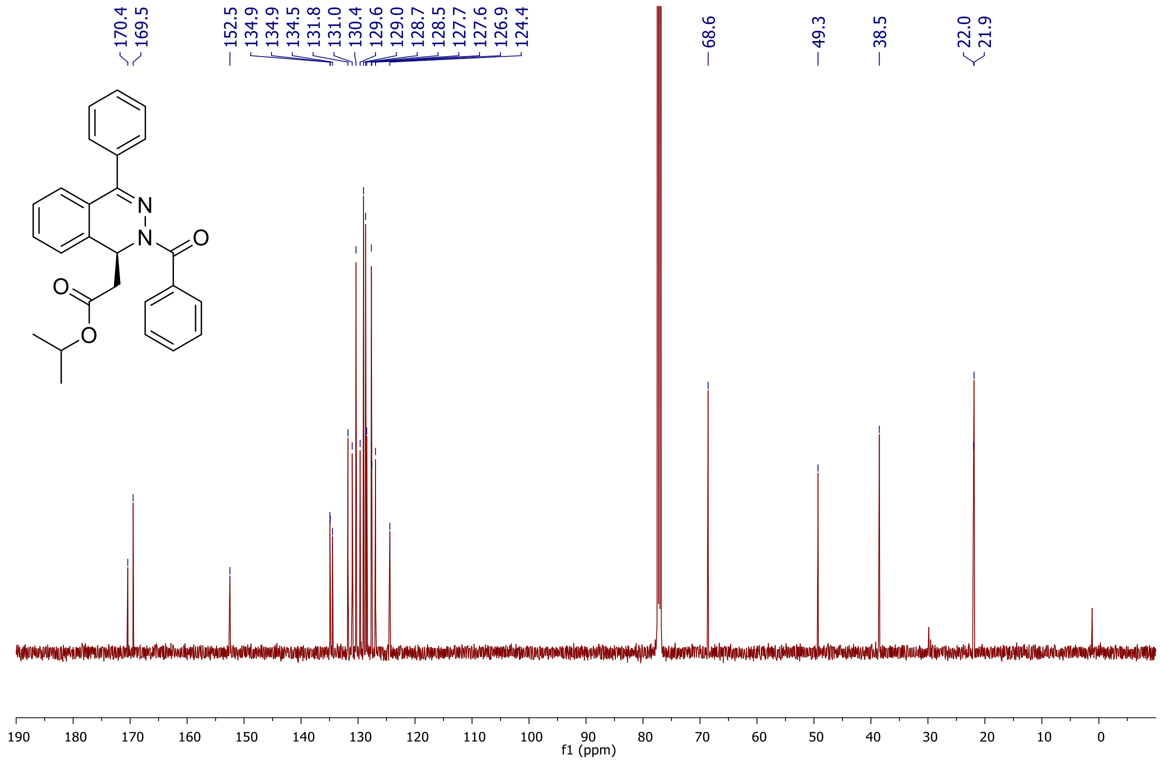

^{1}H NMR (CDCl$_3$, 300 MHz) of **(*S*)-8ma**

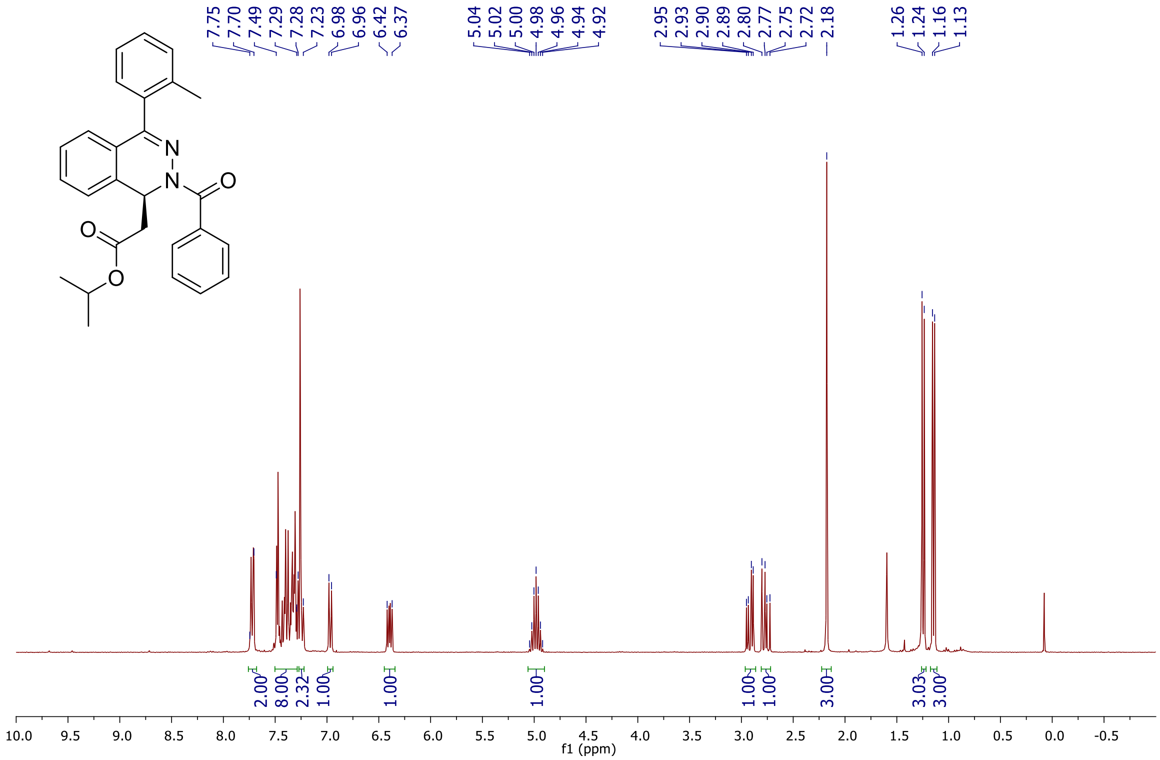

^{13}C NMR (CDCl$_3$, 75.5 MHz) of **(*S*)-8ma**

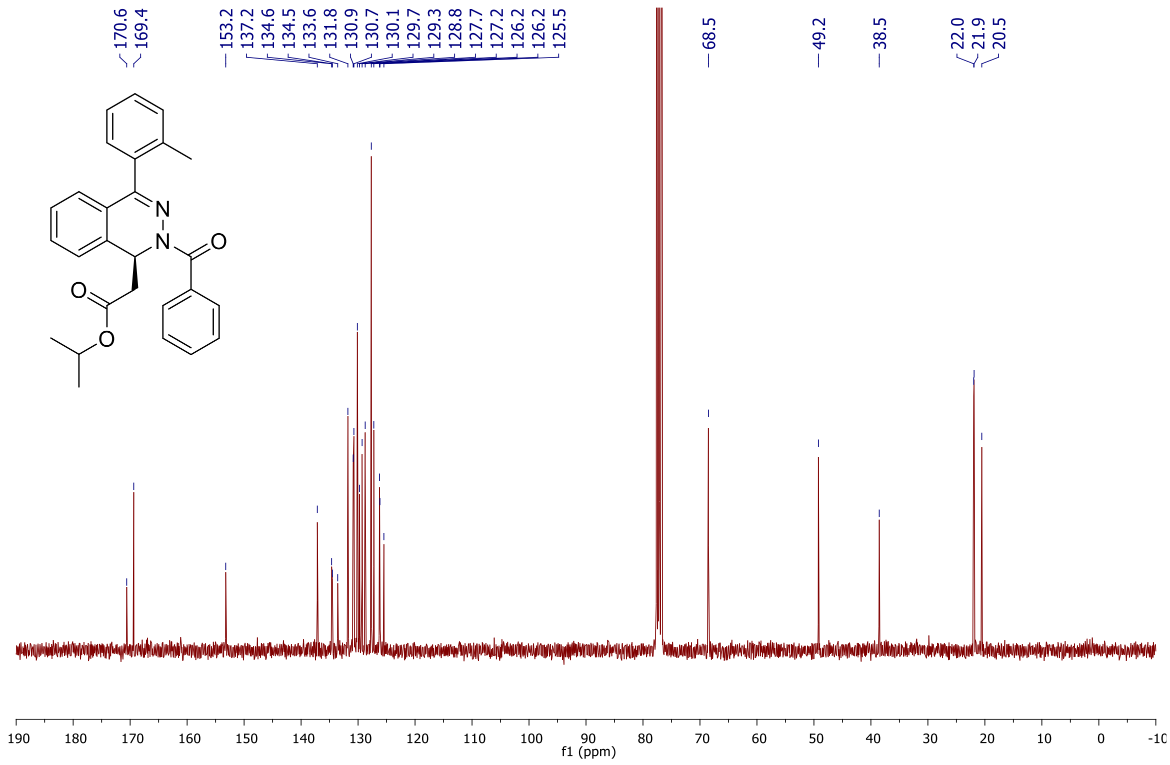

^{1}H NMR ($CDCl_3$, 300 MHz) of **(*S*)-8na**

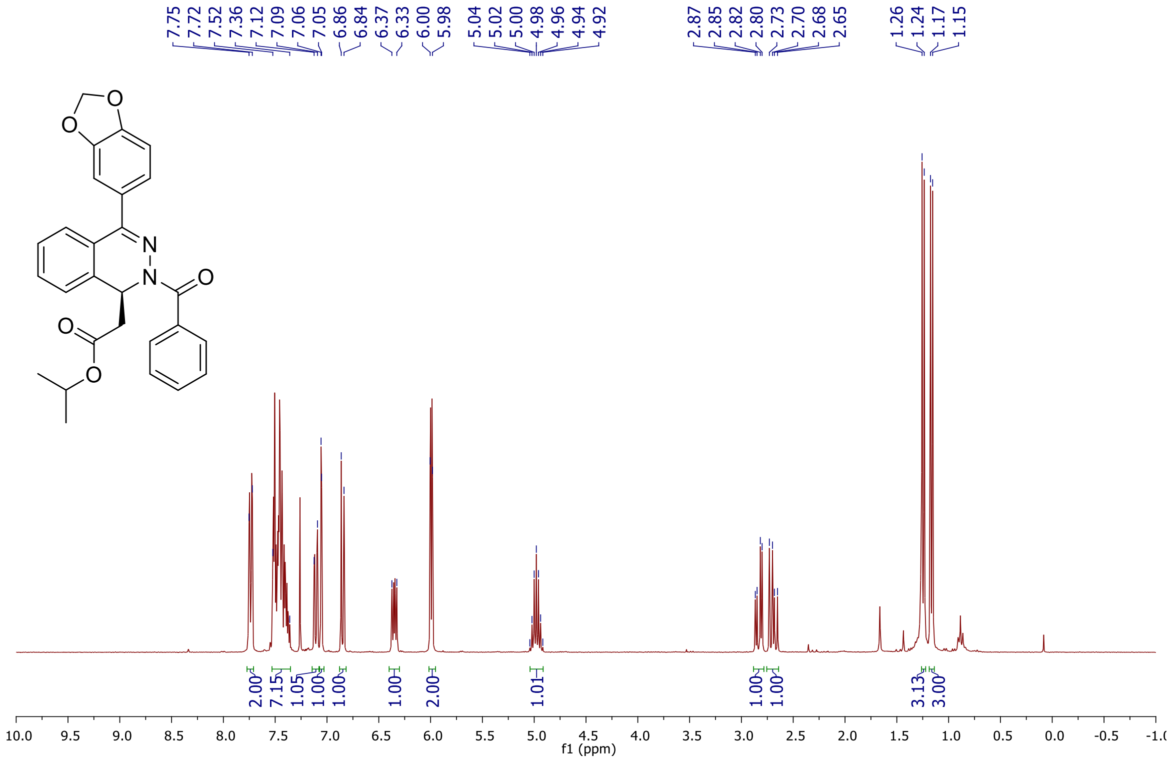

^{13}C NMR ($CDCl_3$, 75.5 MHz) of **(*S*)-8na**

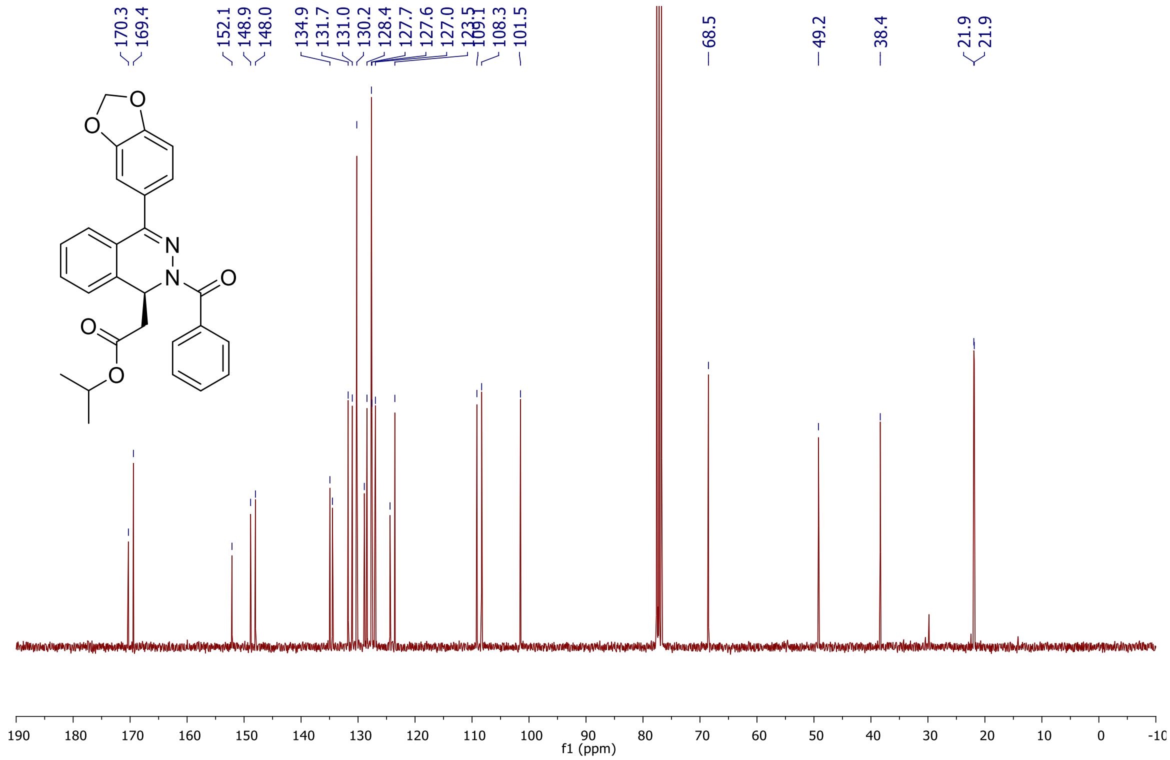

^{1}H NMR ($CDCl_3$, 300 MHz) of **(*S*)-8oa**

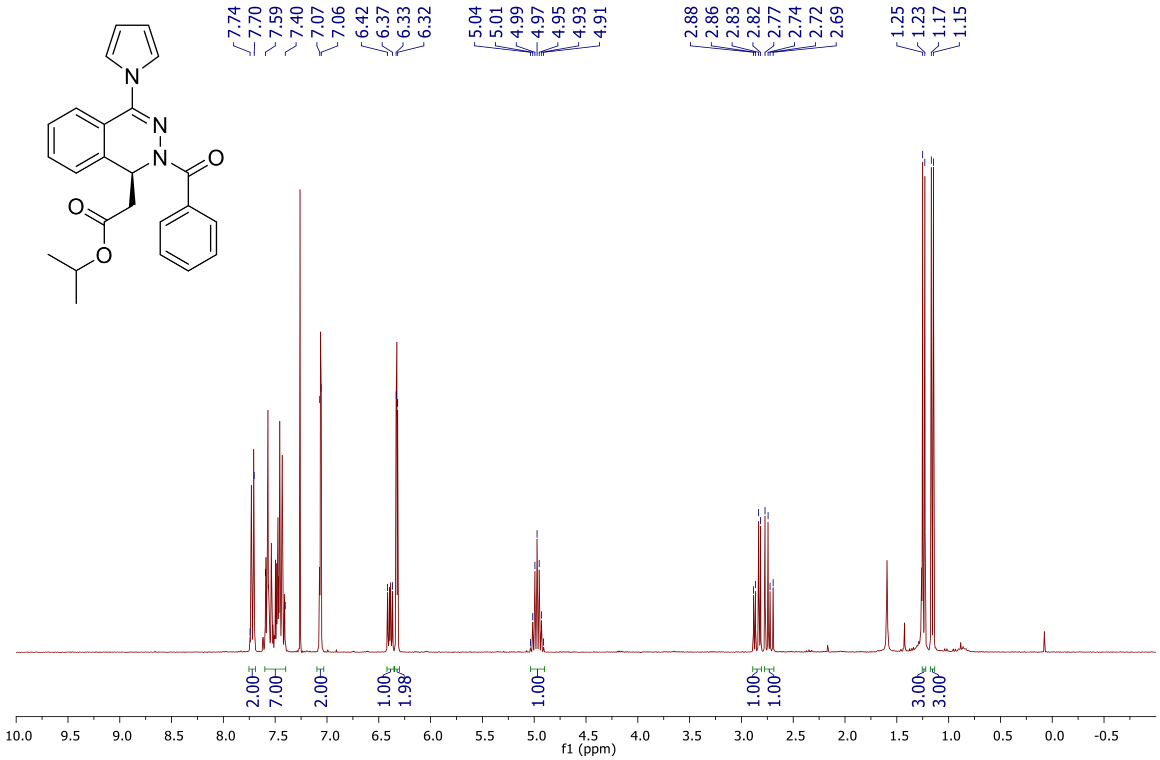

^{13}C NMR ($CDCl_3$, 75.5 MHz) of **(*S*)-8oa**

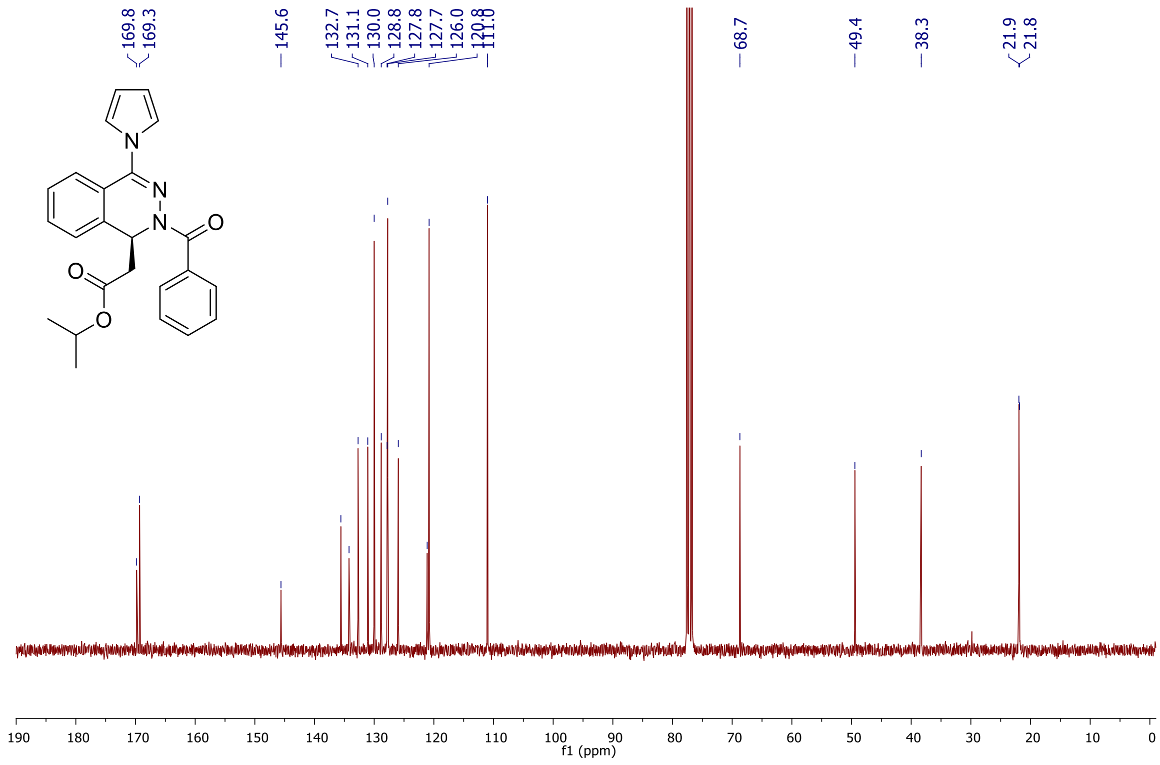

^{1}**H NMR** (CDCl$_3$, 300 MHz) of **(*S*)-8pa**

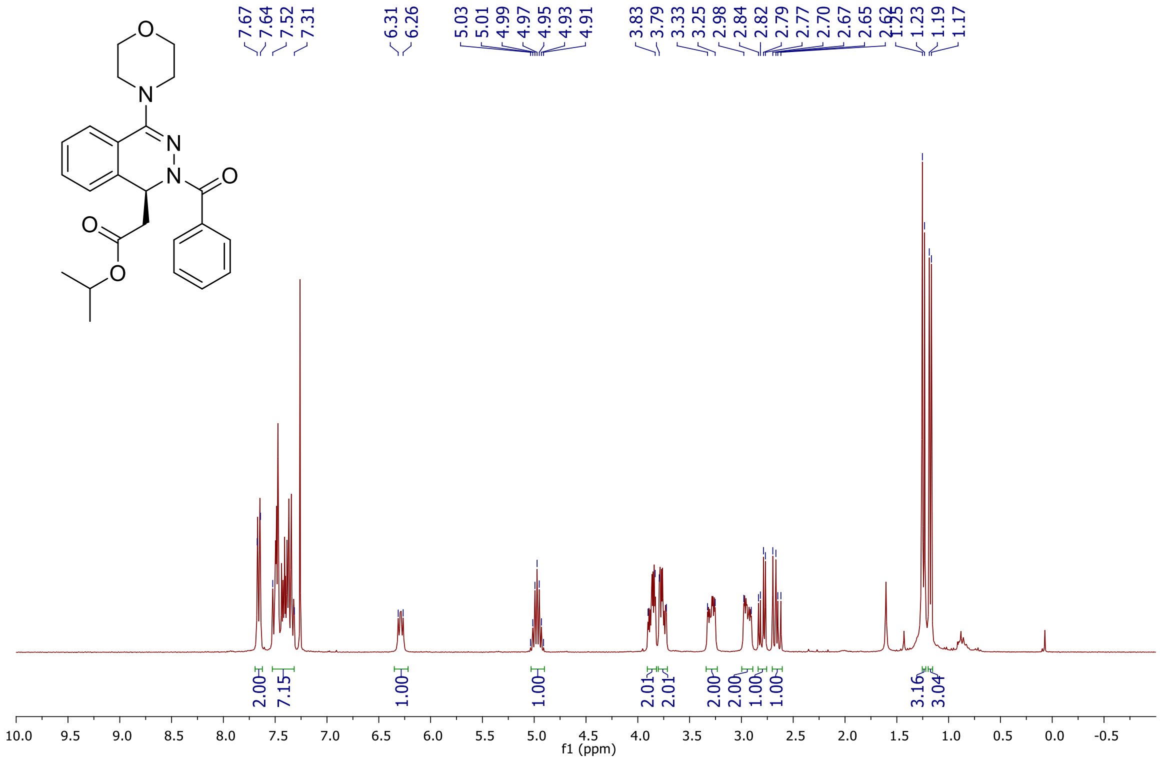

^{13}**C NMR** (CDCl$_3$, 126 MHz) of **(*S*)-8pa**

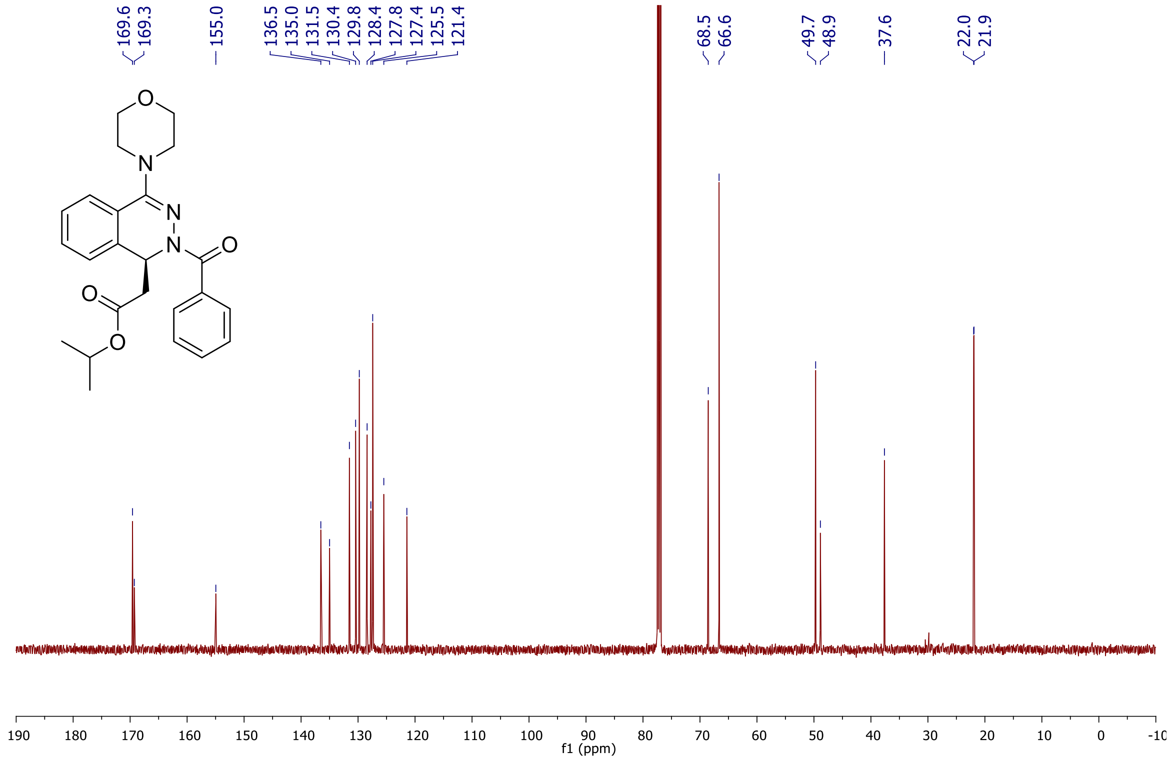

^{1}H NMR (CDCl$_3$, 300 MHz) of **(*S*,*S*)-8qa**

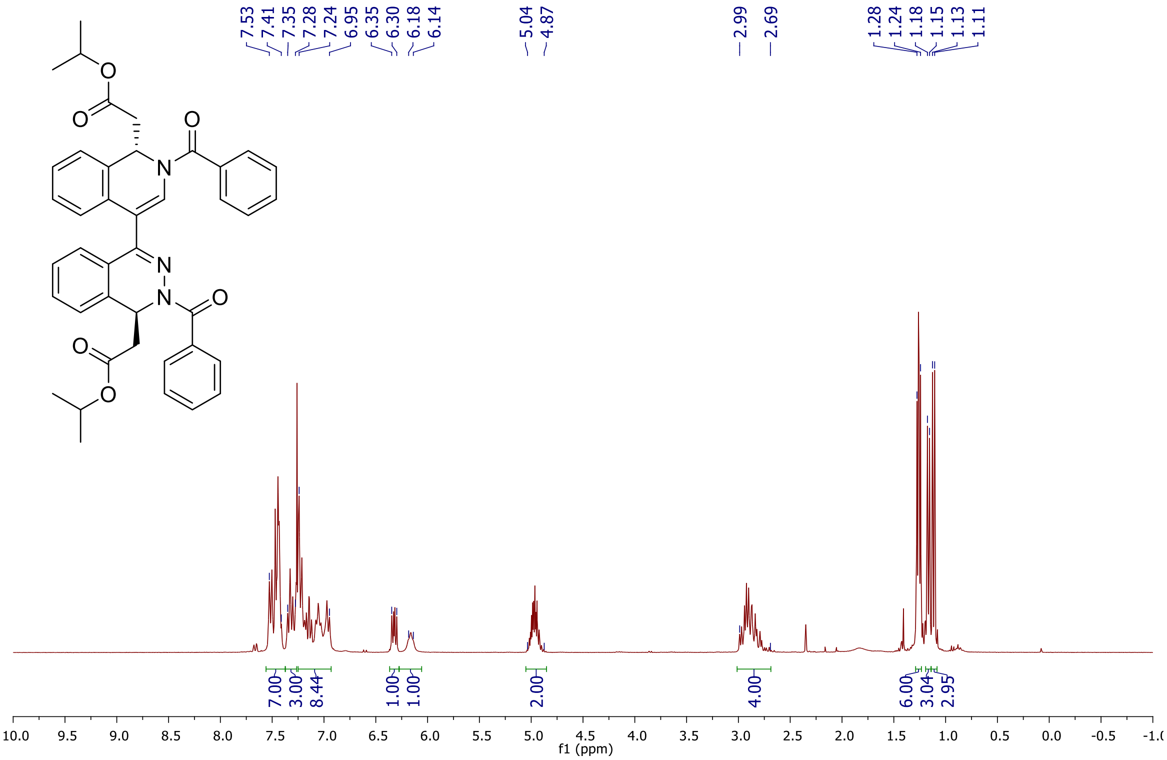

^{13}C NMR (CDCl$_3$, 75.5 MHz) of **(*S*,*S*)-8qa**

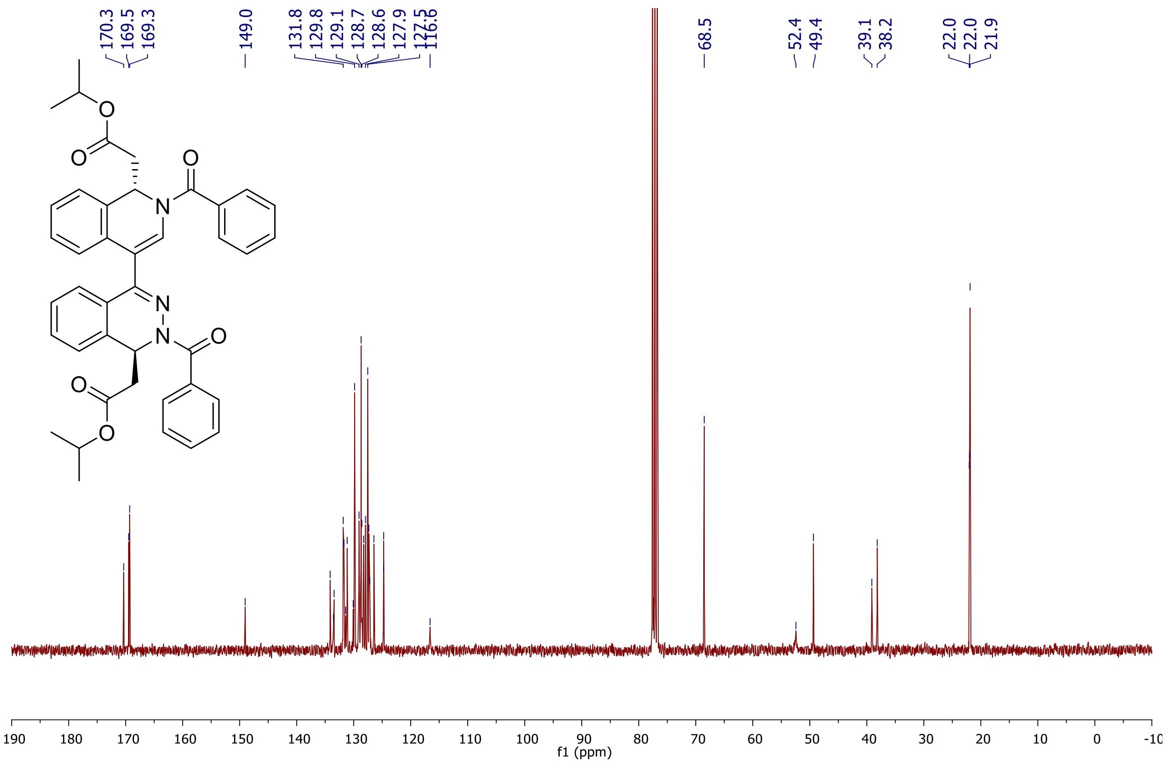

^{1}H NMR (CDCl$_3$, 300 MHz) of **(*S*)-14aa**

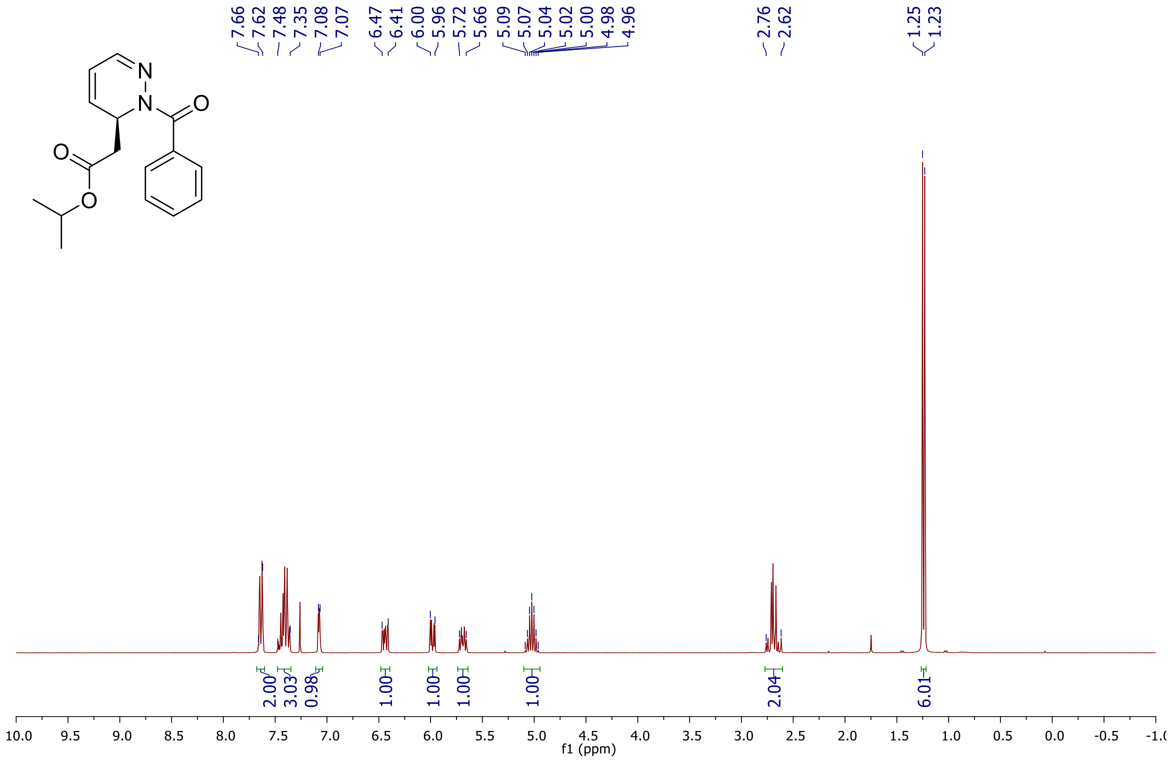

^{13}C NMR (CDCl$_3$, 75.5 MHz) of **(*S*)-14aa**

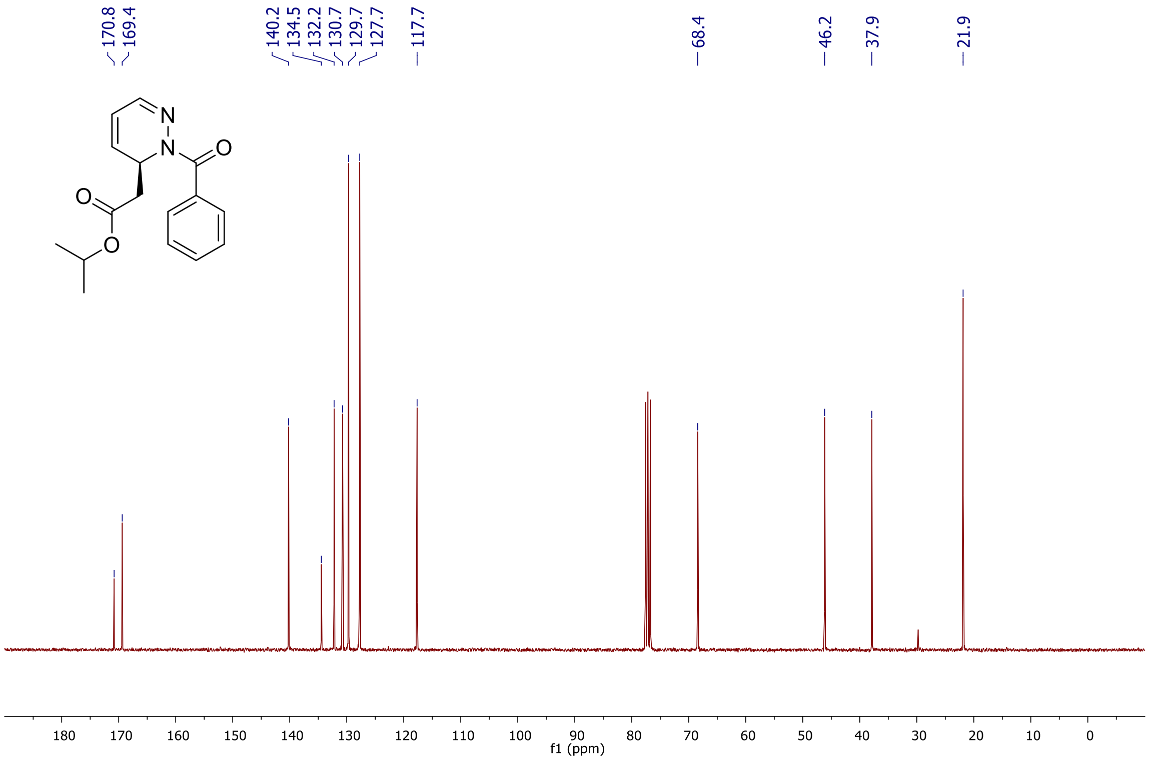

^{1}H **NMR** ($CDCl_3$, 300 MHz) of **(*S*)-14ae**

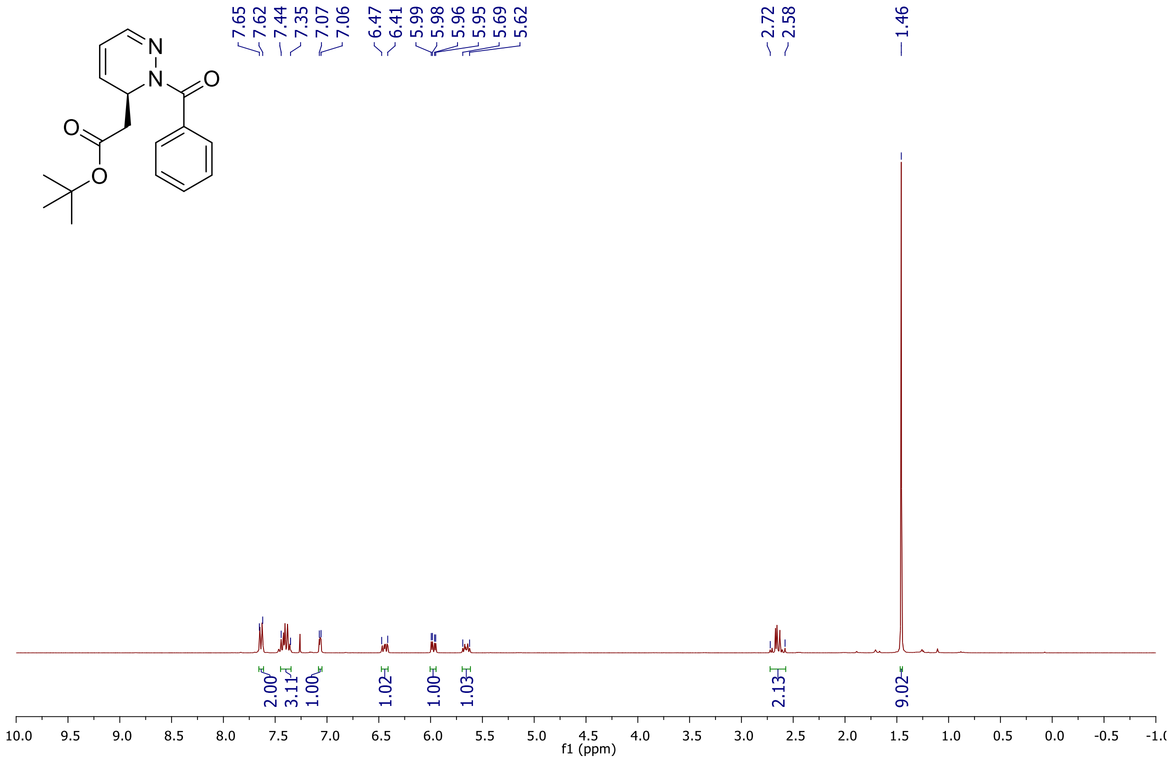

^{13}C **NMR** ($CDCl_3$, 75.5 MHz) of **(*S*)-14ae**

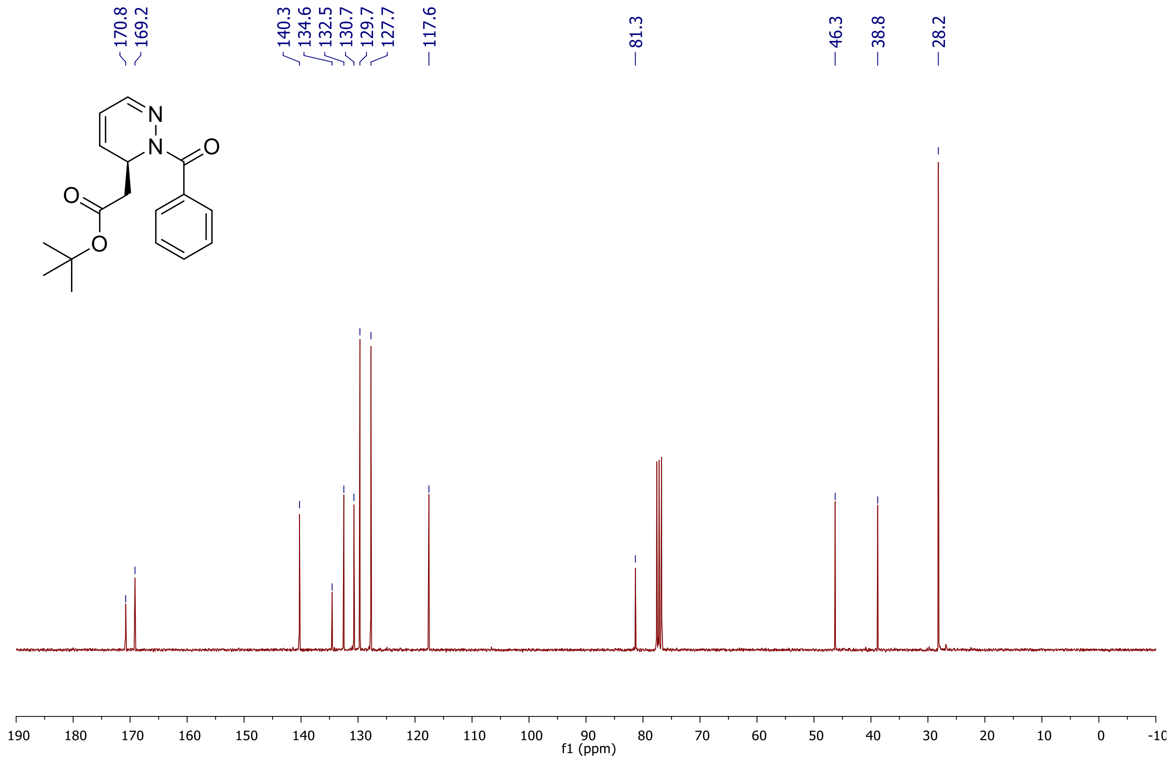

^{1}H NMR ($CDCl_3$, 300 MHz) of **(*S*)-15aa**

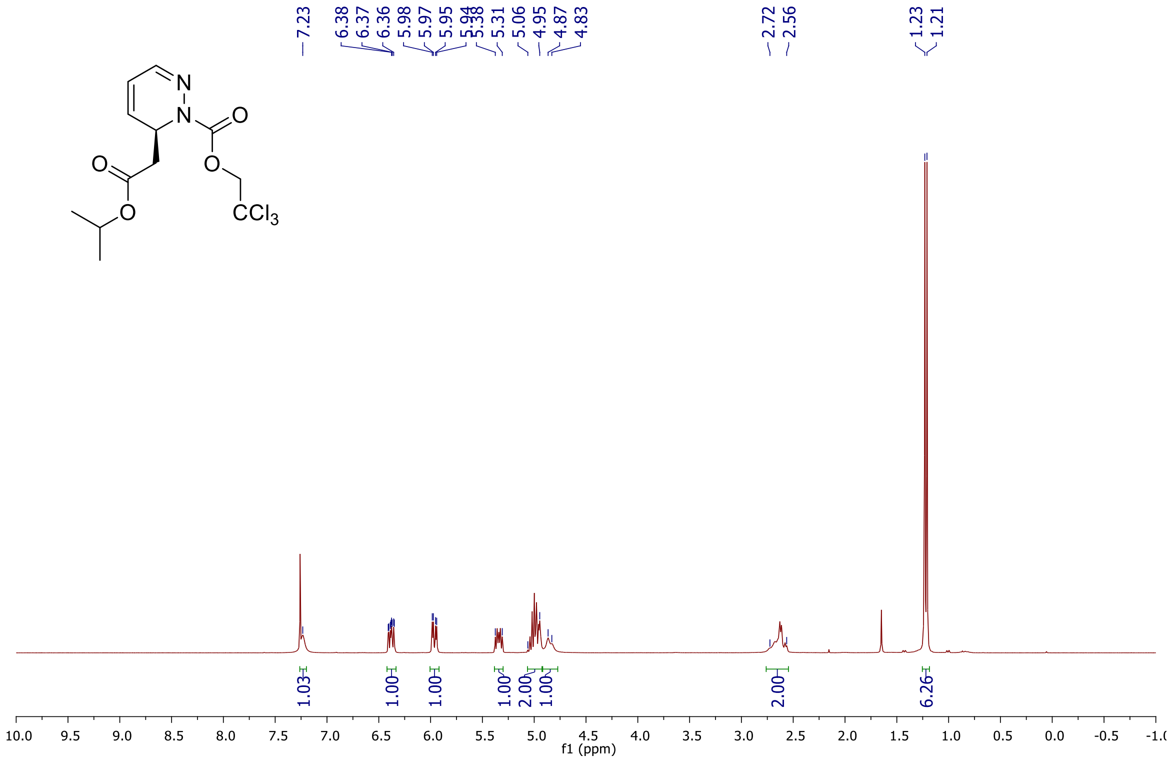

^{13}C NMR ($CDCl_3$, 126 MHz) of **(*S*)-15aa**

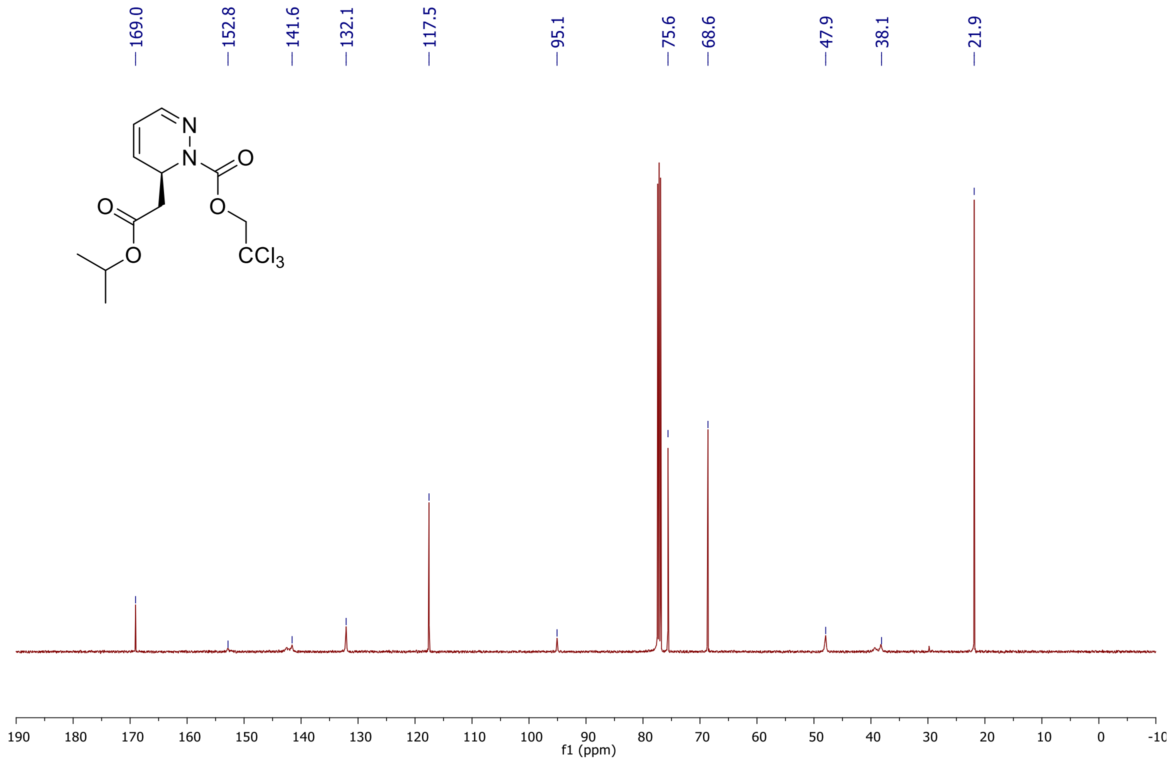

^{1}H NMR ($CDCl_3$, 300 MHz) of **(*S*)-15ad**

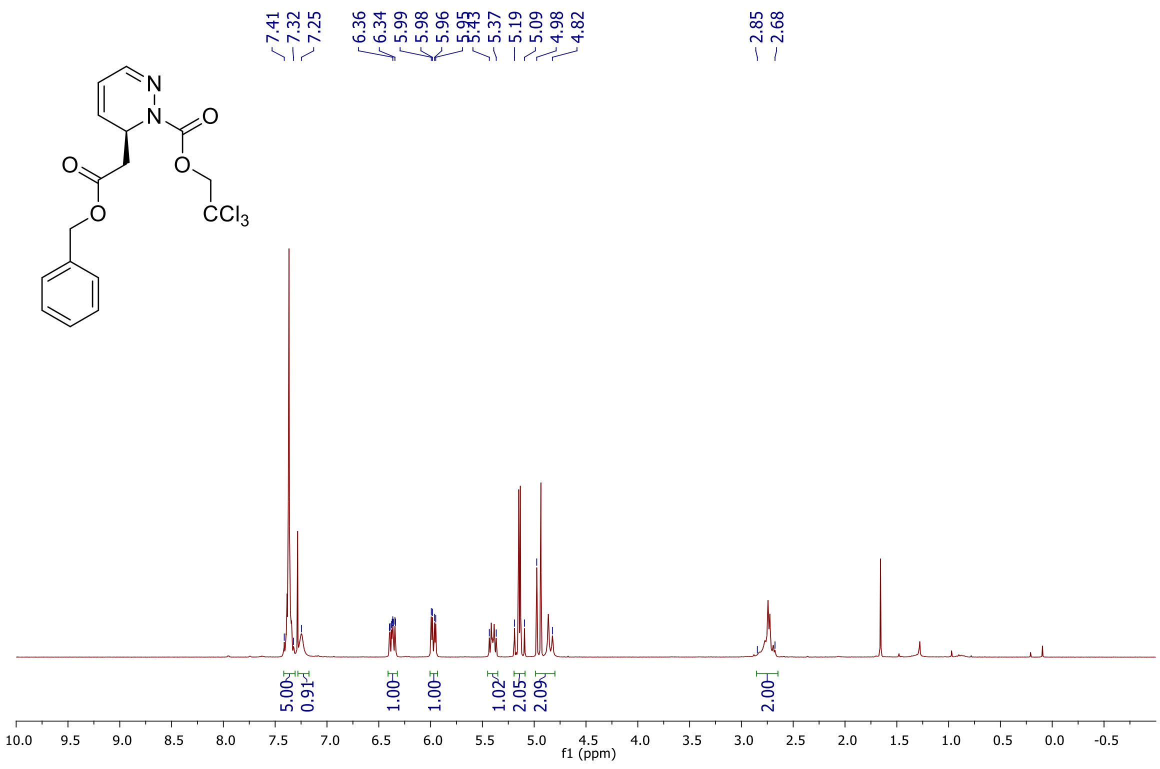

^{13}C NMR ($CDCl_3$, 126 MHz) of **(*S*)-15ad**

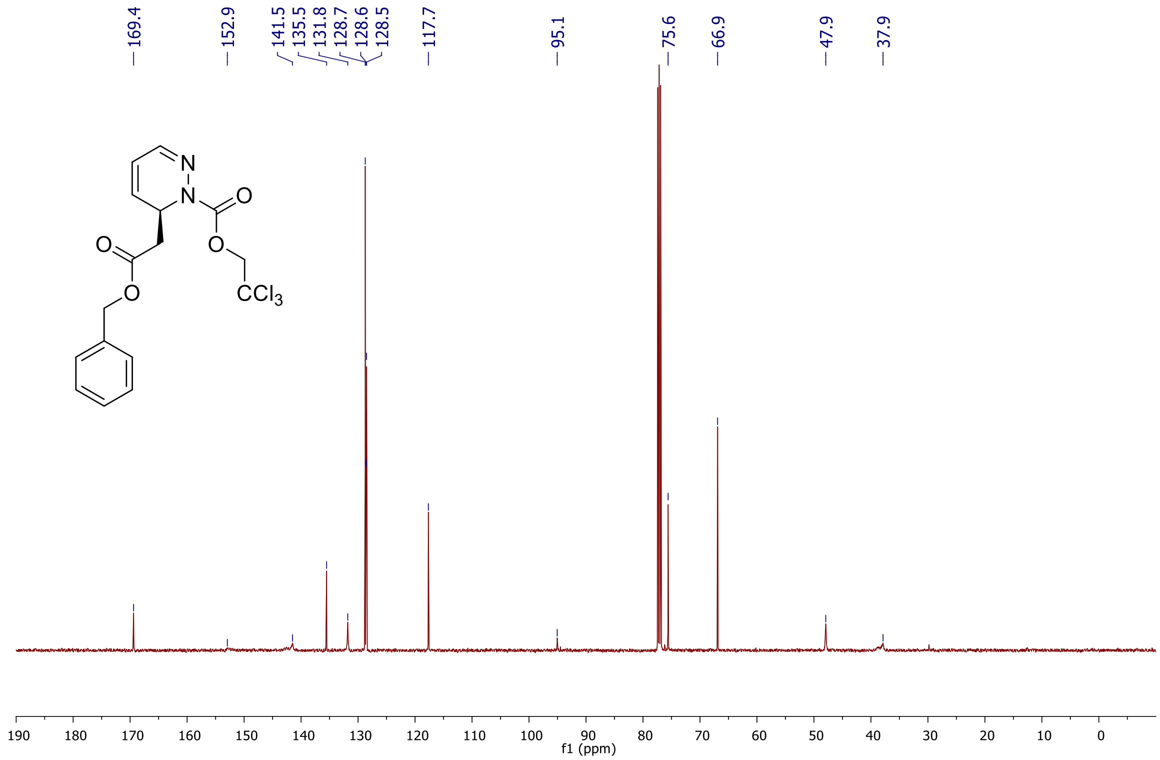

^{1}H NMR (CDCl$_3$, 300 MHz) of **(*S*)-15ae**

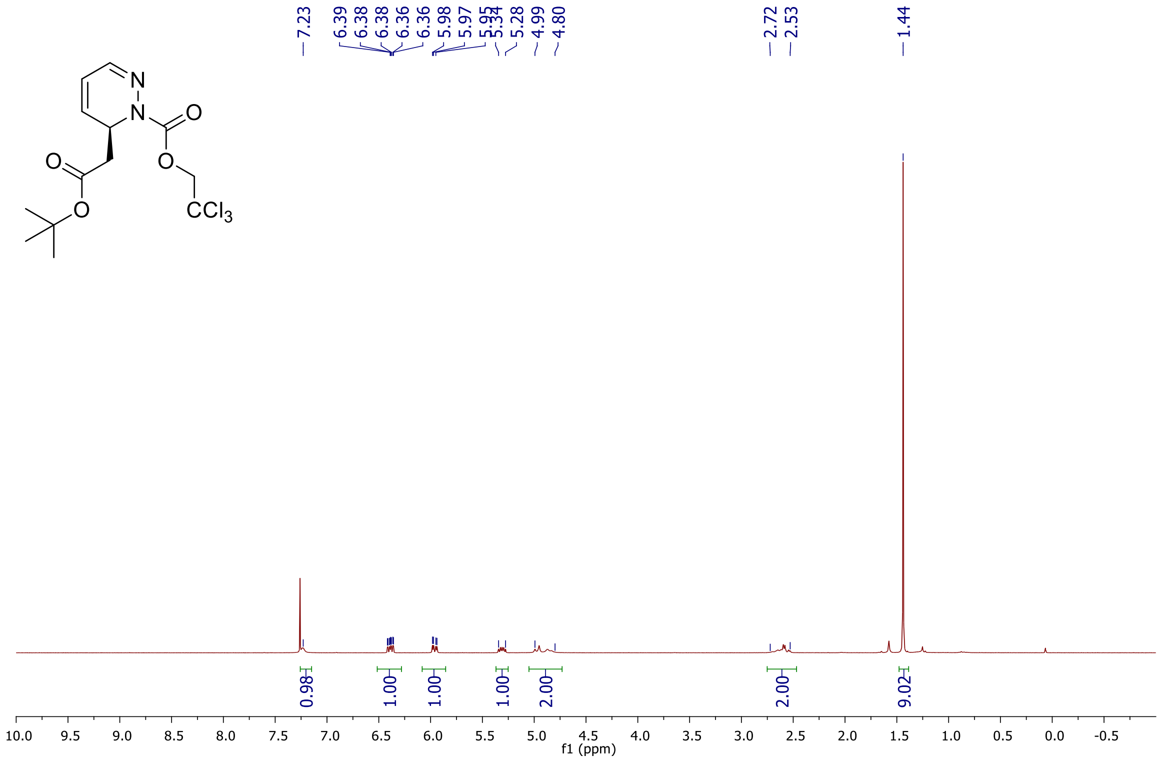

^{13}C NMR (CDCl$_3$, 126 MHz) of **(*S*)-15ae**

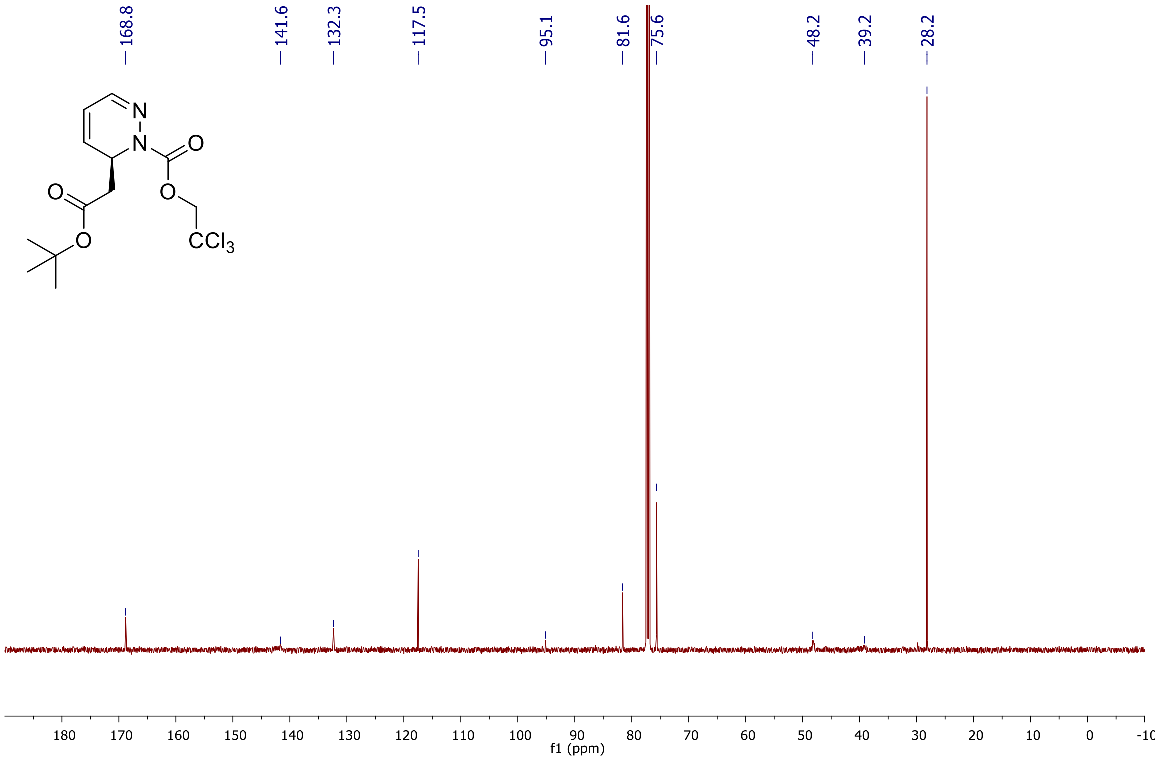

^{1}H NMR (Acetone-d^{6}, 300 MHz) of **(*S*)-16**

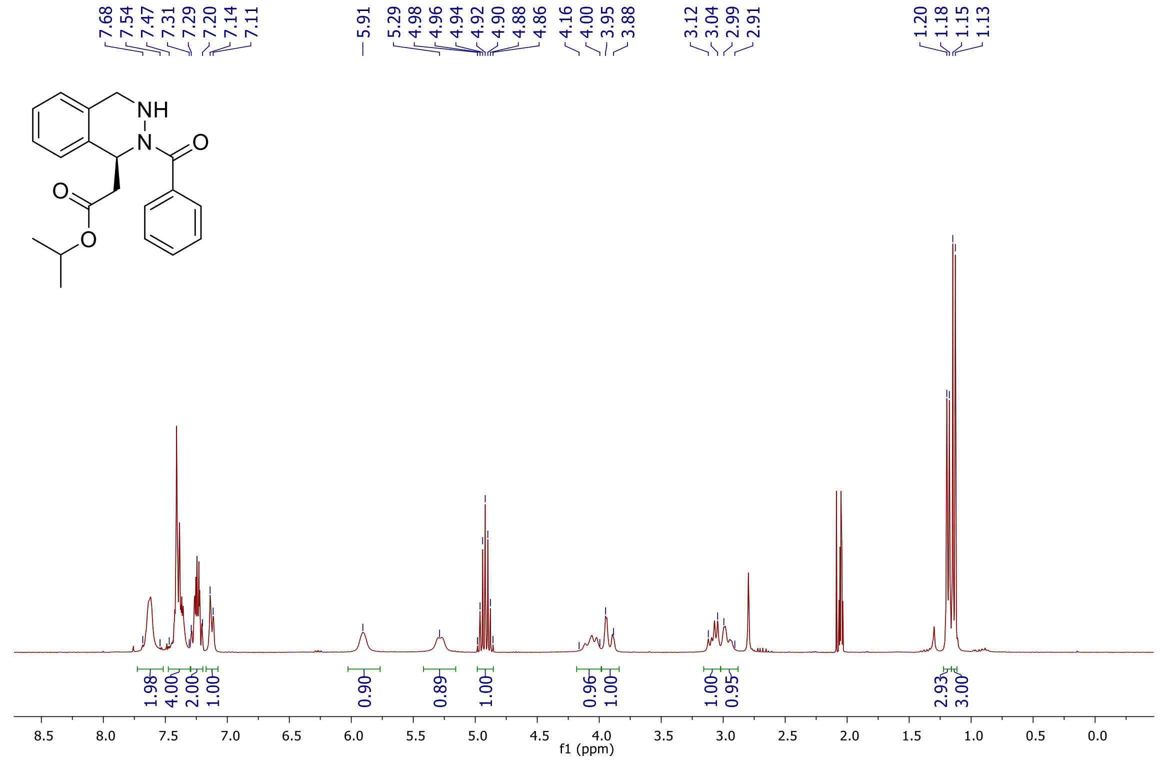

^{13}C NMR (CDCl$_3$, 75.5 MHz) of **(*S*)-16**

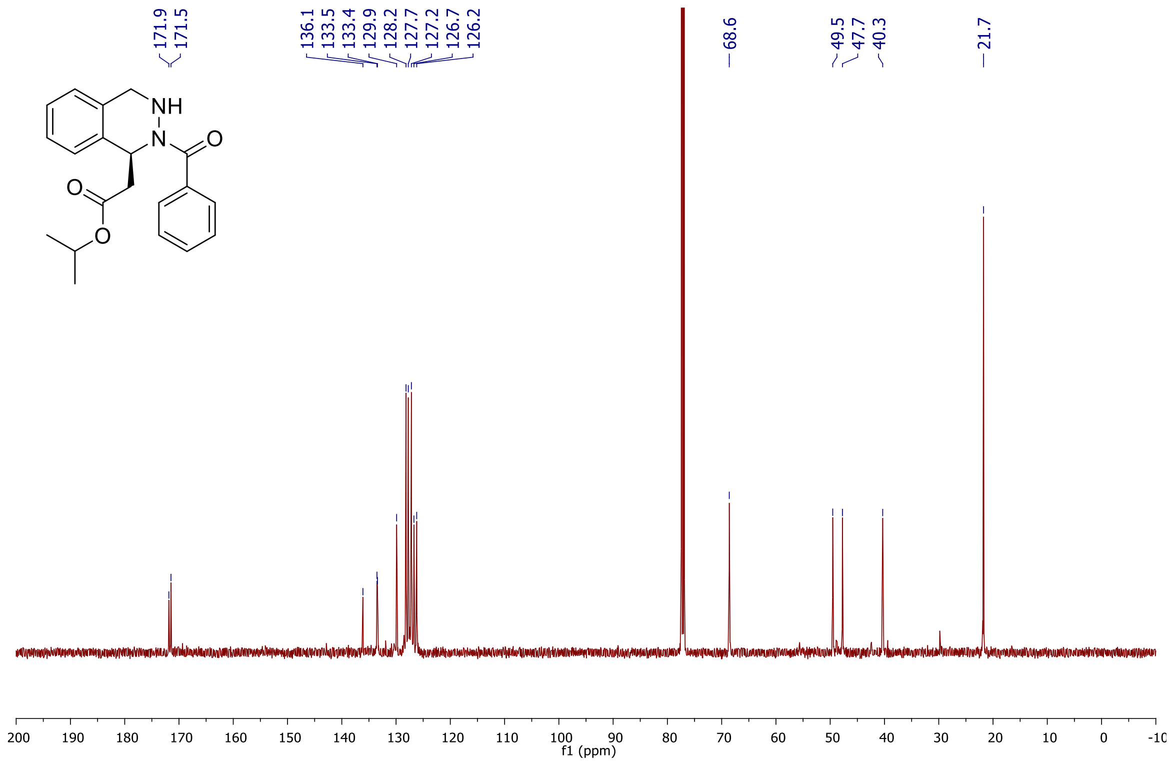

^{1}H NMR ($CDCl_3$, 300 MHz) of **(*S*)-17**

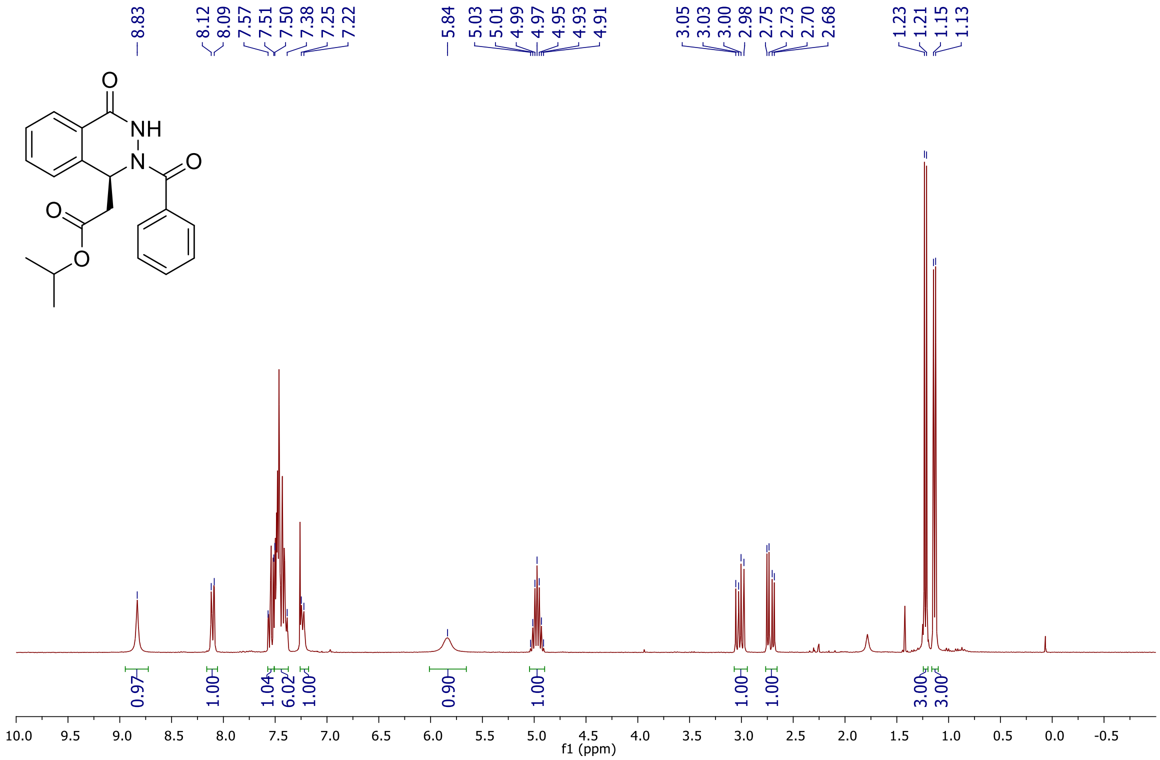

^{13}C NMR ($CDCl_3$, 75.5 MHz) of **(*S*)-17**

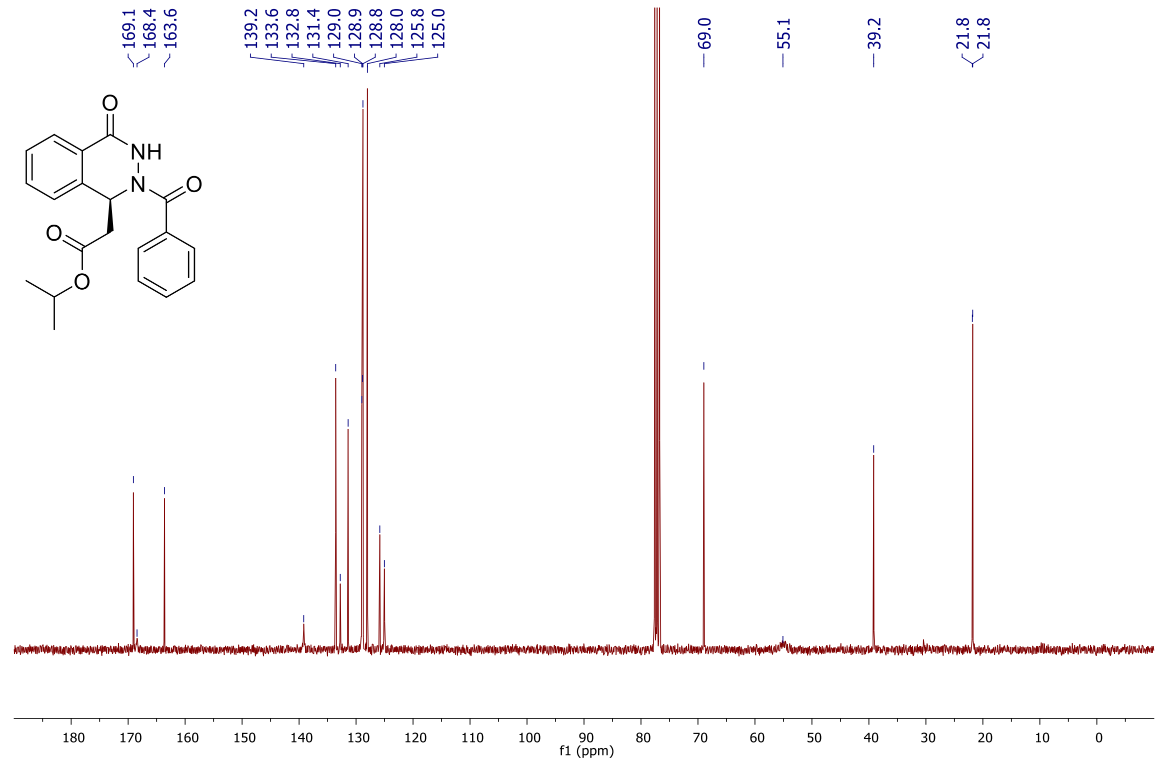

^{1}H NMR ($CDCl_3$, 300 MHz) of **(*S*)-18**

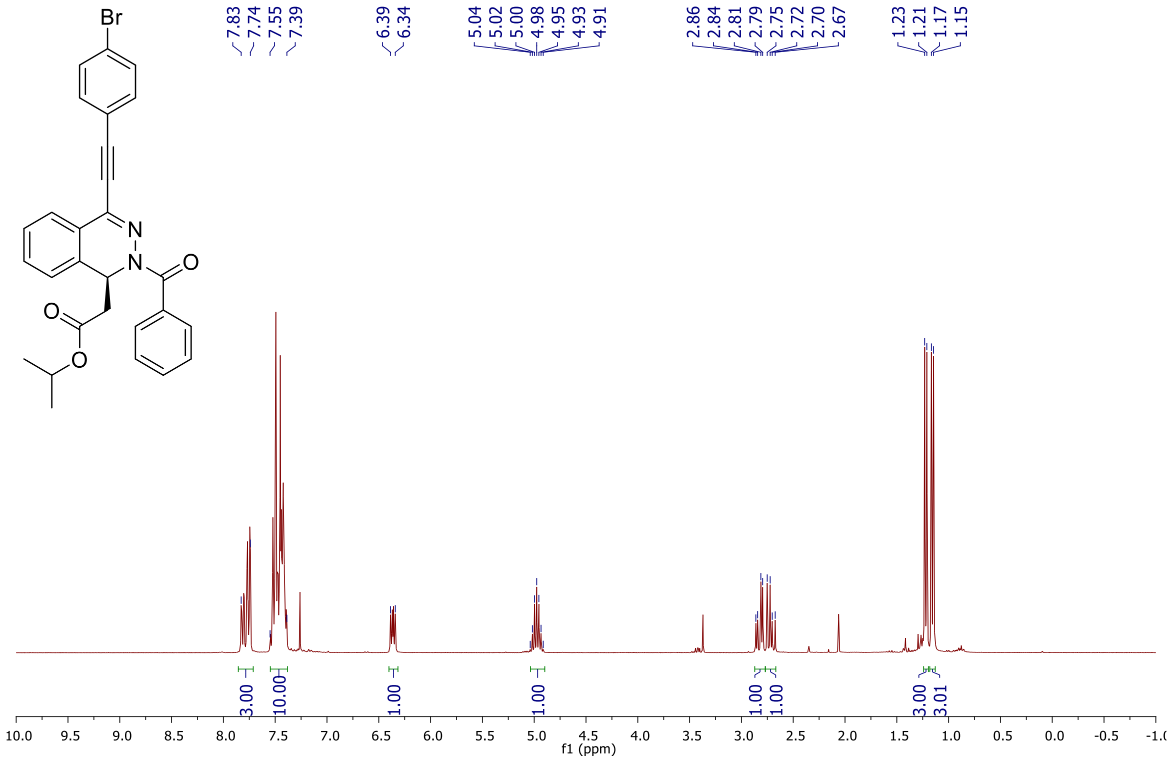

^{13}C NMR ($CDCl_3$, 75.5 MHz) of **(*S*)-18**

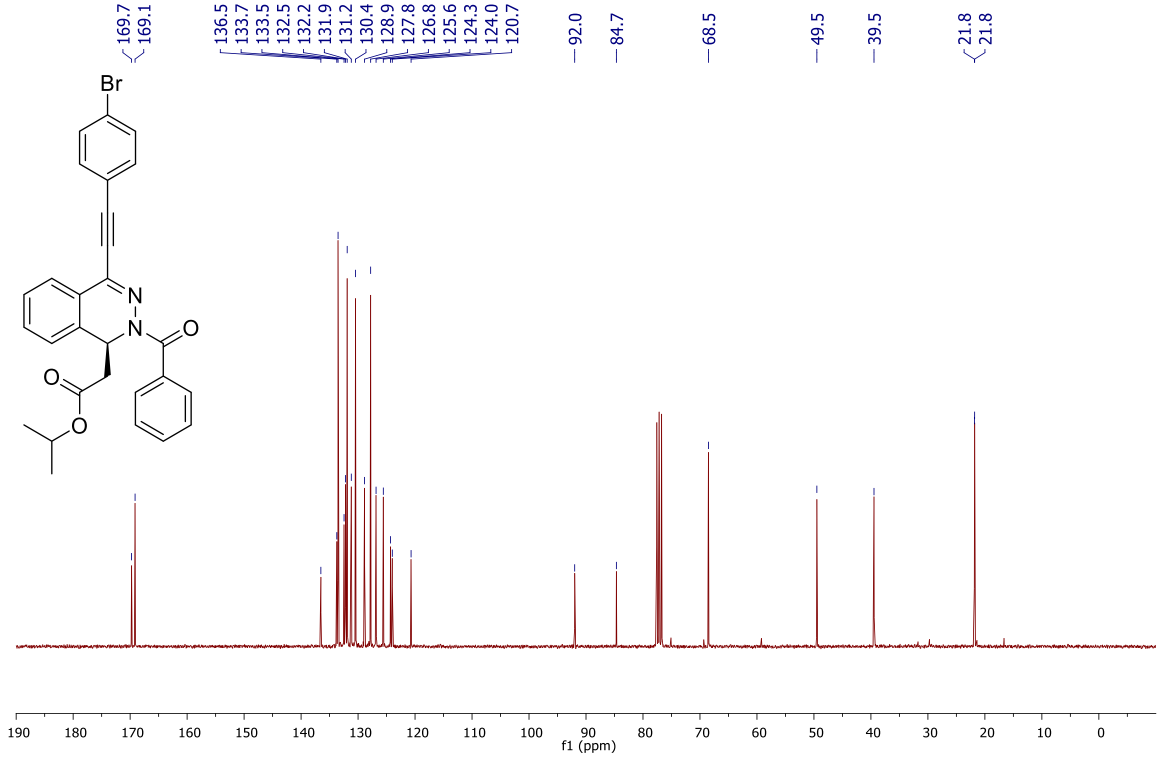

^{1}H NMR (CDCl$_3$, 300 MHz) of **(*S*)-19**

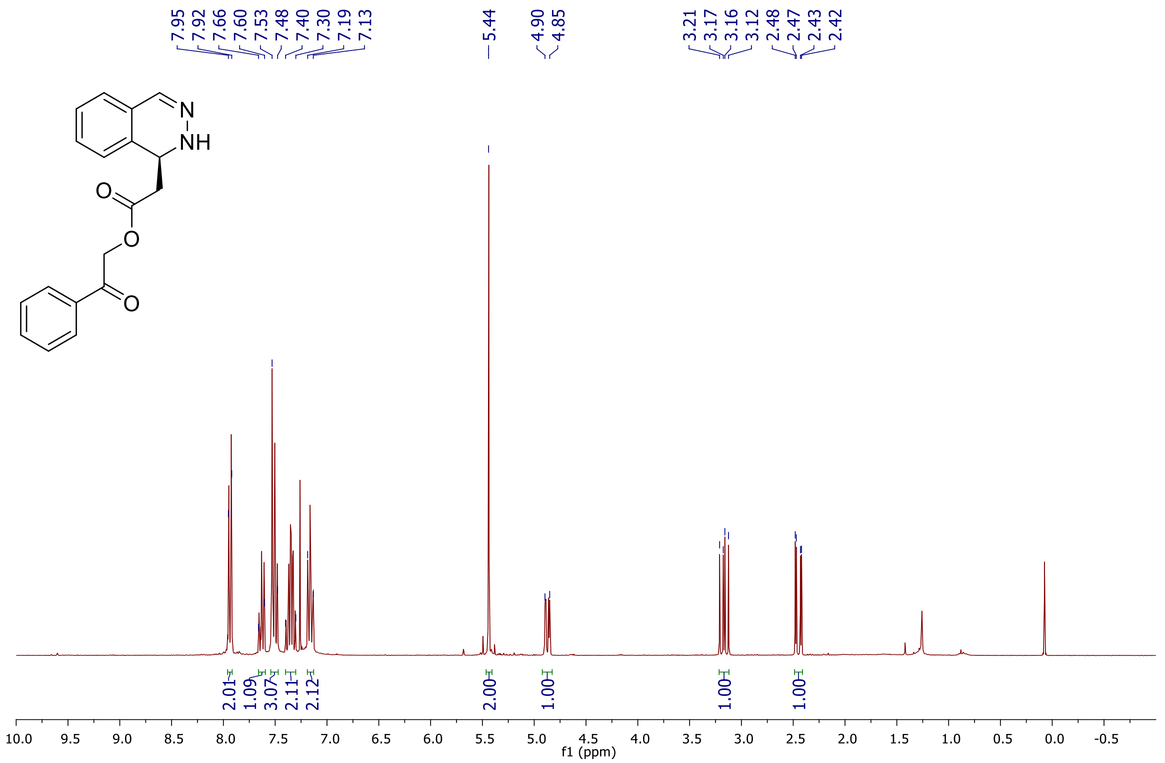

^{13}C NMR (CDCl$_3$, 75.5 MHz) of **(*S*)-19**

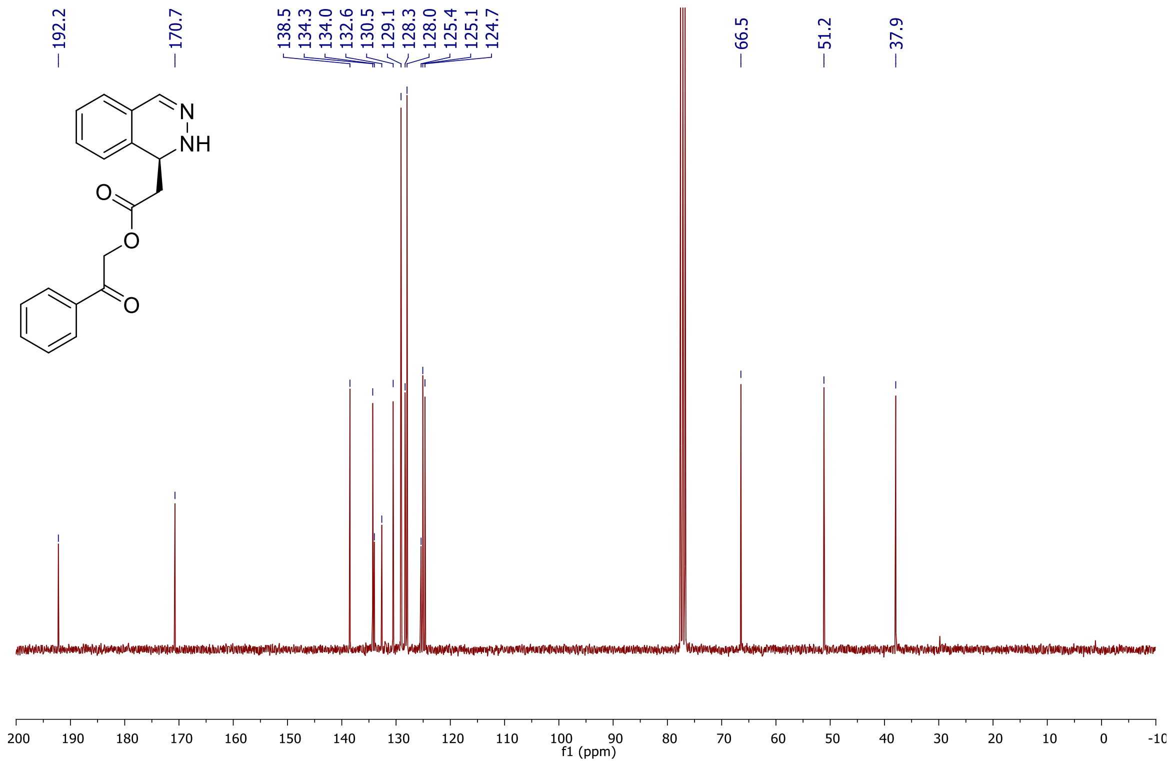

^{1}**H NMR** (CDCl$_3$, 300 MHz) of ***(S,S)*-20**

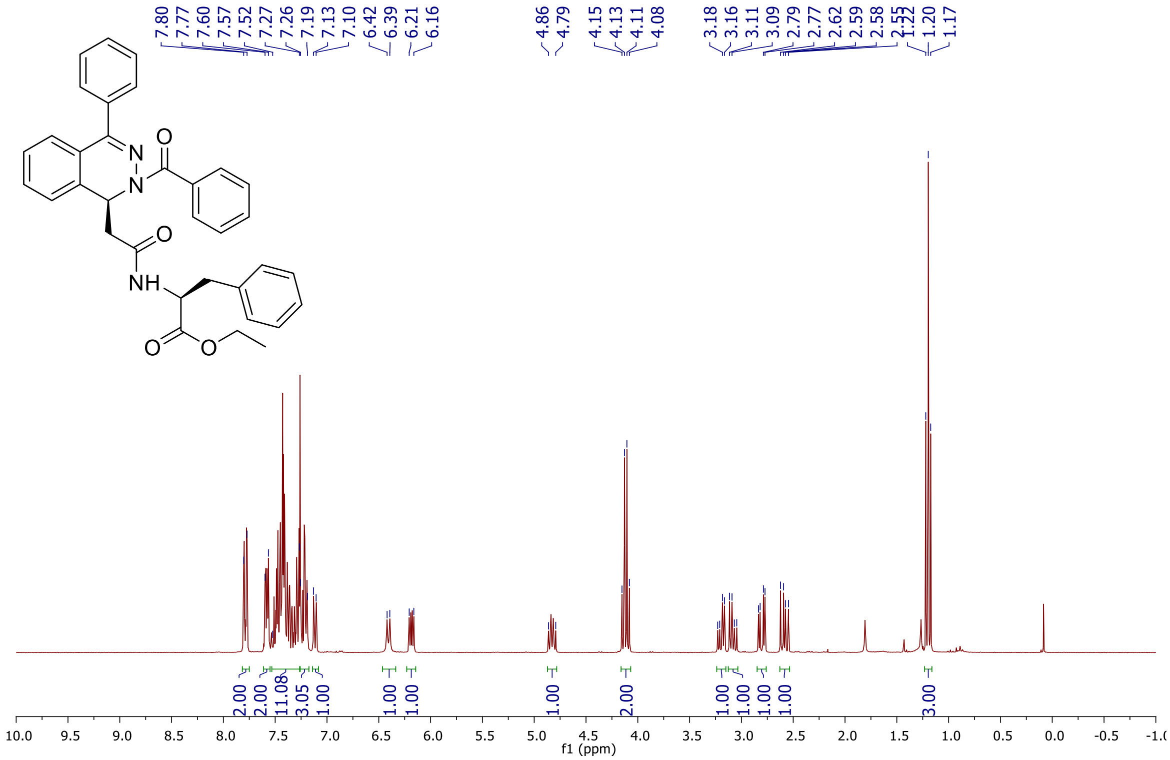

^{13}**C NMR** (CDCl$_3$, 75.5 MHz) of ***(S,S)*-20**

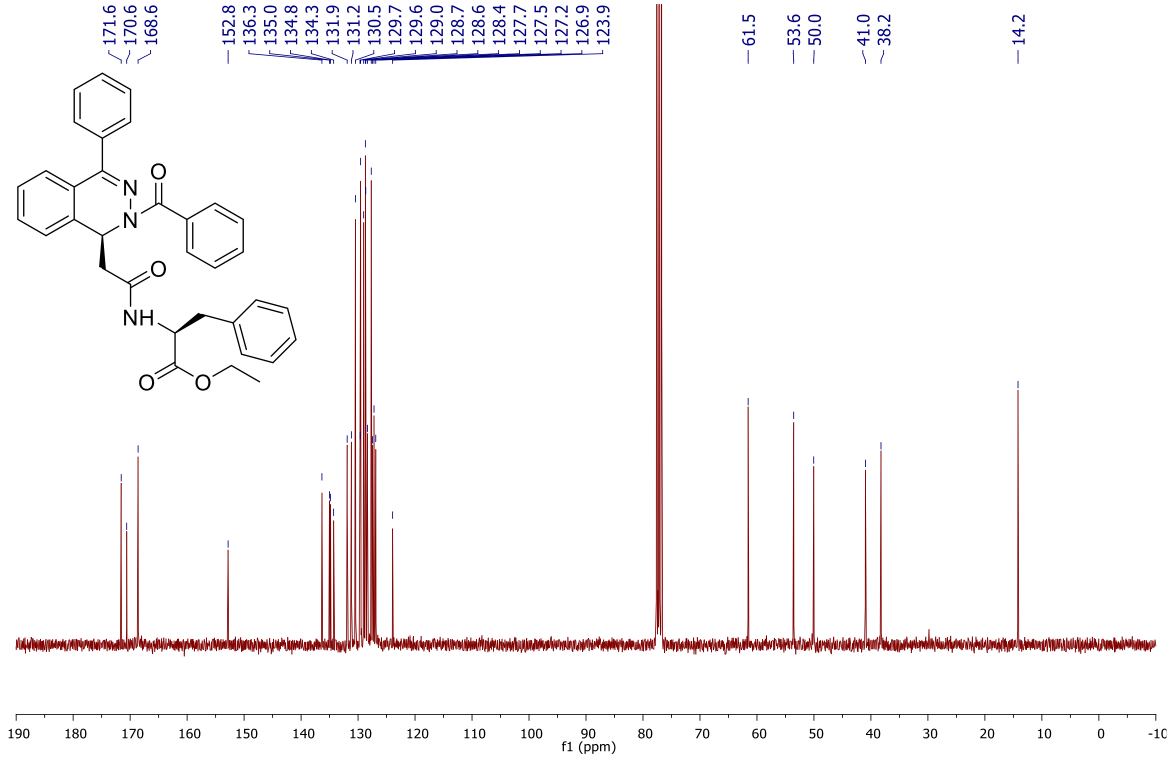

16. References

[1]Montavon, T. J.; Türkmen Y. E.; Shamsi, N. A.; Miller C.; Sumaria C. S.; Rawal V. H.; Kozmin S. A. [2+2+2] Cycloadditions of siloxy alkynes with 1,2-diazines: from reaction discovery to identification of an antiglycolytic chemotype. *Angew. Chem. Int. Ed*. **2013**, *52*, 13576-13579.

[2]Bunce, R. A.; Harrison, T.; Nammalwar B. Efficient synthesis of selected phthalazine derivatives. *Heterocycl. Commun*. **2012**, *18*, 123-126.

[3]Kessler, S. N.; Wegner H. A. One-pot synthesis of phthalazines and pyridazino-aromatics: a novel strategy for substituted naphthalenes. *Org. Lett*. **2012**, *14*, 3268-3271.

[4]Chang, S-M.; Jain, V.; Chen, T-L.; Patel, A. S.; Pidugu, H. B.; Lin, Y-W.; Wu, M-H.; Huang, J-R.; Wu, H-C.; Shah, A.; Su, T-L.; Lee, T-C. Design and synthesis of 1,2-bis(hydroxymethyl)pyrrolo[2,1-*a*]phthalazine hybrids as potent anticancer agents that inhibit angiogenesis and induce DNA interstrand cross-links. *J. Med. Chem.* **2019**, *62*, 2404-2418.

[5]Elbert, B. L.; Farley, A. J. M.; Gorman, T. W.; Johnson, T. C.; Genicot, C.; Lallemand, B.; Pasau, P.; Flasz, J.; Castro, J. L.; MacCoss, M.; Paton, R. S.; Schofield, C. J.; Smith, M. D.; Willis, M. C.; Dixon, D. J. C-H Cyanation of 6-ring *N*-containing heteroaromatics. *Chem. Eur. J.* **2017**, *23*, 14733-14737.

[6]Wenzel, A. G.; Jacobsen, E. N. Asymmetric catalytic Mannich reactions catalyzed by urea derivatives: enantioselective synthesis of *β*-aryl-*β*-amino acids. *J. Am. Chem. Soc.* **2002**, *124*, 12964-12965.

[7]Ford, D. D.; Lehnherr, D.; Kennedy, C. R.; Jacobsen, E. N. On- and off-cycle catalyst cooperativity in anion-binding catalysis. *J. Am. Chem. Soc*. **2016**, *138*, 7860-7863.

[8]Ratjen, L.; García-García, P.; Lay, F.; Beck, M. E.; List, B. Disulfonimide-catalyzed asymmetric vinylogous and bisvinylogous Mukaiyama aldol reactions. *Angew. Chem. Int. Ed*. **2011**, *50*, 754-758.

[9]Shi, M.; Liu, X-G. Asymmetric Morita-Baylis-Hillman reaction of arylaldehydes with 2-cyclohexen-1-one catalyzed by chiral bis(thio)urea and DABCO. *Org. Lett*. **2008**, *10*, 1043-1046.

[10]Sohtome, Y.; Takemura, N.; Takagi, R.; Hashimoto, Y.; Nagasawa K. Thiourea-catalyzed Morita–Baylis–Hillman reaction. *Tetrahedron* **2008**, *64*, 9423-9429.

[11]Sibi, M. P.; Itoh, K. Organocatalysis in conjugate amine additions. synthesis of β-amino acid derivatives. *J. Am. Chem. Soc.* **2007**, *129*, 8064-8065.

[12]Reisman, S. E.; Doyle, A. G.; Jacobsen, E. N. Enantioselective thiourea-catalyzed additions to oxocarbenium ions. *J. Am. Chem. Soc.* **2008**, *130*, 7198-7199.

[13]Zuend, S. J.; Jacobsen, E. N. Mechanism of amido-thiourea catalyzed enantioselective imine hydrocyanation: transition state stabilization via multiple non-covalent interactions. *J. Am. Chem. Soc.* **2009**, *131*, 15358-15374.

[14]Brown, A. R.; Uyeda, C.; Brotherton, C. A.; Jacobsen, E. N. Enantioselective thiourea-catalyzed intramolecular Cope-type hydroamination. *J. Am. Chem. Soc.* **2013**, *135*, 6747-6749.

[15]Lehnherr, D.; Ford, D. D.; Bendelsmith, A. J.; Kennedy, C. R.; Jacobsen, E. N. Conformational control of chiral amido-thiourea catalysts enables improved activity and enantioselectivity. *Org. Lett.* **2016**, *18*, 3214−3217.

[16]Kutateladze, D. A.; Strassfeld, D. A.; Jacobsen, E. N. Enantioselective tail-to-head cyclizations catalyzed by dual-hydrogen-bond donors. *J. Am. Chem. Soc.* **2020**, *142*, 6951-6956.

[17]Strassfeld, D. A.; Wickens, Z. K.; Picazo, E.; Jacobsen, E. N. Highly enantioselective, hydrogen-bond-donor catalyzed additions to oxetanes. *J. Am. Chem. Soc*. **2020**, *142*, 9175-9180.

[18]Bendelsmith, A. J; Kim, S. C.; Wasa, M.; Roche, S. P; Jacobsen, E. N. Enantioselective synthesis of α-allyl amino esters via hydrogen-bond-donor catalysis. *J. Am. Chem. Soc.* **2019**, *141*, 11414-11419.

[19]Fischer, T.; Bamberger, J.; García Mancheño, O. Asymmetric nucleophilic dearomatization of diazarenes by anion-binding catalysis. *Org. Biomol. Chem.* **2016**, *14*, 5794-5802.
